# Synthesis and *meta*-Amination of Pyridines via Multicomponent Cascade Reaction

**DOI:** 10.1021/acs.orglett.5c03509

**Published:** 2025-10-13

**Authors:** Telmo N. Francisco, Nuno R. Candeias, Samuel Guieu, Joana L. C. Sousa, Rafael F. A. Gomes, Carlos A. M. Afonso, Artur M. S. Silva, Hélio M. T. Albuquerque

**Affiliations:** † LAQV-REQUIMTE, Department of Chemistry, 201876 University of Aveiro, 3810-193 Aveiro, Portugal; ‡ Faculty of Engineering and Natural Sciences, Tampere University, Korkeakoulunkatu 8, 33101 Tampere, Finland; § Research Institute for Medicines (iMed.ULisboa), Faculty of Pharmacy, 70880 Universidade de Lisboa, Av. Prof. Gama Pinto, 1649-003 Lisbon, Portugal

## Abstract

We report a green, multicomponent cascade synthesis of 3-aminopyridines *via* [1,3′-bipyridin]-1-ium intermediates undergoing Zincke aminolysis with ammonia supported by experimental evidence and DFT calculations. The method offers broad scope (>20 examples), good functional group tolerance, and scalability in batch and flow procedures. Synthetic utility is demonstrated through conversion into 3-halo, 3-hydroxy, and 3-thiopyridines, expanding the access to *meta*-substituted pyridines through peripheral editing of the unprotected amino group.

## Graphical abstract

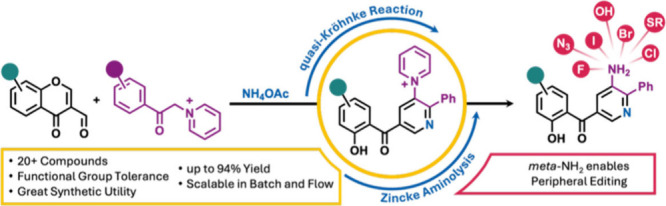

Pyridines are crucial in biological, material, and agrochemical sciences,^1–4^ topping the list of heterocycles in FDA-approved drugs,^5^ standing in 18% of the top-selling agrochemicals,^4^ and appearing as building blocks in ligands and functional materials.^6–8^ The electronic properties of pyridines hinder *meta*-substituted derivative preparation compared to *ortho*- and *para*-substituted isomers.^9,10^ Literature analysis (Figure S1) revealed that approximately 99% of the transformations for the synthesis of *meta*-aminopyridines already had a preconstructed pyridine ring, with a nitrogen-containing precursor in the *meta*-position. Around 89% of synthetic pathways involve nitro group reduction protocols preceded by nitration steps, usually requiring harsh conditions leading to low levels of chemo and regioselectivity for the *meta*-position (Scheme 1A).^11,12^ Other strategies based on nitrogen-containing precursors are also limited by low yields and use of specific and of limited availability reagents, with limited substrate scope.^13–17^ Modern approaches have been reported for *meta*-selective pyridine C–H functionalization relying on the use of directing groups, nondirected metalation, or temporary dearomatization.^10,18–21^ Important developments have been made by Studer regarding the *meta*-fluorination, hydroxylation, and nitration of pyridines upon temporary dearomatization strategy (Scheme 1B).^22–25^ Another elegant approach for the *meta*-selective pyridine halogenation involving Zincke imine intermediates was recently developed (Scheme[Fig sch1]C).^25–27^ However, these methods fail for direct *meta*-selective amination of pyridines with unprotected amino groups. Although C3-aminated pyridines with the NTsMe group have been achieved, this method has regioselectivity issues and cannot produce pyridines with C3 unprotected amino groups even after further transformations and using expensive iridium catalyst (Scheme 1C).^28^ An interesting option in this regard is the use of pyridine (pseudo)­halides through transition-metal catalytic amination.^29–31^ Nonetheless, this strategy is limited to tertiary and secondary amines, whereas the primary ones are extremely rare and underdeveloped.^29,30,32^ For the latter case, the use of Pd- or Cu-catalyzed procedures with aqueous ammonia contributed to this challenge (Scheme 1D), although very few examples of 3-aminopyridines have been produced and very careful ligand control must be considered.^33,34^ Despite these advances, straightforward access to pyridines functionalized with the “free” amino group (−NH_2_) at the *meta*-position (C-3) remains undiscovered.

**Scheme 1:**
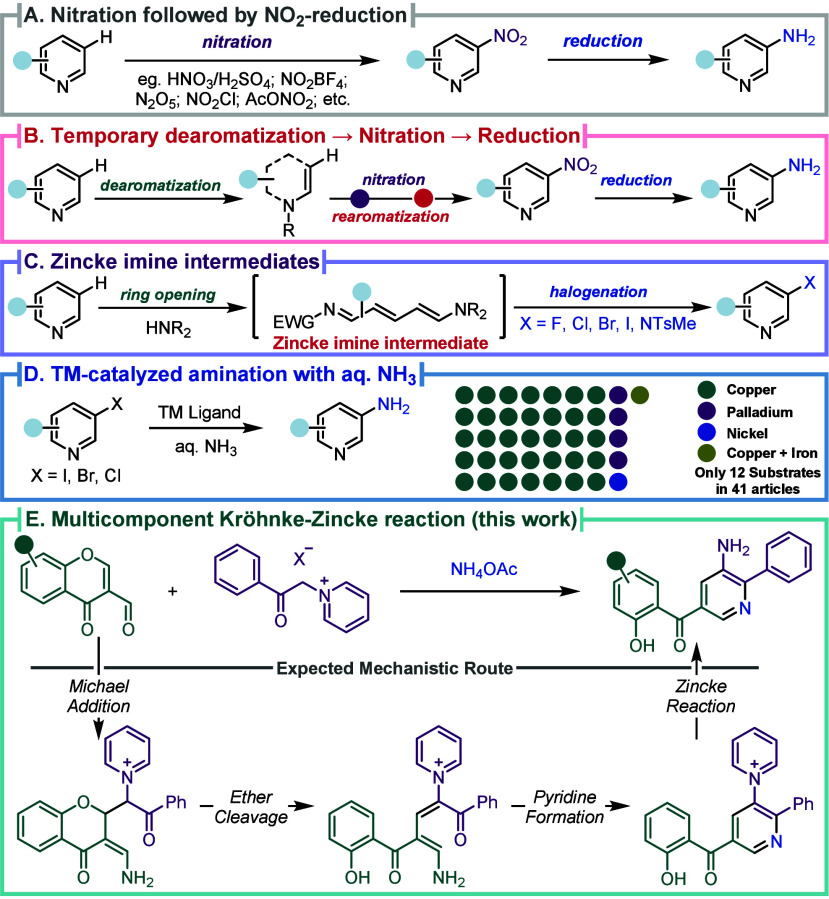
Depiction of (A) Nitration Methods, (B) Studer’s Pyridine Dearomatization, (C) *meta*-Functionalization *via* Zincke Imines, (D) TM-Catalyzed Amination, and (E) Multicomponent Cascade for *meta*-Amination of Pyridines

Herein, we report a multicomponent reaction (MCR) platform for the one-pot assembly of 3-aminopyridines from readily accessible materials, such as 3-formylchromones, pyridinium, and ammonium salts (Scheme 1E). The ammonium salt plays a double role in the reaction mechanism, being involved in the construction of the pyridine moiety and as the initiator of the Zincke reaction. The key feature of this cascade reaction is that the chromone ether-cleavage enables the aromatization of the pyridine ring without removal of the pyridinium, granting a subsequent Zincke reaction to complete the synthesis of 3-aminopyridines.

The initial experiments to efficiently deploy this MCR involved extensive optimization (Scheme 2C, Tables S1–S3) using 3-formylchromone **1a** and pyridinium salt **2a** as substrates, synthesizing 3-aminopyridine **3a** in 90% yield with the optimal conditions (Scheme 2A), as confirmed through crystal X-ray diffraction (Scheme 2B). Preliminary studies demonstrated that the reaction was more effective under microwave (MW) irradiation, although it was not essential for obtaining 3-aminopyridine **3a**. NH_4_OAc proved to be the best nitrogen source, with (NH_4_)_2_CO_3_ and NH_4_HCO_2_ as suitable alternatives. The MCR demonstrated compatibility with a wide range of protic and nonprotic solvents, including H_2_O, with EtOH, THF, and toluene as the best options. It is noteworthy that MeOH exhibited comparable results to the reaction using EtOH; however, the formation of imine **S2** (Figure S3) as a byproduct resulted in lower yields.

**Scheme 2:**
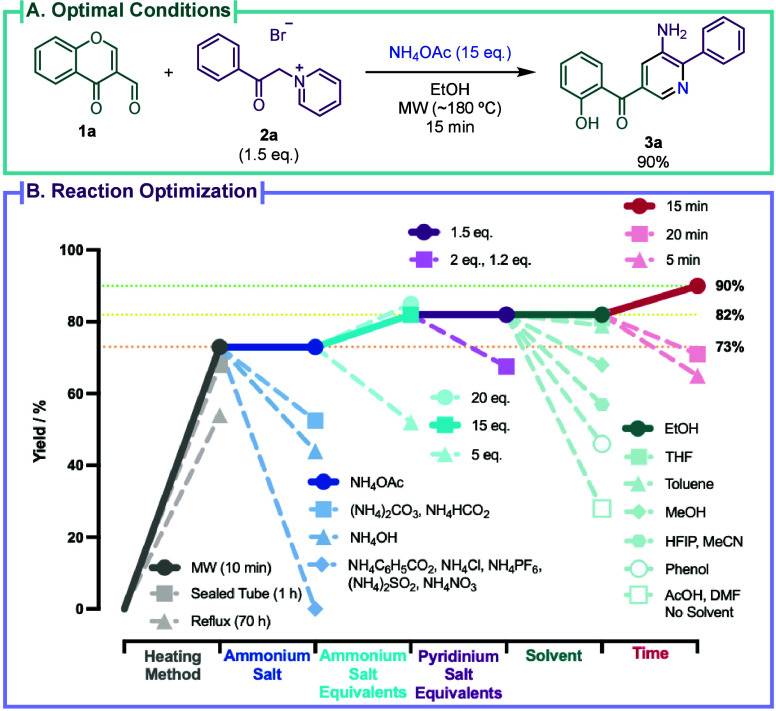
(A) Optimal Conditions for 3-Aminopyridine **3a** Synthesis; (B) Graphical Optimization Summary

Having set the optimal reaction conditions, we investigated the scope of this MCR platform by evaluating various 3- formylchromones **1** and pyridinium salts **2** (Scheme 3). The methodology was compatible with electron-donating (EDGs), electron-neutral, and electron-withdrawing groups (EWGs) at different positions of chromone components **1a**–**j**, with the corresponding 3-aminopyridines **3a**–**j** obtained in 37–90% yield (Scheme 3A). Even sterically encumbered substrates proved successful, despite the lower yield for **3j**. Notwithstanding, this methodology was incompatible with two chromone substrates, **1k** and **1l** (Scheme 3C). The presence of a COOH group in the 3-formylchromone **1k**, led to the formation of the decarboxylated 3-aminopyridine **3a**. The presence of a CF_3_ group as substituent, **1l**, did not produce the expected 3-aminopyridine, even with THF or toluene; instead, amidst multiple side reactions and/or decomposition, the 3-aminopyridne **4** was isolated in 5% yield, resulting from the reaction with EtOH under alkaline conditions after the CF_3_ group was anionically activated (Scheme 3C).^35^ Following the scope studies, we surveyed the viable substitutions that could be applied to pyridinium salts **2**, leading to the synthesis of 3-aminopyridines **3k**–**s** in 32–94% yield (Scheme 3B). The methodology was well-suited for EDGs, neutral, and EWGs, tolerating the presence of sensitive COOH (**3o**), CN (**3q**), and NH_2_ (**3p**) groups. Sterically demanding groups such as naphthalene (**3t**) and pyrene (**3u**) are better tolerated in pyridinium salts than in 3-formylchromone partners. The reaction was also compatible with *O*-, *N*-, and *S*-heterocycles such as dioxane, pyridine, and thiophene delivering pyridines **3v**–**x** in 32–83% yield (Scheme 3B). When using the pyridinium salt **2p**, the coumarin moiety would engage a divergent reactivity at the *ortho*-position of the pyridinium leading to the formation of pyrido indolizine **5** (Scheme 3C). The presence of the COOH moiety resulted in the formation of the corresponding ester **S4** (Figures S96 and S97) through reaction with EtOH, which could be hampered by replacing the EtOH by THF. Similarly, when synthesizing 3-aminopyridine **3p**, (NH_4_)_2_CO_3_ was used as the nitrogen source, to avoid formation of amide **S5** (Figures S98 and S99) observed when NH_4_OAc was used. The presence of OH groups in the aromatic ring (**2q**) was incompatible with this procedure due to side reactions and/or decomposition (Scheme 3C).

**Scheme 3:**
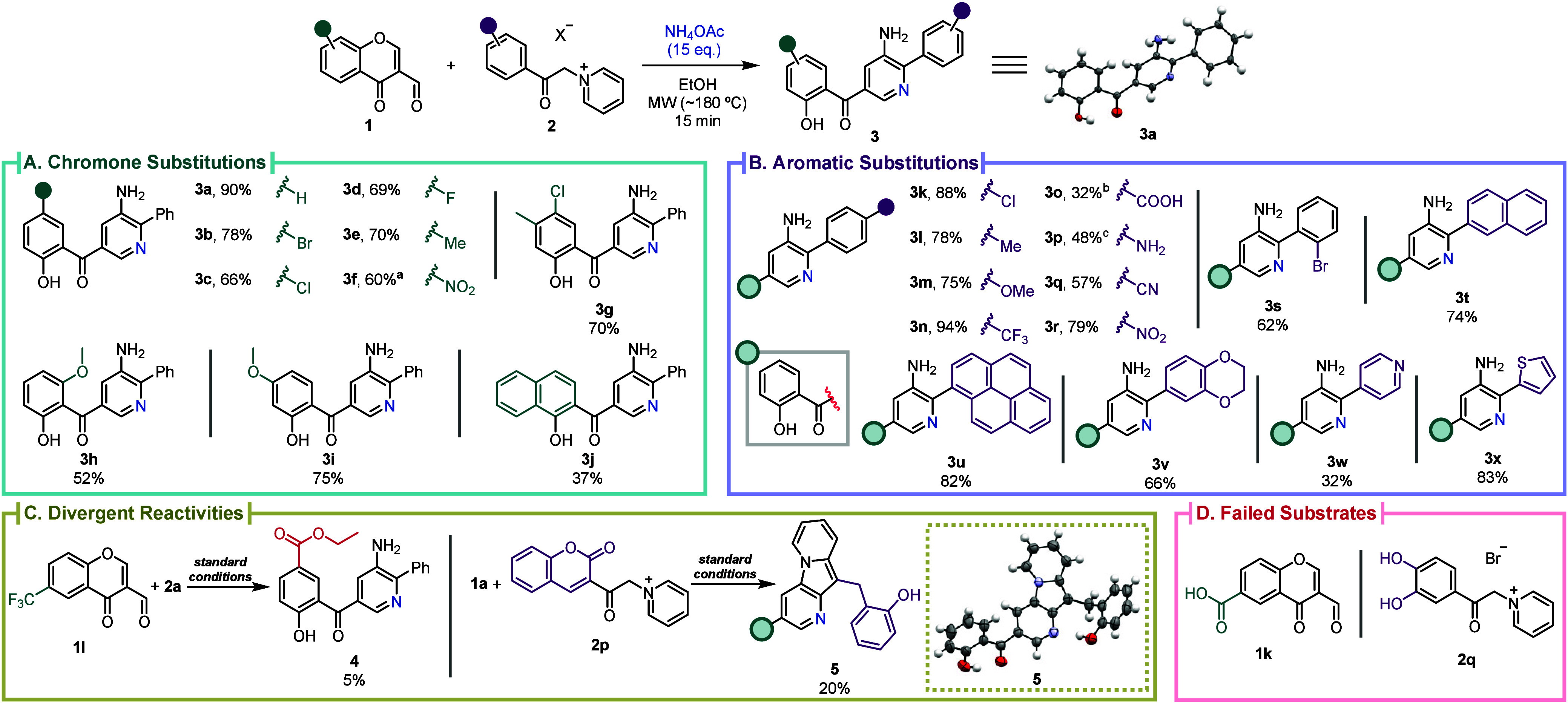
Scope of 3-Aminopyridines **3** from 3-Formylchromones **1** and Pyridinium Salts **2** [Fn sch3-fn1] and Structure of **3a** and **5** via Single-Crystal X-ray Diffraction^e^

a
**1** (0.459 mmol); **2** (1.5 equiv); NH_4_OAc (15 equiv); EtOH (1 mL); 180 °C (MW), 15 min.
b Toluene as solvent.
c THF as solvent.
d (NH_4_)_2_CO_3_ as nitrogen source.
e Structure of **3a** and **5** via single-crystal X-ray diffraction. Thermal ellipsoids are shown at the 50% probability level; hydrogen atoms are shown with an arbitrary radius (0.30 Å). C, gray; H, white; O, red; N, blue.

To elucidate the mechanism, density functional theory (DFT) calculations and experimental studies were conducted. Considering the formylchromone imine as an energetically favorable intermediate for Michael addition and the ylide as the starting set of reactants (see Scheme S1 for further details), the pyridine moiety construction mechanism has two principal phases: the addition of ylide to chromone imine and the cyclization phase where the key [1,3′-bipyridin]-1-ium intermediate **K** (detected with HPLC-MS) is formed (Figures S11 and S16). From here on, the electronically biased pyridine by the presence of the pyridinium moiety in the intermediate **K** could lead to the substitution of the pyridinium with ammonia, or the Zincke reaction to liberate the 3-NH_2_ group. The control reaction employing ^15^NH_4_OAc (Figures 1A and S12–S15) revealed that the ammonia identified in the reaction mixture did not serve as the source of the 3-amino group. Therefore, the amino group is derived from the pyridinium moiety after Zincke aminolysis with ammonia. The presence of a strong electron acceptor such as the dinitrophenyl (DNP) group is usually an electronic requirement for a Zincke reaction to take place.^27,36^ Nevertheless, such a transformation would originate the herein-described 3-aminopyridines. The analysis of the frontier orbitals of cation **K** and its zwitterion analogue **K′** shows a clear difference in the location and coefficient of the LUMO orbitals (Figure 1B).Conversion of cation **K** to zwitterion **K′** is slightly endergonic but becomes exergonic at higher theory levels. Ring opening to 3-aminopyridine is moderately uphill, whereas pyridine formation via rearrangement or intramolecular addition is strongly favored, with aromatization driving the process (Figure 1C). The analysis of HPLC-MS of the crude of the 3-aminopyridine synthesis using ^15^N-labbeled ammonium acetate (Figure S17) allowed the detection of the ^15^N-pyridine which further supports the Zincke reaction hypothesis (Figure 1D). Two control reactions were performed to support the reaction mechanism. The reaction was carried out under an inert atmosphere (Scheme 4A) to determine whether the presence of air could be key in the aromatization of the pyridine. The synthesis of 3-aminopyridine **3a** was successfully accomplished under these conditions, demonstrating a marginal increase in yield (90% in air and 93% in an inert atmosphere), indicating that air is not essential for the aromatization step. Next, 3-formylchromone **1a** was switched to 3-formylchromene **6** (Scheme 4B) to verify the effect of the ketone moiety in the reaction. Pyrimidine **7** (see Scheme S5 for mechanistic details) was obtained instead of the expected 3-aminopyridine, implying that the carbonyl was a key element, according to the DFT studies, in the stabilization of intermediate **B** after the conjugate addition of the ylide to the chromone imine.

**Figure 1:**
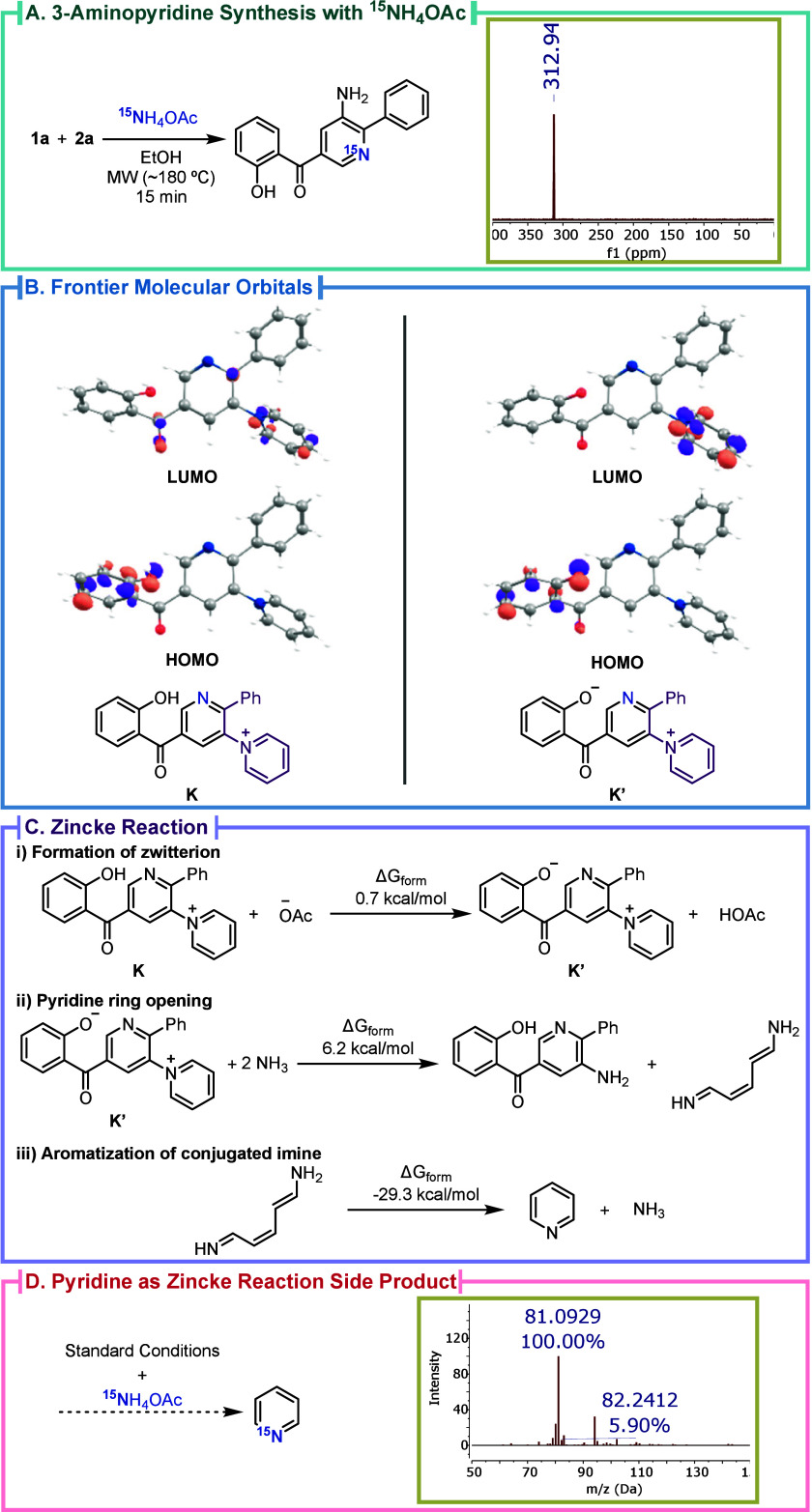
(A) Reaction of 3-formylchromone **1a** (0.459 mmol), pyridinium salt **2a** (1.5 equiv), and ^15^NH_4_OAc (15 equiv) and ^15^N NMR. (B) FMOs of intermediate **K** and zwitterionic **K**′, with solvent effects. (C) Zincke reaction intermediates and Δ*G*_form_: (i) zwitterion **K′** formation; (ii) pyridinium opening via Zincke reaction; (iii) aromatization of conjugated imine. (D) ^15^N-pyridine detected by HPLC-MS when using ^15^NH_4_OAc.

**Scheme 4:**
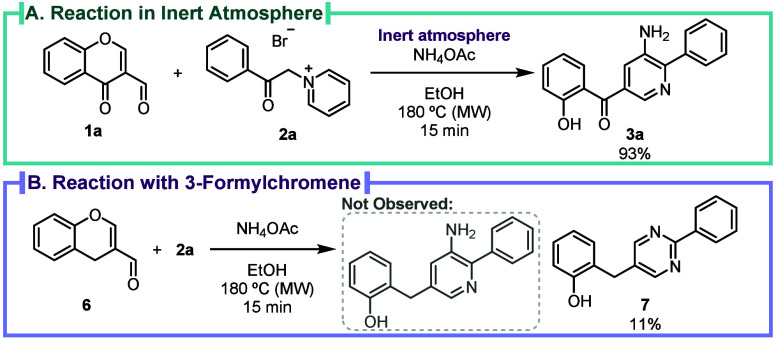
(A) 3-Aminopyridine **3a** Synthesis in Inert Conditions; (B) Reaction of 3-Formylchromene **6** under Standard Conditions

One-pot, scalable, and metal-free protocols for the synthesis of amino group-containing pyridines are highly desirable, as drug molecules typically contain amines and N-heterocycles.^37^ The scalability of the reaction was first studied by reproducing the reaction under MW irradiation at gram-scale with a slight yield decrease (Scheme 5). Therefore, to convey a more appropriate option for the large-scale production of 3-aminopyridines, we performed preliminary studies to adapt this methodology to continuous flow conditions. After optimization (monitored by HPLC), by using a mixture of dioxane and water (2:1) and a packed bed reactor using sand as the solid material (see SI for details), 3-aminopyridine **3a** was produced in 81% yield. We also verified that, at the stationary state, 3-aminopyridine **3a** has been produced in 95–100% yield. Following a duration of slightly above 5 h, 327.3 mg of 3-aminopyridine **3a** was obtained in 93% yield after isolation (Scheme 5).

**Scheme 5:**
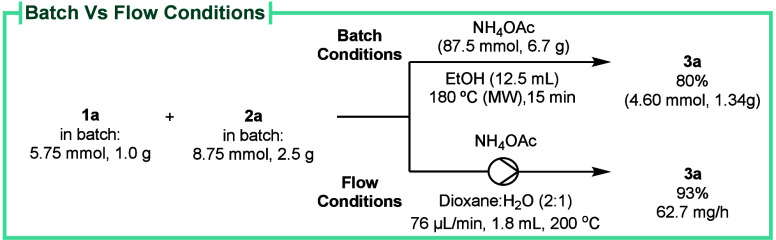
3-Aminopyridine **3a** Synthesis in Batch and Flow

The NH_2_ group in **3a** serves as a versatile synthetic handle that can be efficiently transformed into various functional groups (Schemes 6): for instance, the conversion into a pyrrole for the synthesis of pyridine **8** in 48% yield (Scheme 6A), and the conversion into amides **9** and **10** in 44% and 84% yield, respectively (Scheme 6B). Subsequently, the amino group of **3a** was used to install a wide array of substituents in the *meta*-position, such as halogens (F, Cl, Br, and I) leading to pyridines **11**, **12**, **13**, and **14** in 78–96% yield, through the respective diazonium salts (Scheme 6C). It was also possible to install an azide group to prepare pyridine **15** in 97% yield (Scheme 6D), as well as the cyclization of **3a** into 4-azacarbazole **16** in 46% yield (Scheme 6E). The 3-aminopyridine **3a** can be converted into 3-hydroxypyridine **17** in 41% yield (Scheme 6F), while treatment with potassium ethyl xanthate led to the pyridinyl carbonodithioate **18** in 43% yield, which was then converted into pyridine-3-sulfinic acid **19** in 71% yield upon treatment with NaOH (Scheme 6G).

**Scheme 6:**
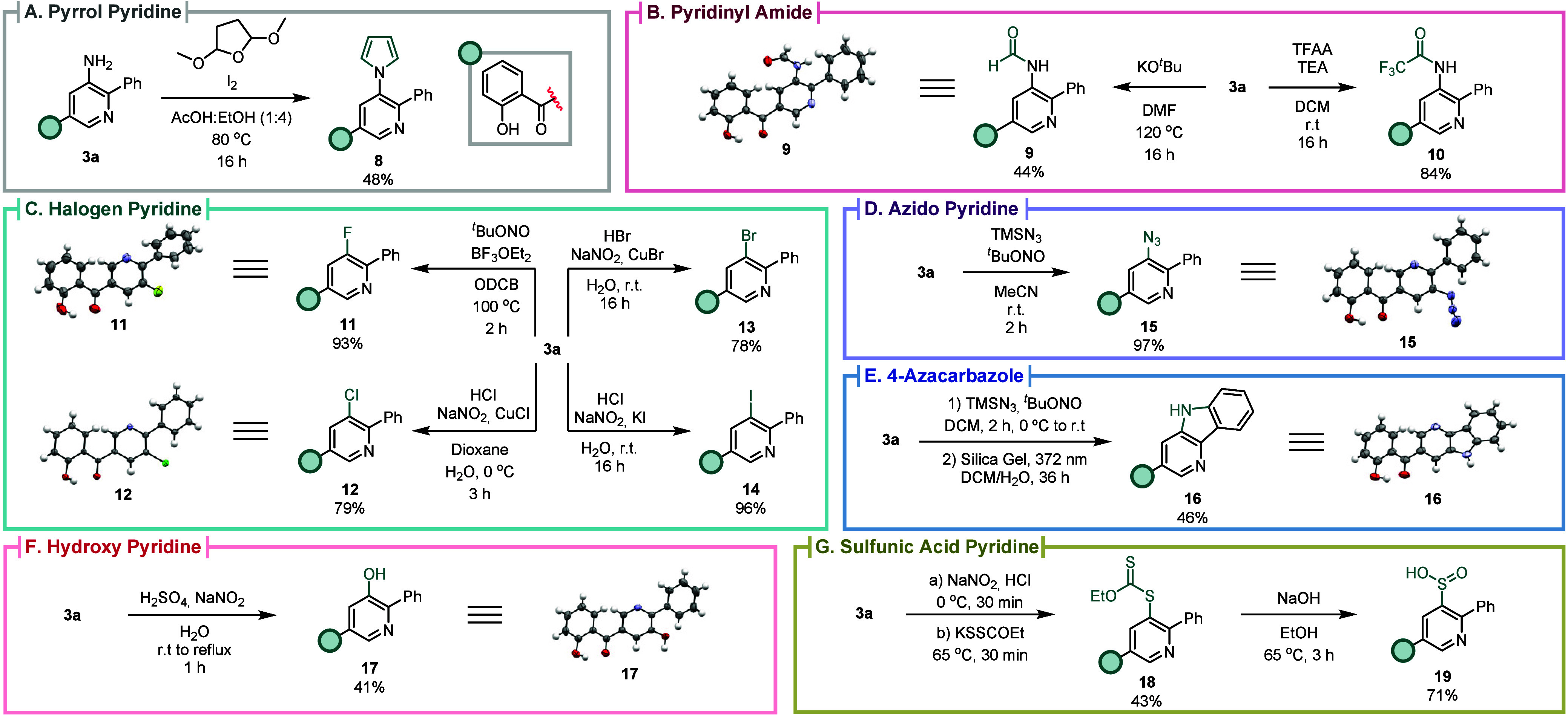
(A) Synthesis of 3-Pyrrolpyridine **8**; (B) Pyridinyl Amides **9** and **10**; (C) 3-Halopyridines **11**, **12**, **13** and **14**; (D) 3-Azidopyridine **15**; (E) 4-Azacarbazole **16**; (F) 3-Hydroxypyridine **17**; (G) Pyrinidyl Carbonodithioate **18**; and Pyridine-3-sulfinic Acid **19**; with Structures of **9**, **11**, **12**, **15**, **16**, and **17** Confirmed by X-ray Diffraction^a^

a Thermal ellipsoids are shown at the 50% probability level; hydrogen atoms are shown with an arbitrary radius (0.30 Å). C, grey; H, white; O, red; N, blue; F, yellow; Cl, green.

In summary, we have developed a new methodology for the synthesis of challenging 3-aminopyridines through a multicomponent cascade reaction characterized by a broad substrate scope, easy operation, and scalability in both batch and flow. The 3-aminopyridines revealed compelling synthetic utility and were subsequently converted into multiple *meta*-functionalized pyridines of high interest. Subsequent investigations to deploy this MCR platform in a diversity-oriented synthesis (DOS) context are underway in our laboratory.

## Supplementary Material



## Data Availability

The data underlying this study is available in the published article and its online Supporting Information.

## Notes

Experimental details, NMR spectroscopic data, X-ray crystal information, mechanistic studies, and flow optimization (PDF)
