# Supplementary material for: Synthesis and meta-Amination of Pyridines via Multicomponent Cascade Reaction
Source: Org Lett. 2025 Oct 13;27(42):11757–63. doi: 10.1021/acs.orglett.5c03509 (PMC12818832; doi:10.1021/acs.orglett.5c03509)
Supplement: Supplementary file 1 [file ol5c03509_si_001.pdf]

Supporting Information

Synthesis and *meta*-Amination of Pyridines *via* Multicomponent Cascade Reaction

Telmo N. Francisco, Nuno R. Candeias, Samuel Guieu, Joana L. C. Sousa, Rafael F. A. Gomes, Carlos A. M. Afonso, Artur M. S. Silva and Hélio M. T. Albuquerque*

Supporting Information

Synthesis and *meta*-Amination of Pyridines *via* Multicomponent Cascade Reaction

Telmo N. Francisco,[a] Nuno R. Candeias,[a],[b] Samuel Guieu, [a] Joana L. C. Sousa,[a] Rafael F. A. Gomes, [c] Carlos A. M. Afonso,[c] Artur M. S. Silva[a] and Hélio M. T. Albuquerque*[a]

[a] T. N. Francisco, Prof. N. R. Candeias, Dr. S. Guieu, Dr. J. L. C. Sousa, Prof. A. M. S. Silva, Dr. H. M. T. Albuquerque
LAQV-REQUIMTE, Department of Chemistry
University of Aveiro
3810-193 Aveiro, Portugal
E-mail: helio.albuquerque@ua.pt

[b] Prof. N. R. Candeias
Faculty of Engineering and Natural Sciences
Tampere University
Korkeakoulunkatu 8, 33101 Tampere, Finland

[c] Prof. R. F. A. Gomes, Prof. C. A. M. Afonso
Research Institute for Medicines (iMed.ULisboa)
Faculty of Pharmacy, Universidade de Lisboa
Av. Prof. Gama Pinto, 1649-003 Lisbon, Portugal

Contents

1. GENERAL INFORMATION 5
2. PROCEDURES FOR 3-AMINOPYRIDINE SYNTHESIS 6
3. OPTIMIZATION STUDIES 7
   3.1. Characterization Data 10
4. MECHANISTIC STUDIES 15
   4.1. Computational Studies 15
      4.1.1. *Detailed analysis of the mechanism* *15*
      4.1.2. *Absolute Calculation Energies and Free energies* *19*
      4.1.3. *Atomic coordinates for all the optimized species PBE1PBE/6-31+G(d,p)* *20*
   4.2. 3-Aminopyridine Synthesis using $^{15}NH_4OAc$ 31
      4.2.1. *Characterization* *31*
   4.3. HPLC-MS of reaction crude 34
   4.4. Control Reactions 37
      4.4.1. *3-Aminopyridine Synthesis under Inert Atmosphere* *37*
      4.4.2. *Reaction with 3-Formylchromene: Pyrimidine Synthesis and Characterization* *37*
5. SCALABILITY STUDIES 41
   5.1. Batch Procedure 41
   5.2. Continuous Flow Procedure 42
6. X-RAY DIFFRACTION STUDY 51
   6.1. Crystal Data and Structure Refinement 52
7. SYNTHESIS OF 3-AMINOPYRIDINES 84
   7.1. General Procedure 84
   7.2. Scope and Characterization 85
   7.3. Unexpected products 135
8. POST-FUNCTIONALIZATION OF 3-AMINOPYRIDINE 143
   8.1. Pyrrol Pyridine Synthesis 143
      8.1.1. *Procedure* *143*
      8.1.2. *Characterization* *143*
   8.2. Pyridinyl Amide Synthesis 145
      8.2.1. *Pyridinyl Formamide – Procedure* *145*
      8.2.2. *Pyridinyl Formamide – Characterization* *146*
      8.2.3. *Pyridinyl Trifluoroacetamide – Procedure* *147*
      8.2.4. *Pyridinyl Trifluoroacetamide – Characterization* *148*
   8.3. Halogen Pyridine 151
      8.3.1. *3-Fluoropyridine – Procedure* *151*
      8.3.2. *3-Fluoropyridine – Characterization* *151*
      8.3.3. *3-Chloropyridine – Procedure* *154*
      8.3.4. *3-Chloropyridine – Characterization* *154*
      8.3.5. *3-Bromopyridine – Procedure* *157*
      8.3.6. *3-Bromopyridine – Characterization* *157*
      8.3.7. *3-Iodopyridine – Procedure* *159*
      8.3.8. *3-Iodopyridine – Characterization* *160*
   8.4. Azido Pyridine Synthesis 162
      8.4.1. *Procedure* *162*
      8.4.2. *Characterization* *163*
   8.5. 4-Azacarbazole 165
      8.5.1. *Procedure* *165*

8.5.2. Characterization *165*
8.6. HYDROXYPYRIDINE SYNTHESIS 167
8.6.1. Procedure *167*
8.6.2. Characterization *168*
8.7. PYRIDINE-3-SULFINIC ACID SYNTHESIS 170
8.7.1. Pyridinyl Carbonodithioate – Procedure *170*
8.7.2. Pyridinyl Carbonodithioate – Characterization *170*
8.7.3. Pyridine-3-Sulfinic Acid – Procedure *172*
8.7.4. Pyridine-3-Sulfinic Acid– Characterization *173*

9. SYNTHESIS OF 3-FORMYLCHROMONES 175

9.1. GENERAL PROCEDURE 175
9.2. CHARACTERIZATION 175

10. SYNTHESIS OF PYRIDINIUM SALTS 178

10.1. GENERAL PROCEDURES 178
10.2. CHARACTERIZATION 178

11. REFERENCES 203

1. General Information

Unless otherwise stated, all glassware was oven-dried before use and all reactions were performed under normal atmosphere. Solvents of commercial grade were used as received unless otherwise stated. All reagents were used as received from commercial suppliers unless otherwise stated. MW-assisted reactions were carried out in a CEM Discover SP apparatus.

Reaction progress was monitored by thin layer chromatography (TLC) performed on aluminum plates coated with silica gel F254 with 0.2 mm thickness. TLC chromatograms were visualized by fluorescence quenching with UV irradiation at 254 nm (or 366 nm). Flash column chromatography was performed using silica gel 60 (230–400 mesh, Merck and co.) or a Teledyne ISCO Combiflash® RF 100 with a Teledyne ISCO RediSep® Silica Gel Flash Columns (40 grams). Preparative TLC was performed with Macherey-Nagel silica gel G/UV254.

NMR spectra were recorded with Bruker Avance 300 spectrometer (300 MHz for ^{1}H, 75 MHz for ^{13}C and 282 MHZ for ^{19}F), or Bruker Avance III TM HD - 500 spectrometer with CryoProbe Prodigy BBO (500 MHz for ^{1}H, 126 MHz for ^{13}C and 51 MHZ for ^{15}N). Chemical shifts (δ) are reported in ppm and coupling constants (*J*) in Hz; the internal standard was tetramethylsilane (TMS). Unequivocal ^{13}C assignments were made with the aid of 2D gHSQC and gHMBC (delays for one-bond and long-range *J* C/H couplings were optimised for 145 and 7 Hz, respectively) experiments. ^{1}H or ^{13}C NMR splitting patterns were designated as singlet (s), doublet (d), triplet (t), quartet (q), pentet (p). Splitting patterns that could not be interpreted or easily visualized were designated as multiplet (m) or broad (br).

Melting points were measured in a Büchi B-540 apparatus fitted with a microscope and are uncorrected.

High resolution mass spectra analysis (HRMS-ESI) was performed on a microTOF (focus) mass spectrometer. Ions were generated using an ApolloII (ESI) source. Ionization was achieved by electrospray, using a voltage of 4500 V applied to the needle, and a counter voltage between 100 and 150 V applied to the capillary.

2. Procedures for 3-Aminopyridine Synthesis

Reaxys search made in February of 2025 englobed **1237** transformations from **1147** articles (**1894–2025**) (Figure S1).

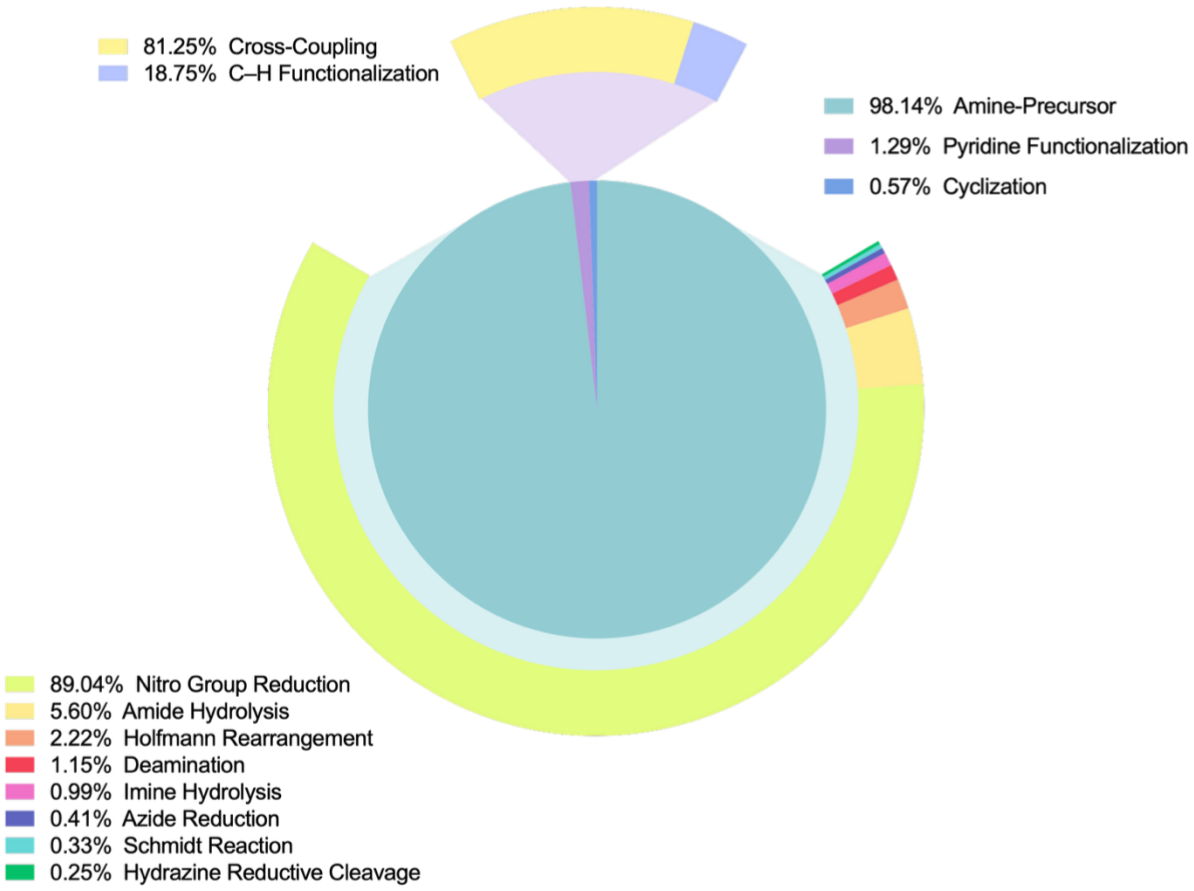

Figure S1 - Representation of the Reaxys search made in February 2025.

3. Optimization Studies

In the following three tables (Table S1, S2 and S3) is summarized the optimization study for the model reaction using 3-formylchromone **1a** and pyridinium salt **2a**. Table S1 go over experiments regarding the optimal heating method for this multicomponent reaction; Table S2 screens several ammonium salts to find optimized yields; and Table S3 deals with the optimization of different stoichiometric amounts of pyridinium salt **2a** and ammonium acetate, solvent and reaction time.

Table S1 - Screening for the optimal heating method.

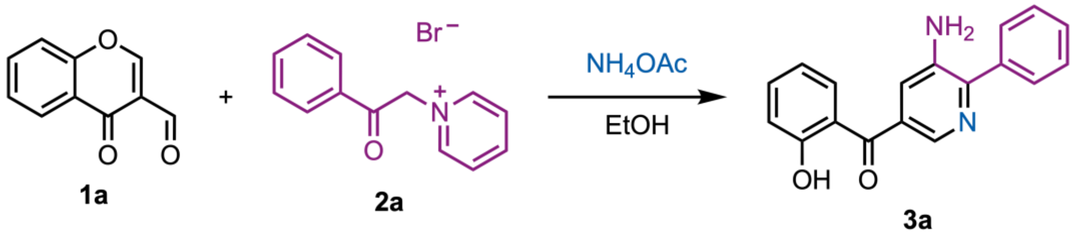

Entry	Pyridinium salt eq.	NH_4OAc eq.	Time	Control Temperature / °C	Control Pressure / psi	Heating method	Isolated yield (%)
**1**	2	30	70 h	reflux		Oil bath	54
**2**	1.5	10	10 min	70		Microwave	Vestigial
**3**	1.5	10	10 min	140		Microwave	25
**4**	1.5	10	10 min	170		Microwave	60
**5**	1.5	10	10 min		250	Microwave	73
**6**	1.5	15	20 min	200		Sealed Tube	15
**7**	1.5	15	1 h	200		Sealed Tube	68

Standard conditions: 3-formylchromone **1a** (0.46 mmol), pyridinium salt **2a**, and NH_4OAc in EtOH (1 mL) were added into the respective container.

Table S2 - Screening for the optimal ammonium salt.

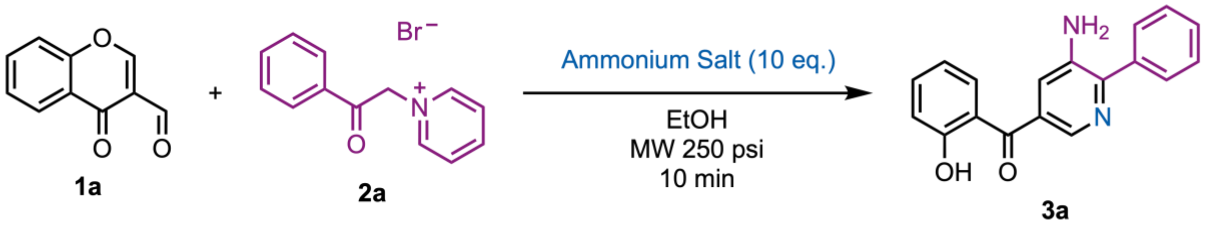

Entry	Ammonium Salt	Isolated yield (%)
1	NH_4OAc	73
2	NH_4OH	44
3	$(NH_4)_2CO_3$	52
4	NH_4HCO_2	53
5	$NH_4C_6H_5CO_2$	[a]
6	NH_4Cl	NR
7	NH_4PF_6	[b]
8	$(NH_4)_2SO_2$	[b]
9	NH_4NO_3	[b]

Standard conditions: 3-formylchromone **1a** (0.46 mmol), pyridinium salt **2a** (0.69 mmol), and ammonium salt (4.59 mmol) in EtOH (1 mL) were added into a 10 mL microwave reactor, then heated at 50 °C (20 W) for 2 min, followed by 250 psi (30 W) for 10 min. NR – No Reaction. [a] Non-isolatable due to R_f **3a** similar to R_f $NH_4C_6H_5CO_2$ [b] decomposition of the starting material.

When using NH_4OH (Table S2, Entry 3), the following indolizine (**S1**) (Figure S2) was also obtained in 21% yield.

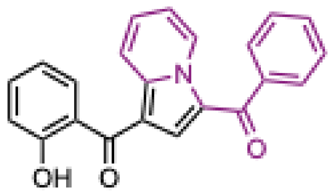

S1

Figure S2 - Structure of the indolizine **S1** obtained when NH_4OH is used as the ammonium salt.

Table S3 - Optimization of the reaction conditions.

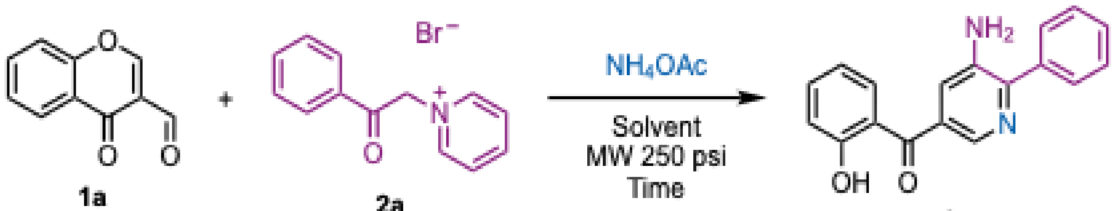

1a 2a 3a

Entry	Pyridinium salt (equiv.)	NH_4OAc (equiv.)	Solvent	Time (min)	Isolated yield (%)
1	1.5	5	EtOH	10	52
2	1.5	15	EtOH	10	82
3	1.5	20	EtOH	10	85
4	1.2	15	EtOH	10	68
5	2	15	EtOH	10	67
6	1.5	15	MeOH	10	68
7	1.5	15	Phenol	10	46
8	1.5	15	HFIP	10	57
9	1.5	15	AcOH	10	30
10	1.5	15	MeCN	10	57
11	1.5	15	DMF	10	26
12	1.5	15	THF	10	81
13	1.5	15	Toluene	10	79
14	1.5	15	No Solvent	10	28
15	1.5	15	EtOH	5	71
16	1.5	15	EtOH	15	90
17	1.5	15	EtOH	20	79
18	1.5	15	H_2O	15	65

Standard conditions: 3-formylchromone **1a** (0.46 mmol), pyridinium salt **2a**, and NH_4OAc in 1 mL of the chosen solvent were added into a 10 mL microwave reactor, heated at 50 °C (20 W) for 2 min, followed by 250 psi (30 W) for the specified time.

When using MeOH as the solvent (Table S3, entry 6), the following imine (**S2**) (Figure S3) was also obtained in 14% yield.

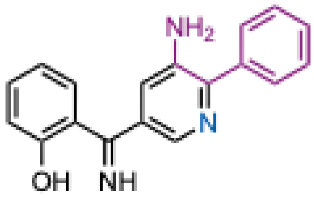

S2

Figure S3 - Structure of the imine (**S2**) obtained when MeOH is used as the solvent.

When using acetic acid as the solvent (Table S3, Entry 9), the following amide (**S3**) (Figure S4) was also obtained in 27% yield.

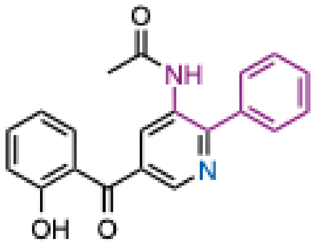

S3

Figure S4 - Structure of the amide obtained when acetic acid is used as the solvent.

3.1. Characterization Data

(3-benzoylindolizin-1-yl)(2-hydroxyphenyl)methanone (S1)

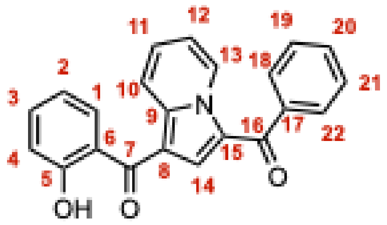

S1

Light yellow solid. Melting point of 125.8–126.6 °C.

^{1}H NMR (300 MHz, $CDCl_3$): δ 11.97 (s, O*H*, 1H), 10.03 (dt, *J* = 7.0, 1.1 Hz, H-13, 1H), 8.50 (dt, *J* = 8.9, 1.4 Hz, H-10, 1H), 7.87 – 7.80 (m, H-18 and 22, 2H), 7.77 (dd, *J* = 8.1, 1.7 Hz, H-1, 1H), 7.72 (s, H-14, 1H), 7.63 – 7.55 (m, H-11 and 20, 2H), 7.55 – 7.50 (m, H-19 and 21, 2H), 7.49 – 7.42 (m, H-3, 1H), 7.21 (td, *J* = 7.0, 1.4 Hz, H-12, 1H), 7.06 (dd, *J* = 8.4, 1.2 Hz, H-4, 1H), 6.89 (ddd, *J* = 8.1, 7.2, 1.2 Hz, H-2, 1H) ppm (Figure S5).

^{13}C NMR (75 MHz, $CDCl_3$): δ 193.4 (C-7), 185.9 (C-16), 162.5 (C-5), 141.0 (C-9), 139.8 (C-17), 135.2 (C-3), 132.0 (C-20), 131.8 (C-1), 130.2 (C-14), 129.4 (C-13), 129.2 (C-18 and 22, 2C), 129.1 (C-11), 128.7 (C-19 and 21, 2C), 122.9 (C-15), 120.9 (C-6), 120.3 (C-10), 118.9 (C-2), 118.5 (C-4), 116.6 (C-12), 113.2 (C-8) ppm (Figure S6).

HRMS (ESI) *m/z*: $[M + H]^+$ Calcd for $C_{22}H_{15}NO_3^+$ 342.1125 ; Found 342.1123.

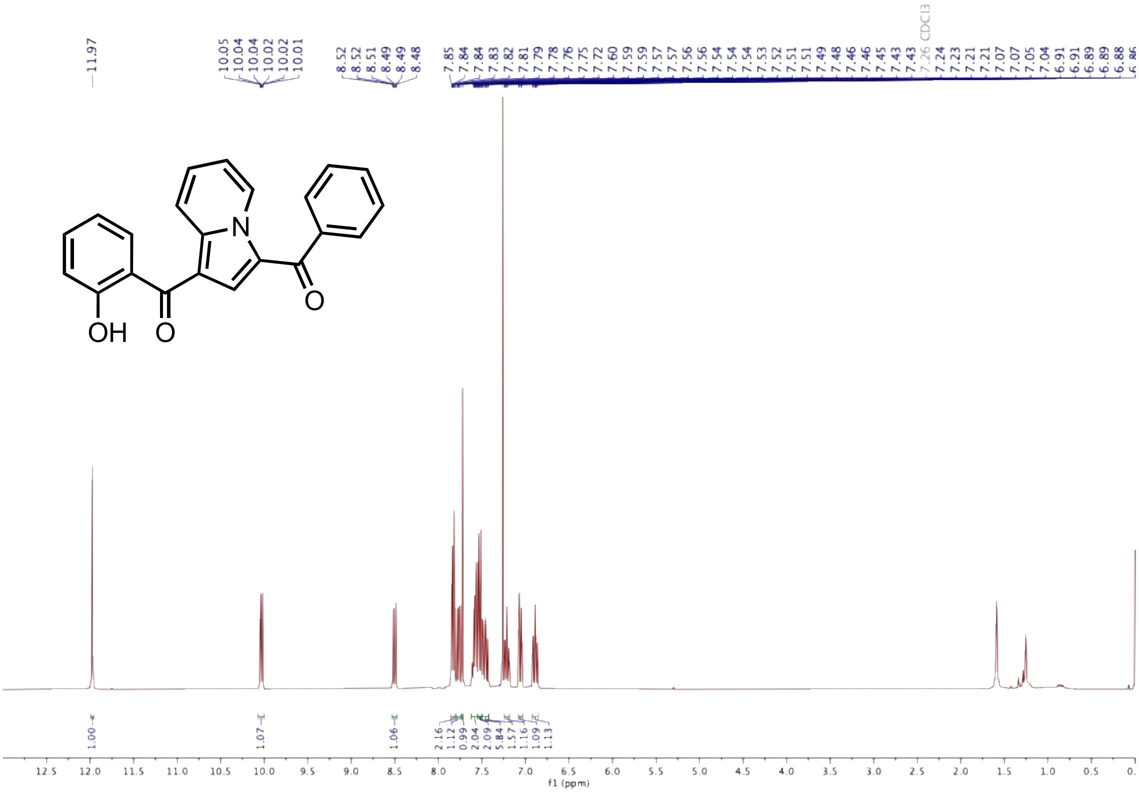

Figure S5 – ^{1}H-NMR (300 MHz, $CDCl_3$) of compound **S1**.

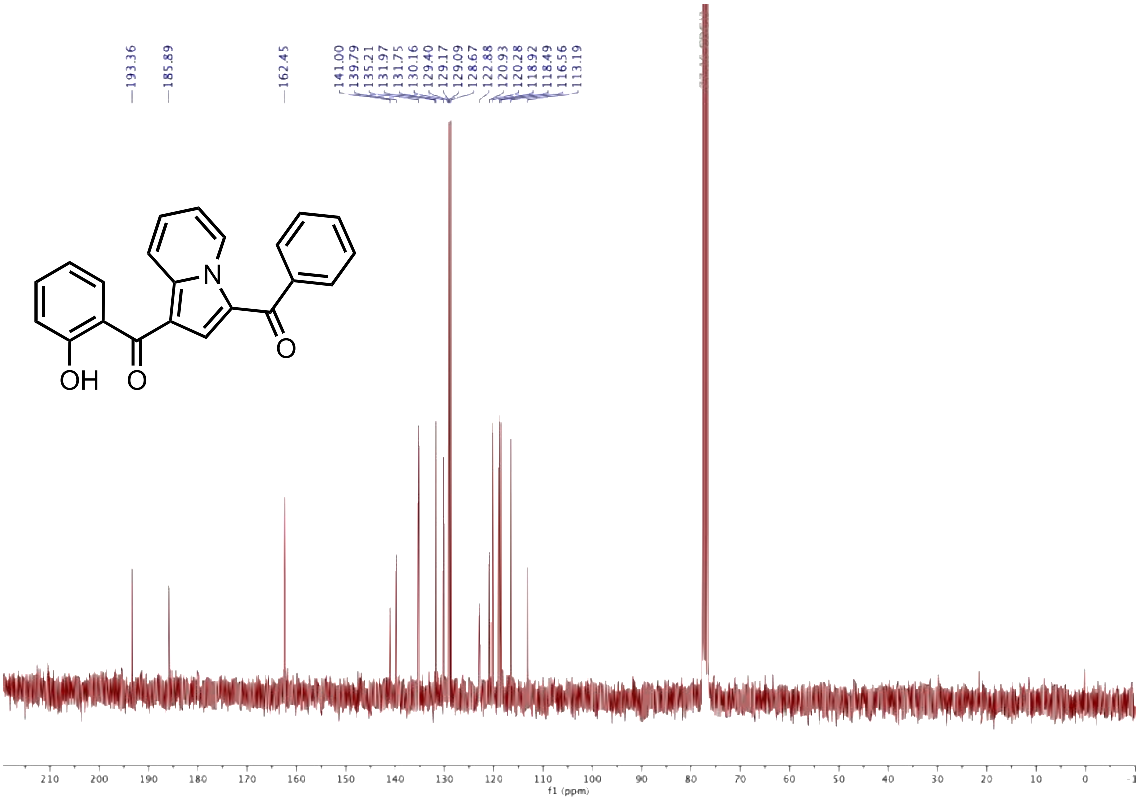

Figure S6 – ^{13}C-NMR (75 MHz, $CDCl_3$) of compound **S1**.

2-[(5-amino-6-phenylpyridin-3-yl)(imino)methyl]phenol (S2)

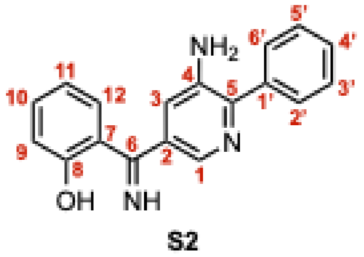

S2 Orange solid. Melting point of 135.2–137.9 °C.

^{1}H NMR (300 MHz, $CDCl_3$): δ 14.12 (s, O*H*, 1H), 9.46 (s, 6-N*H*, 1H), 8.14 (d, *J* = 1.9 Hz, H-1, 1H), 7.76 – 7.67 (m, H-2' and 6', 2H), 7.56 – 7.43 (m, H-3', 4' and 5', 3H), 7.41 – 7.35 (m, H-10, 1H), 7.31 (dd, *J* = 8.0, 1.6 Hz, H-12, 1H), 7.05 (dd, *J* = 8.4, 1.2 Hz, H-9, 1H), 7.02 (d, *J* = 1.9 Hz, H-3, 1H), 6.79 (ddd, *J* = 8.2, 7.2, 1.2 Hz, H-11, 1H), 4.08 (s, 4-N*H*$_2$, 2H) ppm (Figure S7).

^{13}C NMR (75 MHz, $CDCl_3$): δ 178.7 (C-6), 163.5 (C-8), 146.4 (C-5), 139.9 (C-4 or 1'), 137.8 (C-4 or 1'), 137.6 (C-1), 134.4 (C-2), 133.9 (C-10), 132.0 (C-12), 129.2 (C-3' and 5', 2C), 129.0 (C-4'), 128.5 (C-2' and 6', 2C), 120.8 (C-3), 118.5 (C-9), 118.3 (C-7), 118.1 (C-11) ppm (Figure S8).

HRMS (ESI) *m/z*: $[M + H]^+$ Calcd for $C_{18}H_{16}N_3O^+$ 290.1288 ; Found 290.1288.

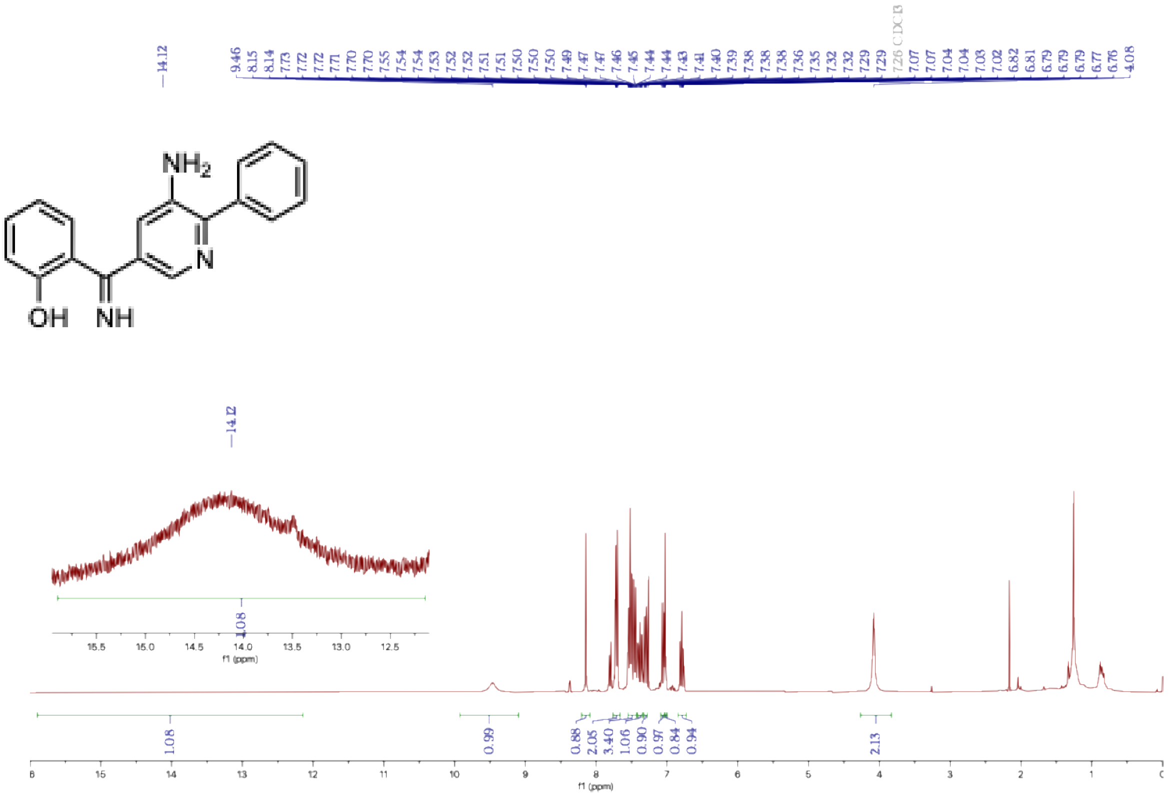

Figure S7 – ^{1}H-NMR (300 MHz, $CDCl_3$) of compound **S2**.

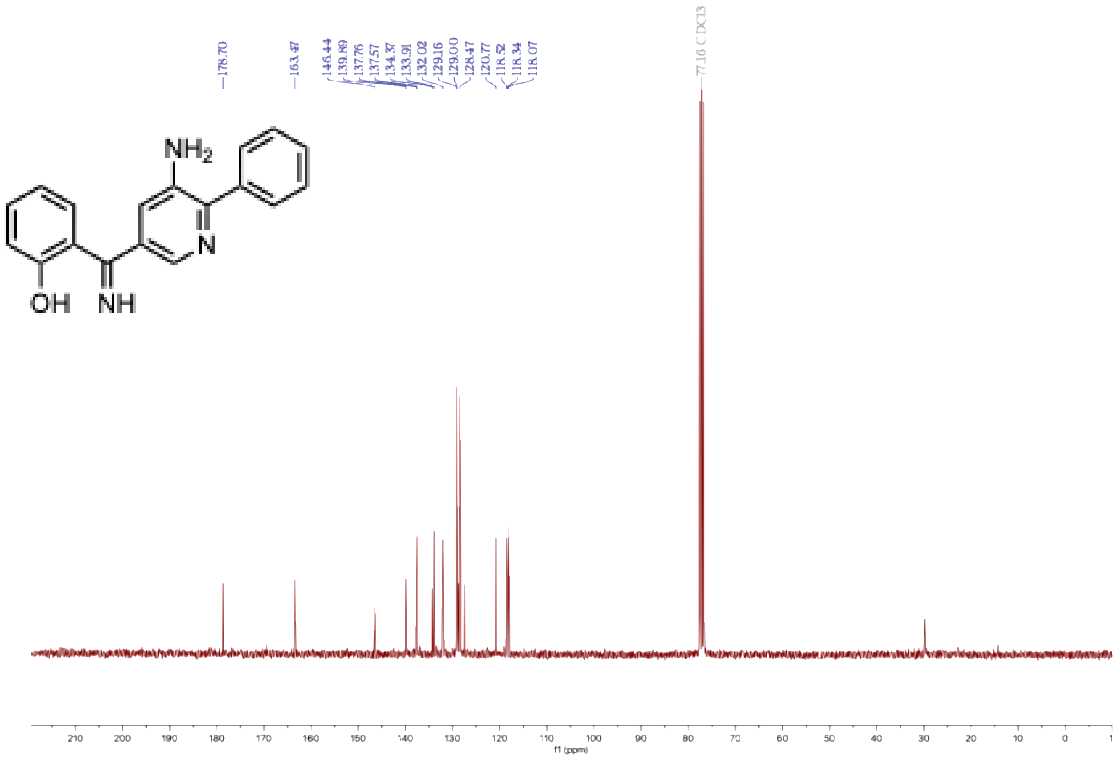

Figure S8 – ^{13}C-NMR (75 MHz, $CDCl_3$) of compound **S2**.

N-[5-(2-hydroxybenzoyl)-2-phenylpyridin-3-yl]acetamide (S3)

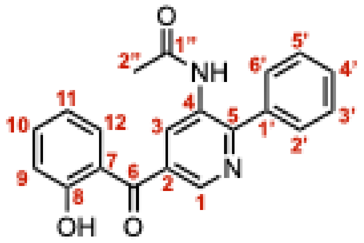

S3

Light yellow solid. Melting point of 164.8–166.1 °C.

^{1}H NMR (300 MHz, $CDCl_3$): δ 11.87 (s, O*H*, 1H), 8.83 (s, H-3, 1H), 8.62 (d, *J* = 1.2 Hz, H-1, 1H), 7.70 (s, N*H*, 1H), 7.67 (dd, *J* = 8.2, 1.7 Hz, H-12, 1H), 7.60 – 7.43 (m, H-10, 2', 3', 4', 5' and 6', 6H), 7.06 (dd, *J* = 8.5, 1.2 Hz, H-9, 1H), 6.93 (ddd, *J* = 8.2, 7.2, 1.2 Hz, H-11, 1H), 2.06 (s, H-2'', 3H) ppm (Figure S9).

^{13}C NMR (75 MHz, $CDCl_3$): δ 198.7 (C-6), 169.0 (C-1''), 163.3 (C-8), 151.9 (C-5), 144.8 (C-1), 137.1 (C-10), 136.4 (C-5), 133.2 (C-12), 132.5 (C-4), 131.4 (C-2), 130.2 (C-3), 129.8 (C-4'), 129.3 (C-3' and 5', 2C), 128.7 (C-2' and 6', 2C), 119.2 (C-11), 119.0 (C-7), 118.6 (C-9), 24.5 (C-2'') ppm (Figure S10).

HRMS (ESI) *m/z*: $[M + H]^+$ Calcd for $C_{20}H_{17}N_2O_3^+$ 333.1234 ; Found 333.1217.

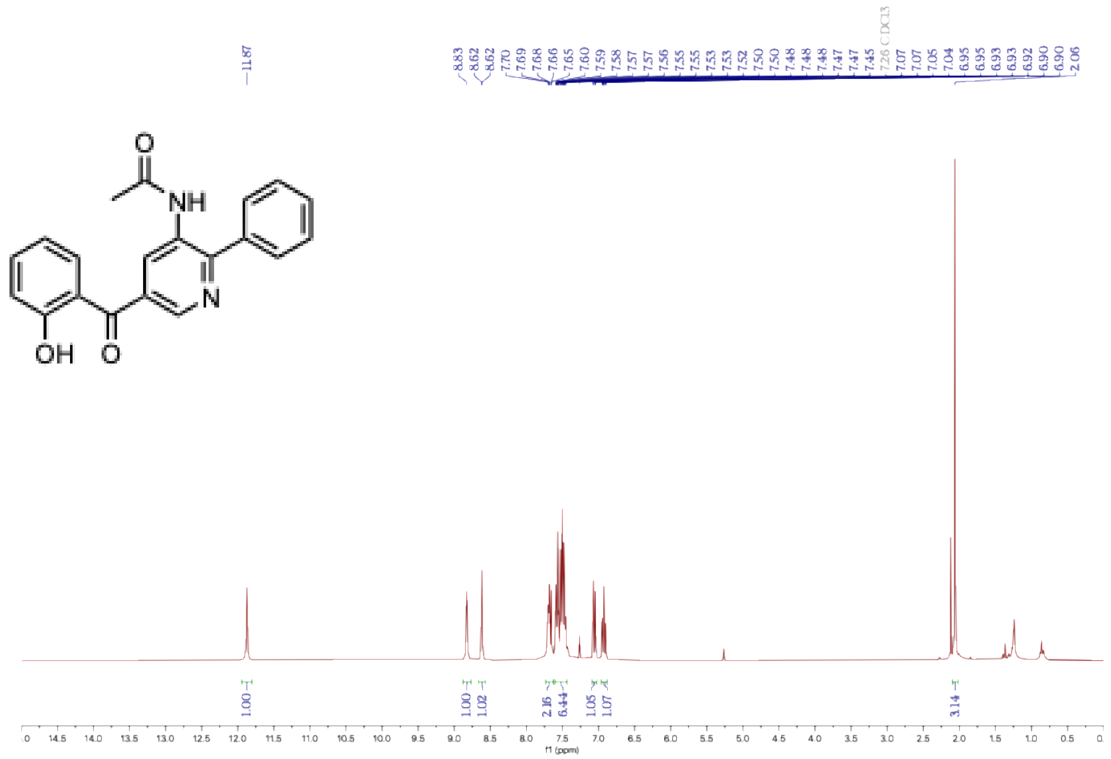

Figure S9 – ^{1}H-NMR (300 MHz, $CDCl_3$) of compound **S3**.

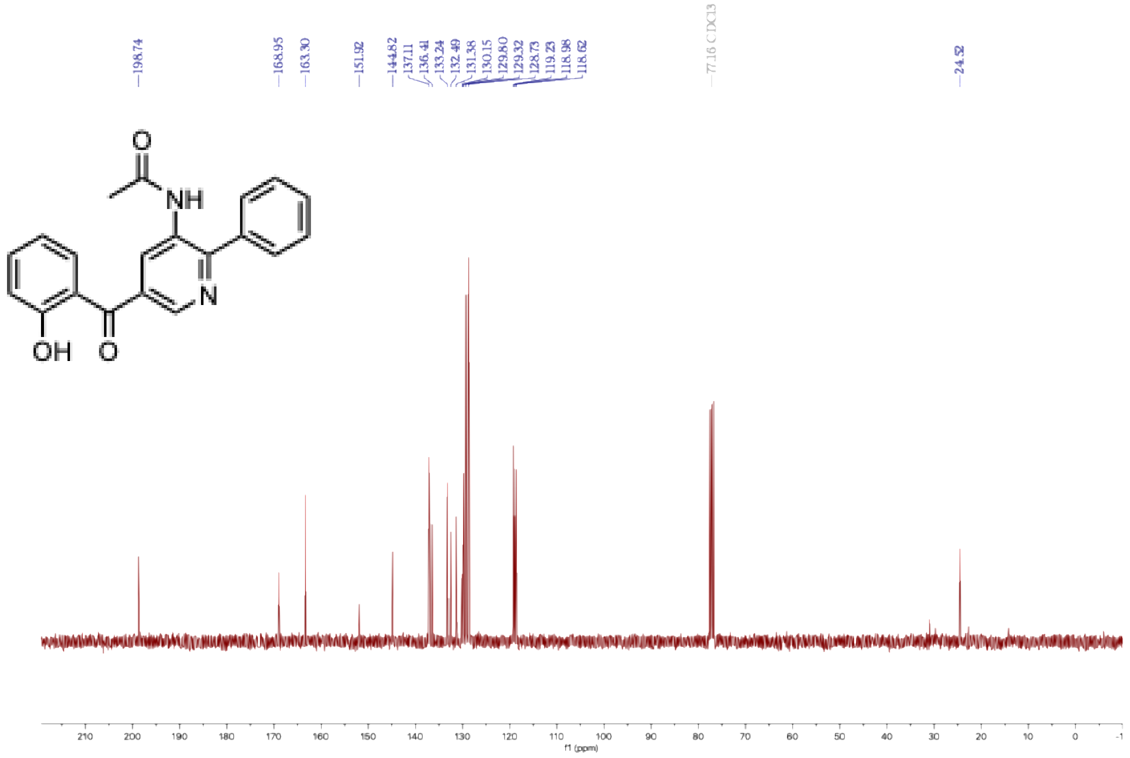

Figure S10 – ^{13}C-NMR (75 MHz, $CDCl_3$) of compound **S3**.

4. Mechanistic Studies

4.1. Computational Studies

All calculations were performed using the Gaussian 16 software package,[1] without symmetry constraints. The PBE1PBE functional was employed in the geometry optimizations. That functional uses a hybrid generalized gradient approximation (GGA), including a 25 % mixture of Hartree-Fock[2] exchange with DFT[3] exchange-correlation, given by Perdew, Burke, and Ernzerhof functional (PBE).[4] The optimized geometries were obtained using a standard 6-31+G(d,p)[5] basis set as implemented on Gaussian 16.

Transition state optimizations were performed with the Synchronous Transit-Guided Quasi-Newton Method (STQN) developed by Schlegel *et al.*[6] Frequency calculations were performed to confirm the nature of the stationary points, yielding one imaginary frequency for the transition states and none for the minima. Each transition state was further confirmed by following its vibrational mode downhill on both sides and obtaining the minima presented on the energy profile. A Natural Population Analysis (NPA)[7] and the resulting Wiberg indices (WI)[8] were used to study the electronic structure and bonding of the optimized species, and calculate as implemented on Gaussian 16.

The electronic energies (E_{b1}) obtained at the PBE1PBE/6-31+G(d,p) level of theory were converted to free energy at 298.15 K and 1 atm (G_{b1}). Single point energy calculations were performed using the PBE1PBE functional and a standard 6-311++G(d,p) basis set, taking into account the solvent effects (toluene) using the Polarizable Continuum Model (PCM) initially devised by Tomasi and coworkers[9] as implemented on Gaussian 09, with radii and non-electrostatic terms for Truhlar and coworkers' SMD solvation model.[10]

The free energy values presented along the text (G_{b2}) were derived from the electronic energy values obtained at the PBE1PBE/6-311++G(d,p)// PBE1PBE/6-31+G(d,p) level, including solvent effects (E_{b2}), according to the following expression: $G_{b2} = E_{b2} + G_{b1} - E_{b1}$.

4.1.1. Detailed analysis of the mechanism

The study began with an analysis of the deprotonation of the pyridinium salt (Scheme S1A). Although the Gibbs free energy of the deprotonation step by the acetate ion was shown slightly unfavored at the augmented basis set (5.7 kcal/mol), a slight propensity towards spontaneity was determined at the PBE1PBE/6-31+G(d,p) level (-2.1 kcal/mol). The putative subsequent step, the addition of the ylide to the chromone system was investigated to consider the addition of the

ylide directly to the 3-formylchromone or its imine congener. The conjugate addition of the ylide to the 3-formylchromone 1a requires a free energy barrier of 25.6 kcal/mol to be overcome to form the zwitterion species, itself being 11.0 kcal/mol less stable than the initial pair of reactants (Scheme S1B). On the other hand, the determined Gibbs free energy (-0.7 kcal/mol) for the imine formation (Scheme S1C), by condensation with liberated ammonia,54-56 shows that such a system would be at equilibrium, making the imine a likely intermediate of the reaction.

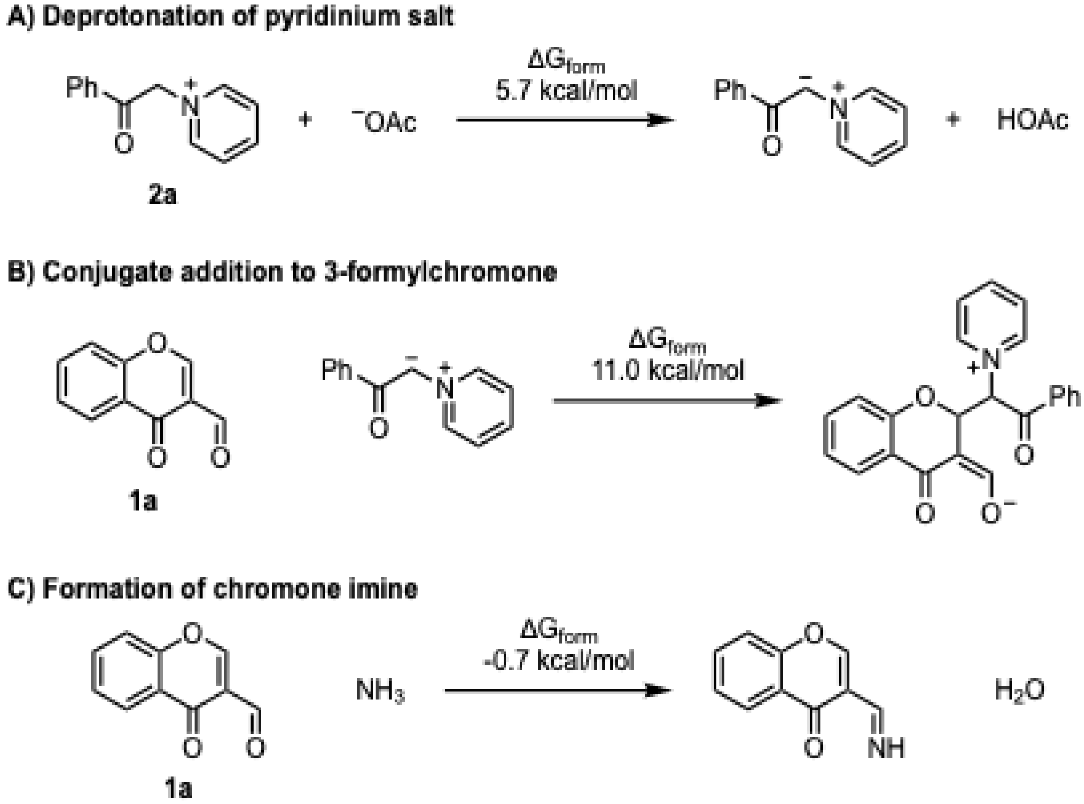

Scheme S1 – Formation of the initial intermediates for the synthesis of 3-aminopyridine 3a and DFT calculations: (A) deprotonation of the pyridinium salt leading to the ylide intermediate; (B) conjugate addition of the ylide to the 3-formylchromone 1a; and (C) formation of the chromone imine from the 3-formylchromone 1a and ammonia.

Considering the imine congener of the formylchromone as a starting point, the reaction proceeds with the conjugate addition of the ylide to deliver more stable **B** (Figure S11). The early transition state **AB‡** is characterized by an energy step of only 8.1 kcal/mol and the incipient formation of a new carbon-carbon bond (d = 2.22 Å and WI = 0.32) that develops into a stronger bond in **B** (d = 1.550 Å, WI = 0.95). The conformer **B**, being less stable than B by 2.6 kcal/mol, undergoes a proton exchange between the nitrogen and the α-ketone position to form ylide C. The **B'C‡** transition state for the proton exchange is characterized by an energy of only 2.3 kcal/mol higher than the initial set of reactants, with a clear transposition of the hydrogen atom from α-carbonyl carbon (d_{C-H} = 1.30 Å and WI_{C-H} = 0.47) to the nitrogen atom (d_{N-H} = 1.39 Å and

WI_{N-H} = 0.32). The interaction of the resultant zwitterion species **C** with ammonia results in a small destabilization of 2.1 kcal/mol to provide the ion pair **D** that undergoes opening of the *O*-heterocyclic moiety through **DE‡**. The energy barrier of 10.5 kcal/mol to overcome **DE‡** corresponds to the cleavage of the C-O bond stabilized by hydrogen bonding between the two oxygen atoms and ammonia (d_{O-HN} = 1.66-1.81 Å). The **DE‡** is characterized by a lengthening and weakening of the C-O bond (d_{C-O} = 2.00 Å and WI_{C-O} = 0.31 in **DE‡** vs d_{C-O} = 1.47 Å and WI_{C-O} = 0.80 in **D**). The ring-opened pair **E**, being less stable than **D** by 3.6 kcal/mol is stabilized to -7.7 kcal/mol upon *O*-protonation with ammonia, as represented in **EF‡**. The protonation is described by a transition state that is lower in energy than its predecessor by 1.1 kcal/mol, at the Gibbs scale and PBE1PBE/6-311++G(d,p)// PBE1PBE/6-31+G(d,p) level of theory, but a 0.6 kcal/mol higher at the electronic scale. Transition state **EF‡** is characterized by the hydrogen transposition from nitrogen (d_{N-H} = 1.22 Å and WI_{N-H} = 0.48) to oxygen atom (d_{O-H} = 1.27 Å and WI_{O-H} = 0.31). The pyridinium ammonia pair **F** equilibrates to its conformer **F'**, which suffers *N*-deprotonation by ammonia through **F'G‡**, with an energy barrier of 9.4 kcal/mol. Ammonia works a proton shuttle to form azaenolate **G** followed by nitrogen nucleophilic attack to the carbonyl with concomitant *O*-protonation to form hemiaminal **H**. The transition state for the ring closure (**GH‡**) is described by the incipient formation of a new C-N bond (d_{C-N} = 2.29 Å and WI_{C-N} = 0.18 in **GH‡** vs d_{C-N} = 1.47 Å and WI_{C-N} = 0.93 in **H**) and being 3.4 kcal/mol higher in energy than the initial set of reactants. The hemiaminal **H** is considerably more stable than the initial set of reactants by 18.9 kcal/mol, but the replacement of ammonia by the ammonium ion results in the less stable pair **I** that further engages in the dehydration process to provide **J.** Overcoming the barrier of 21.3 kcal/mol for the dehydration process is the limiting step in the annulation process, although well in line with the reaction conditions used. The dehydration transition state **IJ‡** corresponds to a simultaneous formation of a new O-H bond (d_{O-H} = 1.10 Å and WI_{O-H} = 0.47), and cleavage of a C-O bond (d_{C-O} = 1.88 Å and WI_{C-O} = 0.38) and of a N-H bond (d_{N-H} = 1.43 Å and WI_{N-H} = 0.29). The final deprotonation step is again described by a transition state that is lower in energy that its predecessor by 0.4 kcal/mol, at the Gibbs scale and PBE1PBE/6-311++G(d,p)// PBE1PBE/6-31+G(d,p) level of theory, but a 1.6 kcal/mol higher at the electronic scale. The obtained geometry for the transition state is described by a rather long interaction between the acidic proton and ammonia (d_{N-H} = 3.04 Å), a reflection of the pyridinium ion acidity.

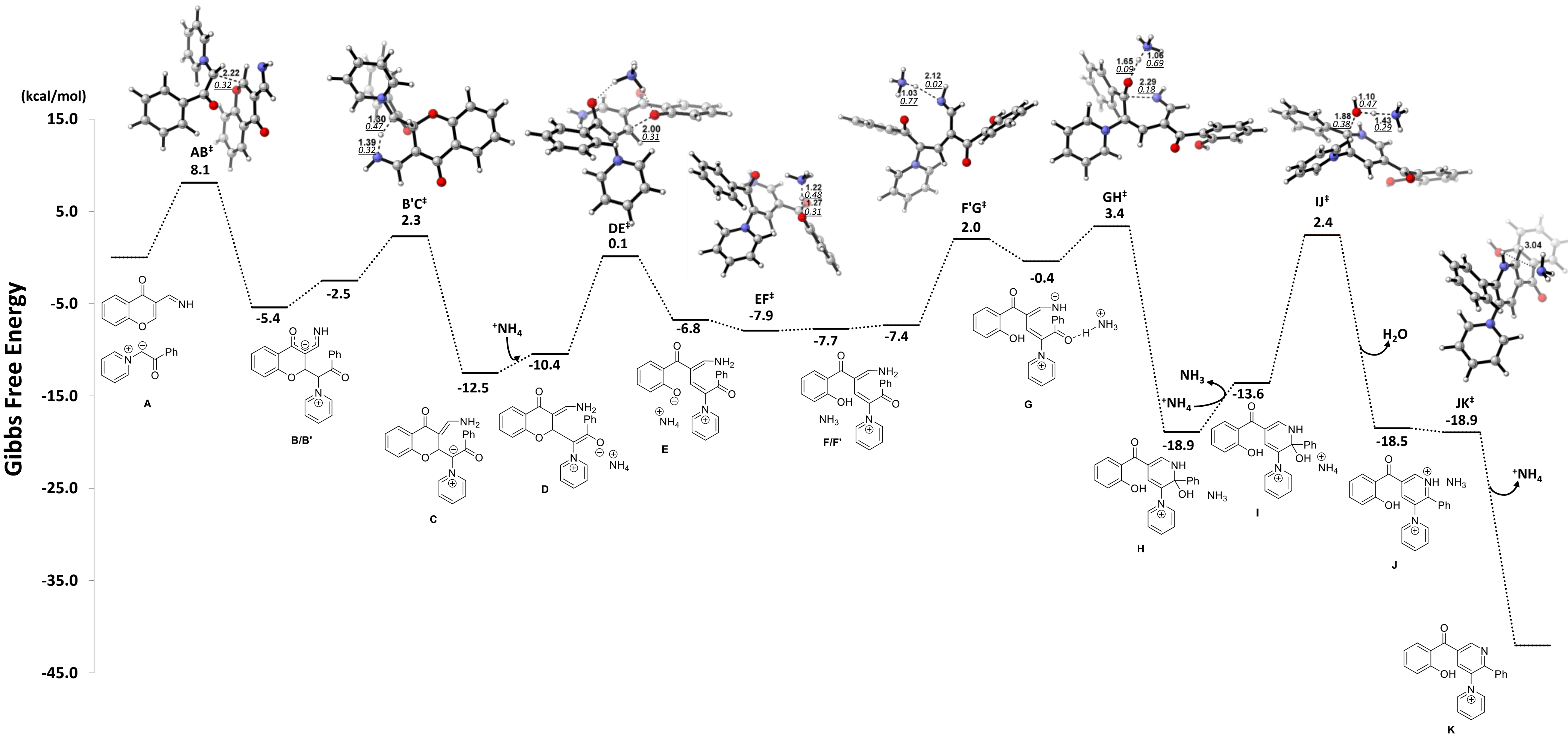

Figure S11 –Free energy profile (PBE1PBE/6-311++G(d,p)// PBE1PBE/6-31+G(d,p)) and mechanistic representation for conjugate addition – nucleophilic substitution sequence starting from pyridinium ylide and the formylchromone imine (**A**) as the initial pair of reactants. The energy values of the minima and the transition states refer to the optimized pair of reactants (**A**) and include the thermal correction to Gibbs Free energy in ethanol.

4.1.2. Absolute Calculation Energies and Free energies

Geometry	E_{b1}[a)]	G_{b1}[b)]	E_{b2}[c)]	G_{b2}[d)]	IF[e)]
A	-1221.057263	-1220.755038	-1221.330551	-1221.028327	-
AB‡	-1221.051274	-1220.743271	-1221.323468	-1221.015465	-299.6
B	-1221.075492	-1220.765214	-1221.347241	-1221.036963	-
B’	-1221.070919	-1220.759300	-1221.343965	-1221.032346	-
B’C‡	-1221.059942	-1220.753306	-1221.331289	-1221.024653	-1184.6
C	-1221.086201	-1220.775569	-1221.358899	-1221.048266	-
D	-1278.07233	-1277.713116	-1278.354050	-1277.994837	-
DE‡	-1278.055493	-1277.697857	-1278.335610	-1277.977974	-245.3
E	-1278.061925	-1277.707183	-1278.343706	-1277.988963	-
EF‡	-1278.061701	-1277.709730	-1278.342785	-1277.990813	-542.2
F	-1278.063633	-1277.709733	-1278.344416	-1277.990516	-
F’	-1278.058985	-1277.705865	-1278.343025	-1277.989905	-
F’G‡	-1278.044267	-1277.689988	-1278.329283	-1277.975004	-176.9
G	-1278.045559	-1277.693043	-1278.331370	-1277.978854	-
GH‡	-1278.040343	-1277.686894	-1278.326248	-1277.972799	-157.1
H	-1278.080962	-1277.725799	-1278.363502	-1278.008339	-
I	-1278.541445	-1278.172172	-1278.817736	-1278.448463	-
IJ‡	-1278.511754	-1278.146528	-1278.788141	-1278.422915	-415.1
J	-1202.167177	-1201.823586	-1202.417441	-1202.073851	-
JK‡	-1202.160879	-1201.820614	-1202.414818	-1202.074553	-
K	-1145.220235	-1144.923105	-1145.458623	-1145.161493	-43.4
K’	-1144.739828	-1144.456442	-1144.990216	-1144.706830	-
Formylchromone	-609.675500	-609.572647	-609.820016	-609.717164	-
Kröhnke salt	-631.732497	-631.549381	-631.861054	-631.677938	-
Ylide	-631.250098	-631.079048	-631.386352	-631.215302	-
Product	-953.2759504	-953.038851	-953.485747	-953.248647	-
Conjugated imine	-304.4680658	-304.374886	-304.544003	-304.450824	-
Pyridine	-248.005313	-247.943100	-248.058413	-247.996200	-
AcO^-	-228.361996	-228.341114	-228.450185	-228.429303	-
AcOH	-228.849637	-228.814766	-228.917791	-228.882920	-
H_2O	-76.349410	-76.346225	-76.385580	-76.382395	-
NH_3	-56.494137	-56.478588	-56.516840	-56.501290	-
NH_4^+	-56.966392	-56.936153	-56.980112	-56.949873	-

[a)]Electronic energy calculated at PBE1PBE/6-31+G(d,p) including solvent effects (ethanol); [b)]Free energy values calculated at PBE1PBE/6-31+G(d,p) including solvent effects (ethanol); [c)]Electronic energy calculated at PBE1PBE/6-311++G(d,p) including solvent effects (ethanol); [d)]Free energy values at PBE1PBE/6-311++G(d,p), determined according to $G_{b2} = E_{b2} + G_{b1} - E_{b1}$; [e)]Calculated Imaginary Frequencies for optimized geometries at PBE1PBE/6-31+G(d,p)

4.1.3. Atomic coordinates for all the optimized species PBE1PBE/6-31+G(d,p)

A

6	-4.905784	-0.475573	-2.807979
6	-4.878645	-1.821911	-2.416717
6	-5.081076	-2.161170	-1.091404
6	-5.312233	-1.166698	-0.129433
6	-5.332465	0.167161	-0.547963
6	-5.132278	0.527090	-1.879226
6	-5.541573	-1.502810	1.283919
6	-5.751550	-0.351107	2.150474
6	-5.764077	0.899082	1.619486
8	-5.557123	1.186830	0.335093
8	-5.559162	-2.670161	1.688482
6	-6.020165	-0.551354	3.578689
7	-6.274017	0.421171	4.365576
6	-9.109719	0.935325	1.497204
6	-9.230746	-0.442395	1.273665
7	-9.177598	1.962999	0.544821
6	-9.752062	-1.011472	-0.018510
8	-8.961495	-1.271375	2.187640
6	-9.162558	-2.175218	-0.523912
6	-9.657349	-2.774399	-1.678421
6	-10.767047	-2.232977	-2.326184
6	-11.377969	-1.089714	-1.813505
6	-10.871151	-0.479596	-0.669065
6	-8.728571	1.809485	-0.732454
6	-8.808126	2.836175	-1.649903
6	-9.294193	4.082804	-1.267978
6	-9.699759	4.246782	0.054527
6	-9.650118	3.183740	0.928436
1	-4.748567	-0.211192	-3.849556
1	-4.699893	-2.596128	-3.156569
1	-5.066296	-3.196463	-0.764294
1	-5.159158	1.575478	-2.158937
1	-5.931447	1.791182	2.213400
1	-5.985336	-1.597318	3.899016
1	-8.969019	1.287625	2.511680
1	-8.312777	-2.606416	-0.001200
1	-9.181900	-3.670088	-2.069616
1	-11.159729	-2.705391	-3.222641
1	-12.255347	-0.674977	-2.302833
1	-11.359285	0.405267	-0.267705
1	-8.285596	0.852799	-0.969918
1	-8.446333	2.652888	-2.656008
1	-9.341246	4.905391	-1.973412
1	-10.079213	5.195369	0.418584
1	-9.990641	3.249305	1.954376
1	-6.430688	0.063027	5.308878

AB‡

6	-5.067405	-1.145363	-2.733652
6	-4.690974	-2.313519	-2.060155
6	-4.767866	-2.366791	-0.677555
6	-5.227774	-1.266853	0.057725
6	-5.604581	-0.114976	-0.640188
6	-5.522620	-0.039904	-2.029500
6	-5.305491	-1.303562	1.533760
6	-5.875885	-0.128743	2.129414
6	-6.340791	0.916733	1.329576
8	-6.045242	1.002804	0.010133
8	-4.880821	-2.283675	2.173184
6	-6.076609	-0.060069	3.560053
7	-6.704671	0.908833	4.125656
6	-8.553309	0.749589	1.340443
6	-8.813606	-0.608852	0.970051
7	-8.949835	1.878888	0.580025
6	-9.507540	-1.001669	-0.301764
8	-8.441088	-1.517527	1.738577
6	-8.971626	-2.033780	-1.079504
6	-9.637249	-2.472591	-2.219651
6	-10.859414	-1.904851	-2.577972
6	-11.412256	-0.895240	-1.792498
6	-10.737496	-0.440087	-0.662751
6	-8.742196	1.945827	-0.761806
6	-9.083687	3.073205	-1.480123
6	-9.608993	4.183123	-0.827328
6	-9.781032	4.117147	0.552968
6	-9.454236	2.962883	1.229699
1	-5.005614	-1.098733	-3.817145
1	-4.337132	-3.173404	-2.621083
1	-4.474078	-3.257993	-0.130672
1	-5.815549	0.876767	-2.532238
1	-6.495801	1.903465	1.753785
1	-5.663082	-0.907636	4.117864
1	-8.539285	0.949832	2.407696
1	-8.027823	-2.486158	-0.787011
1	-9.205252	-3.263930	-2.826374
1	-11.382852	-2.253420	-3.464163
1	-12.372915	-0.462149	-2.057803
1	-11.178970	0.340790	-0.048332
1	-8.291414	1.079753	-1.223505
1	-8.909475	3.074024	-2.550375
1	-9.868791	5.081092	-1.377893
1	-10.180020	4.952826	1.117057
1	-9.589593	2.851678	2.298384
1	-6.734142	0.750291	5.133594

B

6	-3.280985	-0.743709	-2.029492
6	-3.007857	-1.897551	-1.292995
6	-3.706274	-2.132241	-0.114367
6	-4.678688	-1.240986	0.344189
6	-4.942030	-0.092339	-0.412001
6	-4.243787	0.160451	-1.592967
6	-5.397964	-1.486661	1.623996
6	-6.381827	-0.535876	1.960681
6	-6.726498	0.609105	1.060537

8	-5.847867	0.846908	-0.035800
8	-5.073452	-2.481752	2.326140
6	-7.053985	-0.595918	3.210368
7	-7.972059	0.251408	3.579220
6	-8.192079	0.525001	0.562085
6	-8.549271	-0.667193	-0.332774
7	-8.571176	1.787921	-0.102339
6	-9.984045	-0.960774	-0.583112
8	-7.669371	-1.346979	-0.832529
6	-10.289800	-1.892276	-1.586445
6	-11.609596	-2.215425	-1.865482
6	-12.640798	-1.615383	-1.141506
6	-12.347128	-0.692909	-0.139758
6	-11.025288	-0.362284	0.139145
6	-8.260553	1.985217	-1.401803
6	-8.533912	3.192302	-2.011494
6	-9.120306	4.214127	-1.271366
6	-9.414776	3.996991	0.071432
6	-9.128890	2.771865	0.636485
1	-2.742859	-0.543691	-2.952380
1	-2.256512	-2.602721	-1.636595
1	-3.513432	-3.016274	0.487309
1	-4.467154	1.063270	-2.154486
1	-6.712887	1.539787	1.649555
1	-6.733532	-1.422490	3.856750
1	-8.771407	0.465403	1.491475
1	-9.477116	-2.350792	-2.141448
1	-11.838407	-2.933899	-2.647227
1	-13.674884	-1.868926	-1.358198
1	-13.148909	-0.230849	0.428596
1	-10.819429	0.348410	0.933162
1	-7.795995	1.158914	-1.925114
1	-8.282595	3.319132	-3.058099
1	-9.343556	5.169554	-1.735217
1	-9.866964	4.765974	0.687002
1	-9.326018	2.537291	1.676099
1	-8.289165	0.015943	4.519372

B'

6	-2.866855	2.534191	-1.754020
6	-3.884433	2.296258	-0.827807
6	-3.886419	1.108665	-0.107392
6	-2.880670	0.154058	-0.280865
6	-1.860463	0.420359	-1.201351
6	-1.855142	1.599685	-1.946058
6	-2.925243	-1.148348	0.439844
6	-1.804102	-1.979578	0.250565
6	-0.612848	-1.528602	-0.528707
8	-0.859087	-0.470410	-1.454207
8	-3.942336	-1.433038	1.126283
6	-1.731708	-3.265067	0.851799
7	-0.680306	-4.026678	0.774517
6	0.594151	-1.198740	0.407674
6	0.400147	0.022703	1.304857
7	1.918405	-1.228355	-0.269658
6	1.054205	1.333527	1.053206
8	-0.305171	-0.139678	2.289670
6	1.319511	1.839477	-0.225551
6	1.896303	3.096972	-0.370689
6	2.230376	3.849388	0.753407
6	1.968311	3.352032	2.029735
6	1.373555	2.106372	2.179038
6	2.059162	-1.091992	-1.603702
6	3.308602	-1.160473	-2.189510
6	4.428850	-1.365497	-1.393453
6	4.264550	-1.501486	-0.017614
6	2.996930	-1.434207	0.518328
1	-2.857340	3.454968	-2.331075
1	-4.671349	3.029979	-0.678702
1	-4.676488	0.883703	0.603655
1	-1.060625	1.768267	-2.667051
1	-0.254780	-2.370622	-1.136767
1	-2.634208	-3.565290	1.398750
1	0.613575	-2.061955	1.082714
1	1.034281	1.281818	-1.110377
1	2.082253	3.490745	-1.365719
1	2.691660	4.825999	0.634999
1	2.225767	3.937805	2.907505
1	1.159695	1.711073	3.167609
1	1.159705	-0.912221	-2.177750
1	3.386733	-1.047739	-3.264538
1	5.417609	-1.420045	-1.837442
1	5.106572	-1.666985	0.644404
1	2.802384	-1.548907	1.578660
1	-0.862	-4.898534	1.272741

B'C‡

6	4.376115	1.844251	-2.082473
6	5.353365	1.067388	-1.455852
6	4.970132	0.107656	-0.528055
6	3.625287	-0.082981	-0.199007
6	2.660228	0.717395	-0.826967
6	3.031475	1.673187	-1.772894
6	3.210734	-1.162230	0.739488
6	1.844778	-1.139821	1.122187
6	0.978513	0.024222	0.713751
8	1.331659	0.561095	-0.581534
8	4.056274	-2.010559	1.114215
6	1.354950	-2.100104	2.018255
7	0.125493	-2.089585	2.473917
6	-0.514037	-0.294773	0.699639
6	-1.013119	-1.098748	-0.435678
7	-1.349910	0.839131	1.103955
6	-2.381805	-0.907574	-1.019027
8	-0.334059	-2.040604	-0.843445
6	-2.857552	0.324586	-1.480580
6	-4.104052	0.406422	-2.095756
6	-4.893078	-0.732867	-2.236767
6	-4.423198	-1.964320	-1.781969
6	-3.167914	-2.053764	-1.191811
6	-1.017948	2.110345	0.786102
6	-1.802904	3.169048	1.195014
6	-2.962052	2.920374	1.924308
6	-3.295636	1.606109	2.232931

6	-2.464666	0.583366	1.821165
1	4.662659	2.594791	-2.814375
1	6.402756	1.208642	-1.697726
1	5.705433	-0.526218	-0.040174
1	2.260553	2.270475	-2.251362
1	1.116007	0.844397	1.439699
1	2.058937	-2.875522	2.330359
1	-0.516423	-1.168985	1.660813
1	-2.247046	1.218258	-1.396955
1	-4.458	1.364256	-2.466695
1	-5.870536	-0.662733	-2.706109
1	-5.032559	-2.856724	-1.894917
1	-2.787237	-3.012702	-0.851645
1	-0.128711	2.238771	0.180870
1	-1.506682	4.176018	0.924146
1	-3.595282	3.740271	2.247786
1	-4.184543	1.362490	2.803228
1	-2.653436	-0.457947	2.051625
1	-0.131651	-2.886823	3.046422

C

6	4.731621	1.655055	-1.934554
6	5.605223	0.874954	-1.169328
6	5.085715	-0.048018	-0.274611
6	3.703005	-0.195600	-0.115219
6	2.840811	0.606735	-0.876061
6	3.356361	1.525464	-1.794502
6	3.146811	-1.247999	0.766100
6	1.716846	-1.198783	0.987381
6	0.962520	0.021796	0.500797
8	1.496140	0.489877	-0.786346
8	3.902789	-2.113085	1.246205
6	1.210549	-2.173666	1.812388
7	-0.031022	-2.322894	2.263955
6	-0.517639	-0.079298	0.346740
6	-1.130472	-1.026359	-0.499169
7	-1.260216	1.041059	0.845794
6	-2.576590	-0.886450	-0.910736
8	-0.515492	-2.021046	-0.958561
6	-3.088943	0.282178	-1.483795
6	-4.404556	0.326170	-1.940515
6	-5.226728	-0.791839	-1.817609
6	-4.721399	-1.962990	-1.253757
6	-3.401176	-2.013209	-0.818269
6	-0.836390	2.307345	0.602124
6	-1.515111	3.398785	1.103150
6	-2.684270	3.207056	1.833859
6	-3.120624	1.905856	2.061429
6	-2.380135	0.843892	1.583561
1	5.128418	2.376593	-2.643925
1	6.679459	0.987120	-1.281798
1	5.737821	-0.685075	0.316389
1	2.668270	2.127599	-2.380815
1	1.171602	0.839310	1.209476
1	1.923047	-2.916956	2.163794
1	-0.761951	-1.698142	1.948828
1	-2.454325	1.158412	-1.590727
1	-4.786705	1.236306	-2.395350
1	-6.255343	-0.753643	-2.166108
1	-5.355763	-2.840630	-1.160565
1	-2.996865	-2.931002	-0.399251
1	0.039878	2.400421	-0.027610
1	-1.135151	4.391610	0.889392
1	-3.240119	4.054151	2.222236
1	-4.014619	1.698848	2.638851
1	-2.636485	-0.187550	1.786962
1	-0.272574	-3.116952	2.835181

D

6	-2.868337	-1.260434	-2.169907
6	-2.177836	-1.543566	-0.986762
6	-2.861312	-1.537594	0.219572
6	-4.226445	-1.232120	0.269125
6	-4.897854	-0.928190	-0.923966
6	-4.223138	-0.955174	-2.146150
6	-4.986733	-1.333324	1.538169
6	-6.341485	-0.815075	1.506546
6	-6.738260	-0.013795	0.284434
8	-6.226096	-0.649915	-0.934461
8	-4.460633	-1.865093	2.531800
6	-7.044535	-0.929694	2.682273
7	-8.252335	-0.475493	2.996994
6	-8.185772	0.274399	0.051648
6	-9.172619	-0.698016	-0.065151
7	-8.476407	1.645699	-0.287203
6	-10.567144	-0.362494	-0.516686
8	-8.980910	-1.927222	0.224226
6	-10.827488	0.312406	-1.713791
6	-12.139658	0.524320	-2.130997
6	-13.203961	0.075937	-1.352149
6	-12.951545	-0.603580	-0.160886
6	-11.641709	-0.833396	0.246629
6	-7.780397	2.252137	-1.279030
6	-8.012343	3.571338	-1.607930
6	-9.002204	4.280878	-0.933493
6	-9.717126	3.643203	0.074657
6	-9.421025	2.333227	0.394057
1	-2.341860	-1.270398	-3.120528
1	-1.117079	-1.773904	-1.015464
1	-2.356589	-1.778423	1.150988
1	-4.768959	-0.727732	-3.057258
1	-6.214317	0.953276	0.341673
1	-6.531477	-1.456533	3.484411
1	-8.815188	0.017469	2.318620
1	-10.005139	0.657814	-2.334837
1	-12.329415	1.039373	-3.068910
1	-14.226974	0.248932	-1.675074
1	-13.777514	-0.960528	0.448521
1	-11.441877	-1.378707	1.165149
1	-7.057787	1.632638	-1.796688
1	-7.431242	4.022730	-2.404017
1	-9.208383	5.315760	-1.186705
1	-10.486753	4.157334	0.639001
1	-9.909049	1.802188	1.200734

1	-8.640512	-0.658021	3.908875
1	-7.538568	-4.476263	-0.544539
1	-6.273064	-3.571967	0.031275
7	-7.105320	-3.552890	-0.557455
1	-7.804146	-2.810784	-0.203209
1	-6.823001	-3.335147	-1.512751

DE‡

6	-2.697391	-0.768031	-2.095080
6	-2.055260	-0.987452	-0.869934
6	-2.824533	-1.189446	0.263920
6	-4.226216	-1.159199	0.212696
6	-4.875889	-0.929444	-1.026925
6	-4.080635	-0.743460	-2.176637
6	-4.983406	-1.459523	1.451379
6	-6.392670	-1.050358	1.546868
6	-6.909219	-0.107404	0.562959
8	-6.196663	-0.907667	-1.121325
8	-4.414671	-2.060375	2.377243
6	-7.009859	-1.393973	2.736658
7	-8.192376	-1.043376	3.209285
6	-8.253297	0.206945	0.282427
6	-9.361232	-0.699589	0.266793
7	-8.443302	1.524150	-0.298496
6	-10.695834	-0.304813	-0.297479
8	-9.273628	-1.856514	0.735454
6	-10.856156	0.243531	-1.574826
6	-12.131905	0.490863	-2.075054
6	-13.255690	0.210888	-1.301104
6	-13.102538	-0.340190	-0.029570
6	-11.831048	-0.612078	0.462512
6	-7.772522	1.862448	-1.423093
6	-7.920432	3.117499	-1.977291
6	-8.787613	4.028934	-1.381483
6	-9.473713	3.659820	-0.228633
6	-9.273490	2.402314	0.303672
1	-2.107367	-0.611254	-2.995133
1	-0.971011	-1.913	-0.810986
1	-2.355248	-1.377227	1.226032
1	-4.582043	-0.576822	-3.126569
1	-6.232862	0.727189	0.375386
1	-6.411645	-2.005414	3.408230
1	-8.824540	-0.454199	2.688995
1	-9.990548	0.452092	-2.196908
1	-12.246212	0.901844	-3.074274
1	-14.249404	0.415003	-1.690267
1	-13.975832	-0.566125	0.576108
1	-11.707155	-1.060565	1.444311
1	-7.140228	1.084889	-1.840046
1	-7.368258	3.363081	-2.877244
1	-8.924178	5.017281	-1.808674
1	-10.149693	4.341736	0.274238
1	-9.748233	2.060812	1.215341
1	-8.475195	-1.341494	4.130333
1	-7.743046	-3.787304	-1.498025
1	-6.726956	-3.936399	-0.191163
7	-7.355790	-3.305857	-0.687071
1	-8.116668	-2.993986	-0.058080
1	-6.839220	-2.429284	-0.976096

E

6	-2.621725	-0.234663	-1.905392
6	-2.108153	-0.411801	-0.612947
6	-2.956616	-0.873321	0.376983
6	-4.318012	-1.141336	0.136332
6	-4.842106	-1.001865	-1.189221
6	-3.943765	-0.525215	-2.182965
6	-5.086281	-1.627916	1.312218
6	-6.496710	-1.215759	1.530268
6	-7.026410	-0.171833	0.720731
8	-6.068325	-1.289845	-1.530234
8	-4.518759	-2.360149	2.135363
6	-7.066407	-1.757848	2.684439
7	-8.194262	-1.451519	3.280968
6	-8.301230	0.176146	0.339881
6	-9.490459	-0.659653	0.394850
7	-8.379077	1.427797	-0.395212
6	-10.812823	-0.218688	-0.150975
8	-9.426397	-1.782669	0.918642
6	-10.992365	0.339530	-1.422322
6	-12.273213	0.637054	-1.879226
6	-13.381358	0.400961	-1.069334
6	-13.209691	-0.158990	0.195763
6	-11.935423	-0.482689	0.645485
6	-7.717784	1.533957	-1.570699
6	-7.758364	2.714586	-2.283526
6	-8.494738	3.787004	-1.787017
6	-9.166977	3.653071	-0.575857
6	-9.088360	2.458326	0.110250
1	-1.975042	0.128595	-2.701406
1	-1.068799	-0.188292	-0.391658
1	-2.584780	-1.018318	1.388194
1	-4.337469	-0.416	-3.190287
1	-6.258724	0.500368	0.337920
1	-6.449537	-2.482782	3.209814
1	-8.850704	-0.786766	2.900560
1	-10.145505	0.509931	-2.079313
1	-12.403092	1.053157	-2.874237
1	-14.377982	0.646109	-1.425794
1	-14.070729	-0.351137	0.829685
1	-11.797492	-0.935723	1.623042
1	-7.179551	0.642633	-1.889013
1	-7.221668	2.780694	-3.222879
1	-8.541548	4.720685	-2.338338
1	-9.742790	4.467065	-0.151095
1	-9.568641	2.286997	1.066201
1	-8.432552	-1.891330	4.158524
1	-7.795102	-3.773284	-1.474638
1	-6.801852	-3.977254	-0.169002
7	-7.372293	-3.302012	-0.675853
1	-8.113019	-2.962768	-0.051968
1	-6.751857	-2.430647	-1.013338

EF‡

6	-2.652989	-0.231268	-1.909260
6	-2.142346	-0.417450	-0.618711
6	-2.987412	-0.898945	0.365564
6	-4.344421	-1.177465	0.117138
6	-4.858272	-1.030234	-1.205154
6	-3.971891	-0.536078	-2.193209
6	-5.116257	-1.672588	1.291339
6	-6.506696	-1.219246	1.536445
6	-7.028652	-0.165312	0.732566
8	-6.089861	-1.326778	-1.560924
8	-4.558586	-2.442931	2.084295
6	-7.067893	-1.741309	2.704117
7	-8.176726	-1.402610	3.317601
6	-8.300816	0.185691	0.352999
6	-9.495392	-0.646631	0.421862
7	-8.380617	1.435933	-0.383751
6	-10.810612	-0.214622	-0.148002
8	-9.436530	-1.754164	0.973266
6	-10.974204	0.314192	-1.433930
6	-12.248972	0.604038	-1.912170
6	-13.366753	0.389495	-1.109489
6	-13.210891	-0.141040	0.170237
6	-11.942590	-0.456971	0.641871
6	-7.721352	1.544775	-1.559969
6	-7.763489	2.726291	-2.271028
6	-8.500226	3.797310	-1.772125
6	-9.171948	3.660486	-0.561010
6	-9.091432	2.464879	0.123221
1	-2.008917	0.147437	-2.699732
1	-1.105955	-0.185876	-0.392052
1	-2.616502	-1.049742	1.376064
1	-4.365274	-0.418378	-3.220
1	-6.257011	0.499251	0.343563
1	-6.458985	-2.477216	3.223636
1	-8.825667	-0.729209	2.939234
1	-10.119608	0.466833	-2.085255
1	-12.366620	0.997040	-2.918035
1	-14.358643	0.628530	-1.482918
1	-14.079575	-0.316172	0.798628
1	-11.816561	-0.886826	1.631434
1	-7.186366	0.654406	-1.883772
1	-7.228166	2.793912	-3.211031
1	-8.548282	4.731833	-2.321884
1	-9.748800	4.472944	-0.134728
1	-9.570931	2.291740	1.079274
1	-8.410143	-1.828582	4.203274
1	-7.713803	-3.806580	-1.458080
1	-6.725410	-3.949535	-0.149197
7	-7.309325	-3.299934	-0.672557
1	-8.066006	-2.988138	-0.059037
1	-6.671142	-2.337598	-1.057231

F

6	-2.675166	-0.143717	-1.885140
6	-2.193705	-0.336620	-0.587718
6	-3.037609	-0.882399	0.365757
6	-4.370398	-1.218359	0.072183
6	-4.836897	-1.064144	-1.254003
6	-3.970668	-0.510920	-2.212251
6	-5.160834	-1.760410	1.221433
6	-6.518282	-1.251551	1.509297
6	-7.030472	-0.181971	0.716895
8	-6.063312	-1.407105	-1.671162
8	-4.626490	-2.604944	1.948531
6	-7.061803	-1.753405	2.694764
7	-8.138352	-1.373580	3.339387
6	-8.300196	0.178003	0.346894
6	-9.510767	-0.635080	0.447532
7	-8.385630	1.432756	-0.381763
6	-10.814043	-0.195051	-0.143747
8	-9.472098	-1.719461	1.038005
6	-10.958958	0.272496	-1.455113
6	-12.223555	0.575756	-1.951540
6	-13.348363	0.435920	-1.141965
6	-13.210688	-0.033149	0.163547
6	-11.953136	-0.362734	0.654635
6	-7.758864	1.549843	-1.574228
6	-7.811467	2.738153	-2.272812
6	-8.526967	3.809059	-1.743898
6	-9.167203	3.664318	-0.516797
6	-9.076368	2.461624	0.153268
1	-2.030725	0.283426	-2.649250
1	-1.175959	-0.062179	-0.327291
1	-2.685629	-1.036947	1.382113
1	-4.344800	-0.397366	-3.226146
1	-6.254527	0.471977	0.317301
1	-6.462394	-2.507381	3.199203
1	-8.782505	-0.690786	2.969172
1	-10.097796	0.366583	-2.109121
1	-12.328108	0.920929	-2.976225
1	-14.332010	0.685687	-1.529910
1	-14.085201	-0.149021	0.797545
1	-11.841459	-0.744135	1.665608
1	-7.241797	0.662197	-1.926681
1	-7.301535	2.810603	-3.226421
1	-8.583529	4.749109	-2.283277
1	-9.727331	4.475944	-0.067336
1	-9.531137	2.282355	1.120291
1	-8.358134	-1.785435	4.235246
1	-7.608266	-4.045098	-1.356054
1	-6.674181	-4.037295	-0.015584
7	-7.248086	-3.449089	-0.614726
1	-8.041193	-3.142493	-0.053735
1	-6.495924	-2.218323	-1.170348

F’

6	-1.753478	-1.905962	-1.473323
6	-1.693989	-2.275829	-0.129742
6	-2.866279	-2.318350	0.613054
6	-4.130	-1.966404	0.052542
6	-4.145993	-1.614347	-1.308177
6	-2.969865	-1.584914	-2.062691
6	-5.318647	-2.108441	0.915549
6	-6.289412	-1.016474	1.058801

6	-7.482471	-1.401695	1.729716
8	-5.353343	-1.337997	-1.856460
8	-5.460207	-3.172713	1.529497
6	-5.954162	0.224630	0.485536
7	-6.725845	1.245909	0.232929
6	-8.448017	-0.729144	2.437053
6	-8.380098	0.643659	2.942737
7	-9.550324	-1.560844	2.882627
6	-9.559848	1.305789	3.584231
8	-7.315186	1.260438	2.873756
6	-9.296595	2.204232	4.626347
6	-10.333504	2.896870	5.238824
6	-11.645132	2.718517	4.800246
6	-11.914063	1.843456	3.750037
6	-10.879939	1.131541	3.149187
6	-10.326274	-2.181942	1.965767
6	-11.356643	-3.009971	2.361272
6	-11.605280	-3.187603	3.718605
6	-10.797598	-2.538576	4.648121
6	-9.766416	-1.736950	4.205946
1	-0.847662	-1.873655	-2.072622
1	-0.744735	-2.532791	0.330256
1	-2.842890	-2.616310	1.657770
1	-3.022665	-1.316141	-3.115290
1	-7.614586	-2.484454	1.726634
1	-4.917366	0.356131	0.184467
1	-7.772621	1.273044	0.314115
1	-8.270468	2.345518	4.952889
1	-10.119606	3.579442	6.056431
1	-12.456534	3.263576	5.274628
1	-12.932630	1.713415	3.395574
1	-11.107905	0.464201	2.323609
1	-10.095968	-1.975201	0.927925
1	-11.959766	-3.495890	1.603051
1	-12.416957	-3.827740	4.049085
1	-10.946518	-2.660751	5.714744
1	-9.079689	-1.236990	4.877533
1	-6.299689	2.052120	-0.206056
7	-9.500529	1.579575	-0.329
1	-9.942229	2.262700	0.609176
1	-9.578713	1.949288	-0.944941
1	-10.089541	0.751533	0.028554
1	-5.242092	-1.104514	-2.788200

F’G‡

6	-2.145075	-2.084176	-1.967396
6	-1.819168	-2.215310	-0.617777
6	-2.837296	-2.221662	0.327285
6	-4.178680	-2.064098	-0.040211
6	-4.491371	-1.952581	-1.406275
6	-3.471867	-1.961859	-2.361947
6	-5.215246	-2.152605	1.042825
6	-6.256436	-1.131123	1.195425
6	-7.308588	-1.529840	2.023755
8	-5.796388	-1.866970	-1.758756
8	-5.129489	-3.115706	1.818812
6	-6.132455	0.077850	0.406707
7	-7.033143	0.978931	0.208213
6	-8.381435	-0.867804	2.618225
6	-8.481697	0.528401	2.951973
7	-9.418354	-1.744480	3.118197
6	-9.810671	1.146226	3.249408
8	-7.464281	1.255	2.999193
6	-9.881279	2.141208	4.232856
6	-11.084203	2.788281	4.491525
6	-12.222956	2.462609	3.755230
6	-12.155861	1.487205	2.761725
6	-10.957727	0.825249	2.512721
6	-9.986031	-2.646770	2.284255
6	-10.941414	-3.531165	2.741687
6	-11.346625	-3.472137	4.071074
6	-10.760366	-2.532676	4.914575
6	-9.785667	-1.692084	4.420323
1	-1.363305	-2.084617	-2.722230
1	-0.783627	-2.317650	-0.307808
1	-2.604296	-2.336478	1.382527
1	-3.733652	-1.881026	-3.414673
1	-7.279622	-2.599887	2.240786
1	-5.158189	0.169393	-0.084789
1	-8.194577	2.678621	0.717188
1	-8.987446	2.392407	4.797332
1	-11.134952	3.547910	5.266613
1	-13.162030	2.972039	3.953440
1	-13.038199	1.243576	2.176640
1	-10.907983	0.078212	1.725081
1	-9.660191	-2.611188	1.252190
1	-11.371070	-4.243239	2.046474
1	-12.105539	-4.151001	4.446372
1	-11.031822	-2.460347	5.961492
1	-9.256425	-0.979628	5.040200
1	-6.635514	1.660069	-0.442714
7	-8.112332	3.382835	1.460961
1	-7.812436	2.831607	2.294068
1	-7.405570	4.072841	1.205531
1	-9.003965	3.848940	1.626356
1	-5.864483	-1.784316	-2.719677

G

6	-2.226746	-1.974418	-2.033205
6	-1.866987	-2.084258	-0.690326
6	-2.864045	-2.134876	0.275605
6	-4.218878	-2.043452	-0.064205
6	-4.564912	-1.955075	-1.423893
6	-3.565765	-1.918379	-2.400304
6	-5.226049	-2.166126	1.042542
6	-6.307393	-1.191215	1.199796
6	-7.323885	-1.604960	2.061547
8	-5.879185	-1.938937	-1.750788
8	-5.077264	-3.110096	1.833056
6	-6.272304	0.018799	0.396853
7	-7.252417	0.838116	0.244521
6	-8.412187	-0.946365	2.638206
6	-8.536045	0.461334	2.897968
7	-9.431470	-1.817004	3.176306

6	-9.871497	1.091591	3.112511
8	-7.516793	1.183983	2.944918
6	-9.972501	2.157915	4.015176
6	-11.185655	2.812004	4.196075
6	-12.304569	2.429	3.461714
6	-12.207279	1.371536	2.548307
6	-10.998489	0.704484	2.376847
6	-9.959396	-2.789580	2.396030
6	-10.897846	-3.668668	2.896451
6	-11.330047	-3.532691	4.211619
6	-10.785912	-2.521793	4.998955
6	-9.824868	-1.689243	4.466175
1	-1.461791	-1.939839	-2.804303
1	-0.821542	-2.135485	-0.401661
1	-2.604166	-2.232512	1.326286
1	-3.852019	-1.854856	-3.447855
1	-7.253840	-2.663644	2.320135
1	-5.317100	0.181429	-0.115598
1	-8.230979	3.582337	1.020500
1	-9.094855	2.456824	4.581904
1	-11.260155	3.628443	4.909
1	-13.251960	2.933991	3.599287
1	-13.074216	1.075532	1.964353
1	-10.923732	-0.099088	1.649078
1	-9.617834	-2.813611	1.368867
1	-11.294622	-4.437679	2.243586
1	-12.076622	-4.206620	4.619128
1	-11.078336	-2.387352	6.034061
1	-9.324908	-0.924696	5.046862
1	-6.956393	1.571358	-0.403161
7	-7.597635	3.619766	1.819364
1	-7.600297	2.680201	2.313111
1	-6.656884	3.828435	1.483725
1	-7.900196	4.361986	2.450466
1	-5.970532	-1.865561	-2.710604

GH‡

6	-2.537926	-1.520372	-2.302895
6	-2.091588	-1.637014	-0.987430
6	-3.018126	-1.856215	0.025534
6	-4.389327	-1.931431	-0.239328
6	-4.820790	-1.831275	-1.572485
6	-3.892634	-1.623818	-2.596379
6	-5.314388	-2.240319	0.907512
6	-6.402839	-1.354853	1.235786
6	-7.365054	-1.835493	2.156895
8	-6.146126	-1.965814	-1.829838
8	-5.064298	-3.262451	1.573720
6	-6.598331	-0.109520	0.556224
7	-7.561709	0.695454	0.856246
6	-8.381084	-1.141093	2.751483
6	-8.454653	0.342054	2.933989
7	-9.401021	-1.891333	3.426480
6	-9.768416	1.019815	2.708001
8	-7.548691	0.906838	3.574739
6	-10.099268	2.130747	3.489686
6	-11.323682	2.770086	3.318324
6	-12.226771	2.300488	2.367181
6	-11.904405	1.188331	1.588215
6	-10.682911	0.548355	1.759417
6	-9.938489	-2.985103	2.831976
6	-10.889937	-3.747170	3.477763
6	-11.341327	-3.362703	4.736040
6	-10.799947	-2.222163	5.323087
6	-9.821237	-1.513869	4.660416
1	-1.829273	-1.355644	-3.110085
1	-1.033301	-1.564281	-0.755234
1	-2.685885	-1.964324	1.054865
1	-4.244110	-1.552791	-3.623352
1	-7.307104	-2.904096	2.368301
1	-5.901664	0.126217	-0.253830
1	-6.479368	3.313625	1.872518
1	-9.397220	2.478389	4.242464
1	-11.575697	3.630583	3.931871
1	-13.183225	2.798399	2.232897
1	-12.606456	0.823097	0.843894
1	-10.422502	-0.306039	1.140398
1	-9.595766	-3.196502	1.827461
1	-11.289042	-4.619284	2.972427
1	-12.103776	-3.939168	5.249453
1	-11.110049	-1.886814	6.306195
1	-9.323722	-0.658204	5.099399
1	-7.613860	1.499222	0.232116
7	-6.770679	3.392790	2.847492
1	-7.140816	2.459793	3.179629
1	-5.969070	3.674787	3.412759
1	-7.497059	4.105483	2.921356
1	-6.295380	-1.894400	-2.782367

H

6	-2.639591	-1.542732	-2.516829
6	-2.190686	-2.077582	-1.309780
6	-3.114099	-2.366012	-0.313756
6	-4.478851	-2.102766	-0.480778
6	-4.918946	-1.581268	-1.710210
6	-3.992767	-1.303831	-2.720136
6	-5.396404	-2.503389	0.635484
6	-6.369954	-1.571311	1.172806
6	-7.344336	-2.028815	2.120316
8	-6.248207	-1.389621	-1.893764
8	-5.268984	-3.634194	1.120637
6	-6.419488	-0.235075	0.833050
7	-7.304213	0.596479	1.374084
6	-8.200457	-1.177784	2.714253
6	-8.154999	0.329915	2.543062
7	-9.218854	-1.700608	3.585755
6	-9.553684	0.888668	2.256151
8	-7.582746	0.878318	3.688334
6	-10.271735	1.562660	3.245204
6	-11.558996	2.029172	2.984246
6	-12.140171	1.822201	1.736105
6	-11.430196	1.142128	0.747956
6	-10.145604	0.673837	1.007796
6	-10.090859	-2.615448	3.102046

6	-11.058991	-3.161497	3.918601
6	-11.150972	-2.742887	5.242372
6	-10.256993	-1.787304	5.716351
6	-9.290055	-1.283257	4.871963
1	-1.934483	-1.316346	-3.311984
1	-1.134508	-2.272380	-1.150740
1	-2.786605	-2.796455	0.628720
1	-4.348264	-0.907316	-3.668498
1	-7.391351	-3.092941	2.337968
1	-5.757023	0.205234	0.095424
1	-6.958396	3.896524	2.835421
1	-9.821279	1.726930	4.218714
1	-12.106666	2.556526	3.760592
1	-13.143103	2.187800	1.533340
1	-11.877461	0.974578	-0.228040
1	-9.598271	0.140311	0.234932
1	-9.982650	-2.870257	2.055380
1	-11.739832	-3.896423	3.504905
1	-11.911567	-3.156018	5.897113
1	-10.289771	-1.437643	6.741697
1	-8.545817	-0.556024	5.170700
1	-7.296261	1.561839	1.067194
7	-7.466606	3.537726	3.639906
1	-7.579271	1.901706	3.640171
1	-6.977453	3.878949	4.463797
1	-8.375662	3.992886	3.633458
1	-6.404860	-1.047206	-2.784502

I

6	-2.718675	-0.868289	-2.396648
6	-2.188717	-1.182121	-1.145046
6	-3.050620	-1.535784	-0.116429
6	-4.439324	-1.554701	-0.298102
6	-4.957618	-1.258311	-1.571834
6	-4.089817	-0.913571	-2.612245
6	-5.275877	-1.983716	0.865246
6	-6.441492	-1.211183	1.277882
6	-7.358552	-1.765467	2.229568
8	-6.294790	-1.348516	-1.765040
8	-4.928485	-2.972744	1.517936
6	-6.715328	0.057380	0.819297
7	-7.732919	0.772555	1.304557
6	-8.347059	-1.019854	2.759801
6	-8.459919	0.468237	2.522867
7	-9.331613	-1.635679	3.608518
6	-9.904390	0.937153	2.375919
8	-7.822047	1.098396	3.637253
6	-10.558065	1.615543	3.405680
6	-11.886239	2.008303	3.250145
6	-12.569926	1.720023	2.072749
6	-11.922403	1.033757	1.046743
6	-10.596561	0.640836	1.197424
6	-10.106972	-2.623116	3.104141
6	-11.044647	-3.250340	3.897806
6	-11.202414	-2.842691	5.218749
6	-10.405032	-1.814949	5.714876
6	-9.468264	-1.227035	4.891968
1	-2.060710	-0.593938	-3.216669
1	-1.116578	-1.155153	-0.976731
1	-2.658364	-1.794516	0.863357
1	-4.504838	-0.692599	-3.592975
1	-7.266282	-2.816810	2.490205
1	-6.144647	0.533413	0.029490
1	-4.879474	0.474180	5.092602
1	-10.035145	1.843381	4.329218
1	-12.384509	2.541452	4.054830
1	-13.605133	2.027467	1.953978
1	-12.450166	0.802934	0.125690
1	-10.096899	0.103635	0.395739
1	-9.948432	-2.869078	2.061684
1	-11.648945	-4.041674	3.469689
1	-11.944	-3.320422	5.855615
1	-10.491990	-1.471769	6.739230
1	-8.798334	-0.440194	5.214748
1	-6.080823	0.665021	3.921540
7	-5.095104	0.381578	4.098610
1	-7.936927	2.060895	3.577948
1	-4.460084	0.975307	3.562725
1	-4.962078	-0.590925	3.814890
1	-6.502098	-1.136136	-2.685586
1	-7.915260	1.682831	0.898549

IJ‡

6	-2.640300	-0.988239	-2.397361
6	-2.075744	-1.237027	-1.144716
6	-2.908764	-1.523148	-0.075362
6	-4.304526	-1.539777	-0.218548
6	-4.858657	-1.309286	-1.493312
6	-4.016089	-1.031455	-2.574114
6	-5.100398	-1.880704	0.987314
6	-6.372069	-1.185674	1.297890
6	-7.288097	-1.788792	2.197812
8	-6.197578	-1.403286	-1.645161
8	-4.681078	-2.706865	1.793783
6	-6.682434	0.061413	0.821650
7	-7.793522	0.699562	1.239212
6	-8.389796	-1.110827	2.612676
6	-8.602998	0.278202	2.266533
7	-9.334038	-1.749340	3.492256
6	-9.974951	0.876315	2.283674
8	-7.873901	1.083500	3.802346
6	-10.667832	1.120341	3.474988
6	-11.962680	1.624137	3.432222
6	-12.578766	1.883203	2.209326
6	-11.896050	1.631288	1.023084
6	-10.600200	1.124842	1.055950
6	-10.481371	-2.247539	2.978223
6	-11.392055	-2.876168	3.800183
6	-11.114311	-2.993746	5.159371
6	-9.926039	-2.474227	5.665327
6	-9.044577	-1.848145	4.809488
1	-2.003130	-0.765569	-3.248820
1	-0.999251	-1.208356	-1.009082
1	-2.494435	-1.724675	0.908306

1	-4.456058	-0.861891	-3.554026
1	-7.118385	-2.810349	2.526265
1	-6.080985	0.599421	0.099116
1	-5.085140	0.922161	4.988614
1	-10.187818	0.937846	4.430020
1	-12.489867	1.820204	4.361305
1	-13.590152	2.278502	2.182236
1	-12.370891	1.823312	0.065469
1	-10.087216	0.908324	0.123118
1	-10.622131	-2.127718	1.910662
1	-12.303744	-3.272096	3.368527
1	-11.818990	-3.488665	5.820013
1	-9.674916	-2.547269	6.717017
1	-8.106606	-1.411153	5.128420
1	-6.784550	0.916117	3.867966
7	-5.379959	0.683274	4.044307
1	-8.013864	2.041206	3.739487
1	-4.810267	1.234792	3.406591
1	-5.133124	-0.292603	3.892533
1	-6.434808	-1.228163	-2.566529
1	-7.973488	1.625786	0.862907

J

6	-2.710795	-1.344464	-2.610024
6	-2.073890	-1.084113	-1.392900
6	-2.822865	-1.073075	-0.230711
6	-4.211258	-1.294640	-0.253269
6	-4.835331	-1.577437	-1.486797
6	-4.073881	-1.594843	-2.658825
6	-4.912071	-1.267872	1.040862
6	-6.349088	-0.848760	1.149361
6	-7.130471	-1.379091	2.173792
8	-6.152207	-1.863295	-1.496757
8	-4.336547	-1.530067	2.090939
6	-6.903648	0.136945	0.355163
7	-8.144661	0.568315	0.609051
6	-8.422856	-0.919139	2.377176
6	-8.952963	0.106965	1.591329
7	-9.208187	-1.518823	3.419997
6	-10.279893	0.722109	1.721896
6	-10.762140	1.112483	2.979243
6	-12.004630	1.725241	3.078267
6	-12.774634	1.940739	1.936569
6	-12.298085	1.551058	0.686740
6	-11.051951	0.947419	0.573040
6	-10.361242	-2.157298	3.106880
6	-11.112941	-2.738070	4.103240
6	-10.670450	-2.665167	5.422626
6	-9.473122	-2.017857	5.711309
6	-8.742753	-1.444114	4.690645
1	-2.136592	-1.358452	-3.532236
1	-1.006558	-0.890796	-1.360283
1	-2.355081	-0.871564	0.728307
1	-4.564855	-1.817917	-3.602854
1	-6.730642	-2.171846	2.798842
1	-6.384399	0.629680	-0.457618
1	-5.402968	-0.424134	5.754400
1	-10.157125	0.972641	3.869707
1	-12.369389	2.040750	4.050981
1	-13.748231	2.414603	2.021631
1	-12.898642	1.712719	-0.203166
1	-10.693642	0.626825	-0.401739
1	-10.632848	-2.189794	2.058435
1	-12.031386	-3.248453	3.837868
1	-11.253321	-3.117954	6.218422
1	-9.095465	-1.950419	6.725086
1	-7.778965	-0.928374	4.827844
7	-5.831142	-0.196582	4.860451
1	-5.768862	0.814789	4.775157
1	-5.208563	-0.574071	4.148724
1	-6.445404	-2.025495	-2.404822
1	-8.498014	1.333591	0.035208

JK‡

6	-2.674606	-1.352970	-2.583869
6	-2.028219	-1.473211	-1.350533
6	-2.786947	-1.645367	-0.206580
6	-4.190883	-1.675303	-0.258867
6	-4.827243	-1.575236	-1.513460
6	-4.057935	-1.410419	-2.668367
6	-4.902438	-1.875782	1.018240
6	-6.251208	-1.263858	1.268177
6	-7.094599	-1.853965	2.207323
8	-6.170630	-1.682987	-1.567393
8	-4.399149	-2.501404	1.941467
6	-6.662476	-0.079790	0.682460
7	-7.836857	0.456903	1.027326
6	-8.316503	-1.270602	2.506433
6	-8.708547	-0.069168	1.914469
7	-9.201741	-1.946242	3.417262
6	-9.968774	0.648387	2.142338
6	-10.408620	0.922216	3.444489
6	-11.592667	1.622958	3.634640
6	-12.344221	2.042995	2.538160
6	-11.906973	1.771917	1.243539
6	-10.718644	1.080778	1.039677
6	-10.354051	-2.475934	2.943867
6	-11.199146	-3.153731	3.794740
6	-10.851051	-3.290619	5.136004
6	-9.656593	-2.743046	5.596331
6	-8.838935	-2.069233	4.715314
1	-2.093830	-1.219501	-3.492342
1	-0.945519	-1.430869	-1.289760
1	-2.310914	-1.741246	0.764730
1	-4.560406	-1.339120	-3.629882
1	-6.802668	-2.785032	2.685960
1	-6.073757	0.483325	-0.030950
1	-7.168593	2.836592	4.192944
1	-9.811995	0.624541	4.301248
1	-11.925636	1.847312	4.643452
1	-13.272412	2.585160	2.694157
1	-12.493258	2.094702	0.388616
1	-10.387536	0.853655	0.029697
1	-10.551102	-2.342549	1.886880

1	-12.115985	-3.573554	3.398173
1	-11.505997	-3.822819	5.818382
1	-9.352491	-2.829048	6.632859
1	-7.900712	-1.607924	5.569
7	-7.013571	2.456942	3.261943
1	-6.887759	3.270356	2.664059
1	-6.097154	2.017347	3.304887
1	-6.472183	-1.579119	-2.481133
1	-8.071513	1.364460	0.627512

K

6	-2.601096	-1.162012	-2.330321
6	-1.992509	-1.471860	-1.112127
6	-2.787225	-1.805736	-0.027599
6	-4.187274	-1.810374	-0.120912
6	-4.786090	-1.518123	-1.362385
6	-3.982507	-1.192460	-2.459317
6	-4.947132	-2.194278	1.093912
6	-6.226032	-1.512349	1.445630
6	-7.136821	-2.165015	2.269950
8	-6.128822	-1.606109	-1.468361
8	-4.504614	-3.042812	1.861404
6	-6.536687	-0.209692	1.048861
7	-7.632005	0.435835	1.432651
6	-8.293180	-1.500437	2.640688
6	-8.521668	-0.175455	2.228647
7	-9.269295	-2.225434	3.418816
6	-9.713119	0.621531	2.601630
6	-10.229478	0.631166	3.903956
6	-11.330422	1.423199	4.214321
6	-11.933267	2.203873	3.230652
6	-11.421104	2.203294	1.934147
6	-10.313074	1.424442	1.622392
6	-10.484981	-2.476251	2.882654
6	-11.427119	-3.181237	3.600477
6	-11.110496	-3.637866	4.876794
6	-9.849	-3.377045	5.404026
6	-8.938381	-2.662509	4.654581
1	-1.993829	-0.901184	-3.192794
1	-0.911665	-1.451609	-1.014814
1	-2.338786	-2.052141	0.930562
1	-4.456873	-0.975151	-3.413451
1	-6.956932	-3.187757	2.589233
1	-5.853491	0.347645	0.412520
1	-9.754265	0.050689	4.689123
1	-11.713285	1.433722	5.230788
1	-12.797072	2.815819	3.475073
1	-11.885202	2.812415	1.163618
1	-9.907236	1.425242	0.615268
1	-10.655327	-2.107297	1.878295
1	-12.394128	-3.373057	3.150300
1	-11.840348	-4.195585	5.454794
1	-9.564019	-3.715644	6.393265
1	-7.945343	-2.408301	5.004969
1	-6.402431	-1.368981	-2.365421

K’

6	-2.692154	-1.414396	-2.414242
6	-2.017935	-1.789955	-1.230052
6	-2.756970	-1.954199	-0.080452
6	-4.154277	-1.737725	-0.042492
6	-4.876877	-1.389527	-1.254496
6	-4.052029	-1.222752	-2.427916
6	-4.813197	-1.958502	1.233441
6	-6.150427	-1.346729	1.531153
6	-7.058361	-2.038163	2.319233
8	-6.136023	-1.265125	-1.324684
8	-4.300358	-2.615127	2.153038
6	-6.493144	-0.043895	1.155808
7	-7.614947	0.561916	1.524360
6	-8.254429	-1.419828	2.662303
6	-8.513042	-0.098533	2.274558
7	-9.230970	-2.195013	3.391036
6	-9.741473	0.651779	2.629530
6	-10.285032	0.625903	3.920241
6	-11.421434	1.371942	4.218090
6	-12.031836	2.144759	3.232874
6	-11.492559	2.180640	1.947901
6	-10.351101	1.445515	1.649161
6	-10.418824	-2.474252	2.809255
6	-11.360333	-3.225787	3.479811
6	-11.070782	-3.701976	4.755278
6	-9.836198	-3.412877	5.329302
6	-8.926595	-2.651225	4.626236
1	-2.123627	-1.277207	-3.332819
1	-0.941967	-1.937161	-1.228944
1	-2.269221	-2.239778	0.849069
1	-4.565703	-0.946795	-3.346731
1	-6.853233	-3.057449	2.634382
1	-5.807043	0.542500	0.551105
1	-9.804213	0.050655	4.706150
1	-11.825670	1.353579	5.226280
1	-12.922065	2.721983	3.467021
1	-11.961996	2.783738	1.175699
1	-9.925102	1.474947	0.650695
1	-10.568215	-2.088750	1.807765
1	-12.305093	-3.438559	2.993221
1	-11.852	-4.296732	5.295897
1	-9.571900	-3.765280	6.319478
1	-7.954794	-2.372467	5.015372

Formylchromone

6	-10.863344	-1.187498	-0.112640
6	-10.297382	-2.466882	-0.190230
6	-8.921986	-2.617586	-0.184629
6	-8.085099	-1.496110	-0.101248
6	-8.679427	-0.232214	-0.023616
6	-10.059789	-0.060840	-0.028811
6	-6.620062	-1.625743	-0.092640
6	-5.910855	-0.353643	0.947
6	-6.603265	0.815239	0.070621
8	-7.922923	0.910936	0.061142
8	-6.047806	-2.715686	-0.157748
6	-4.445920	-0.315335	0.022784

1	-3.957450	-1.303376	-0.037253
8	-3.787423	0.710436	0.112
1	-11.943198	-1.071536	-0.117886
1	-10.940528	-3.339290	-0.254414
1	-8.461459	-3.599204	-0.243977
1	-10.475426	0.939894	0.032085
1	-6.118658	1.784439	0.140501

Kröhnke salt

6	1.474120	-6.501592	-1.087320
6	2.882165	-6.654983	-0.517728
8	1.165516	-5.471239	-1.661957
7	3.641722	-5.421668	-0.708689
6	4.238767	-5.196544	-1.895657
6	4.909723	-4.013753	-2.126693
6	4.951107	-3.048905	-1.124712
6	4.312699	-3.296278	0.086789
6	3.655168	-4.494996	0.269817
6	0.535559	-7.632473	-0.918042
6	-0.755898	-7.497565	-1.446611
6	-1.676097	-8.527239	-1.313443
6	-1.314785	-9.701639	-0.652068
6	-0.032554	-9.844099	-0.125222
6	0.892889	-8.814546	-0.255475
1	2.845709	-6.879207	0.550238
1	4.168577	-5.991189	-2.629541
1	5.391600	-3.860256	-3.085201
1	5.476156	-2.112957	-1.286785
1	4.318367	-2.571263	0.892390
1	3.135084	-4.752149	1.185199
1	-1.021514	-6.577924	-1.958879
1	-2.675809	-8.417895	-1.723631
1	-2.035453	-10.507999	-0.547145
1	0.247057	-10.758708	0.389203
1	1.887007	-8.943965	0.162032
1	3.405117	-7.473972	-1.018868

Ylide

6	1.522117	-6.642147	-1.557259
6	2.373008	-6.157031	-0.561708
8	1.451483	-6.230897	-2.752097
7	3.327795	-5.156355	-0.739709
6	3.543813	-4.540493	-1.940168
6	4.504249	-3.557235	-2.072381
6	5.275783	-3.164705	-0.985546
6	5.043661	-3.798418	0.234764
6	4.082065	-4.775363	0.335874
6	0.580089	-7.740077	-1.140484
6	-0.611282	-7.884509	-1.861175
6	-1.524894	-8.882003	-1.537469
6	-1.254528	-9.765058	-0.493157
6	-0.063392	-9.642775	0.220137
6	0.847137	-8.639395	-0.100109
1	2.319966	-6.520317	0.453377
1	2.905801	-4.889323	-2.742557
1	4.633836	-3.103435	-3.049415
1	6.031697	-2.392711	-1.081037

1	5.608647	-3.541608	1.124480
1	3.871886	-5.293637	1.263233
1	-0.809348	-7.198938	-2.679817
1	-2.449394	-8.972370	-2.101924
1	-1.963949	-10.548880	-0.241543
1	0.163391	-10.337635	1.024502
1	1.782365	-8.577685	0.449189

Product

6	-4.328771	0.035234	-3.386601
6	-3.072574	-0.580201	-3.318306
6	-2.199220	-0.228017	-2.306214
6	-2.547553	0.740657	-1.343987
6	-3.806624	1.390162	-1.460263
6	-4.694724	1.009176	-2.473071
6	-1.652732	1.130417	-0.253585
6	-0.443079	0.337904	0.090218
6	0.705265	1.015426	0.487071
8	-4.182627	2.362692	-0.624423
8	-1.889090	2.145442	0.428461
6	-0.435279	-1.065	0.137217
7	0.621443	-1.749350	0.557519
6	1.843244	0.306721	0.887664
6	1.734052	-1.110048	0.934757
7	3.013584	0.972986	1.138520
6	2.854453	-1.977393	1.379262
6	3.534611	-1.748403	2.583234
6	4.556224	-2.601832	2.992309
6	4.914043	-3.694341	2.205956
6	4.235946	-3.936867	1.012183
6	3.211545	-3.088865	0.604952
1	-5.023592	-0.240942	-4.175293
1	-2.784080	-1.322755	-4.055296
1	-1.217186	-0.687026	-2.262887
1	-5.657746	1.507433	-2.530862
1	0.730652	2.101824	0.460567
1	-1.318927	-1.636037	-0.124479
1	3.244333	-0.918094	3.222840
1	5.066648	-2.415760	3.933368
1	5.714008	-4.357238	2.524114
1	4.506346	-4.789922	0.395620
1	2.679385	-3.279605	-0.322785
1	3.739622	0.494008	1.647274
1	2.936668	1.957100	1.351357
1	-3.397966	2.538678	-0.040139

Conjugated imine

6	-13.518978	-0.712069	-0.111234
6	-13.618745	-2.073833	-0.158120
6	-14.691864	0.094191	-0.014869
6	-14.869	1.454123	0.039978
7	-12.599137	-2.944133	-0.237087
6	-13.696918	2.380186	0.007486
7	-13.875402	3.655856	0.062345
1	-12.532509	-0.254190	-0.149792
1	-14.603622	-2.537461	-0.122043
1	-15.628837	-0.465161	0.019005

1	-15.791606	1.897205	0.113341	1	-12.692665	1.943244	-0.067408
1	-12.772490	-3.931908	-0.320620	1	-12.966958	4.121591	0.024386
1	-11.645202	-2.625642	-0.314127				

4.2. 3-Aminopyridine Synthesis using $^{15}NH_4OAc$

In a 10 mL sealed microwave reactor, 3-formylchromone **1a** (1 equiv., 0.230 mmol, 40.0 mg), pyridinium salt **2a** (1.5 equiv., 0.345 mmol, 96.0 mg) and $^{15}NH_4OAc$ (15 equiv., 3.45 mmol, 271.9 mg) were added into EtOH (0.23 M, 1 mL). The vessel was placed in the microwave and the program was started. The program had two steps: 2 min at 50 °C, followed by 15 min at 250 PSI. When the program ended, the reaction was let cool down to room temperature. The crude was loaded into silica and purified by column chromatography using a gradient of 0% to 40% ethyl acetate in hexane obtaining the 3-aminopyridine (0,174 mmol, 50.7 mg) at 76% yield (Scheme S2).

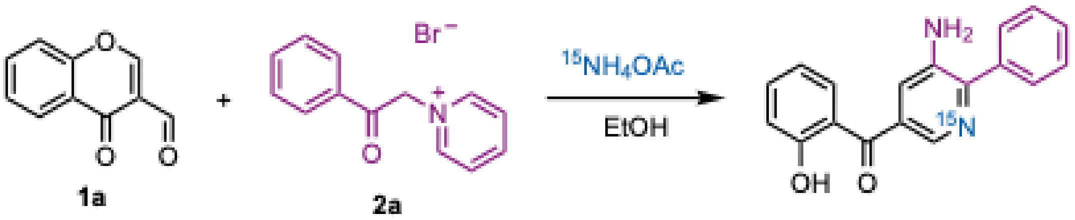

Scheme S2 – Control experiment: synthesis of the 3-aminopyridine **3a** using $^{15}NH4OAc$.

4.2.1. Characterization

By analyzing the ^{15}N-NMR (Figure S12) of the resultant ^{15}N-3-aminopyridine **3a**, it is possible to verify that the $^{15}NH_4OAc$ is only responsible for one of the two nitrogen atoms existent in the final molecule.

^{15}N NMR (51 MHz, $CDCl_3$): δ 312.9 ppm (Figure S12).

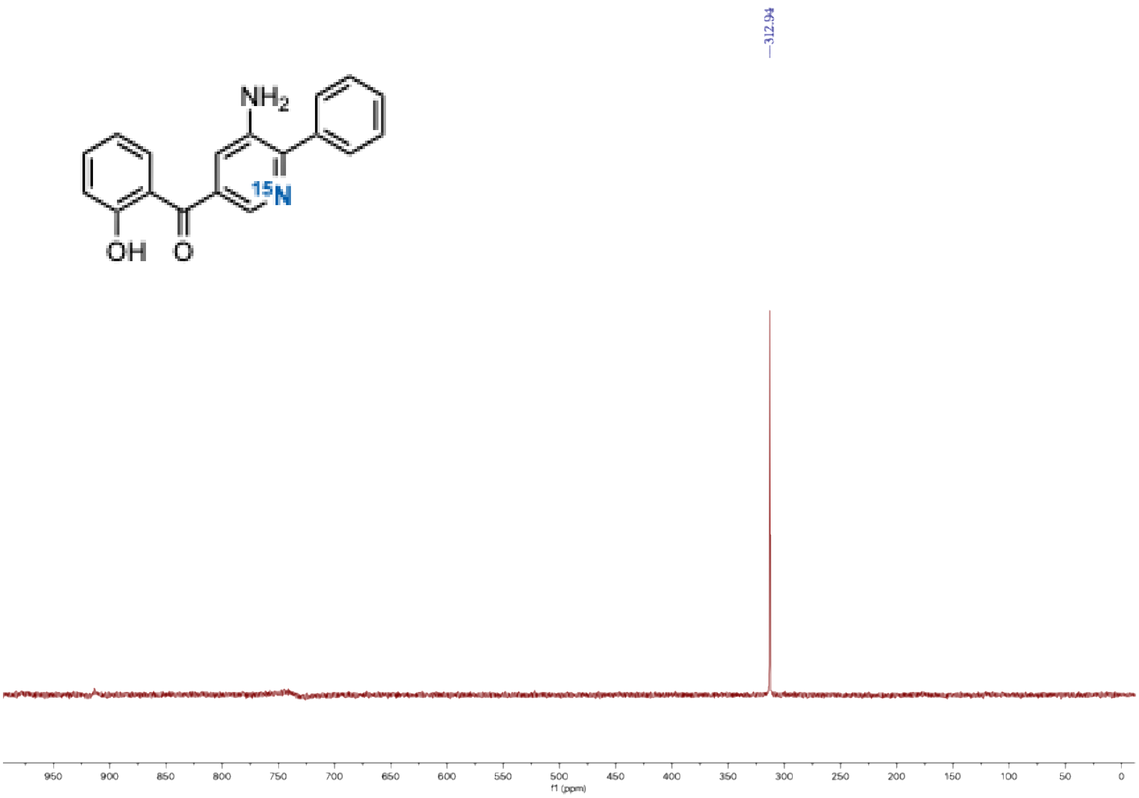

Figure S12 – ^{15}N-NMR (51 MHz, $CDCl_3$) of ^{15}N-3-aminopyridine **3a** synthesized using $^{15}NH_4OAc$.

Due to the chemical shift of the signal, we could propose that it would correspond to the nitrogen atom belonging to the pyridine ring. Nevertheless, deeper NMR analysis were conducted to undoubtedly identify to which nitrogen the signal correlates with.

Using the non-marked 3-aminopyridine **3a**, ^{15}N-NMR (Figure S13) was acquired allowing the detection of the two nitrogen signals. Copulated with ^{15}N–^{1}H HSQC (Figure S14) and HMBC (Figure S15), it was possible to assign the signals corresponding to each nitrogen.

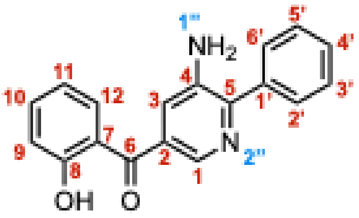

^{15}N NMR (51 MHz, $CDCl_3$): δ 313.2 (N-2''), 53.9 (N-1'') ppm (Figure S13).

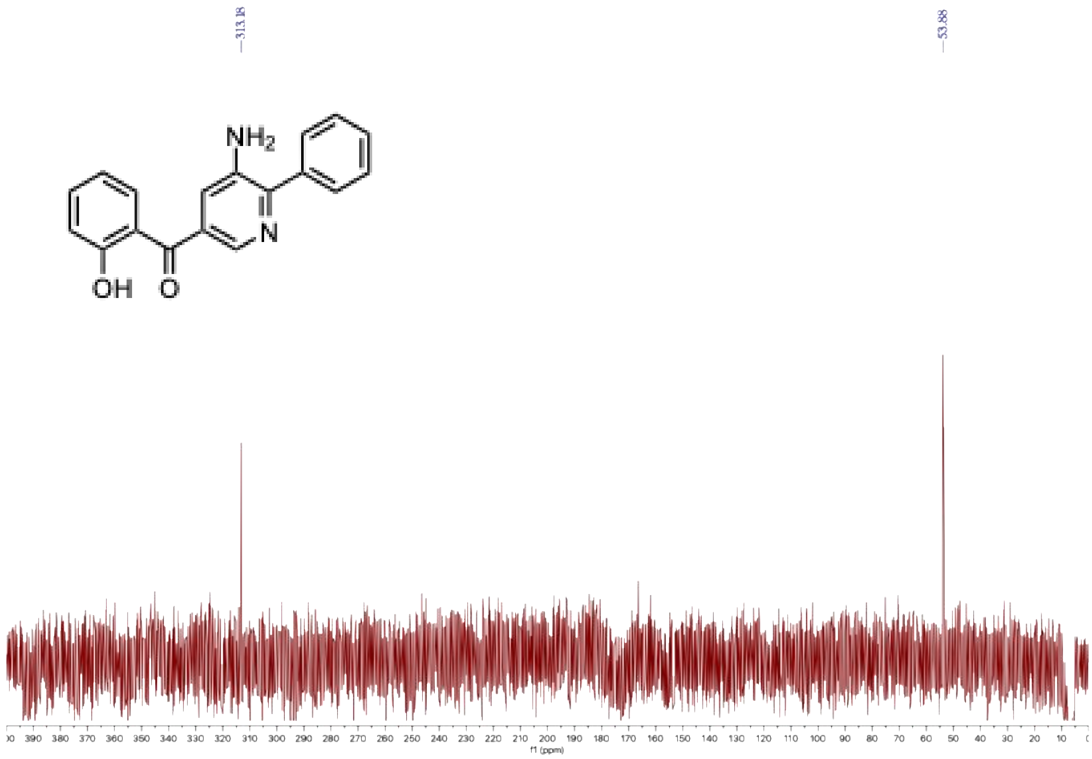

Figure S13 – ^{15}N-NMR (51 MHz, $CDCl_3$) of non-marked 3-aminopyridine **3a**.

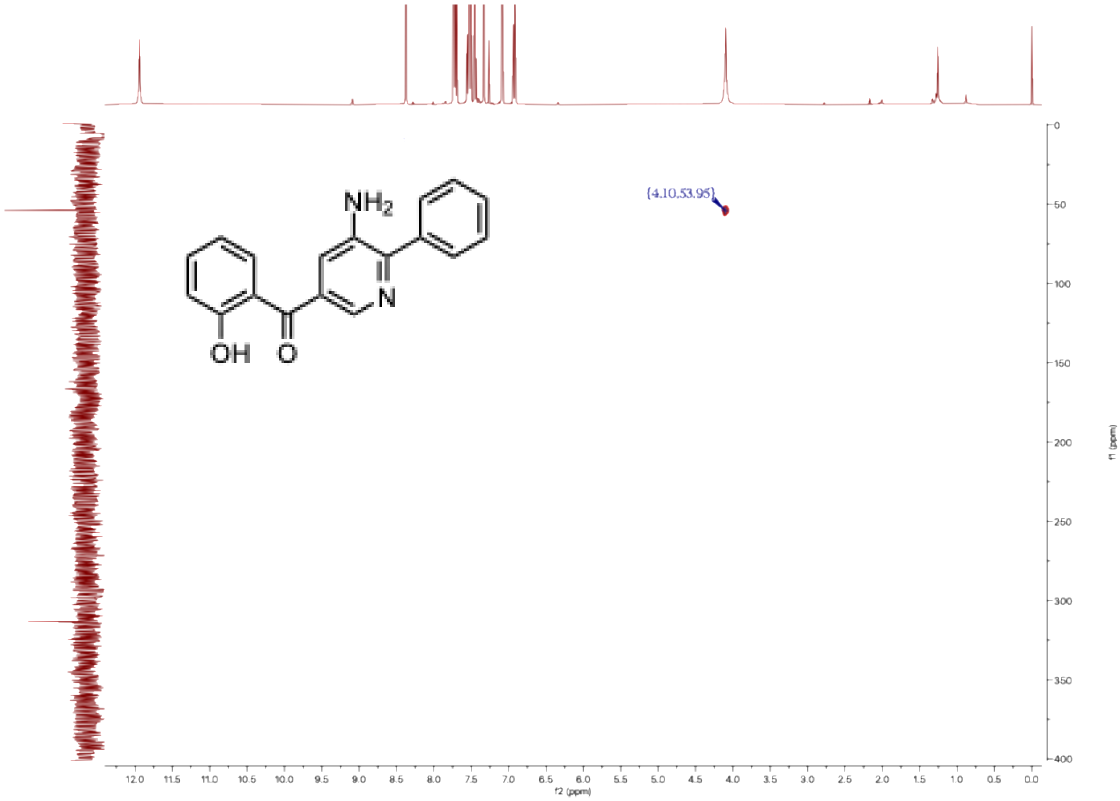

Figure S14 – ^{15}N–^{1}H HSQC of non-marked 3-aminopyridine **3a**.

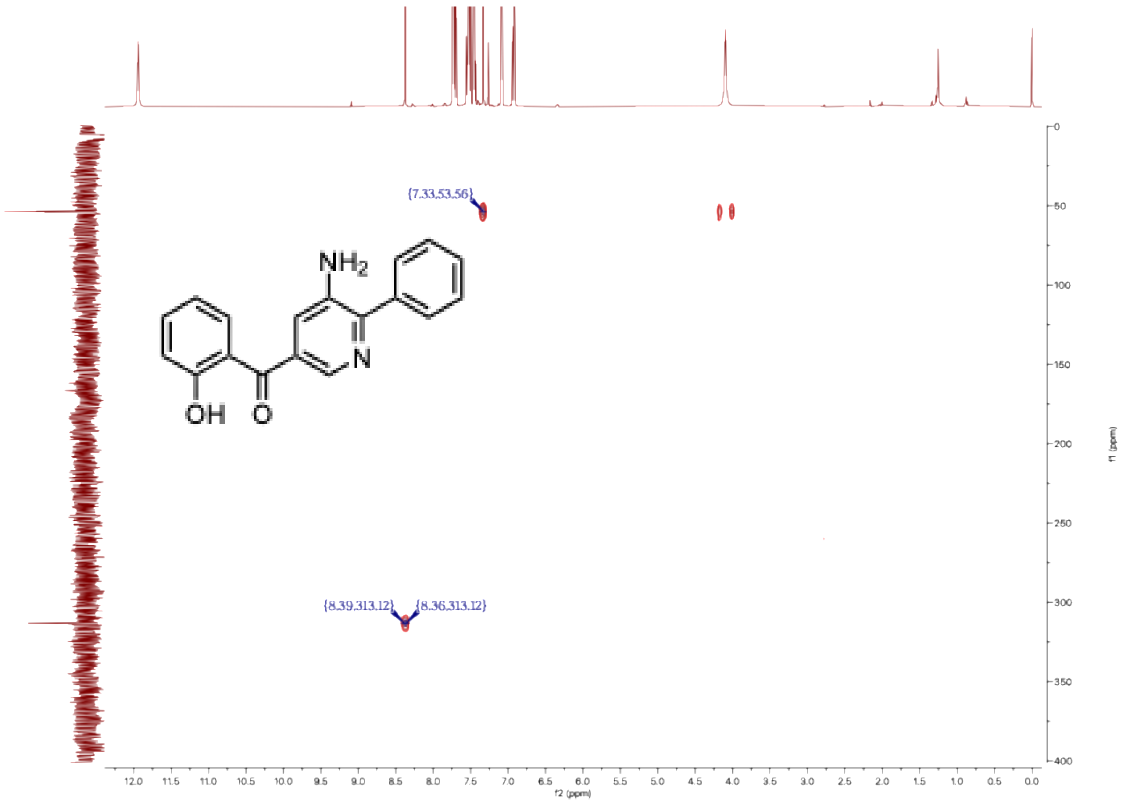

Figure S15 – ^{15}N–^{1}H HMBC of non-marked 3-aminopyridine **3a**.

4.3. HPLC-MS of reaction crude

The crude of the reaction for the synthesis of 3-aminopyridine **3a**, and the crude of the reaction using the $^{15}NH_4OAc$ were run through HPLC-MS analysis.

The analysis of the HPLC-MS of the crude reaction of the synthesis of 3-aminopyridine **3a** (Scheme S7) revealed the formation of the key intermediate, [1,3'-bipyridin]-1-ium [M^+] = 353.20, was detected (Figure S16). We could also identify the 3-aminopyridine **3a** and pyridine (Figure S16).

The analysis of the HPLC-MS of the crude reaction using $^{15}NH_4OAc$ (Scheme S2) allowed not only the observation of ^{15}N-[1,3'-bipyridin]-1-ium, but also the ^{15}N-pyridine which results from the Zincke reaction with $^{15}NH_4OAc$ (Figure S17).

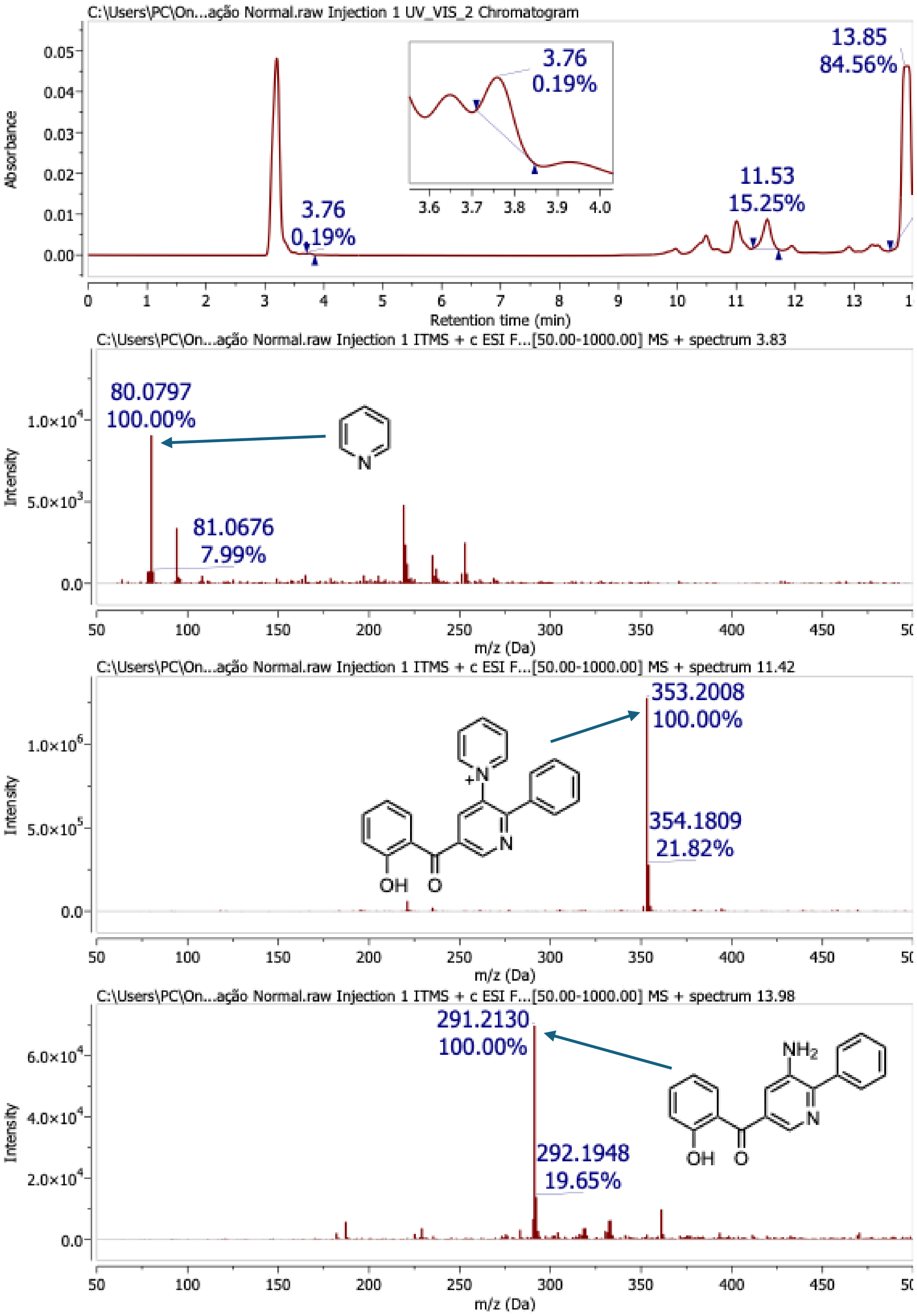

Figure S16 –HPLC-MS chromatogram of the crude reaction of the synthesis of 3-aminopyridine **3a**.

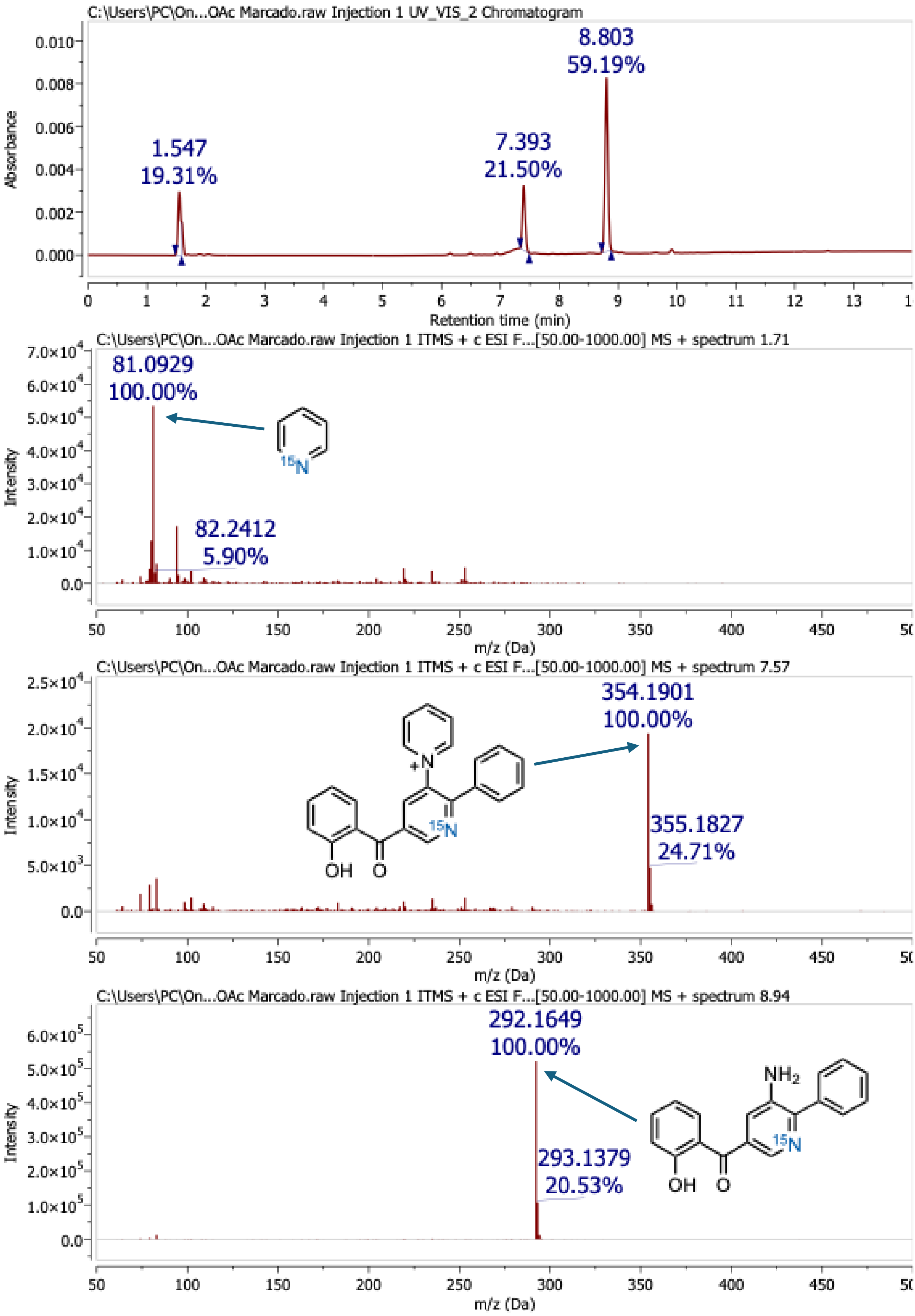

Figure S17 –HPLC-MS chromatogram of the crude reaction of 3-aminopyridine **3a** using $^{15}NH_4OAc$.

4.4. Control Reactions

4.4.1. 3-Aminopyridine Synthesis under Inert Atmosphere

In a 10 mL sealed microwave reactor under argon, 3-formylchromone **1a** (1 equiv., 0.459 mmol, 80.0 mg), pyridinium salt **2a** (1.5 equiv., 0.689 mmol, 191.7 mg) and NH_4OAc (15 equiv., 6.89 mmol, 531.1 mg) were added into EtOH (0.46 M, 1 mL). The vessel was closed and placed in the microwave and the program was started. The program had two steps: 2 min at 50 °C, followed by 15 min at 250 PSI. When the program ended, the reaction was let to cool down to room temperature. The crude was loaded into silica and purified by column chromatography using a gradient of 0% to 40% ethyl acetate in hexane obtaining the 3-aminopyridine **3a** (0.428 mmol, 214.4 mg) in 93% yield (Scheme S3).

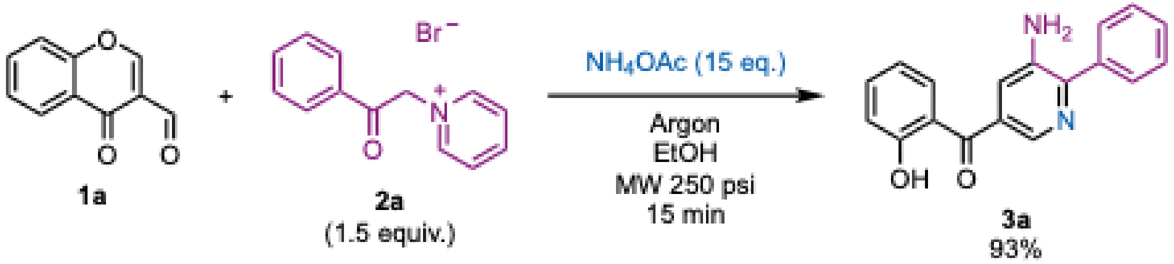

Scheme S3 - Synthesis of 3-aminopyridine **3a** under inert atmosphere.

4.4.2. Reaction with 3-Formylchromene: Pyrimidine Synthesis and Characterization

In a 10 mL sealed microwave reactor, the 4*H*-chromene-3-carbaldehyde **6** (1 equiv., 0.459 mmol, 73.7 mg), pyridinium salt **2a** (1.5 equiv., 0.689 mmol, 191.7 mg) and NH_4OAc (15 equiv., 6.89 mmol, 531.1 mg) were added into EtOH (0.46 M, 1 mL). The vessel was placed in the microwave and the program was started. The program had two steps: 2 min at 50 °C, followed by 15 min at 250 PSI. When the program ended, the reaction was let to cool down to room temperature. The crude was loaded into silica and purified by column chromatography using a gradient of 0% to 40% ethyl acetate in hexane obtaining the pyrimidine **7** (0.0503 mmol, 13.9 mg) in 11% yield (Scheme S4). The postulated mechanism for the synthesis of pyrimidine **7** is depicted in Scheme S5.[11]

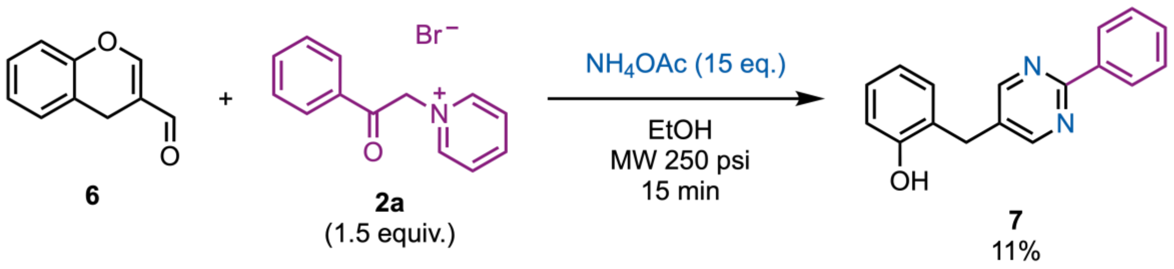

Scheme S4 - Synthesis of pyrimidine **7**.

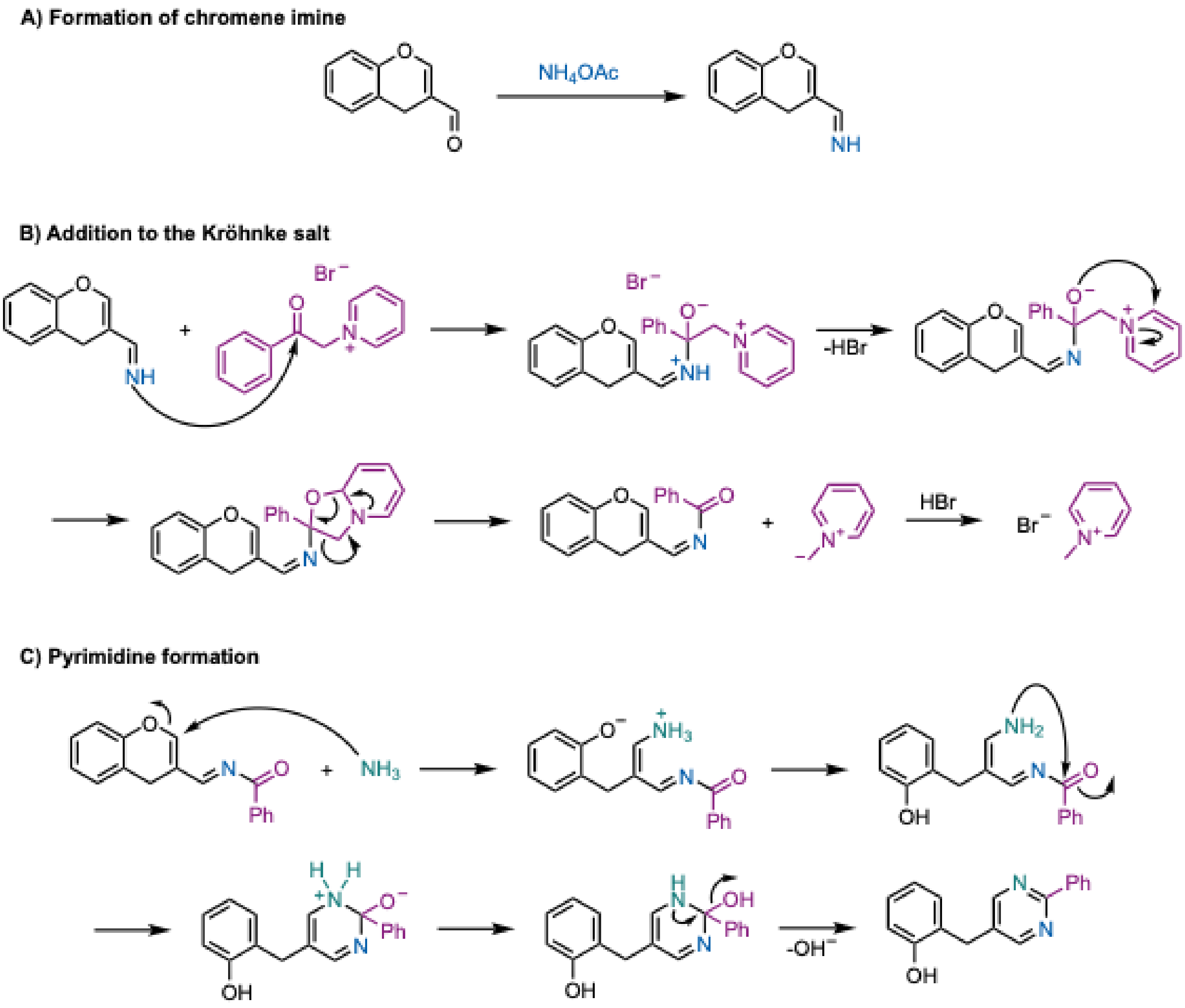

Scheme S5 – Postulated mechanism for the synthesis of pyrimidine **7**.

2-[(2-phenylpyrimidin-5-yl)methyl]phenol (7)

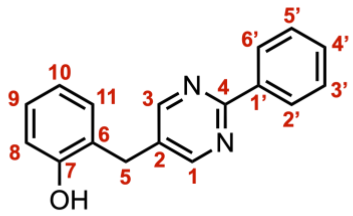

7 Yellow solid. Melting point of 165.5–167.9 °C.

^{1}H NMR (300 MHz, $CDCl_3$): δ 8.80 (s, H-1 and 3, 1H), 8.45 – 8.30 (m, H-2' and 6', 2H), 7.57 – 7.39 (m, H-3', 4' and 5', 3H), 7.18 (dd, *J* = 7.5, 1.7 Hz, H-11, 1H), 7.06 (ddd, *J* = 7.9, 7.7, 1.7 Hz, H-9, 1H), 6.85 (ddd, *J* = 7.7, 7.5, 1.2 Hz, H-10, 1H), 6.71 (dd, *J* = 7.9, 1.2 Hz, H-8, 1H), 4.00 (s, H-5, 2H) ppm.

^{13}C NMR (75 MHz, $CDCl_3$): δ 162.6 (C-4), 157.6 (C-1 and 3, 2C), 154.3 (C-7), 137.4 (C-1'), 132.5 (C-2), 130.7 (C-4 or 11'), 130.7 (C-4 or 11'), 128.9 (C-3' and 5', 2C), 128.6 (C-10), 128.1 (C-2' and 6', 2C), 125.7 (C-6), 120.8 (C-10), 115.6 (C-8), 31.3 (C-5) ppm.

HRMS (ESI) *m/z*: $[M + H]^+$ Calcd for $C_{17}H_{15}N_2O^+$ 263.1179 ; Found 263.1175.

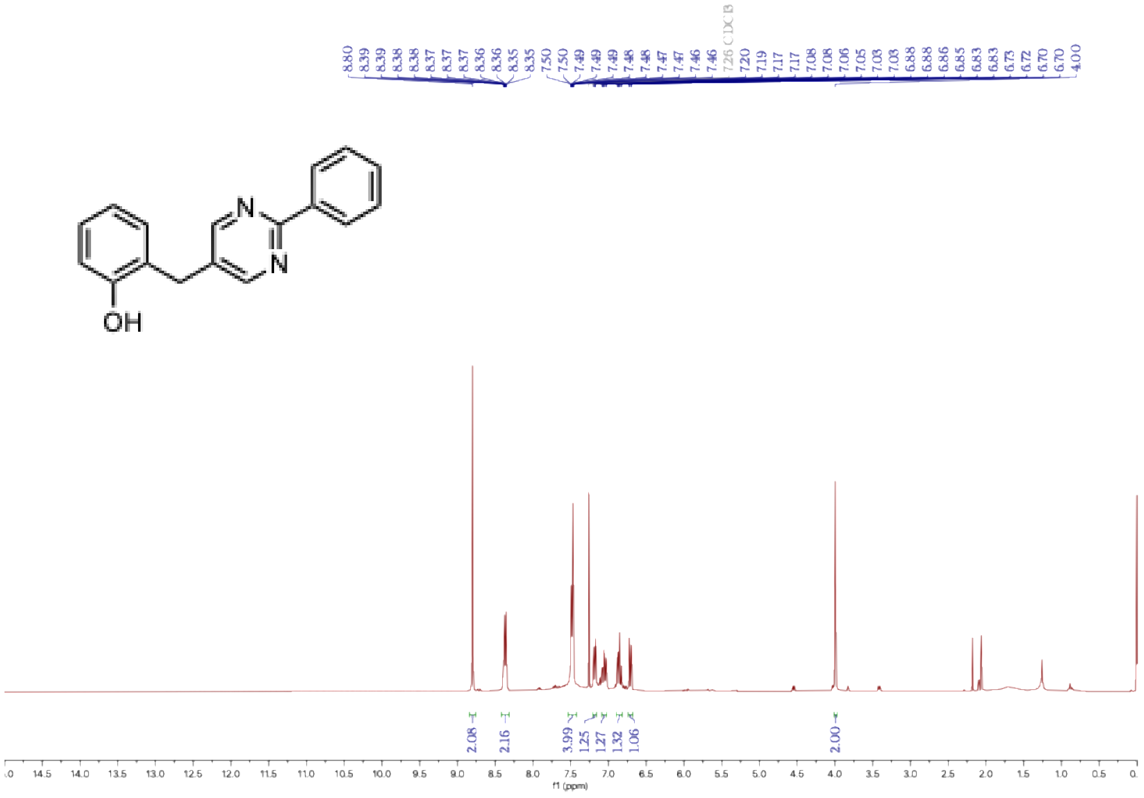

Figure S18 – ^{1}H-NMR (300 MHz, $CDCl_3$) of pyrimidine **7**.

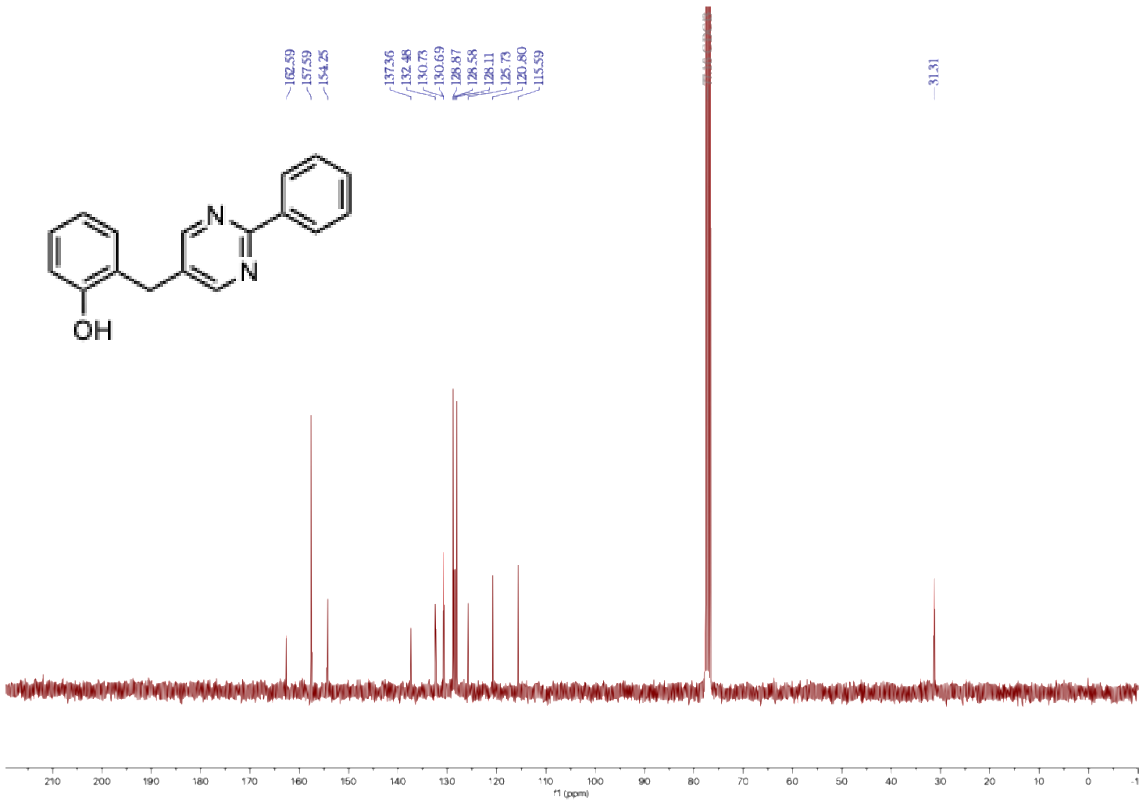

Figure S19 – ^{13}C-NMR (75 MHz, $CDCl_3$) of pyrimidine **7**.

5. Scalability Studies

5.1. Batch Procedure

In a 35 mL sealed microwave reactor, the 3-formylchromone **1a** (1 equiv., 5.75 mmol, 1.00 g), pyridinium salt **2a** (1.5 equiv., 8.74 mmol, 2.40 g) and NH_4OAc (15 equiv., 87.4 mmol, 6.64 g) were added into EtOH (0.46 M, 12.5 mL). The vessel was placed in the microwave and the program was started. The program had two steps: 2 min at 50 °C (50 W), followed by 15 min at 250 PSI (30 W) (Figure S20). When the program ended, the reaction was let to cool down to room temperature. The crude was loaded into silica and purified by column chromatography using a gradient of 0% to 40% ethyl acetate in hexane. After purification, the 3-aminopyridine **3a** (1.34 mg, 80% yield) was obtained (Scheme S6).

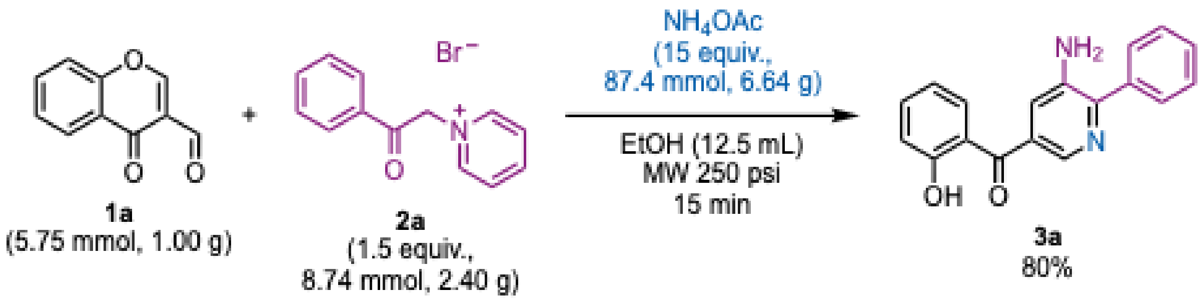

Scheme S6 - Synthesis of 3-aminopyridine **3a** at gram scale in batch.

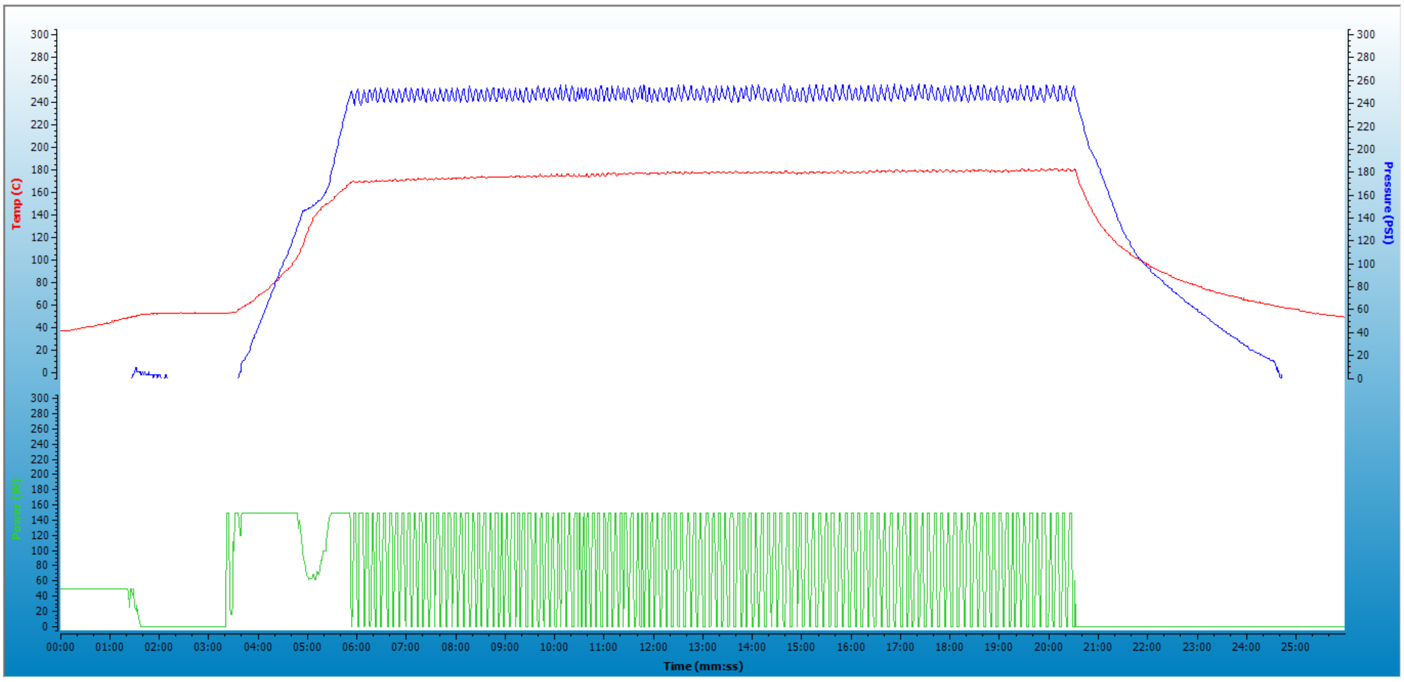

Figure S20 –Variation of temperature (°C) (red), pressure (PSI) (blue), and microwave power (W) (green) over time during a microwave-assisted synthesis of 3-aminopyridine **3a** at gram scale.

5.2. Continuous Flow Procedure

The flow setup (Figure S21) consists of an HPLC pump connected to an HPLC column filled with glass beads (2 mm) or sand inside a GC pump followed by a collection container.

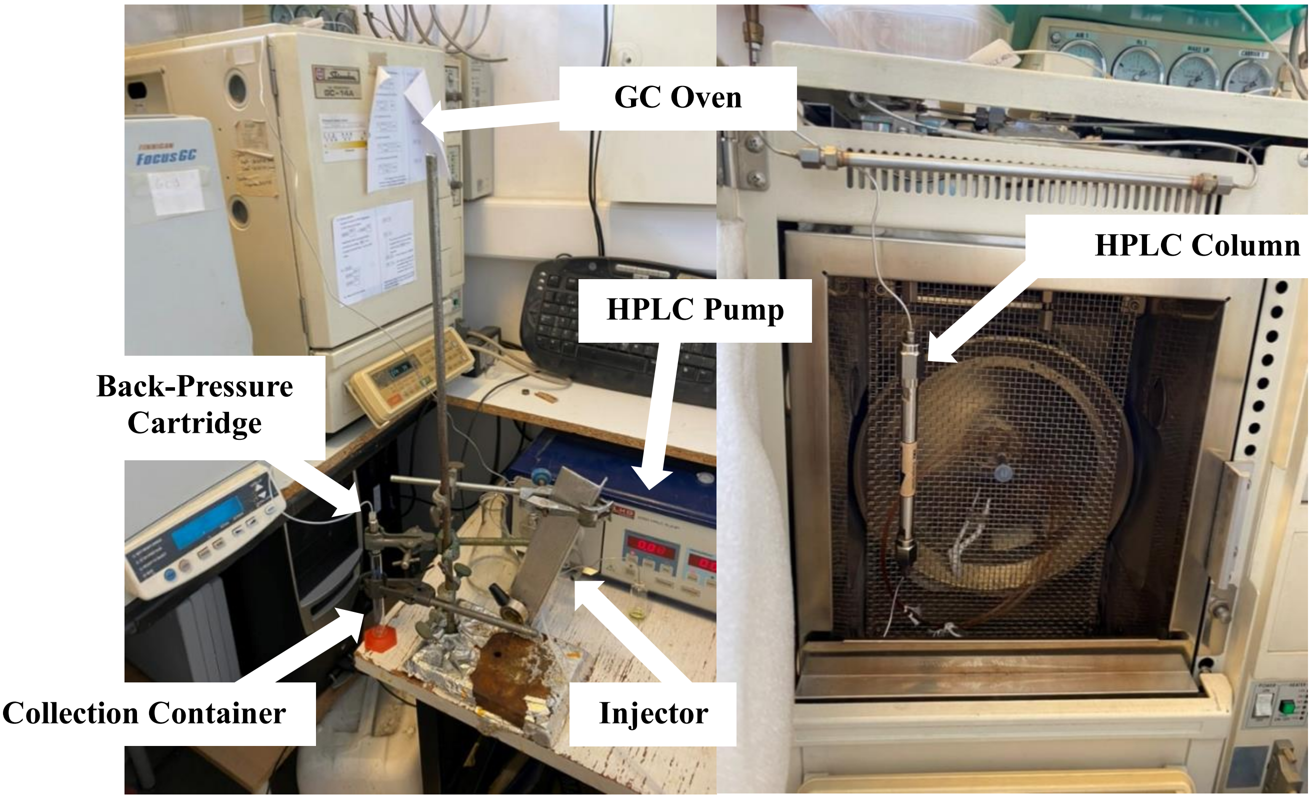

Figure S21 - Setup of the reaction for the synthesis of the 3-aminopyridine **3a** in flow.

In attempting to implement our method for the synthesis of 3-aminopyridines in a flow chemistry strategy, the solubility of all the components was investigated with the objective of finding an optimal feeding solution that could have 3-formylchromone **1a** (1 equiv.), pyridinium salt **2a** (1.5 equiv.) and NH_4OAc (15 equiv.) completely dissolved.

In the initial attempts, we tried to keep the 3-formylchromone **1a** at 80 mg/mL concentration (0.459 M). The following solvents were evaluated: EtOH, MeOH, HFIP, THF, toluene and H_2O. In the end, at this given concentration, it was impossible to dissolve the 3-formylchromone **1a** component.

In the subsequent attempts, we evaluated the dissolution of 3-formylchromone **1a** in multiple solvents in reduced concentration (20 and 10 mg/mL). The outcomes are summarized in the following Table S4, where we could observe that the 3-formylchromone **1a** could be dissolved in THF or 1,4-dioxane at 20 mg/mL (0.115 M).

Table S4 - Dissolution of 3-formylchromone **1a** in EtOH, ^{i}PrOH, THF and dioxane.

V / mL	EtOH	^{i}PrOH	THF	Dioxane
1	ND	ND	D	D
2	ND	ND		

D – Dissolved; ND – No-Dissolution

Then, we attempted to solubilize the other two reaction components asdepicted on Table S5. Separately, 47.9 mg of pyridinium salt **2a** and 132.8 mg of NH_4OAc were tried to be solubilized in multiple solvents in different volumes. In the end, both components were effectively solubilized in 1 mL of H_2O or 2 ml of EtOH.

Table S5 - Dissolution of Kröhnke salt **2a** and ammonium acetate in ethanol, isopropanol, tetrahydrofuran, dioxane and water.

V / mL	Pyridinium salt (2a)					NH_4OAc				
	H_2O	EtOH	^{i}PrOH	THF	Diox.	H_2O	EtOH	^{i}PrOH	THF	Diox.
1	D	ND	ND	ND	ND	D	ND	ND	ND	ND
2		D	ND	ND	ND		D	ND	ND	ND

D – Dissolved; ND – Non-Dissolution

Knowing that THF and Dioxane were the optimal solvent for the solubilization of 1a, and that H2O and EtOH were required for the solubilization of 2a and NH4OAc, we set out to find a solvent mixture that could dissolve the required reagents (Table S6). Namely, 1 mL of THF or dioxane were mixed with increasing amounts of H_2O or EtOH. Although the mixtures of THF/H_2O and THF/EtOH were able to dissolve all the components, phase separation was observed in both mixtures. In contrast, both dioxane:H_2O (2:1) with **1a** at 13.3 mg/mL; and dioxane:EtOH (1:2) with **1a** at 6.67 mg/mL were able to dissolve the components without phase separation. The NH_4OAc was the most troublesome component to be dissolved.

Table S6 - Dissolution of all the components in mixtures with tetrahydrofuran, dioxane, water and ethanol.

1 mL THF				1 mL Dioxane			
H_2O		EtOH		H_2O		EtOH	
V / mL	Results	V / mL	Results	V / mL	Results	V / mL	Results
0.00	ND	0.00	ND	0.00	ND	0.00	ND
0.25	D*	0.25	D*	0.25	ND	0.25	ND
				0.50	D	0.50	ND
						0.75	ND
						1.00	ND
						1.25	ND
						1.50	ND
						1.75	ND
						2.00	D

D – Dissolved; ND – Non-Dissolution; * – Two Phases

With the solubility of the reagents properly evaluated we accessed their potential under standard batch conditions using microwave. Interestingly, both dioxane:EtOH (1:2) and dioxane:H_2O (2:1) yielded the desired product in 68 and 62 % yield respectively. The latter has the advantage of the higher boiling point of water, improved greenness and improved dissolution. This could allow for higher productivities under continuous flow conditions.

Focusing on the mixture of dioxane:H_2O (2:1) we screened different conditions under continuous flow, namely concentration , temperature, solvents, flow rates and the filling of the packed-bed reactor (Table S7).

General method for the optimization studies under flow conditions (Table S7): The packed bed reactor was prepared by filling an empty HPLC column (L = 15 cm, ID = 4.6 mm) with glass beads (D = 2 mm) or sand. The reactor volume was determined by the weight increase after filling with water. The reactor was connected to the set-up described in Figure S21 consisted by an HPLC Pump (LKB, Mod. 2510), HPLC loop (1 mL), GC oven and back-pressure cartridge (34 bar). For each experiment (entries 1-31) the system was run for a specific concentration, solvent system, temperature and flow for 10 to 20 min before reaching the steady-state. Then the reaction solution consisted of **1a**, **2a** and NH_4OAc was inject thru the HPLC loop. The reaction mixture was collected (3 to 5 mL), diluted to the desired reaction concentration, and quantified by HPLC.

Quantification method: Using an Thermo Scientific™ Ultimate™ 3000 with a Kinetex® 5 µm C18 100 Å, LC Column 150 x 3.0 mm, 20 µL were inject. The method, with a flow of 1 mL/min started with a mixture of 5% of acetonitrile in water for 2 minutes, followed by a gradient to 95% acetonitrile in water for 10 minutes. After an isocratic 5 minutes washing step the eluent returned to the 5% acetonitrile in water gradient to equilibrate the system for 5 minutes.

The desired 3-aminopyridine **3a** eluted at 12.72 minutes (Figure S23) and was quantified through integration of the respective absorbance peak at 250 nm. The yield was determined using the calibration curve (Figure S22).

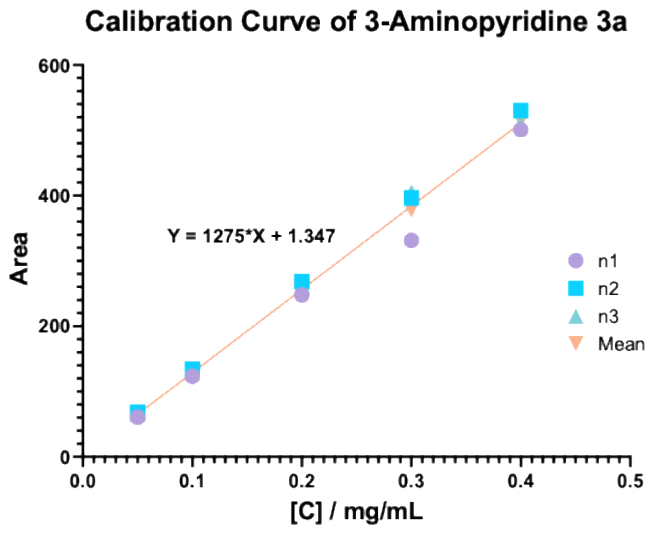

Figure S22 - Calibration curve for the 3-aminopyridine **3a**.

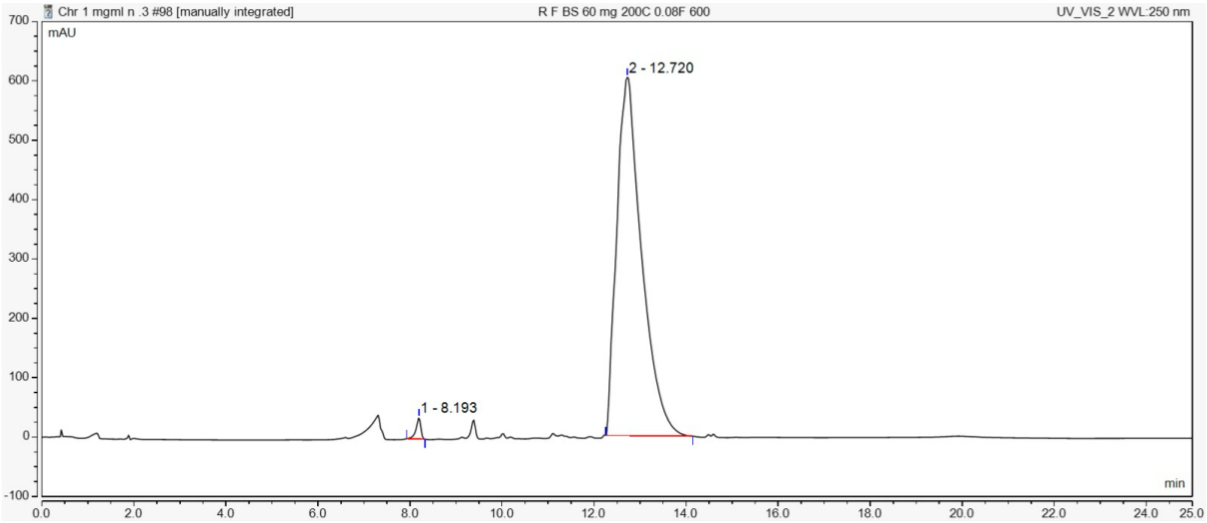

Figure S23 – Representative chromatogram of the optimal conditions (Table S7, Entry 29).

Table S7 - Optimization study for the synthesis of the 3-aminopyridine **3a** in flow.

Entry	[1a] / mg/mL [a]	T / °C	Solvent	Fill	Res. Time / min	Yield / %	Prod. / mmol/h
**1**	6,7	200	Dioxane/H_2O (2:1)	Glass beads [b]	19,42	71	0,16
**2**	6,7	180	Dioxane/H_2O (2:1)	Glass beads [b]	19,42	39	0,09
**3**	6,7	160	Dioxane/H_2O (2:1)	Glass beads [b]	19,42	15	0,03
**4**	6,7	190	Dioxane/H_2O (2:1)	Glass beads [b]	19,42	57	0,13
**5**	6,7	200	Dioxane/H_2O (2:1)	Glass beads [b]	12,95	58	0,20
**6**	6,7	200	Dioxane/H_2O (2:1)	Glass beads [b]	9,71	49	0,22
**7**	6,7	200	Dioxane/H_2O (2:1)	Glass beads [b]	6,47	35	0,24
**8**	6,7	200	Dioxane/H_2O (2:1)	Glass beads [b]	3,88	22	0,26
**9**	6,7	200	Dioxane/H_2O (2:1)	Sand [c]	18,24	75	0,17
**10**	6,7	200	Dioxane/H_2O (2:1)	Sand [c]	9,12	64	0,29
**11**	6,7	180	Dioxane/H_2O (2:1)	Sand [c]	18,24	55	0,13
**12**	6,7	200	Dioxane/H_2O (2:1)	Sand [c]	3,65	28	0,32
**13**	6,7	200	Dioxane/H_2O (2:1)	Sand [c]	6,08	53	0,37
**14**	6,7	200	Dioxane/H_2O (2:1)	Sand [c]	12,16	71	0,24
**15**	6,7	190	Dioxane/H_2O (2:1)	Sand [c]	18,24	68	0,16
**16**	6,7	160	Dioxane/H_2O (2:1)	Sand [c]	18,24	28	0,06
**17**	6,7	200	*ᵗ*BuOH/H_2O (2:1)	Sand [c]	18,24	55	0,13
**18**	6,7	210	*ᵗ*BuOH/H_2O (2:1)	Sand [c]	18,24	59	0,13
**19**	6,7	200	NMP/H_2O (2:1)	Sand [c]	18,24	67	0,15
**20**	6,7	200	NMP/H_2O (2:1)	Sand [c]	18,24	69	0,16
**21**	6,7	210	NMP/H_2O (2:1)	Sand [c]	18,24	67	0,15
**22**	6,7	220	NMP/H_2O (2:1)	Sand [c]	18,24	61	0,14
**23**	6,7	230	NMP/H_2O (2:1)	Sand [c]	18,24	65	0,15
**24**	6,7	240	NMP/H_2O (2:1)	Sand [c]	18,24	66	0,15
**25**	6,7	250	NMP/H_2O (2:1)	Sand [c]	18,24	65	0,15

**26**	6,7	200	Dioxane/H_2O (2:1)	Sand [c]	70,17	12	0,01
**27**	6,7	200	Dioxane/H_2O (2:1)	Sand [c]	50,68	73	0,06
**28**	6,7	200	Dioxane/H_2O (2:1)	Sand [c]	22,81	78	0,14
**29**	10,0	200	Dioxane/H_2O (2:1)	Sand [c]	22,81	84	0,23
**30**	8,3	200	Dioxane/H_2O (2:1)	Sand [c]	22,81	78	0,18
**31**	9,2	200	Dioxane/H_2O (2:1)	Sand [c]	22,81	81	0,20

a) Concentration of the formylchromone **1a** in the reaction mixture. The pyridinium salt **2a** and ammonium acetate were added at 1.5 and 15 equivalents, respectively.

b) Reactor volume: 1.9 mL.

c) Reactor volume: 1,8 mL.

Although we observed the best yield (84 %) when using 10.0 mg/mL of **1a** (Table S7, Entry 29), the feeding solution started to divide into two phases. In contrast, a small dilution to 9.2 mg/mL inhibited phase separation (Table S7, Entry 31). Focusing on this concentration, we achieved a yield of 81% with a 22 minutes residence time.. We scaled out the reaction by continuously feeding the reactor and after 319 minutes running, 327.3 mg were purified leading to an isolated yield of 93% for 3-aminopyridine **3a** (Table S8).

General method for the continuous flow experiment (Table S8): The continuous flow experiment was performed using the set-up presented in Figure S21 and the general conditions of entry 31, Table S7 by feeding a reaction solution of **1a** (9.17 mg/mL), **2a** (22.0 mg/mL) and NH_4OAc (60.9 mg/mL) in dioxane/water (2:1), reactor filled with sand, reactor volume (1.82 mL), 200 °C, 0.08 mL/min (residence time = 22.8 min). The reaction was collected in different vials for different time periods in which the first vial corresponds to 15 min (0.6 residence times) after the compound was started to be collected. Each vial was analysed by HPLC (see above). The combined vials were evaporated and purified by column chromatography allowing the isolation of **3a** (327.3 mg, 93 %).

Table S8 - Study of the reaction for the synthesis of 3-aminopyridine **3a** in its stationary state.

Time[a] / min	Tot. V / mL	Col. V.[b]	Sample V.[c] / mL	Yield	Prod. / mmol/h	Prod. / mg/h
15	1,1	0,6	1,1	36	0,086	25,1
28	2,1	1,2	1	84	0,202	58,6
42	3,1	1,7	1	97	0,233	67,7
56	4,1	2,2	1	98	0,234	67,9
70	5,2	2,9	1,1	95	0,228	66,2
84	6,2	3,4	1	96	0,230	66,8
100	7,3	4,0	1,1	97	0,232	67,3
134	9,7	5,3	2,4	96	0,230	66,9
181	12,8	7,0	3,1	94	0,226	65,8
212	15,3	8,4	2,5	104	0,249	72,4
241	17,4	9,5	2,1	95	0,228	66,2
281	20,4	11,2	3	100	0,241	69,9
319	23,3	12,8	2,9	95	0,227	65,9

a) Time of collection since the compound started to be collected.

b) Column volumes collected since the compound started to be collected.

c) Volume of the aliquot collected in the specific time point.

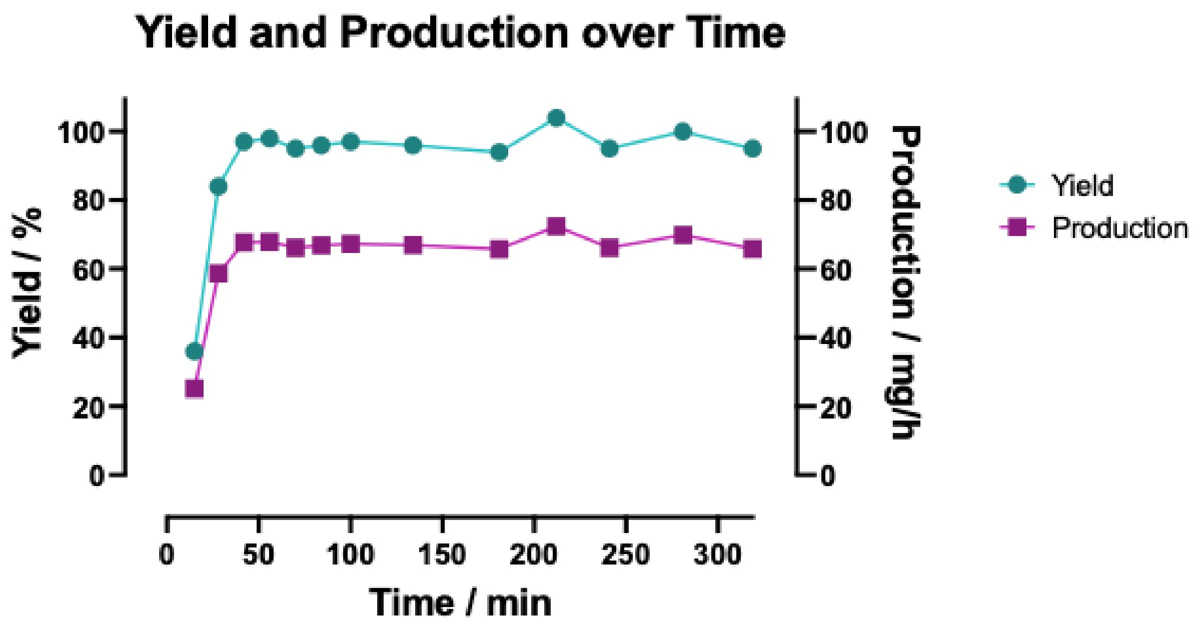

Figure S24 – Graphical representation of the yield and production of the synthesis of 3-aminopyridine **3a** under flow over time.

6. X-ray Diffraction Study

Single-crystals of compounds **3a**, **3b**, **3c**, **3d**, **3e** (two different vials giving two polymorphs), **3h**, **3i**, **3x**, **5**, **10**, **12**, **13**, **16**, **17**, **18** and **S3** were manually selected from the crystallization vial. A suitable single-crystal was mounted on a glass fiber with the help of silicon grease.

Data were collected at 293(2) K or 150 K on a RIGAKU XtaLAB Synergy-i instrument equipped with a Mo Kα (λ = 0.71073 Å) PhotonJet-i micro source and a HyPix3000 detector. The data was controlled by the CrysAlisPro software (Y. Oxford Diffraction Ltd, England, 2022, CrysAlis PRO, Rigaku V1.171.142.173a) and an Oxford Cryosystems Series 800 cryostream was used. The diffraction images were processed using CrysAlisPro software (Y. Oxford Diffraction Ltd, England, 2022, CrysAlis PRO, Rigaku V1.171.142.173a.). The data was corrected for absorption using the multi-scan absorption correction with spherical harmonics implemented in the SCALE3 ABSPACK scaling algorithm. The structures were solved using direct methods with SHELXT 2018/3 and refined using the weighted full-matrix least-squares method on F^2 with SHELXL2018/3. Anisotropic thermal parameters were used to refine all non-hydrogen atoms. Molecular diagrams were created using Mercury software.

Hydrogen atoms bound to carbon were located at their idealized positions using appropriate *HFIX* instructions in SHELXL (*43* for the aromatic and vinylic, *23* for the –CH_2– moieties and *13* for the chiral tertiary carbon atoms) and included in subsequent refinement cycles in riding-motion approximation with isotropic thermal displacements parameters (U_{iso}) fixed at 1.2 times U_{eq} of the atom to which they are attached.

Crystallographic data for the structures reported in this article have been deposited with the Cambridge Crystallographic Data Centre as supplementary publication No. CCDC 2467648-2467666. Copies of the data can be obtained free of charge on application to CCDC, 12 Union Road, Cambridge CB2 2EZ, U.K. Fax: (+44) 1223 336033. E-mail: deposit@ccdc.cam.ac.uk.

6.1. Crystal Data and Structure Refinement

(5-amino-6-phenylpyridin-3-yl)(2-hydroxyphenyl)methanone (3a)

Crystal of compound **3a** suitable for X-ray diffraction was obtained by slow evaporation of a chloroform solution at room temperature.

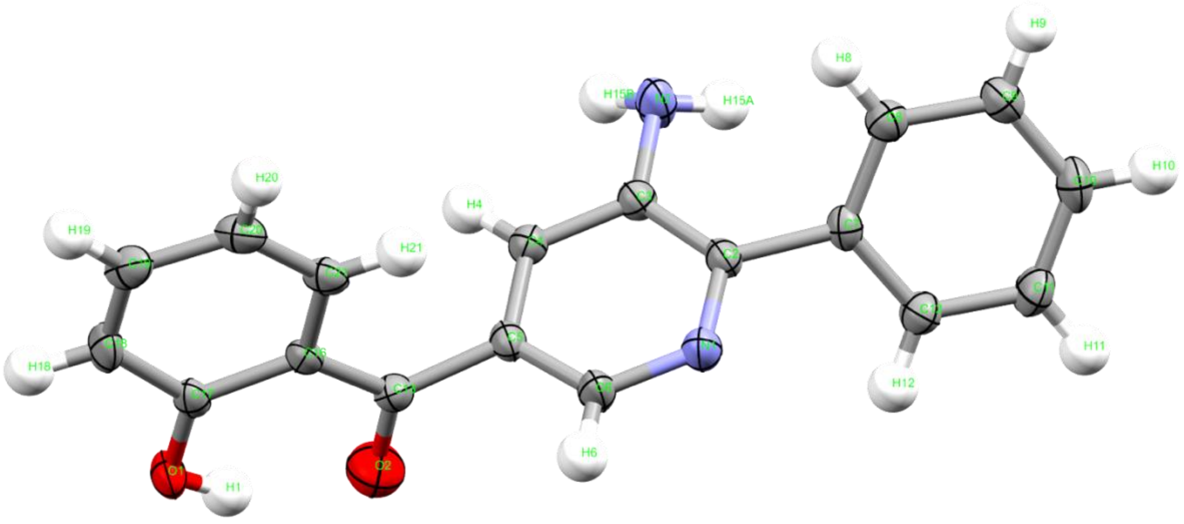

Figure S25 –Single-crystal X-ray diffraction of 3-aminopyridine **3a**. Thermal ellipsoids are shown at the 50% probability level; hydrogen atoms are shown with an arbitrary radius (0.30 Å). C, gray; H, white; O, red; N, blue.

Table S9 – Crystal data and structure refinement details of compound **3a**.

Crystal Data	
CCDC Identifier	2467648
Chemical formula	$C_{18}H_{14}N_2O_2$
M_r	290.31
Crystal system, space group	Orthorhombic, $P2_12_12_1$
Temperature (K)	150 (2)
a, *b*, *c* (Å)	8.7172 (9), 10.253 (2), 15.7717 (16)
β (°)	90
V ($Å^3$)	1409.6 (4)
Z	4
Radiation type	Mo Ka
m (mm^{-1})	0.09
Crystal size (mm)	1.00 × 0.90 × 0.80
**Data collection**	
Diffractometer	Bruker APEX-II CCD
Absorption correction	Multi-scan
T_{min}, T_{max}	0.354, 1.00
No. of measured, independent and observed [I > 2s(I)] reflections	11714, 3104, 2235
R_{int}	0.067
**Refinement**	
$R[F^2 > 2s(F^2)]$, $wR(F^2)$, S	0.067, 0.146, 1.03
No. of reflections	3104
No. of parameters	202
No. of restraints	0
H-atom treatment	H-atom parameters constrained
$D\rho_{max}$, $D\rho_{min}$ (e $Å^{-3}$)	0.39, -0.44
Absolute structure	Flack x determined using 621 quotients [(I+)-(I-)]/[(I+)+(I-)] (Parsons, Flack and Wagner, Acta Cryst. B69 (2013) 249-259).
Absolute structure parameter	-0.2 (10)

(5-amino-6-phenylpyridin-3-yl)(5-bromo-2-hydroxyphenyl)methanone (3b)

Crystal of compound **3b** suitable for X-ray diffraction was obtained by slow evaporation of a chloroform solution at room temperature.

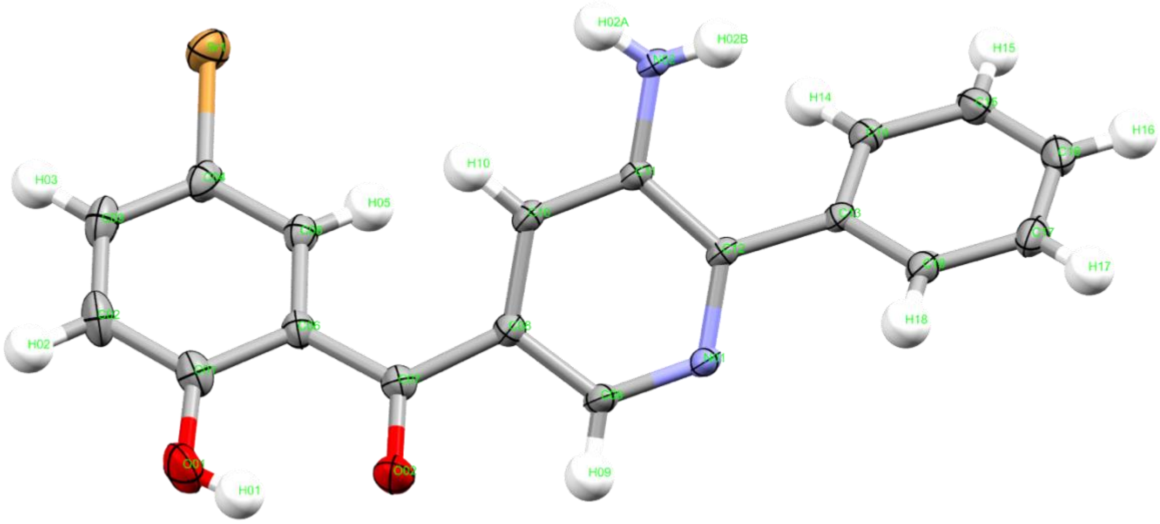

Figure S26 –Single-crystal X-ray diffraction of 3-aminopyridine **3b**. Thermal ellipsoids are shown at the 50% probability level; hydrogen atoms are shown with an arbitrary radius (0.30 Å). C, gray; H, white; O, red; N, blue; Br, orange.

Table S10 – Crystal data and structure refinement details of compound **3b**.

Crystal Data	
CCDC Identifier	2467663
Chemical formula	$C_{18}H_{13}BrN_2O_2$
M_r	369.22
Crystal system, space group	Monoclinic, $P2_1/c$
Temperature (K)	150 (2)
a, *b*, *c* (Å)	6.2994 (4), 13.7551 (10), 17.5847 (13)
β (°)	90.683 (3)
V (Å^3)	1523.59 (19)
Z	4
Radiation type	Mo Ka
m (mm^{-1})	2.71
Crystal size (mm)	0.40 × 0.08 × 0.05
**Data collection**	
Diffractometer	Bruker APEX-II CCD
Absorption correction	Multi-scan
T_{min}, T_{max}	0.410, 0.876
No. of measured, independent and observed [$I > 2s(I)$] reflections	39836, 4588, 3915
R_{int}	0.044
**Refinement**	
$R[F^2 > 2s(F^2)]$, $wR(F^2)$, S	0.029, 0.070, 1.04
No. of reflections	4588
No. of parameters	218
No. of restraints	0
H-atom treatment	H-atom parameters mixed
$D\rho_{max}$, $D\rho_{min}$ (e Å^{-3})	0.48, -0.52

(5-amino-6-phenylpyridin-3-yl)(5-chloro-2-hydroxyphenyl)methanone (3c)

Crystal of compound **3c** suitable for X-ray diffraction was obtained by slow evaporation of a dichloromethane/hexane solution at room temperature.

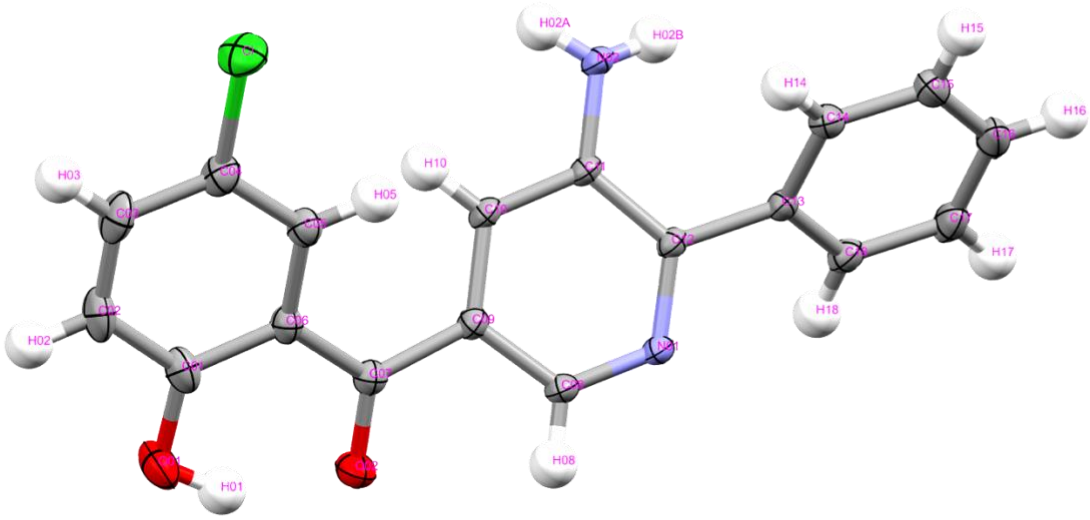

Figure S27 –Single-crystal X-ray diffraction of 3-aminopyridine **3c**. Thermal ellipsoids are shown at the 50% probability level; hydrogen atoms are shown with an arbitrary radius (0.30 Å). C, gray; H, white; O, red; N, blue; Cl, green.

Table S11 – Crystal data and structure refinement details of compound **3c**.

Crystal Data	
CCDC Identifier	2467665
Chemical formula	$C_{18}H_{13}ClN_2O_2$
M_r	324.77
Crystal system, space group	Monoclinic, $P2_1/c$
Temperature (K)	150 (2)
a, *b*, *c* (Å)	6.3010 (2), 13.6603 (4), 17.4479 (5)
β (°)	90.1690 (10)
V (Å^3)	1501.80 (8)
Z	4
Radiation type	Mo Ka
m (mm^{-1})	0.27
Crystal size (mm)	0.40 × 0.30 × 0.20
**Data collection**	
Diffractometer	Bruker APEX-II CCD
Absorption correction	Multi-scan
T_{min}, T_{max}	0.901, 0.949
No. of measured, independent and observed [$I >$ 2s(I)] reflections	39405, 4483, 3956
R_{int}	0.052
**Refinement**	
$R[F^2 > 2s(F^2)]$, wR(F^2), S	0.041, 0.113, 1.06
No. of reflections	4483
No. of parameters	220
No. of restraints	0
H-atom treatment	H-atom parameters mixed
Dρ_{max}, Dρ_{min} (e Å^{-3})	0.42, -0.30

(5-amino-6-phenylpyridin-3-yl)(5-fluoro-2-hydroxyphenyl)methanone (3d)

Crystal of compound **3d** suitable for X-ray diffraction was obtained by slow evaporation of a dichloromethane/hexane solution at room temperature.

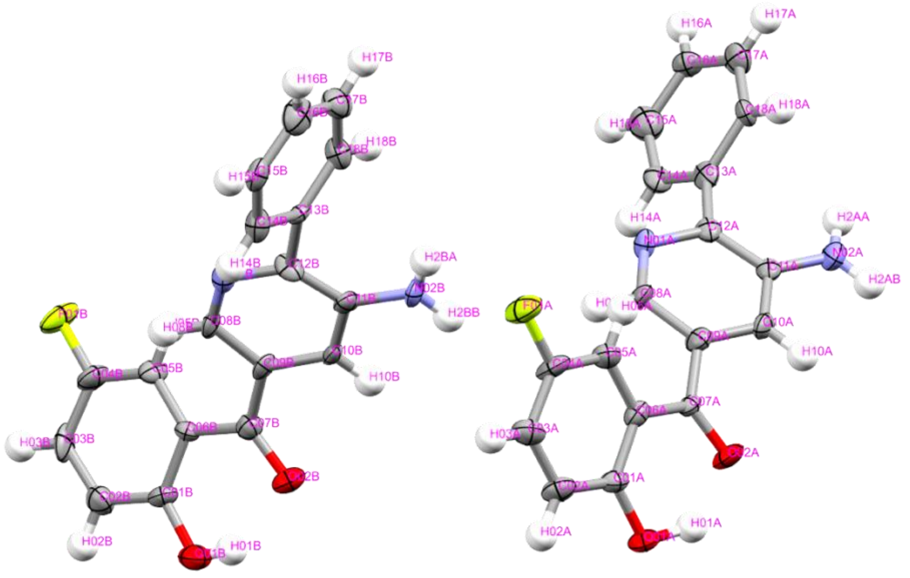

Figure S28 –Single-crystal X-ray diffraction of 3-aminopyridine **3d**. Thermal ellipsoids are shown at the 50% probability level; hydrogen atoms are shown with an arbitrary radius (0.30 Å). C, gray; H, white; O, red; N, blue; F, lime.

Table S12 – Crystal data and structure refinement details of compound **3d**.

Crystal Data	
CCDC Identifier	2467654
Chemical formula	2 $C_{18}H_{13}FN_2O_2$
M_r	616.61
Crystal system, space group	Monoclinic, C2
Temperature (K)	150 (2)
a, *b*, *c* (Å)	10.2115 (14), 6.7152 (9), 42.388 (6)
β (°)	96.120 (3)
V (Å^3)	2890.1 (7)
Z	4
Radiation type	Mo Ka
m (mm^{-1})	0.10
Crystal size (mm)	0.30 × 0.20 × 0.10
**Data collection**	
Diffractometer	Bruker APEX-II CCD
Absorption correction	Multi-scan
T_{min}, T_{max}	0.970, 0.990
No. of measured, independent and observed [$I >$ 2s(I)] reflections	19898, 5727, 5578
R_{int}	0.056
**Refinement**	
$R[F^2 > 2s(F^2)]$, wR(F^2), S	0.131, 0.343, 1.08
No. of reflections	5727
No. of parameters	418
No. of restraints	1
H-atom treatment	H-atom parameters mixed
Dρ_{max}, Dρ_{min} (e Å^{-3})	0.75, -0.57
Absolute structure	Flack x determined using 621 quotients [(I+)-(I-)]/[(I+)+(I-)] (Parsons, Flack and Wagner, Acta Cryst. B69 (2013) 249-259).
Absolute structure parameter	0.3 (5)

(5-amino-6-phenylpyridin-3-yl)(2-hydroxy-5-methylphenyl)methanone (3e)

Crystal of compound **3e** suitable for X-ray diffraction was obtained by slow evaporation of a chloroform solution at room temperature.

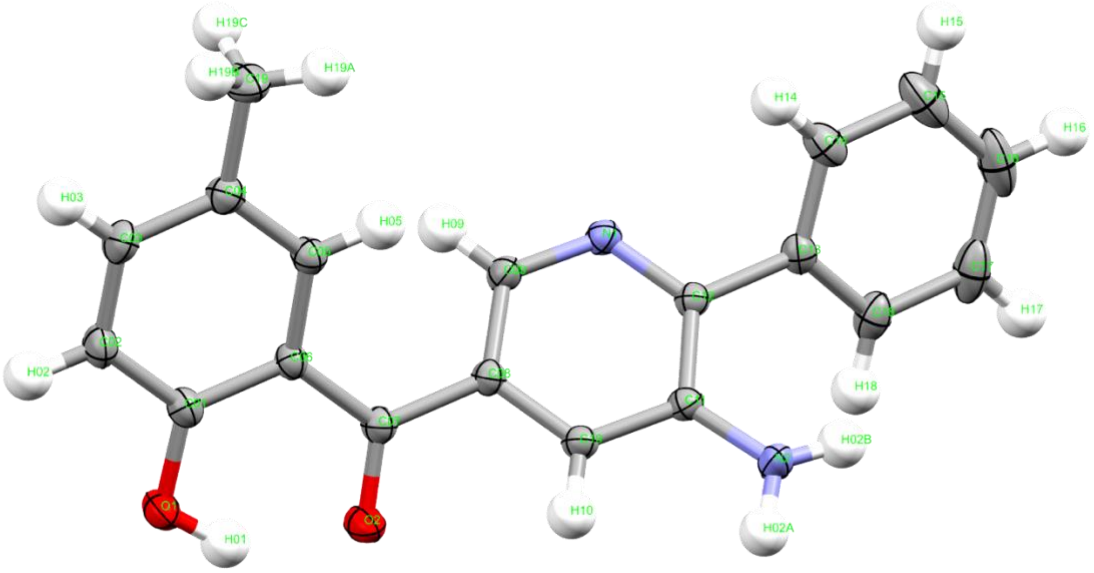

Figure S29 –Single-crystal X-ray diffraction of 3-aminopyridine **3e**. Thermal ellipsoids are shown at the 50% probability level; hydrogen atoms are shown with an arbitrary radius (0.30 Å). C, gray; H, white; O, red; N, blue.

Table S13 – Crystal data and structure refinement details of compound **3e**.

Crystal Data	
CCDC Identifier	2467666
Chemical formula	$C_{19}H_{16}N_2O_2$
M_r	304.35
Crystal system, space group	Monoclinic, $P2_1/c$
Temperature (K)	150 (2)
a, *b*, *c* (Å)	15.6105 (11), 8.6447 (6), 12.1658(9)
β (°)	108.285 (3)
V (Å^3)	1558.86 (19)
Z	4
Radiation type	Mo Ka
m (mm^{-1})	0.09
Crystal size (mm)	0.50 × 0.30 × 0.10
**Data collection**	
Diffractometer	Bruker APEX-II CCD
Absorption correction	Multi-scan
T_{min}, T_{max}	0.959, 0.992
No. of measured, independent and observed [$I > 2s(I)$] reflections	37483, 4672, 3913
R_{int}	0.047
**Refinement**	
$R[F^2 > 2s(F^2)]$, $wR(F^2)$, S	0.047, 0.124, 1.01
No. of reflections	4672
No. of parameters	219
No. of restraints	0
H-atom treatment	H-atom parameters mixed
$D\rho_{max}$, $D\rho_{min}$ (e Å^{-3})	0.34, -0.23

(5-amino-6-phenylpyridin-3-yl)(2-hydroxy-6-methoxyphenyl)methanone (3h)

Crystal of compound **3h** suitable for X-ray diffraction was obtained by slow evaporation of a chloroform solution at room temperature.

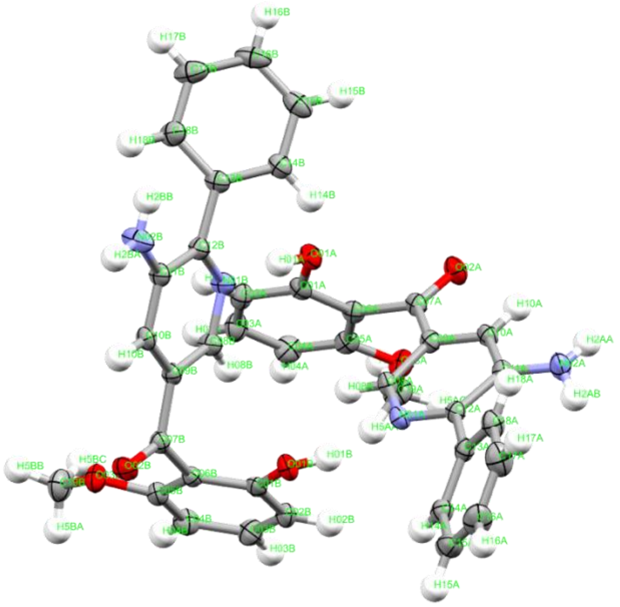

Figure S30 –Single-crystal X-ray diffraction of 3-aminopyridine **3h**. Thermal ellipsoids are shown at the 50% probability level; hydrogen atoms are shown with an arbitrary radius (0.30 Å). C, gray; H, white; O, red; N, blue.

Table S14 – Crystal data and structure refinement details of compound **3h**.

Crystal Data	
CCDC Identifier	2467664
Chemical formula	2 $C_{19}H_{16}N_2O_3$
M_r	640.67
Crystal system, space group	Triclinic, $P\bar{1}$
Temperature (K)	150 (2)
a, *b*, *c* (Å)	11.6942 (10), 11.7776 (10), 11.8613 (10)
α (°)	101.911 (3)
β (°)	90.059 (3)
γ (°)	101.154 (3)
V (Å^3)	1566. 9 (2)
Z	4
Radiation type	Mo Ka
m (mm^{-1})	0.09
Crystal size (mm)	0.30 × 0.20 × 0.10
**Data collection**	
Diffractometer	Bruker APEX-II CCD
Absorption correction	Multi-scan
T_{min}, T_{max}	0.973, 0.991
No. of measured, independent and observed [$I >$ 2s(I)] reflections	43384, 6372, 5102
R_{int}	0.069
**Refinement**	
$R[F^2 > 2s(F^2)]$, $wR(F^2)$, S	0.057, 0.165, 1.10
No. of reflections	6372
No. of parameters	449
No. of restraints	0
H-atom treatment	H-atom parameters mixed
Dρ_{max}, Dρ_{min} (e Å^{-3})	0.38, -0.37

(5-amino-6-phenylpyridin-3-yl)(2-hydroxy-4-methoxyphenyl)methanone (3i)

Crystal of compound **3i** suitable for X-ray diffraction was obtained by slow evaporation of a chloroform solution at room temperature.

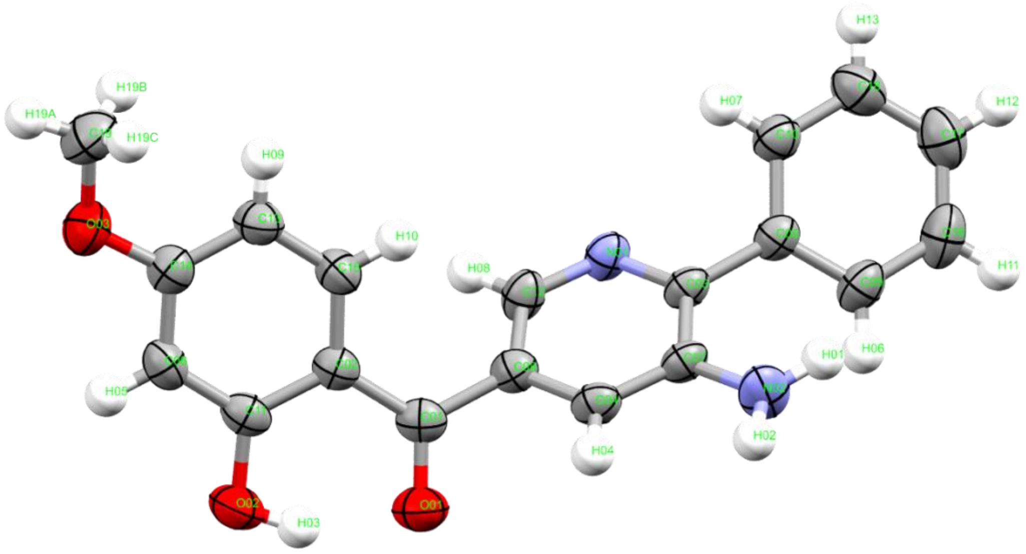

Figure S31 –Single-crystal X-ray diffraction of 3-aminopyridine **3i**. Thermal ellipsoids are shown at the 50% probability level; hydrogen atoms are shown with an arbitrary radius (0.30 Å). C, gray; H, white; O, red; N, blue.

Table S15 – Crystal data and structure refinement details of compound **3i**.

Crystal Data	
CCDC Identifier	2467652
Chemical formula	$C_{19}H_{16}N_2O3$
M_r	320.35
Crystal system, space group	Monoclinic, $P2_1/n$
Temperature (K)	293 (2)
a, *b*, *c* (Å)	7.0106 (4), 21.1175 (13), 10.6126 (12)
β (°)	90.108 (7)
V (Å^3)	1571.2 (2)
Z	4
Radiation type	Mo Ka
m (mm^{-1})	0.09
Crystal size (mm)	0.90 × 0.30 × 0.20
**Data collection**	
Diffractometer	XtaLAB Synergy, Single source at home/near, HyPix3000
Absorption correction	Multi-scan. CrysAlisPro 1.171.43.143a (Rigaku Oxford Diffraction, 2024). Empirical absorption correction using spherical harmonics, implemented in SCALE3 ABSPACK scaling algorithm.
T_{min}, T_{max}	0.494, 1.00
No. of measured, independent and observed [$I > 2s(I)$] reflections	17047, 4261, 2607
R_{int}	0.033
**Refinement**	
$R[F^2 > 2s(F^2)]$, $wR(F^2)$, S	0.056, 0.152, 1.09
No. of reflections	4261
No. of parameters	230
No. of restraints	0
H-atom treatment	H-atom parameters mixed
$D\rho_{max}$, $D\rho_{min}$ (e Å^{-3})	0.17, -0.22

[5-amino-6-(thiophen-2-yl)pyridin-3-yl](2-hydroxyphenyl)methanone (3x)

Crystal of compound **3x** suitable for X-ray diffraction was obtained by slow evaporation of a chloroform solution at room temperature.

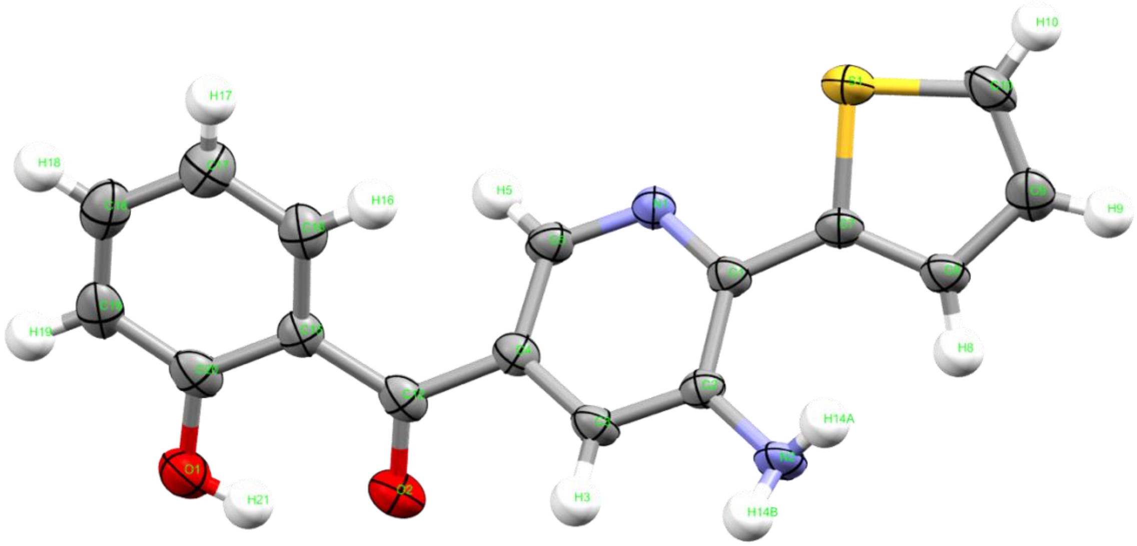

Figure S32 –Single-crystal X-ray diffraction of 3-aminopyridine **3x**. Thermal ellipsoids are shown at the 50% probability level; hydrogen atoms are shown with an arbitrary radius (0.30 Å). C, gray; H, white; O, red; N, blue; S, yellow.

Table S16 – Crystal data and structure refinement details of compound **3x**.

Crystal Data	
CCDC Identifier	2467649
Chemical formula	$C_{16}H_{12}N_2O_2S$
M_r	296.34
Crystal system, space group	Monoclinic, $P2_1/n$
Temperature (K)	150 (2)
a, *b*, *c* (Å)	7.4508 (7), 17.753 (4), 10.3529 (10)
β (°)	90.656 (11)
V (Å^3)	1369.4 (3)
Z	4
Radiation type	Mo Ka
m (mm^{-1})	0.24
Crystal size (mm)	0.80 × 0.60 × 0.20
**Data collection**	
Diffractometer	Bruker APEX-II CCD
Absorption correction	Multi-scan
T_{min}, T_{max}	0.557, 1.00
No. of measured, independent and observed [$I >$ 2s(I)] reflections	10075, 2788, 1763
R_{int}	0.075
**Refinement**	
$R[F^2 > 2s(F^2)]$, wR(F^2), S	0.063, 0.132, 1.04
No. of reflections	2788
No. of parameters	193
No. of restraints	0
H-atom treatment	H-atom parameters constrained
Dρ_{max}, Dρ_{min} (e Å^{-3})	0.33, -0.38

[10-(2-hydroxybenzyl)pyrido[2,3-*b*]indolizin-3-yl](2-hydroxyphenyl)methanone (5)

Crystal of compound **5** suitable for X-ray diffraction was obtained by slow evaporation of a chloroform solution at room temperature.

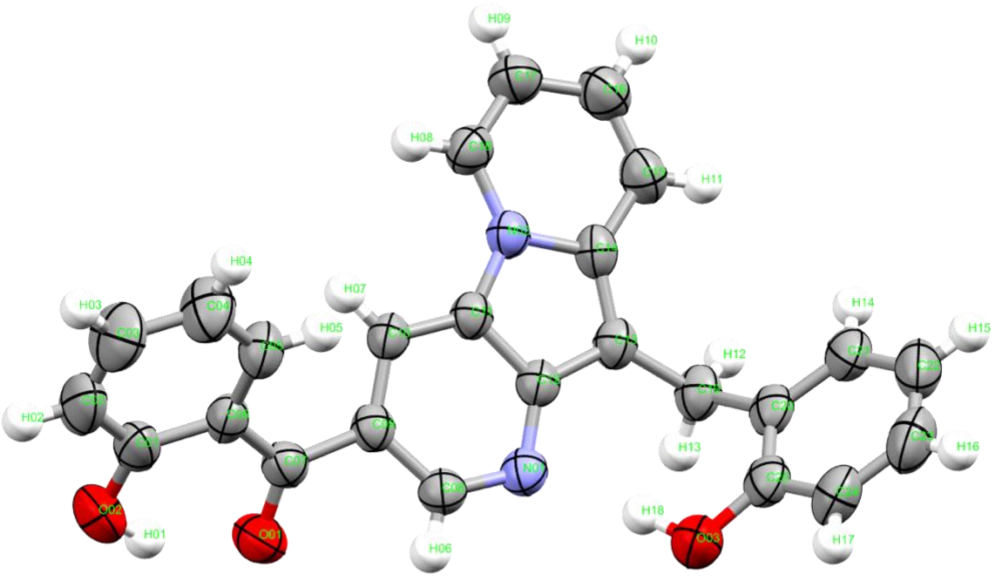

Figure S33 –Single-crystal X-ray diffraction of 3-aminopyridine **5**. Thermal ellipsoids are shown at the 50% probability level; hydrogen atoms are shown with an arbitrary radius (0.30 Å). C, gray; H, white; O, red; N, blue; S, yellow.

Table S17 – Crystal data and structure refinement details of compound **5**.

Crystal Data	
CCDC Identifier	2467651
Chemical formula	$C_{25}H_{18}N_2O_3$
M_r	394.43
Crystal system, space group	Orthorhombic, $Pca2_1$
Temperature (K)	293 (2)
a, *b*, *c* (Å)	25.1674 (18), 10.5427 (9), 7.3630 (5)
β (°)	90
V ($Å^3$)	1953.6 (3)
Z	4
Radiation type	Mo Ka
m (mm^{-1})	0.09
Crystal size (mm)	0.60 × 0.10 × 0.08
**Data collection**	
Diffractometer	Bruker APEX-II CCD
Absorption correction	Multi-scan
T_{min}, T_{max}	0.654, 0.745
No. of measured, independent and observed [$I >$ 2s(I)] reflections	15420, 3817, 2876
R_{int}	0.044
**Refinement**	
$R[F^2 > 2s(F^2)]$, $wR(F^2)$, S	0.036, 0.082, 1.09
No. of reflections	3817
No. of parameters	275
No. of restraints	1
H-atom treatment	H-atom parameters mixed
$D\rho_{max}$, $D\rho_{min}$ (e $Å^{-3}$)	0.11, -0.13
Absolute structure	Flack x determined using 621 quotients [(I+)-(I-)]/[(I+)+(I-)] (Parsons, Flack and Wagner, Acta Cryst. B69 (2013) 249-259).
Absolute structure parameter	-0.6 (8)

N-[5-(2-hydroxybenzoyl)-2-phenylpyridin-3-yl]formamide (9)

Crystal of compound **9** suitable for X-ray diffraction was obtained by slow evaporation of a chloroform solution at room temperature.

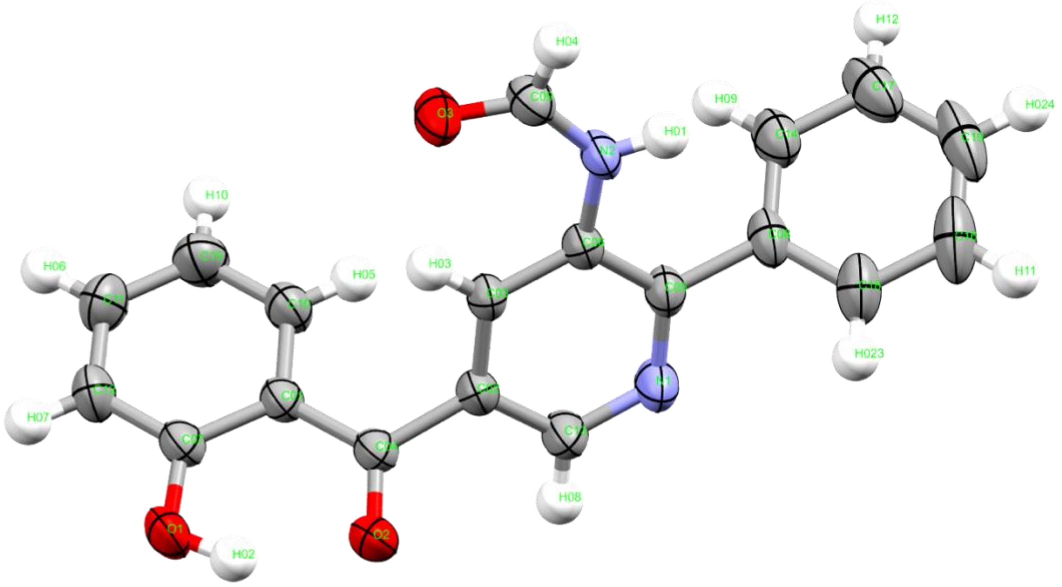

Figure S34 –Single-crystal X-ray diffraction of pyridinyl formamide **9**. Thermal ellipsoids are shown at the 50% probability level; hydrogen atoms are shown with an arbitrary radius (0.30 Å). C, gray; H, white; O, red; N, blue.

Table S18 – Crystal data and structure refinement details of compound **9**.

**Crystal Data**	
CCDC Identifier	2467658
Chemical formula	$C_{19}H_{14}N_2O_3$
M_r	318.33
Crystal system, space group	Triclinic, $P\bar{1}$
Temperature (K)	293 (2)
a, *b*, *c* (Å)	8.5573 (5), 10.1838 (7), 10.4224 (4)
α (°)	67.655 (6)
β (°)	65.995 (6)
γ (°)	88.482 (5)
V (Å^3)	758.53 (9)
Z	2
Radiation type	Mo Ka
m (mm^{-1})	0.10
Crystal size (mm)	0.50 × 0.40 × 0.30
**Data collection**	
Diffractometer	XtaLAB Synergy, Single source at home/near, HyPix3000
Absorption correction	Multi-scan
T_{min}, T_{max}	0.468, 1.00
No. of measured, independent and observed [$I >$ 2s(I)] reflections	23227, 3282, 2672
R_{int}	0.039
**Refinement**	
$R[F^2 > 2s(F^2)]$, $wR(F^2)$, S	0.047, 0.131, 1.09
No. of reflections	3282
No. of parameters	225
No. of restraints	0
H-atom treatment	H-atom parameters mixed
$D\rho_{max}$, $D\rho_{min}$ (e Å^{-3})	0.19, -0.20

(5-fluoro-6-phenylpyridin-3-yl)(2-hydroxyphenyl)methanone (11)

Crystal of compound **11** suitable for X-ray diffraction was obtained by slow evaporation of a dichloromethane/hexane solution at room temperature.

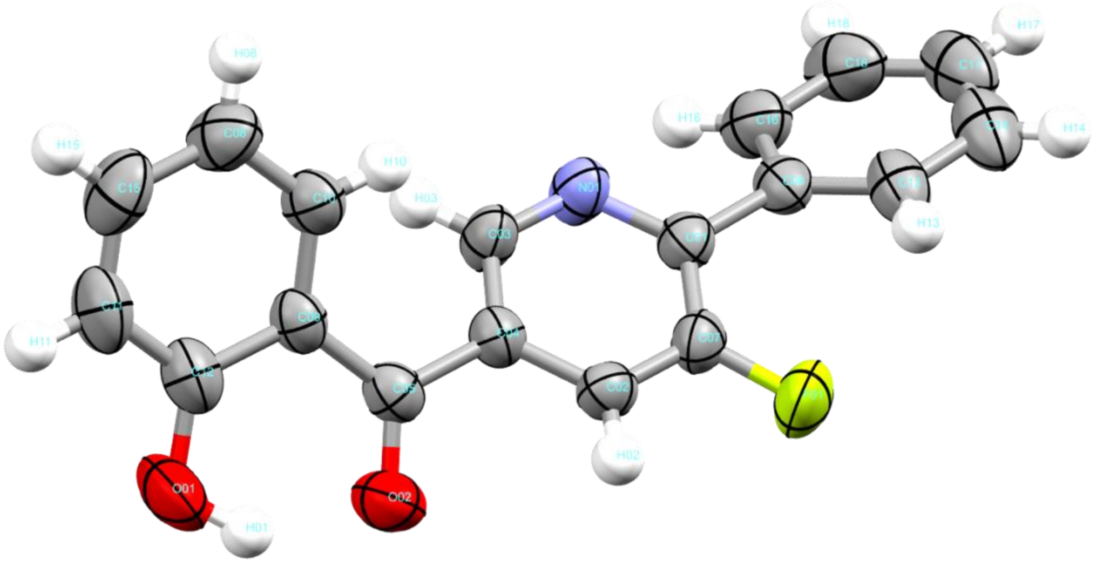

Figure S35 –Single-crystal X-ray diffraction of 3-fluoropyridine **11**. Thermal ellipsoids are shown at the 50% probability level; hydrogen atoms are shown with an arbitrary radius (0.30 Å). C, gray; H, white; O, red; N, blue; F, lime.

Table S19 – Crystal data and structure refinement details of compound **11**.

Crystal Data	
CCDC Identifier	2467649
Chemical formula	$C_{18}H_{12}FNO_2$
M_r	293.30
Crystal system, space group	Orthorhombic, $P2_12_12_1$
Temperature (K)	293 (2)
a, *b*, *c* (Å)	6.4496 (4), 10.3813 (5), 21.3381 (9)
β (°)	90
V (Å^3)	1428.70 (13)
Z	4
Radiation type	Mo Ka
m (mm^{-1})	0.10
Crystal size (mm)	0.60 × 0.50 × 0.20
**Data collection**	
Diffractometer	XtaLAB Synergy, Single source at home/near, HyPix3000
Absorption correction	Multi-scan. CrysAlisPro 1.171.43.143a (Rigaku Oxford Diffraction, 2024). Empirical absorption correction using spherical harmonics, implemented in SCALE3 ABSPACK scaling algorithm.
T_{min}, T_{max}	0.331, 1.00
No. of measured, independent and observed [$I >$ 2s(I)] reflections	13367, 3631, 2254
R_{int}	0.042
**Refinement**	
$R[F^2 > 2s(F^2)]$, $wR(F^2)$, S	0.052, 0.122, 0.996
No. of reflections	3631
No. of parameters	201
No. of restraints	0
H-atom treatment	H-atom parameters mixed
$D\rho_{max}$, $D\rho_{min}$ (e Å^{-3})	0.29, -0.33
Absolute structure	Flack x determined using 621 quotients [(I+)-(I-)]/[(I+)+(I-)] (Parsons, Flack and Wagner, Acta Cryst. B69 (2013) 249-259).
Absolute structure parameter	1.0 (7)

(5-chloro-6-phenylpyridin-3-yl)(2-hydroxyphenyl)methanone (12)

Crystal of compound **12** suitable for X-ray diffraction was obtained by slow evaporation of a dichloromethane/hexane solution at room temperature.

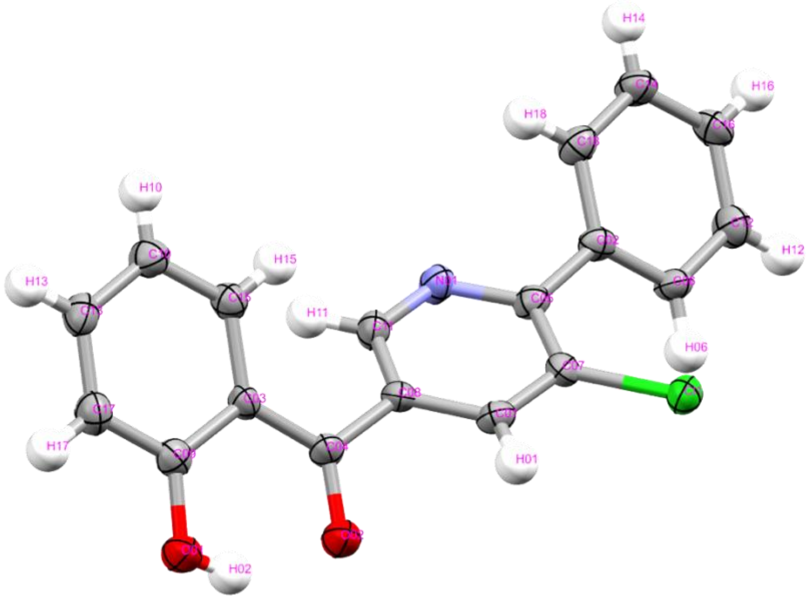

Figure S36 –Single-crystal X-ray diffraction of 3-chloropyridine **12**. Thermal ellipsoids are shown at the 50% probability level; hydrogen atoms are shown with an arbitrary radius (0.30 Å). C, gray; H, white; O, red; N, blue; Cl, green.

Table S20 – Crystal data and structure refinement details of compound **12**.

**Crystal Data**	
CCDC Identifier	2467659
Chemical formula	$C_{18}H_{12}ClNO_2$
M_r	309.75
Crystal system, space group	Monoclinic, $P2_1/n$
Temperature (K)	150 (10)
a, *b*, *c* (Å)	3.8071 (3), 28.787 (2), 12.6438 (11)
β (°)	94.706 (8)
V (Å^3)	1381.01 (19)
Z	4
Radiation type	Mo Ka
m (mm^{-1})	0.28
Crystal size (mm)	0.50 × 0.10 × 0.05
**Data collection**	
Diffractometer	XtaLAB Synergy, Single source at home/near, HyPix3000
Absorption correction	Multi-scan. CrysAlisPro 1.171.43.143a (Rigaku Oxford Diffraction, 2024). Empirical absorption correction using spherical harmonics, implemented in SCALE3 ABSPACK scaling algorithm.
T_{min}, T_{max}	0.673, 1.00
No. of measured, independent and observed [$I > 2s(I)$] reflections	24103, 3533, 1483
R_{int}	0.165
**Refinement**	
$R[F^2 > 2s(F^2)]$, $wR(F^2)$, S	0.080, 0.173, 0.936
No. of reflections	3533
No. of parameters	203
No. of restraints	0
H-atom treatment	H-atom parameters mixed
$D\rho_{max}$, $D\rho_{min}$ (e Å^{-3})	0.48, -0.41

(5-azido-6-phenylpyridin-3-yl)(2-hydroxyphenyl)methanone (15)

Crystal of compound **15** suitable for X-ray diffraction was obtained by slow evaporation of a dichloromethane/hexane solution at room temperature.

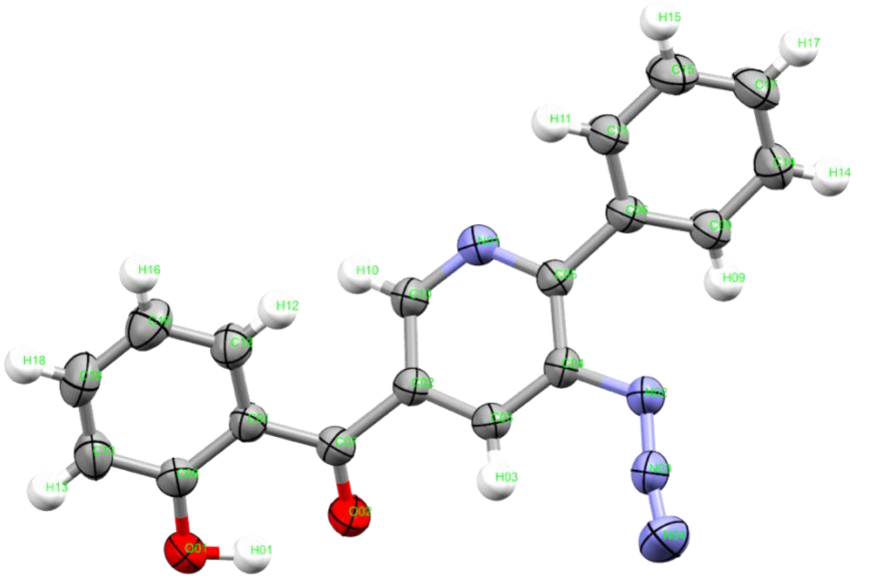

Figure S37 –Single-crystal X-ray diffraction of 3-azidopyridine **15**. Thermal ellipsoids are shown at the 50% probability level; hydrogen atoms are shown with an arbitrary radius (0.30 Å). C, gray; H, white; O, red; N, blue.

Table S21 – Crystal data and structure refinement details of compound **15**.

Crystal Data	
CCDC Identifier	2467657
Chemical formula	$C_{18}H_{12}N_4O_2$
M_r	316.32
Crystal system, space group	Monoclinic, $P2_1/n$
Temperature (K)	293 (2)
a, *b*, *c* (Å)	3.8542 (2), 31.3866 (12), 12.3297 (7)
β (°)	94.366 (4)
V (Å^3)	1487.20 (13)
Z	4
Radiation type	Mo Ka
m (mm^{-1})	0.10
Crystal size (mm)	0.90 × 0.08 × 0.05
**Data collection**	
Diffractometer	XtaLAB Synergy, Single source at home/near, HyPix3000
Absorption correction	Multi-scan. CrysAlisPro 1.171.43.143a (Rigaku Oxford Diffraction, 2024). Empirical absorption correction using spherical harmonics, implemented in SCALE3 ABSPACK scaling algorithm.
T_{min}, T_{max}	0.160, 1.00
No. of measured, independent and observed [$I > 2s(I)$] reflections	23461, 3837, 1961
R_{int}	0.091
**Refinement**	
$R[F^2 > 2s(F^2)]$, $wR(F^2)$, S	0.083, 0.214, 1.04
No. of reflections	3837
No. of parameters	221
No. of restraints	0
H-atom treatment	H-atom parameters mixed
$D\rho_{max}$, $D\rho_{min}$ (e Å^{-3})	0.31, -0.33

(2-hydroxyphenyl)(5*H*-pyrido[3,2-b]indol-3-yl)methanone (16)

Crystal of compound **16** suitable for X-ray diffraction was obtained by slow evaporation of a dichloromethane/hexane solution at room temperature.

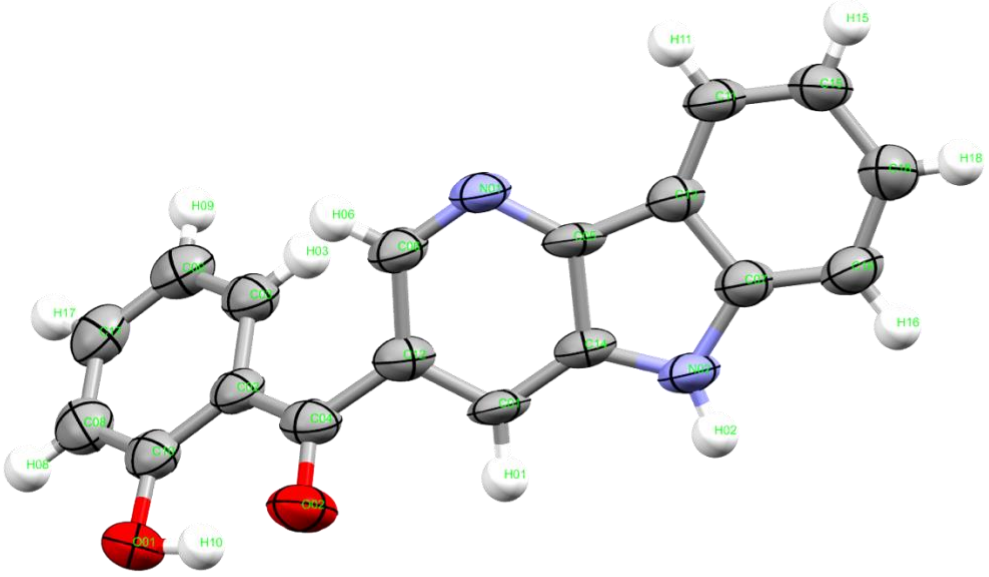

Figure S38 –Single-crystal X-ray diffraction of 4-azacarbazole **16**. Thermal ellipsoids are shown at the 50% probability level; hydrogen atoms are shown with an arbitrary radius (0.30 Å). C, gray; H, white; O, red; N, blue.

Table S22 – Crystal data and structure refinement details of compound **16**.

Crystal Data	
CCDC Identifier	2467653
Chemical formula	$C_{18}H_{12}N_2O_2$
M_r	288.31
Crystal system, space group	Monoclinic, $P2_1/c$
Temperature (K)	293 (2)
a, *b*, *c* (Å)	27.433 (3), 4.1644 (6), 11.8002 (14)
β (°)	95.558 (12)
V (Å^3)	1341.8 (3)
Z	4
Radiation type	Mo Ka
m (mm^{-1})	0.10
Crystal size (mm)	0.40 × 0.30 × 0.05
**Data collection**	
Diffractometer	XtaLAB Synergy, Single source at home/near, HyPix3000
Absorption correction	Multi-scan. CrysAlisPro 1.171.43.143a (Rigaku Oxford Diffraction, 2024). Empirical absorption correction using spherical harmonics, implemented in SCALE3 ABSPACK scaling algorithm.
T_{min}, T_{max}	0.241, 1.00
No. of measured, independent and observed [$I > 2s(I)$] reflections	14184, 2851, 1363
R_{int}	0.141
**Refinement**	
$R[F^2 > 2s(F^2)]$, $wR(F^2)$, S	0.118, 0.295, 1.03
No. of reflections	2851
No. of parameters	203
No. of restraints	0
H-atom treatment	H-atom parameters mixed
$D\rho_{max}$, $D\rho_{min}$ (e Å^{-3})	0.36, -0.41

(5-hydroxy-6-phenylpyridin-3-yl)(2-hydroxyphenyl)methanone (17)

Crystal of compound **17** suitable for X-ray diffraction was obtained by slow evaporation of a dichloromethane/hexane solution at room temperature.

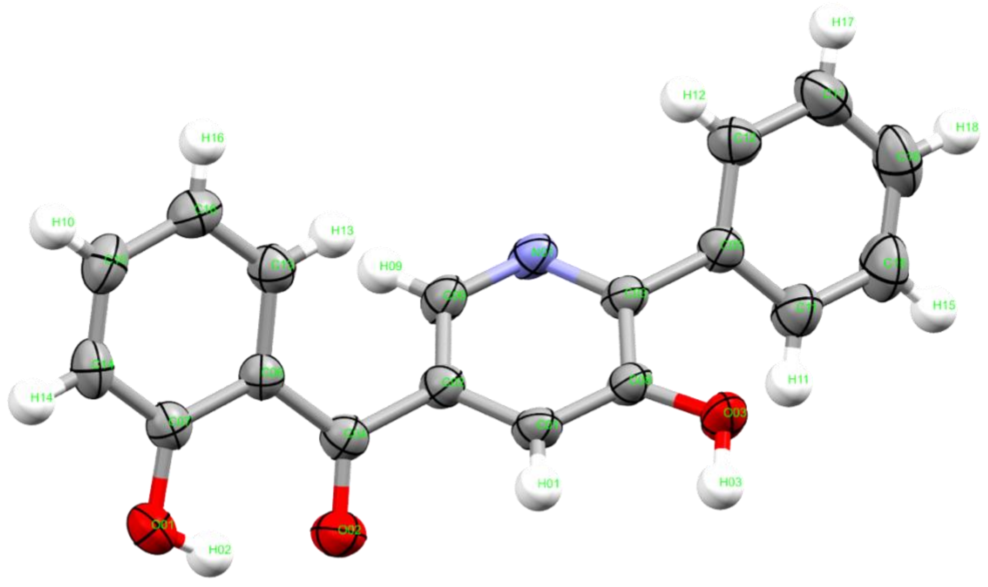

Figure S39 –Single-crystal X-ray diffraction of 3-hydroxypyrdine **17**. Thermal ellipsoids are shown at the 50% probability level; hydrogen atoms are shown with an arbitrary radius (0.30 Å). C, gray; H, white; O, red; N, blue.

Table S23 – Crystal data and structure refinement details of compound **17**.

Crystal Data	
CCDC Identifier	2467660
Chemical formula	$C_{18}H_{13}NO_3$
M_r	291.31
Crystal system, space group	Monoclinic, $P2_1/c$
Temperature (K)	293 (2)
a, *b*, *c* (Å)	28.3538 (8), 4.28290 (10), 11.5140 (3)
β (°)	99.754 (3)
V (Å^3)	1378.01 (6)
Z	4
Radiation type	Mo Ka
m (mm^{-1})	0.10
Crystal size (mm)	0.90 × 0.40 × 0.10
**Data collection**	
Diffractometer	XtaLAB Synergy, Single source at home/near, HyPix3000
Absorption correction	Multi-scan. CrysAlisPro 1.171.43.143a (Rigaku Oxford Diffraction, 2024). Empirical absorption correction using spherical harmonics, implemented in SCALE3 ABSPACK scaling algorithm.
T_{min}, T_{max}	0.538, 1.00
No. of measured, independent and observed [$I > 2s(I)$] reflections	21334, 3563, 2302
R_{int}	0.042
**Refinement**	
$R[F^2 > 2s(F^2)]$, $wR(F^2)$, S	0.049, 0.110, 1.06
No. of reflections	3563
No. of parameters	207
No. of restraints	0
H-atom treatment	H-atom parameters mixed
$D\rho_{max}$, $D\rho_{min}$ (e Å^{-3})	0.21, -0.20

N-[5-(2-hydroxybenzoyl)-2-phenylpyridin-3-yl]acetamide (S3)

Crystal of compound **S3** suitable for X-ray diffraction was obtained by slow evaporation of a chloroform solution at room temperature.

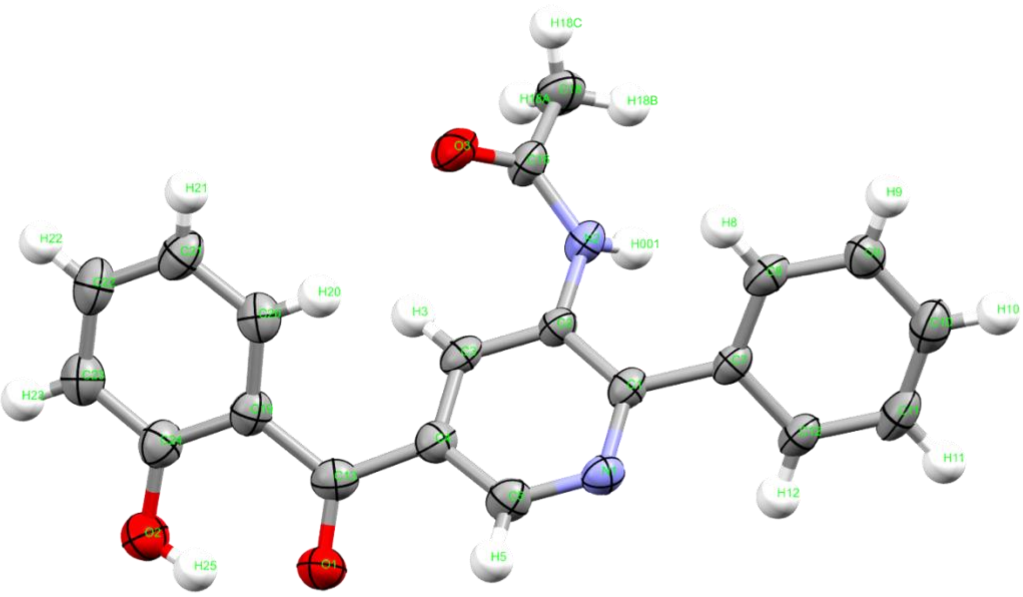

Figure S40 –Single-crystal X-ray diffraction of acetamide **S3**. Thermal ellipsoids are shown at the 50% probability level; hydrogen atoms are shown with an arbitrary radius (0.30 Å). C, gray; H, white; O, red; N, blue.

Table S24 – Crystal data and structure refinement details of compound **S3**.

Crystal Data	
CCDC Identifier	2467661
Chemical formula	$C_{20}H_{16}N_2O_3$
M_r	332.35
Crystal system, space group	Monoclinic, $P2_1/n$
Temperature (K)	150 (2)
a, *b*, *c* (Å)	13.3376 (10), 7.5899 (7), 16.2551(15)
β (°)	94.583 (10)
V (Å^3)	1640.3 (2)
Z	4
Radiation type	Mo Ka
m (mm^{-1})	0.09
Crystal size (mm)	0.60 × 0.15 × 0.10
**Data collection**	
Diffractometer	Bruker APEX-II CCD
Absorption correction	Multi-scan
T_{min}, T_{max}	0.410, 1.00
No. of measured, independent and observed [$I > 2s(I)$] reflections	22606, 3792, 1550
R_{int}	0.103
**Refinement**	
$R[F^2 > 2s(F^2)]$, $wR(F^2)$, S	0.088, 0.173, 1.19
No. of reflections	3792
No. of parameters	233
No. of restraints	0
H-atom treatment	H-atom parameters mixed
$D\rho_{max}$, $D\rho_{min}$ (e Å^{-3})	0.49, -0.37

7. Synthesis of 3-Aminopyridines

7.1. General Procedure

In a sealed 10 mL microwave reactor, the respective 3-formylchromone **1** (1 equiv., 0.46 mmol), pyridinium salt **2** (1.5 equiv., 0.69 mmol) and NH_4OAc (15 equiv., 6.89 mmol, 531.1 mg) were added into EtOH (0.46 M, 1 mL). The vessel was placed in the microwave and the program was started. The program had two steps: 2 min at 50 °C (30 W), followed by 15 min at 250 PSI (50 W) (Figure S41). When the program ended, the reaction was let to cool down to room temperature. The crude was loaded into silica and purified by column chromatography using a gradient of 0% to 40% ethyl acetate in hexane (Scheme S7).

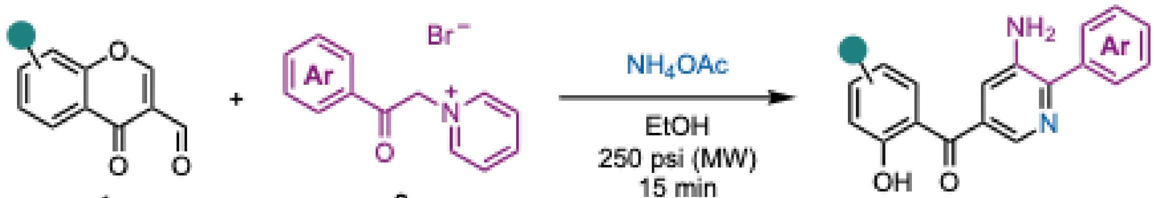

1 2 3

Scheme S7 - General procedure for the synthesis of the aromatic library of 3-aminopyridines **3**.

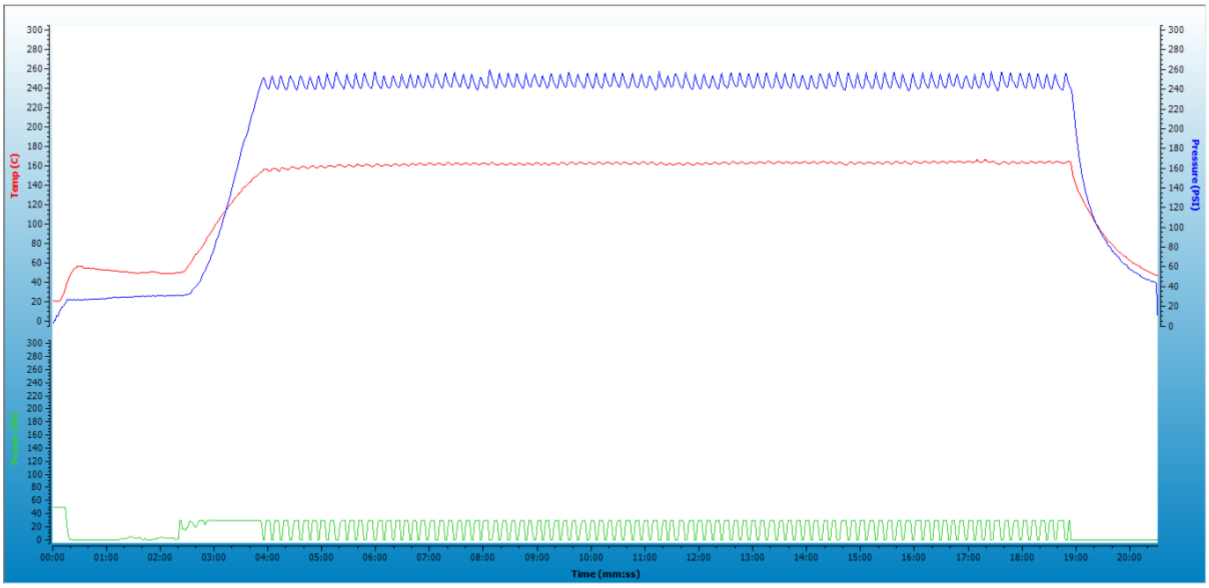

Figure S41 –Variation of temperature (°C) (red), pressure (PSI) (blue), and microwave power (W) (green) over time during a microwave-assisted synthesis of 3-aminopyridine **3a**.

7.2. Scope and Characterization

(5-amino-6-phenylpyridin-3-yl)(2-hydroxyphenyl)methanone (3a)

Following the general procedure using 4-oxo-4*H*-chromene-3-carbaldehyde **1a** (80.0 mg) and 1-(2-oxo-2-phenylethyl)pyridin-1-ium bromide **2a** (191.7 mg). After purification by column chromatography using a gradient of 0% to 40% ethyl acetate in hexane, the compound **3a** (119.9 mg, 90% yield) was obtained.

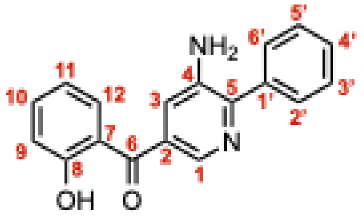

3a

Yellow solid. Melting point of 147.8–148.6 °C.

^{1}H NMR (300 MHz, CDCl$_3$): δ 11.94 (s, O*H*, 1H), 8.38 (d, *J* = 1.9 Hz, H-1, 1H), 7.78 – 7.67 (m, H-12, 2' and 6', 3H), 7.59 – 7.41 (m, H-10, 3', 4' and 5', 4H), 7.35 (d, *J* = 1.9 Hz, H-3, 1H), 7.09 (dd, *J* = 8.4, 1.2 Hz, H-9, 1H), 6.92 (ddd, *J* = 8.2, 7.2, 1.2 Hz, H-11, 1H), 4.08 (s, N*H*$_2$, 2H) ppm (Figure S42).

^{13}C NMR (75 MHz, CDCl$_3$): δ 199.6 (C-6), 163.2 (C-8), 147.9 (C-5), 140.0 (C-4), 139.8 (C- 1), 137.6 (C-1'), 136.8 (C-4'), 133.3 (C-12), 133.0 (C-2), 129.1 (C-10, 3' and 5', 3C), 128.4 (C-2' and 6'), 122.4 (C-3), 119.2 (C-7), 119.0 (C-11), 118.5 (C-9) ppm (Figure S43).

HRMS (ESI) *m/z*: [M + H]$^+$ Calcd for $C_{18}H_{15}N_2O_2^+$ 291.1128 ; Found 291.1122.

IR (neat) $_{max}$: 3463, 3289, 3147, 3060, 2925, 2851, 1978, 1960, 1920, 1906, 1859, 1828, 1787, 1726, 1619, 1584 cm^{-1}.

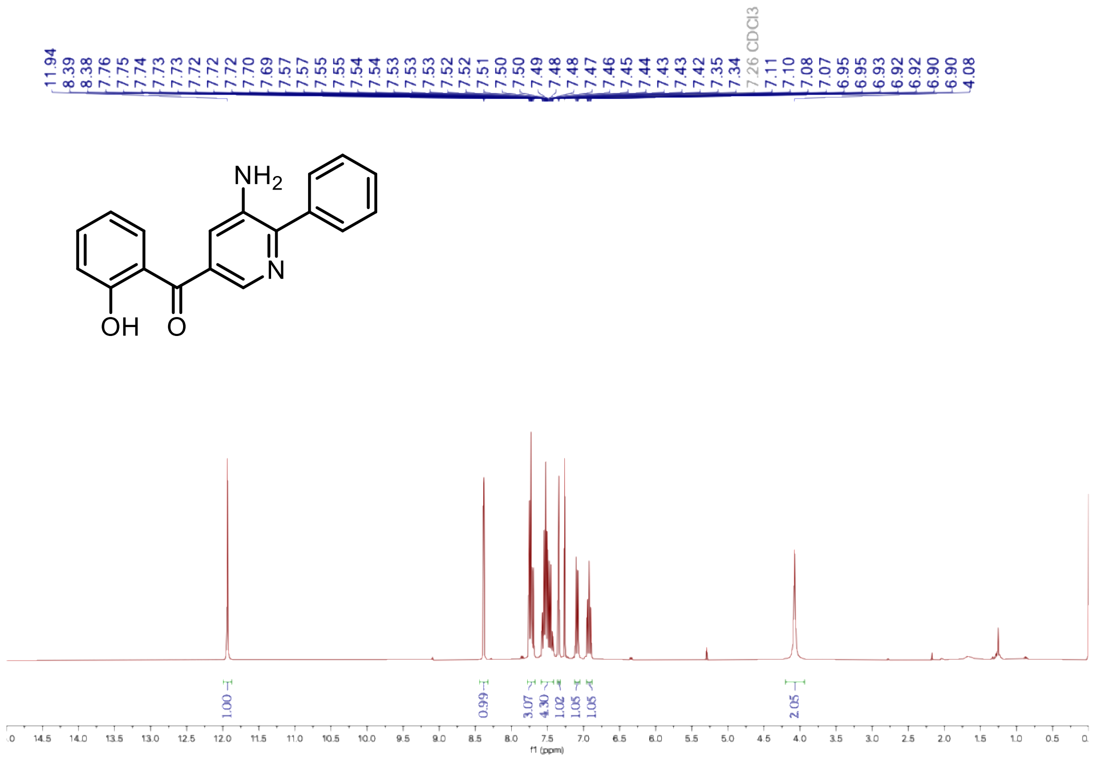

Figure S42 – ^{1}H-NMR (300 MHz, $CDCl_3$) of 3-aminopyridine **3a**.

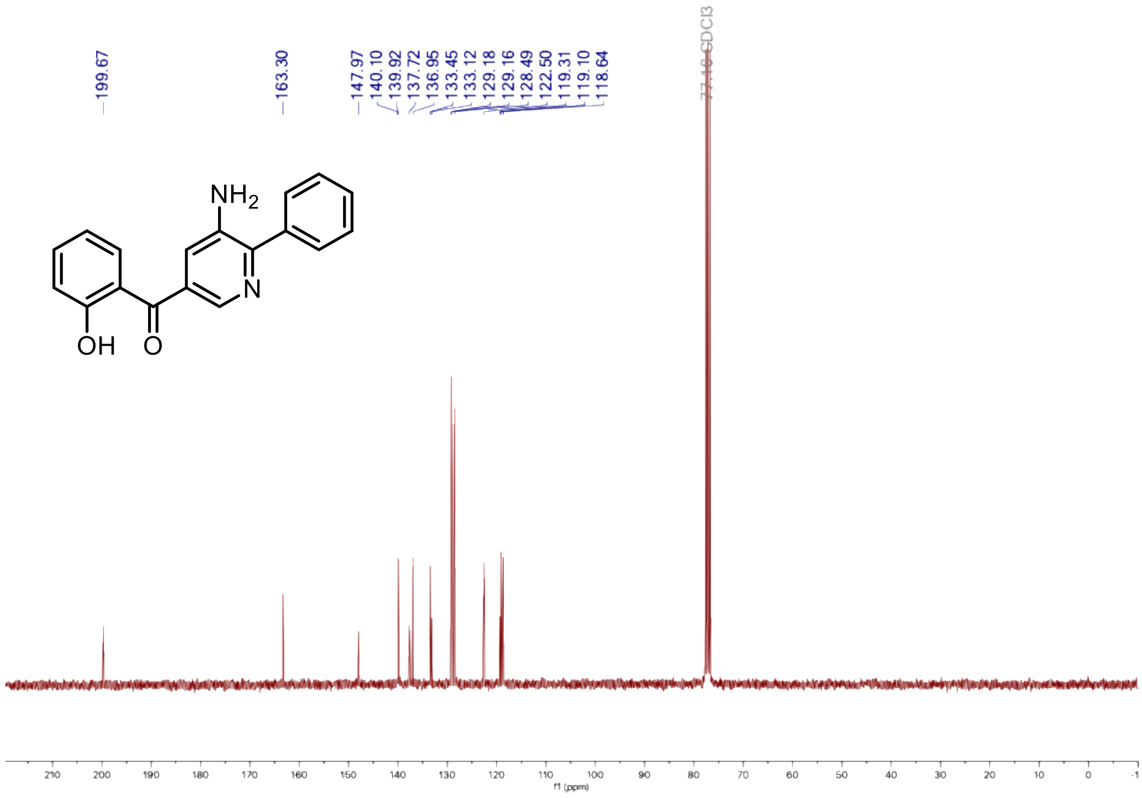

Figure S43 – ^{13}C-NMR (75 MHz, $CDCl_3$) of 3-aminopyridine **3a**.

(5-amino-6-phenylpyridin-3-yl)(5-bromo-2-hydroxyphenyl)methanone (3b)

Following the general procedure using 6-bromo-4-oxo-4*H*-chromene-3-carbaldehyde **1b** (116.2 mg) and 1-(2-oxo-2-phenylethyl)pyridin-1-ium bromide **2a** (191.7 mg). After purification by column chromatography using a gradient of 0% to 40% ethyl acetate in hexane, the compound **3b** (132.2 mg, 78% yield) was obtained.

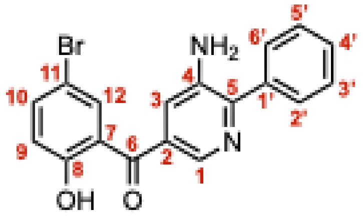

3b

Light yellow solid. Melting point of 177.0–177.8 °C.

^{1}H NMR (300 MHz, $CDCl_3$): δ 11.83 (s, O*H*, 1H), 8.36 (d, *J* = 1.9 Hz, H-1, 1H), 7.81 (d, *J* = 2.5 Hz, H-12, 1H), 7.77 – 7.71 (m, H-2' and 6', 2H), 7.60 (dd, *J* = 8.9, 2.5 Hz, H-10, 1H), 7.56 – 7.41 (m, H-3', 4' and 5', 3H), 7.31 (d, *J* = 1.9 Hz, H-3, 1H), 6.99 (d, *J* = 8.9 Hz, H-9, 1H), 4.13 (s, N*H₂*, 2H) ppm (Figure S44).

^{13}C NMR (75 MHz, $CDCl_3$): δ 198.5 (C-6), 162.1 (C-8), 148.3 (C-5), 140.2 (C-4), 139.6 (C-1), 139.5 (C-10), 137.5 (C-1'), 135.0 (C-12), 132.3 (C-2), 129.2 (C-4'), 129.1 (C-3' and 5', 2C), 128.4 (C-2' and 6', 2C), 122.1 (C-3), 120.6 (C-9), 120.4 (C-7), 110.6 (C-11) ppm (Figure S45).

HRMS (ESI) *m/z*: $[M + H]^+$ Calcd for $C_{18}H_{14}BrN_2O_2^+$ 369.0233 ; Found 369.0228.

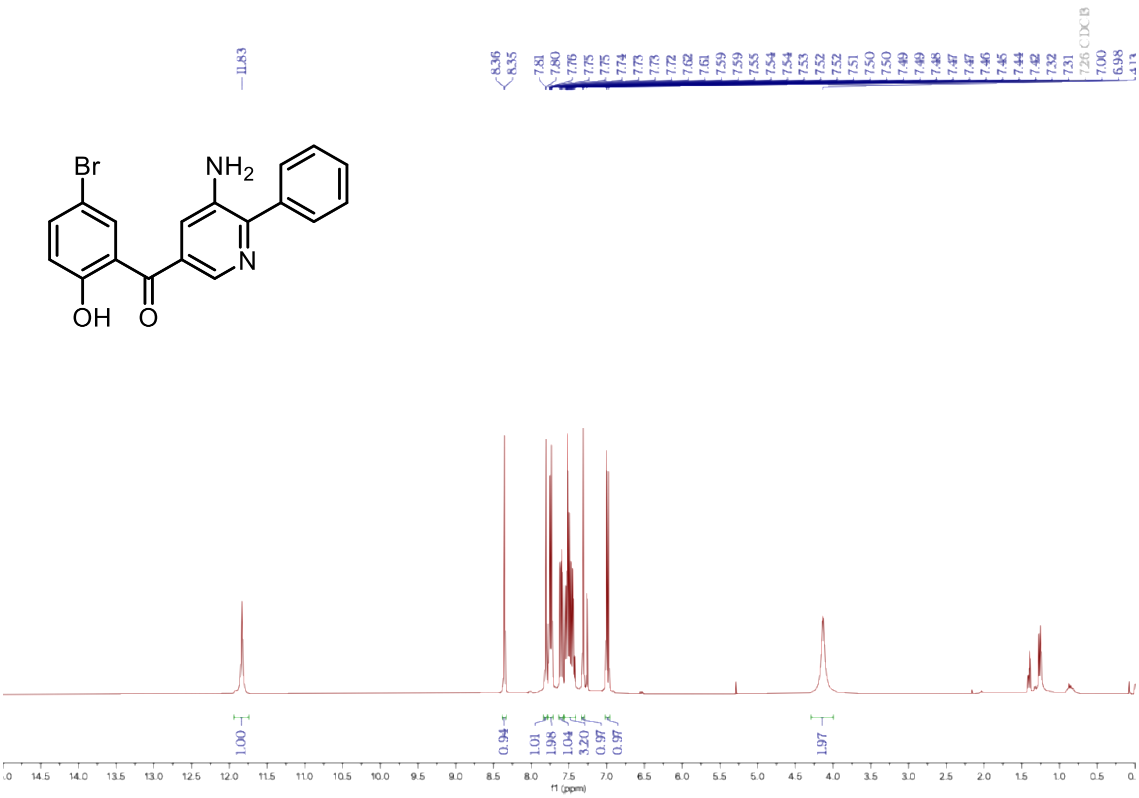

Figure S44 – ^{1}H-NMR (300 MHz, $CDCl_3$) of 3-aminopyridine **3b**.

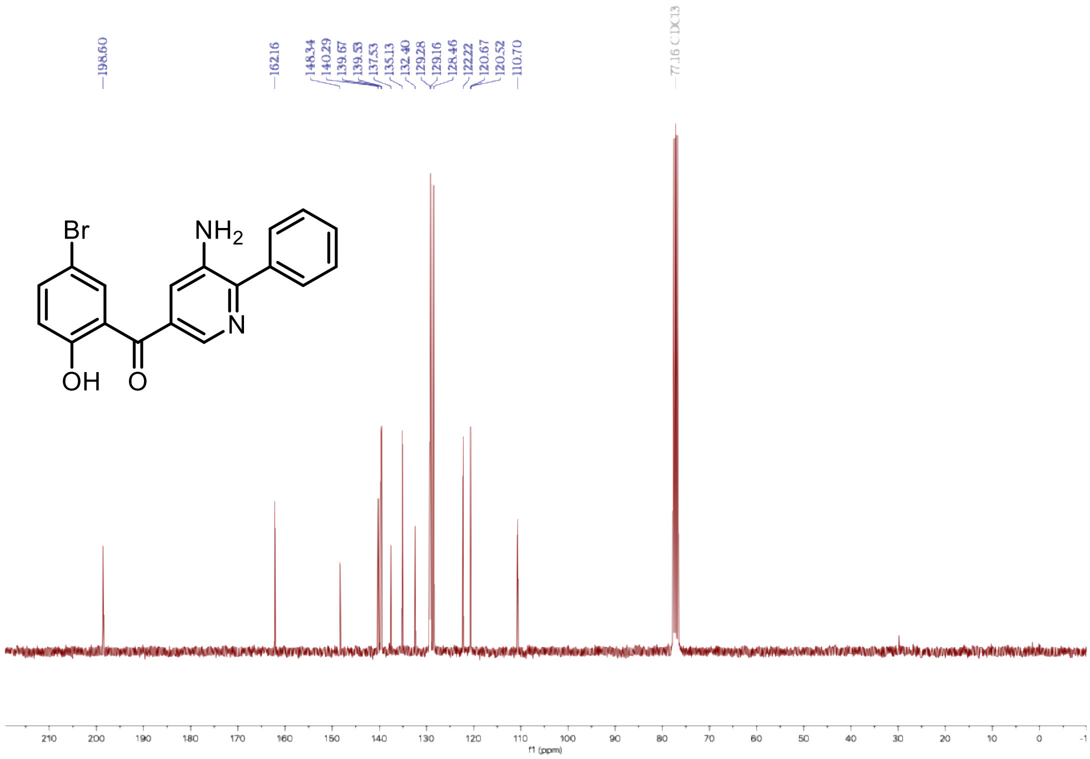

Figure S45 – ^{13}C-NMR (75 MHz, $CDCl_3$) of 3-aminopyridine **3b**.

(5-amino-6-phenylpyridin-3-yl)(5-chloro-2-hydroxyphenyl)methanone (3c)

Following the general procedure using 6-chloro-4-oxo-4*H*-chromene-3-carbaldehyde (95.8 mg) **1c** and 1-(2-oxo-2-phenylethyl)pyridin-1-ium bromide **2a** (191.7 mg). After purification by column chromatography using a gradient of 0% to 40% ethyl acetate in hexane, the compound **3c** (98.2 mg, 66% yield) was obtained.

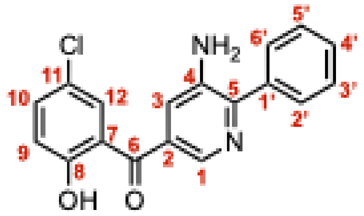

3c Light yellow solid. Melting point of 165.7–167.6 °C.

^{1}H NMR (300 MHz, CDCl$_3$): δ 11.81 (s, O*H*, 1H), 8.36 (d, *J* = 1.9 Hz, H-1, 1H), 7.77 – 7.71 (m, H-2' and 6', 2H), 7.67 (d, *J* = 2.6 Hz, H-12, 1H), 7.56 – 7.41 (m, H-10, 3', 4' and 5', 4H), 7.31 (d, *J* = 1.9 Hz, H-3, 1H), 7.04 (d, *J* = 8.9 Hz, H-9, 1H), 4.13 (s, N*H$_2$*, 2H) ppm (Figure S46).

^{13}C NMR (75 MHz, CDCl$_3$): δ 198.7 (C-6), 161.7 (C-8), 148.3 (C-5), 140.3 (C-4), 139.6 (C-1), 137.5 (C-1'), 136.8 (C-10), 132.4 (C-2), 132.1 (C-12), 129.3 (C-4'), 129.2 (C-3' and 5', 2C), 128.5 (C-2' and 6', 2C), 123.8 (C-11), 122.2 (C-3), 120.3 (C-9), 119.9 (C-7) ppm (Figure S47).

HRMS (ESI) *m/z*: [M + H]$^+$ Calcd for $C_{18}H_{14}ClN_2O_2^+$ 325.0738 ; Found 325.0735.

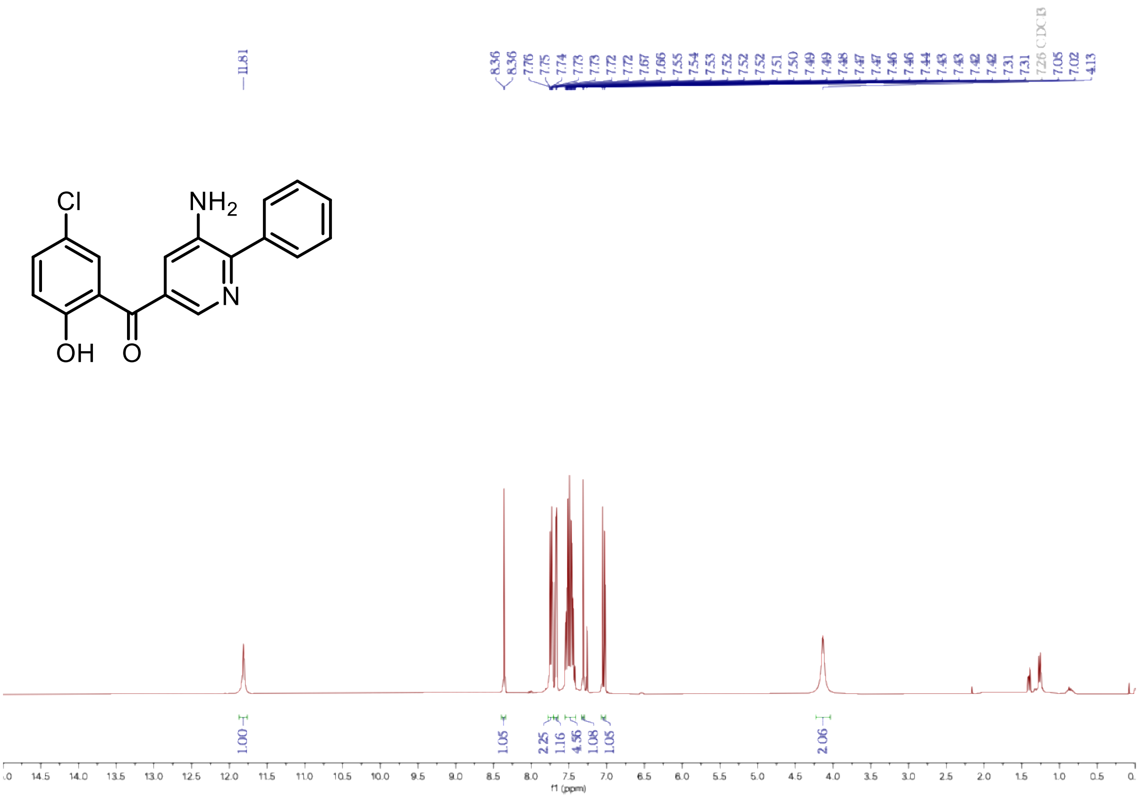

Figure S46 – ^{1}H-NMR (300 MHz, $CDCl_3$) of 3-aminopyridine **3c**.

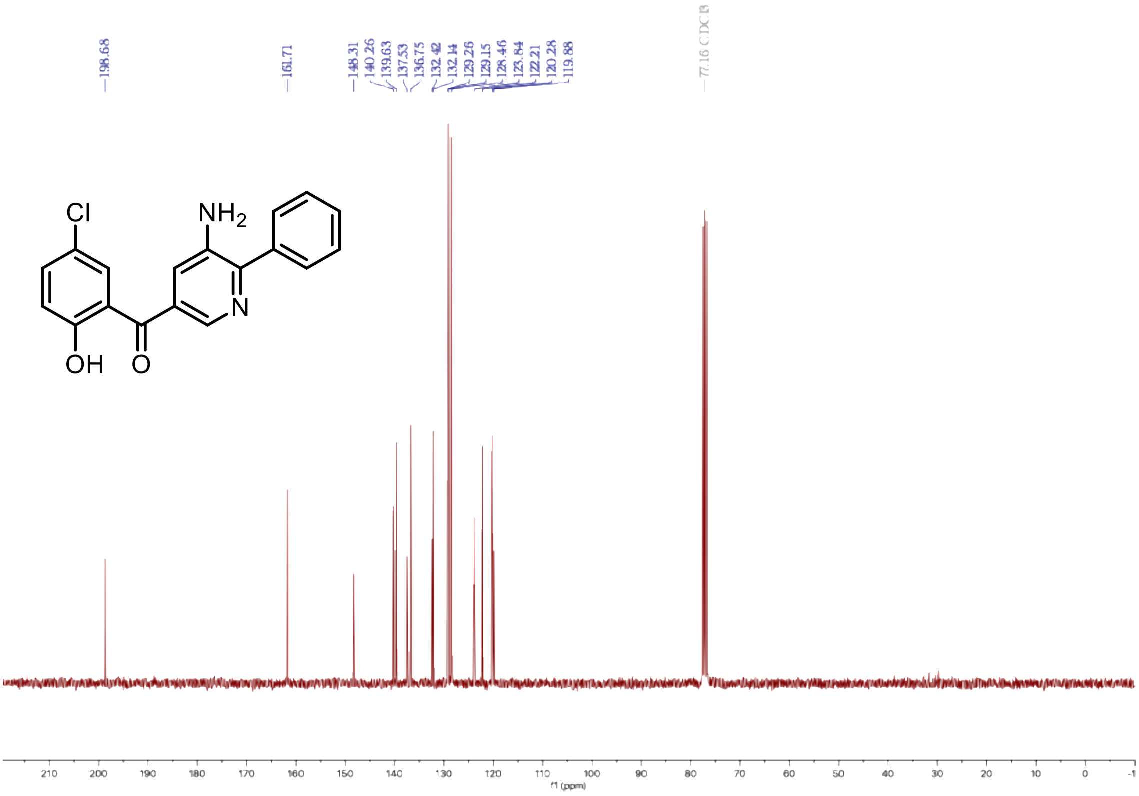

Figure S47 – ^{13}C-NMR (75 MHz, $CDCl_3$) of 3-aminopyridine **3c**.

(5-amino-6-phenylpyridin-3-yl)(5-fluoro-2-hydroxyphenyl)methanone (3d)

Following the general procedure using 6-fluoro-4-oxo-4*H*-chromene-3-carbaldehyde **1d** (88.3 mg) and 1-(2-oxo-2-phenylethyl)pyridin-1-ium bromide **2a** (191.7 mg). After purification by column chromatography using a gradient of 0% to 40% ethyl acetate in hexane, the compound **3d** (98.3 mg, 69% yield) was obtained.

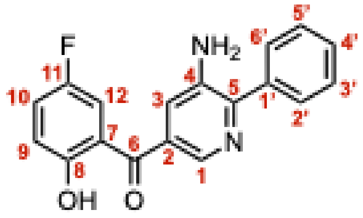

3d

Light yellow solid. Melting point of 162.5–163.5 °C.

^{1}H NMR (300 MHz, $CDCl_3$): δ 11.64 (s, O*H*, 1H), 8.35 (d, *J* = 1.9 Hz, H-1, 1H), 7.72 (dd, *J* = 8.2, 1.5 Hz, H-2' and 6', 2H), 7.55 – 7.42 (m, H-3', 4' and 5', 3H), 7.38 (dd, *J* = 8.8, 3.1 Hz, H-12, 1H), 7.31 – 7.22 (m, H-3 and 10, 2H), 7.04 (dd, *J* = 9.1, 4.5 Hz, H-9, 1H), 4.14 (s, N*H₂*, 2H) ppm (Figure S48).

^{13}C NMR (75 MHz, $CDCl_3$): δ 198.7 (d, *J* = 2.5 Hz, C-6), 159.3 (d, *J* = 1.5 Hz, C-8), 154.7 (d, *J* = 239.1 Hz, C-11), 148.2 (C-5), 140.2 (C-4), 139.5 (C-1), 137.5 (C-1'), 132.5 (C-2), 129.2 (C-4'), 129.1 (C-3' and 5', 2C), 128.4 (C-2' and 6', 2C), 124.5 (d, *J* = 23.7 Hz, C-10), 122.2 (C-3), 119.9 (d, *J* = 7.2 Hz, C-9), 118.8 (d, *J* = 6.4 Hz, C-7), 118.0 (d, *J* = 23.9 Hz, C-12) ppm (Figure S49).

^{19}F NMR (282 MHz, $CDCl_3$): δ -119.74 – -120.08 (m) ppm (Figure S50).

HRMS (ESI) *m/z*: $[M + H]^+$ Calcd for $C_{18}H_{14}FN_2O_2^+$ 309.1034 ; Found 309.1030.

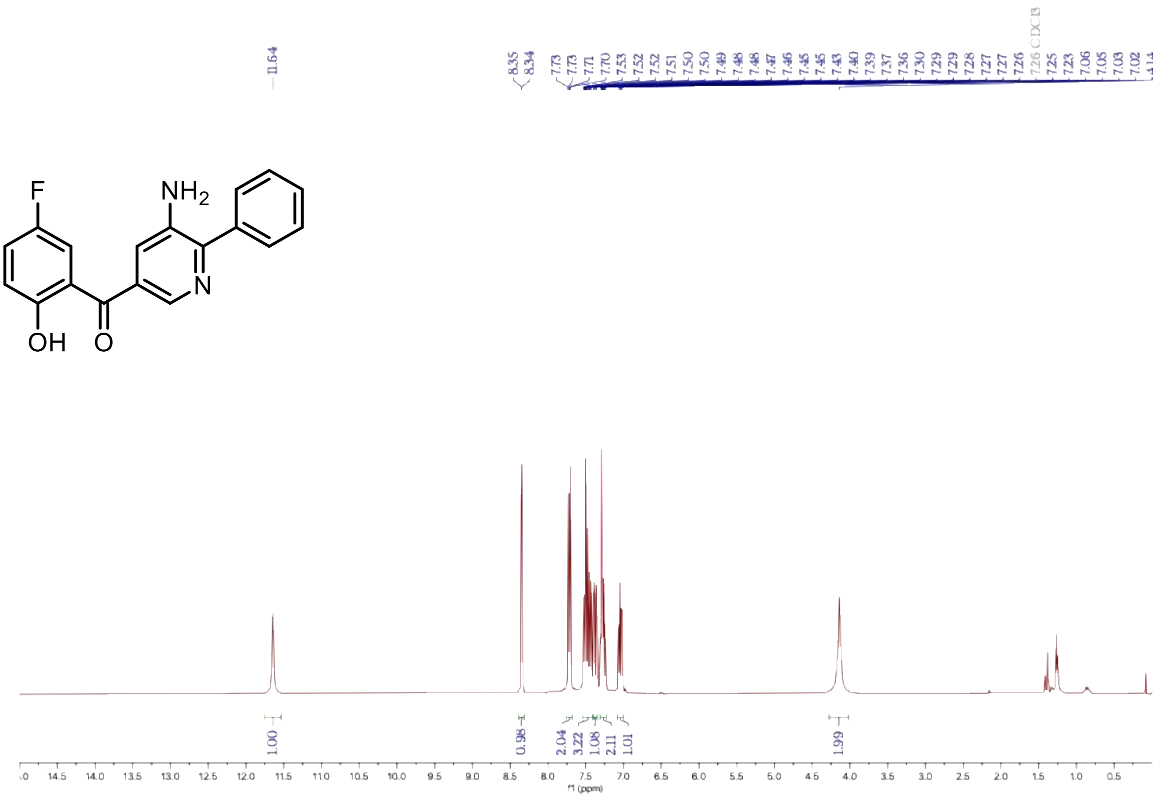

Figure S48 – ^{1}H-NMR (300 MHz, $CDCl_3$) of 3-aminopyridine **3d**.

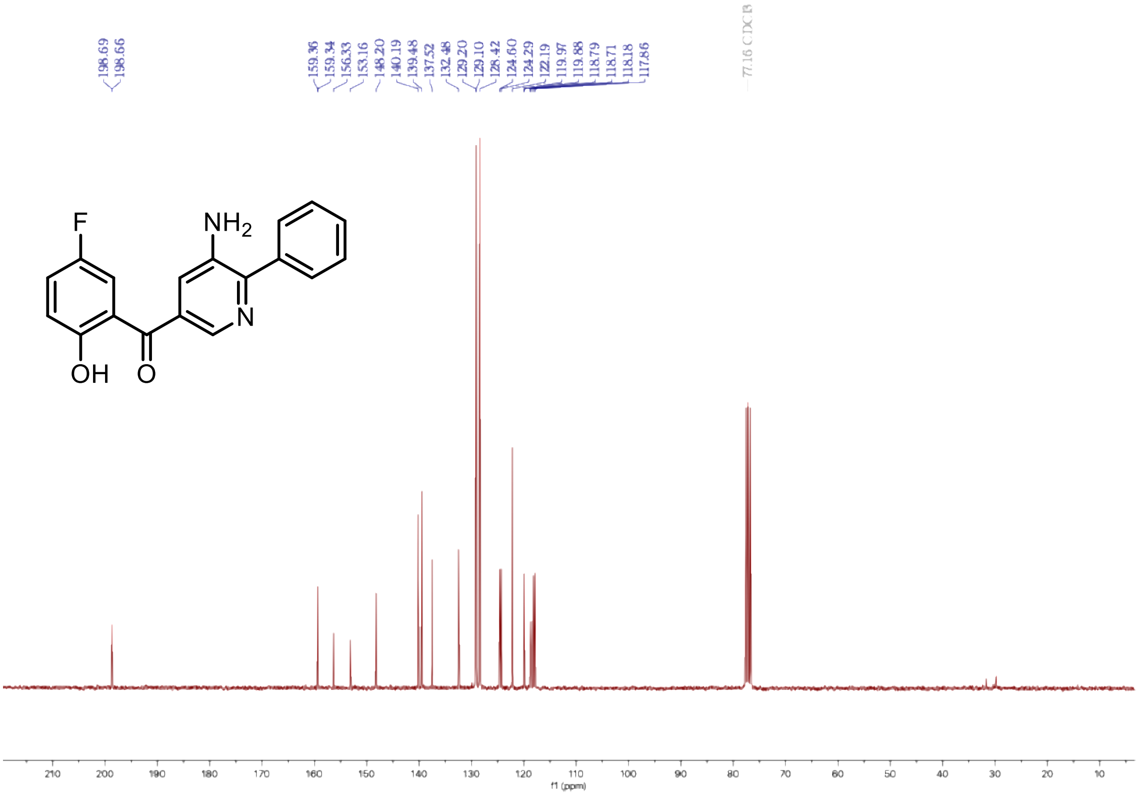

Figure S49 – ^{13}C-NMR (75 MHz, $CDCl_3$) of 3-aminopyridine **3d**.

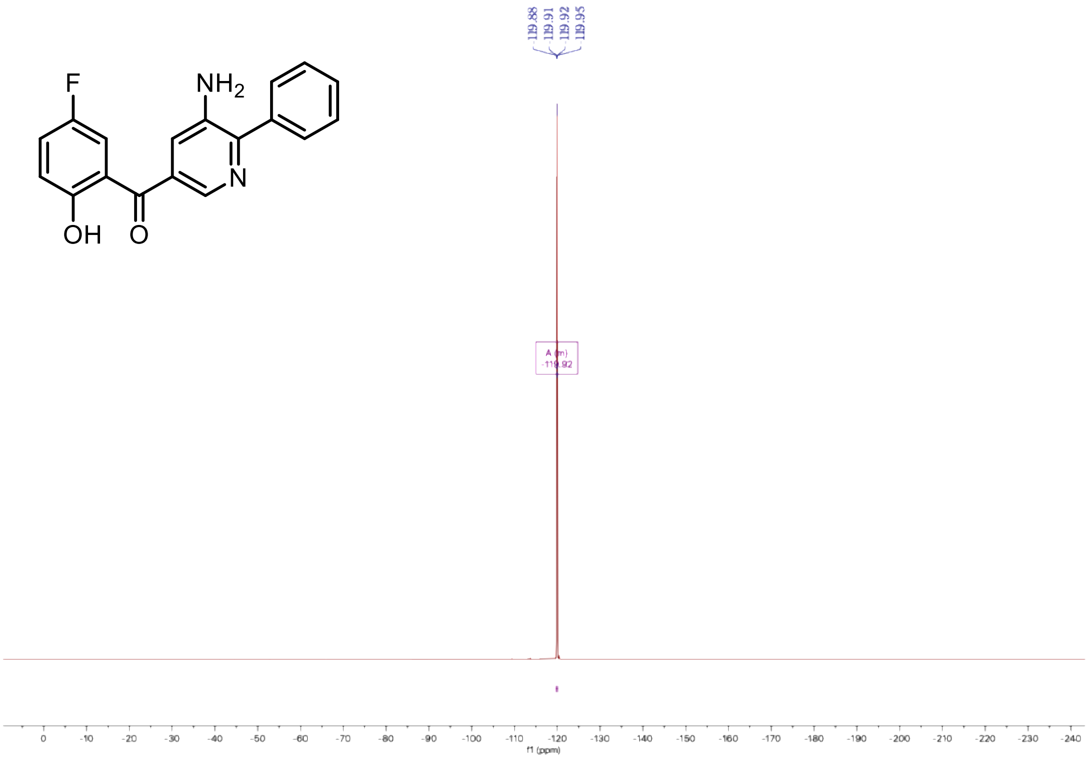

Figure S50 – ^{19}F-NMR (282 MHz, $CDCl_3$) of 3-aminopyridine **3d**.

(5-amino-6-phenylpyridin-3-yl)(2-hydroxy-5-methylphenyl)methanone (3e)

Following the general procedure using 6-methyl-4-oxo-4*H*-chromene-3-carbaldehyde (86.4 mg) **1e** and 1-(2-oxo-2-phenylethyl)pyridin-1-ium bromide **2a** (191.7 mg). After purification by column chromatography using a gradient of 0% to 40% ethyl acetate in hexane, the compound **3e** (97.6 mg, 70% yield) was obtained.

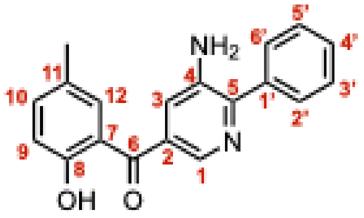

3e

Yellow solid. Melting point of 148.2–149.0 °C.

^{1}H NMR (300 MHz, CDCl$_3$): δ 11.76 (s, O*H*, 1H), 8.33 (d, *J* = 1.9 Hz, H-1, 1H), 7.72 (dd, *J* = 8.3, 1.5 Hz, H-2' and 6', 2H), 7.54 – 7.38 (m, H-12, 3', 4' and 5', 4H), 7.33 (dd, *J* = 8.6, 2.1 Hz, H-10, 1H), 7.29 (d, *J* = 1.8 Hz, H-3, 1H), 6.97 (d, *J* = 8.5 Hz, H-9, 1H), 4.15 (s, NH_2, 2H), 2.25 (s, CH_3, 3H) ppm (Figure S51).

^{13}C NMR (75 MHz, CDCl$_3$): δ 199.4 (C-6), 161.0 (C-8), 147.6 (C-5), 140.2 (C-4), 139.6 (C-1), 137.9 (C-10), 137.6 (C-1'), 133.1 (C-2), 132.9 (C-12), 129.0 (C-4'), 129.0 (C-3' and 5', 2C), 128.4 (C-2' and 6', 2C), 128.2 (C-11), 122.4 (C-3), 118.9 (C-7), 118.2 (C-9), 20.5 (*C*H$_3$) ppm (Figure S52).

HRMS (ESI) *m/z*: [M + H]$^+$ Calcd for $C_{19}H_{17}N_2O_2^+$ 305.1285 ; Found 305.1280.

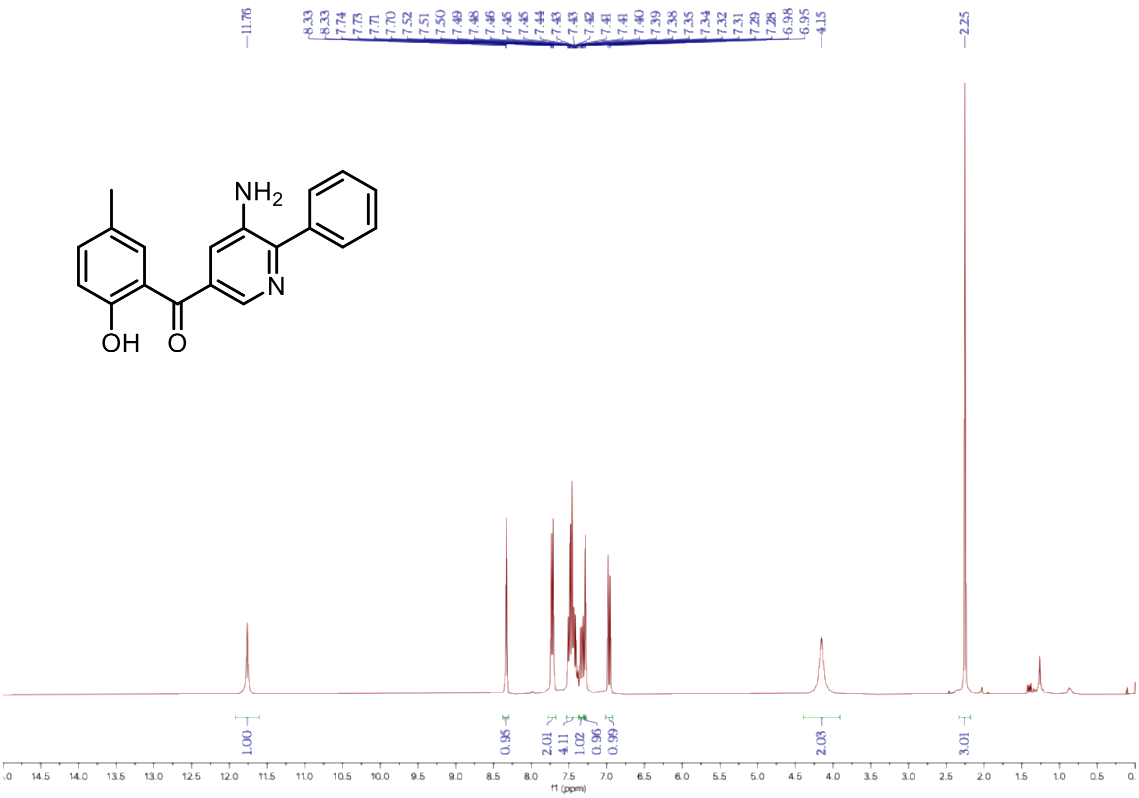

Figure S51 – ^{1}H-NMR (300 MHz, $CDCl_3$) of 3-aminopyridine **3e**.

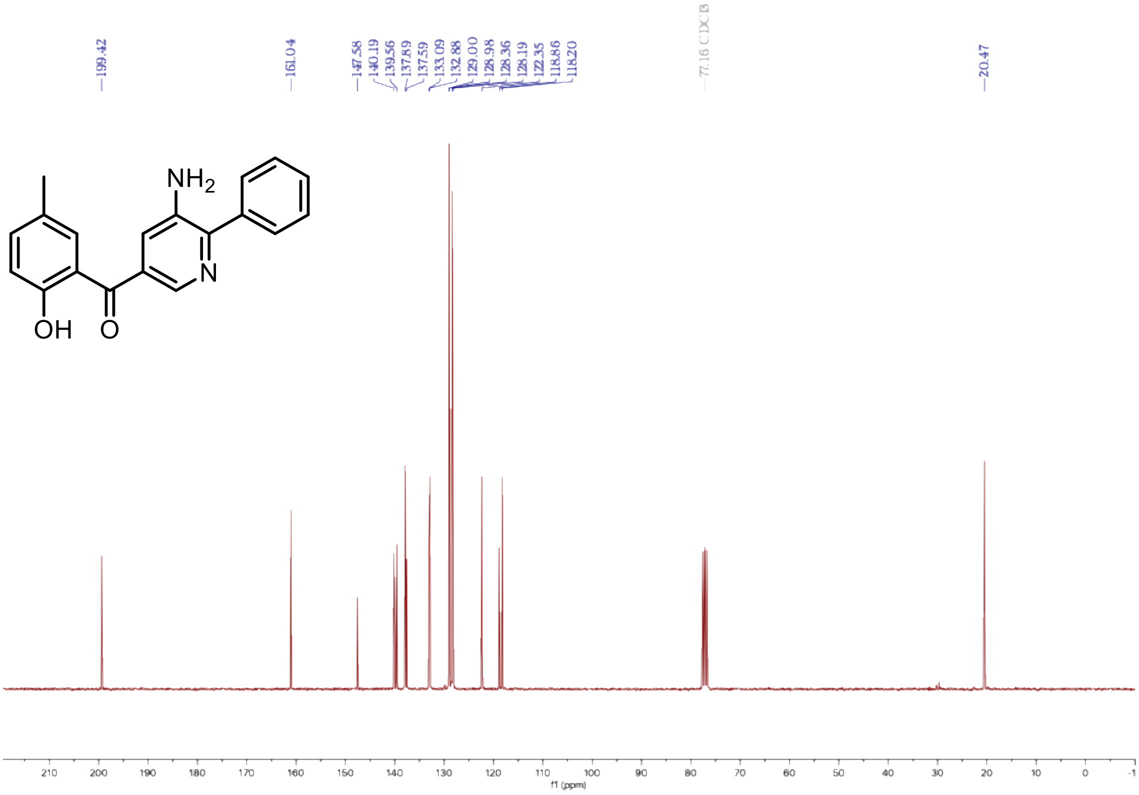

Figure S52 – ^{13}C-NMR (75 MHz, $CDCl_3$) of 3-aminopyridine **3e**.

(5-amino-6-phenylpyridin-3-yl)(2-hydroxy-5-nitrophenyl)methanone (3f)

Following the general procedure using 6-nitro-4-oxo-4*H*-chromene-3-carbaldehyde (100.7 mg) **1f** and 1-(2-oxo-2-phenylethyl)pyridin-1-ium bromide **2a** (191.7 mg), but instead of EtOH, toluene was used as the solvent. After purification by column chromatography using a gradient of 0% to 40% ethyl acetate in hexane, the compound **3f** (92.2 mg, 60% yield) was obtained.

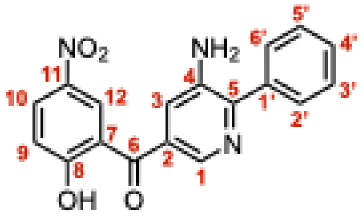

3f

Yellow solid. Melting point of 171.2–172.3 °C.

^{1}H NMR (300 MHz, DMSO-*d$_6$*): δ 11.77 (s, O*H*, 1H), 8.32 (dd, *J* = 9.0, 2.9 Hz, H-10, 1H), 8.25 (d, *J* = 2.9 Hz, H-12, 1H), 8.19 (d, *J* = 1.9 Hz, H-1, 1H), 7.75 – 7.69 (m, H-2' and 6', 2H), 7.53 – 7.39 (m, H-3, 3', 4' and 5', 4H), 7.16 (d, *J* = 9.1 Hz, H-9, 1H), 5.42 (s, N*H$_2$*, 2H) ppm (Figure S53).

^{13}C NMR (75 MHz, DMSO-*d$_6$*): δ 194.0 (C-6), 162.1 (C-8), 147.0 (C-5), 142.3 (C-4), 139.9 (C-11), 138.9 (C-1), 138.6 (C-1'), 131.8 (C-2), 129.0 (C-3' and 5', 2C), 128.9 (C-4'), 128.7 (C-2' and 6', 2C), 128.5 (C-10), 126.8 (C-7), 126.3 (C-12), 122.6 (C-3), 117.7 (C-9) ppm (Figure S54).

HRMS (ESI) *m/z*: $[M + H]^+$ Calcd for $C_{19}H_{17}N_3O_4^+$ 336.0979 ; Found 336.0973.

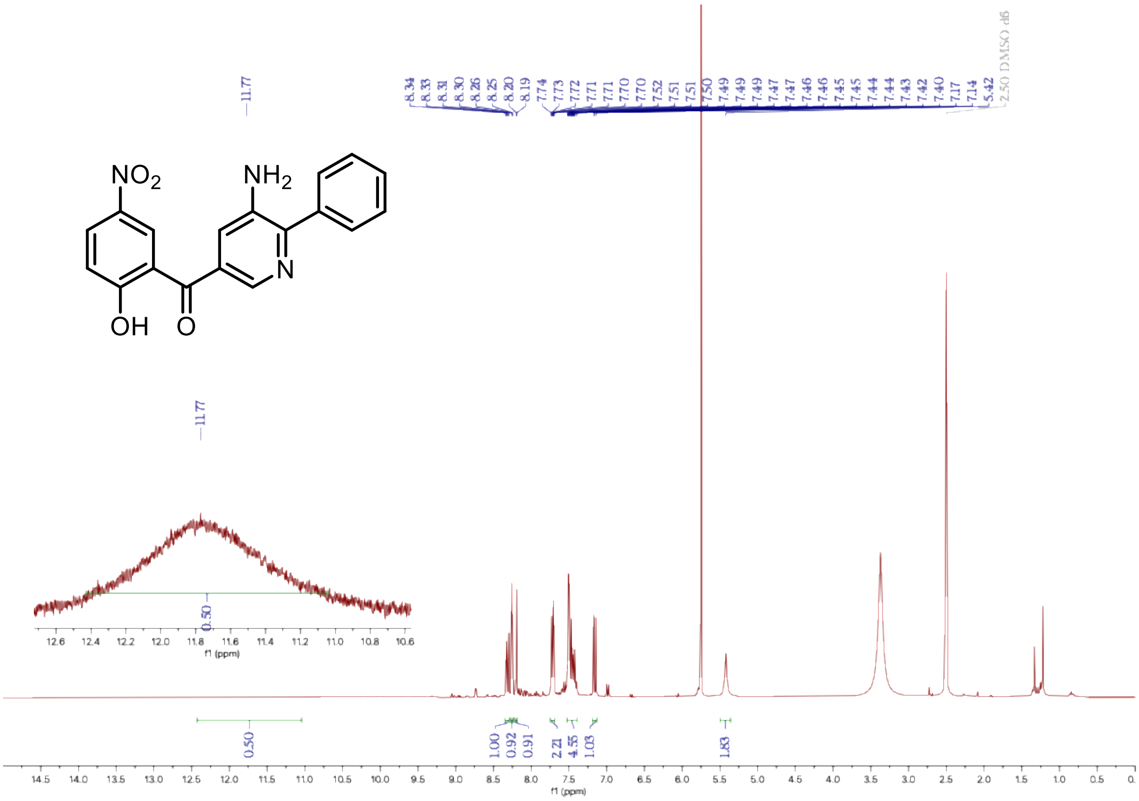

Figure S53 – ^{1}H-NMR (300 MHz, DMSO-d_6) of 3-aminopyridine **3f**.

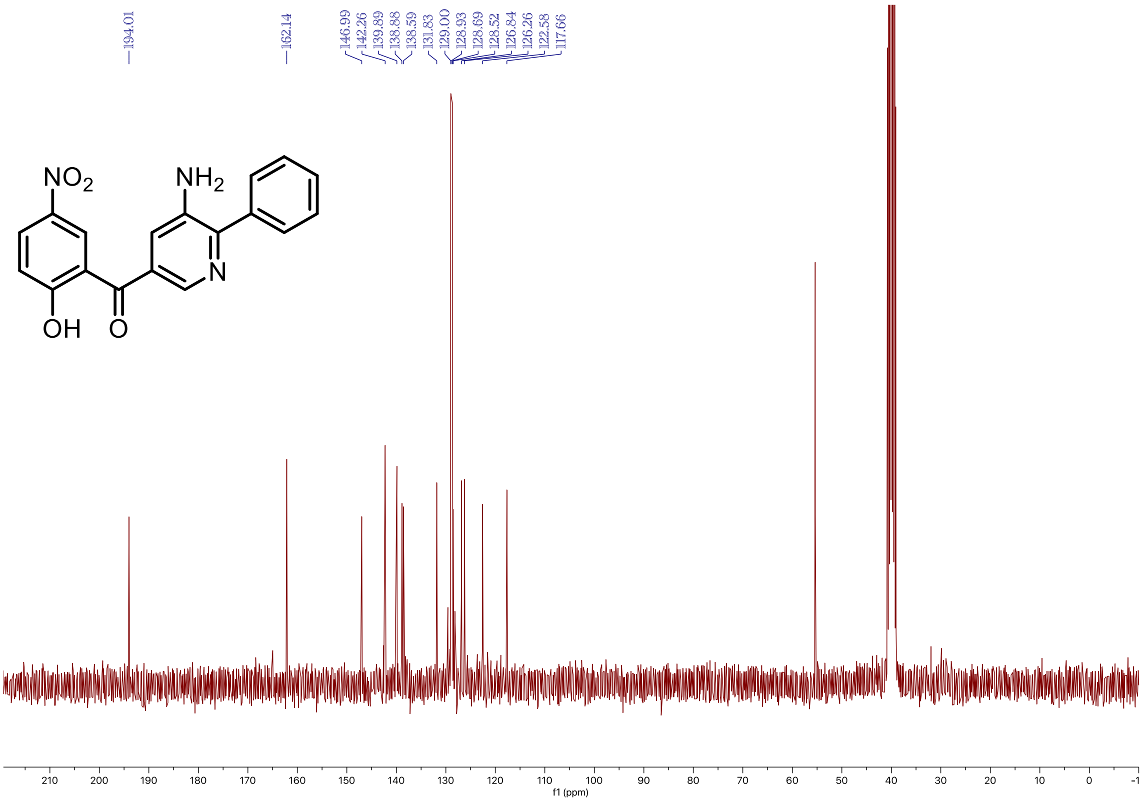

Figure S54 – ^{13}C-NMR (75 MHz, DMSO-d_6) of 3-aminopyridine **3f**.

(5-amino-6-phenylpyridin-3-yl)(5-chloro-2-hydroxy-4-methylphenyl)methanone (3g)

Following the general procedure using 6-chloro-7-methyl-4-oxo-4*H*-chromene-3-carbaldehyde **1g** (102.3 mg) and 1-(2-oxo-2-phenylethyl)pyridin-1-ium bromide **2a** (191.7 mg). After purification by column chromatography using a gradient of 0% to 40% ethyl acetate in hexane, the compound **3g** (108.2 mg, 70% yield) was obtained.

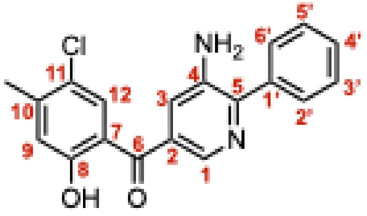

3g

Yellow solid. Melting point of 197.8–198.4 °C.

^{1}H NMR (300 MHz, $CDCl_3$): δ 11.83 (s, O*H*, 1H), 8.36 (d, *J* = 1.9 Hz, H-1, 1H), 7.77 – 7.72 (m, H-2' and 6', 2H), 7.66 (s, H-12, 1H), 7.56 – 7.42 (m, H-3', 4' and 5', 3H), 7.32 (d, *J* = 1.9 Hz, H-3, 1H), 6.98 (s, H-9, 1H), 4.11 (s, NH_2, 2H), 2.41 (s, CH_3, 3H) ppm (Figure S55).

^{13}C NMR (75 MHz, $CDCl_3$): δ 198.3 (C-6), 161.7 (C-8), 148.2 (C-5), 146.4 (C-10), 140.2 (C-4), 139.7 (C-1), 137.6 (C-1'), 132.7 (C-2), 132.6 (C-12), 129.2 (C-4'), 129.2 (C-3' and 5', 2C), 128.5 (C-2' and 6', 2C), 124.5 (C-11), 122.2 (C-3), 120.7 (C-9), 118.2 (C-7), 21.1 (*C*H3) ppm (Figure S56).

HRMS (ESI) *m/z*: $[M + H]^+$ Calcd for $C_{19}H_{16}ClN_2O_2^+$ 339.0895 ; Found 339.0891.

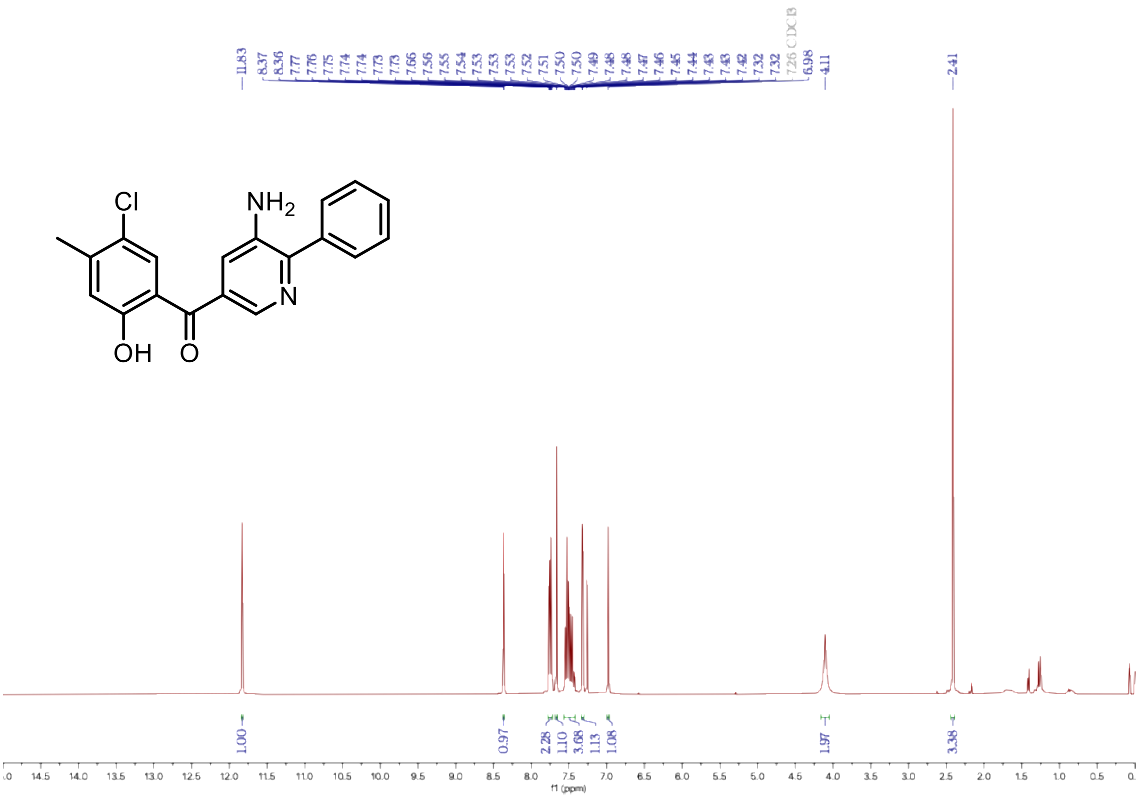

Figure S55 – ^{1}H-NMR (300 MHz, $CDCl_3$) of 3-aminopyridine **3g**.

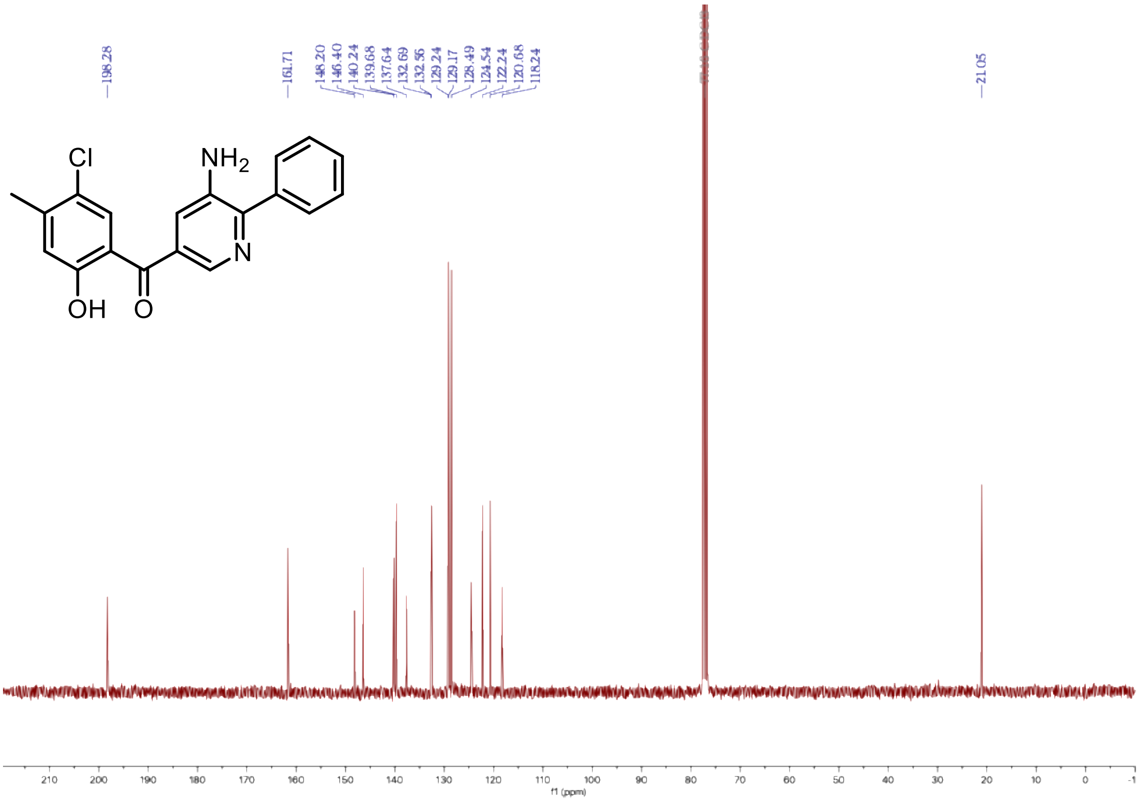

Figure S56 – ^{13}C-NMR (75 MHz, $CDCl_3$) of 3-aminopyridine **3g**.

(5-amino-6-phenylpyridin-3-yl)(2-hydroxy-6-methoxyphenyl)methanone (3h)

Following the general procedure using 5-methoxy-4-oxo-4*H*-chromene-3-carbaldehyde **1h** (93.8 mg) and 1-(2-oxo-2-phenylethyl)pyridin-1-ium bromide **2a** (191.7 mg). After purification by column chromatography using a gradient of 0% to 40% ethyl acetate in hexane, the compound **3h** (76.5 mg, 52% yield) was obtained.

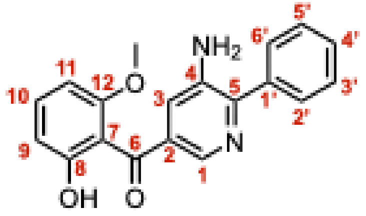

3h Yellow solid. Melting point of 198.0–199.3 °C.

^{1}H NMR (300 MHz, $CDCl_3$): δ 10.81 (s, O*H*, 1H), 8.24 (d, *J* = 1.9 Hz, H-1, 1H), 7.75 – 7.68 (m, H-2' and 6', 2H), 7.52 – 7.35 (m, H-3, 10, 3', 4' and 5', 5H), 6.65 (dd, *J* = 8.4, 0.9 Hz, H-9, 1H), 6.43 (dd, *J* = 8.4, 0.9 Hz, H-11, 1H), 3.99 (s, N*H₂*, 2H), 3.61 (s, OC*H₃*, 3H) ppm (Figure S57).

^{13}C NMR (75 MHz, $CDCl_3$): δ 197.8 (C-6), 162.0 (C-8), 160.3 (C-12), 147.2 (C-5), 140.2 (C-1), 139.8 (C-4), 137.9 (C-1'), 136.1 (C-10), 135.8 (C-2), 129.0 (C-3' and 5', 2C), 128.9 (C-4'), 128.5 (C-2' and 6', 2C), 122.0 (C-3), 111.9 (C-7), 110.5 (C-9), 102.4 (C-11), 55.7 (O*C*H₃) ppm (Figure S58).

HRMS (ESI) *m/z*: [M + H]+ Calcd for $C_{19}H_{17}N_2O_3^+$ 321.1234 ; Found 321.1229.

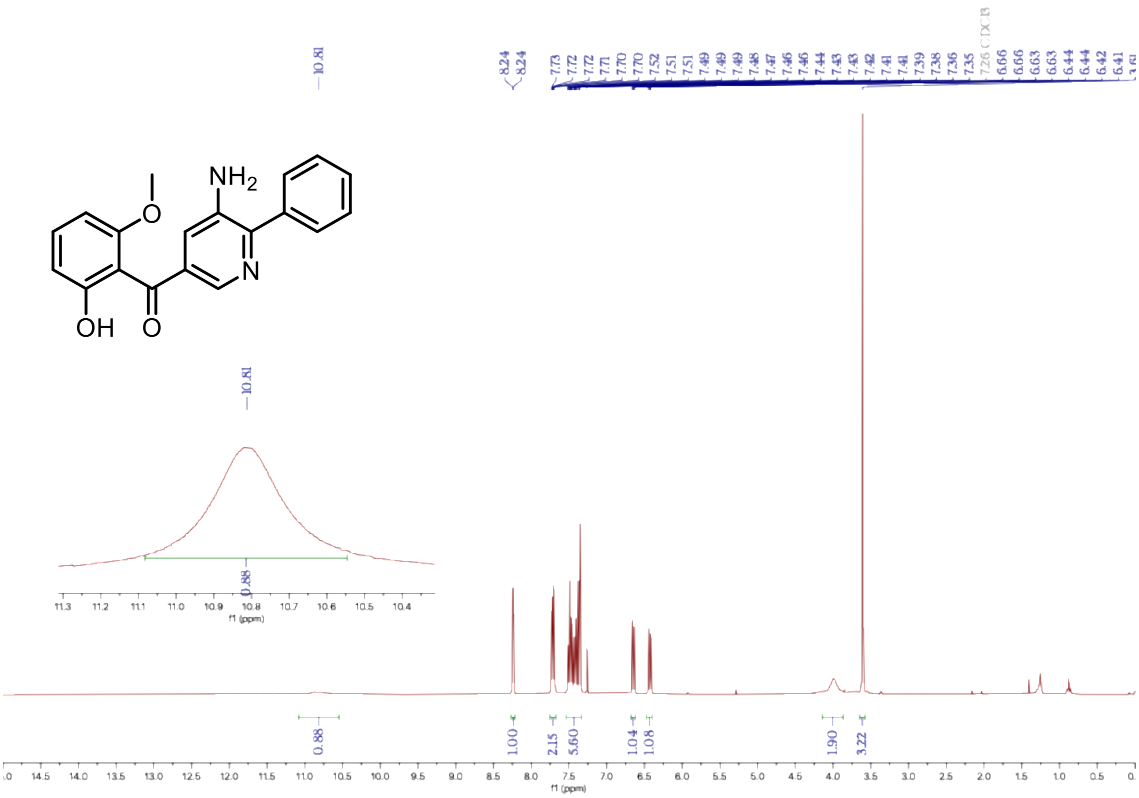

Figure S57 – ^{1}H-NMR (300 MHz, $CDCl_3$) of 3-aminopyridine **3h**.

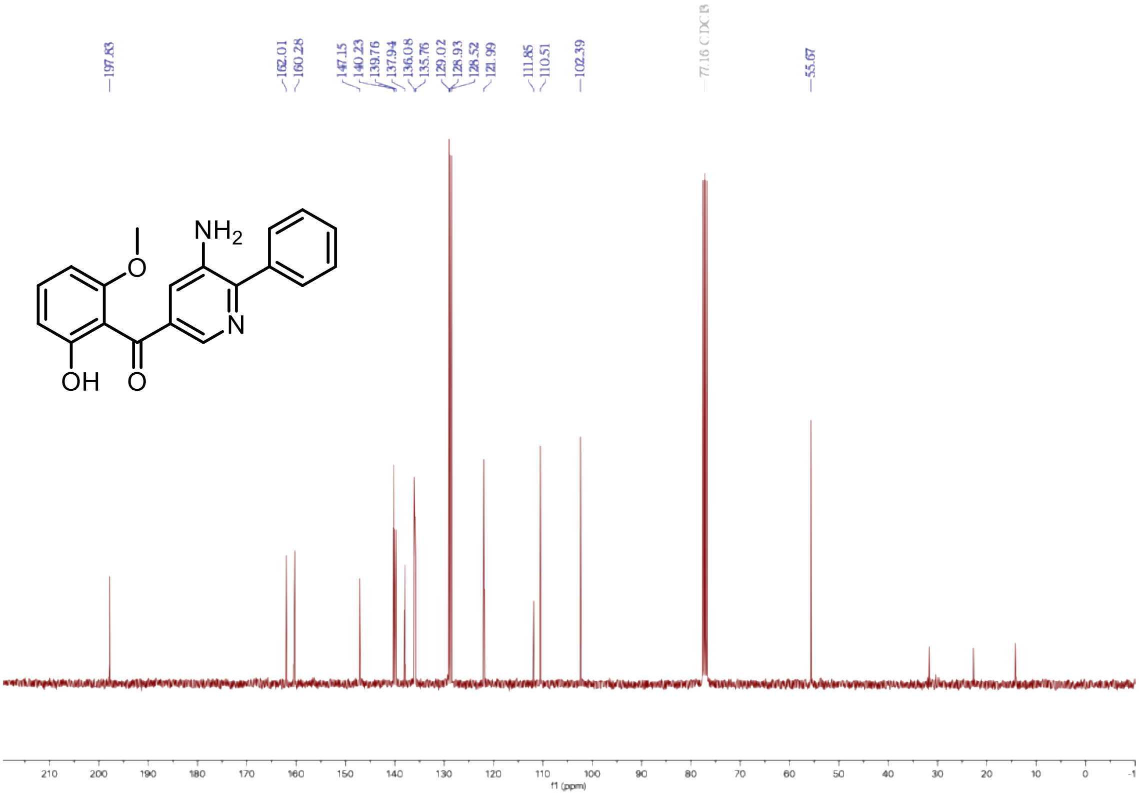

Figure S58 – ^{13}C-NMR (75 MHz, $CDCl_3$) of 3-aminopyridine **3h**.

(5-amino-6-phenylpyridin-3-yl)(2-hydroxy-4-methoxyphenyl)methanone (3i)

Following the general procedure using 7-methoxy-4-oxo-4*H*-chromene-3-carbaldehyde **1i** (100.7 mg) and 1-(2-oxo-2-phenylethyl)pyridin-1-ium bromide **2a** (191.7 mg). After purification by column chromatography using a gradient of 0% to 40% ethyl acetate in hexane, the compound **3i** (109.8 mg, 75% yield) was obtained.

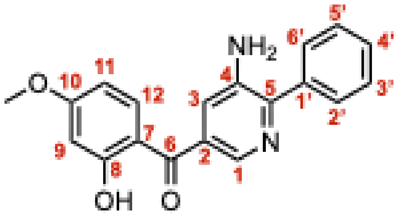

3i

Yellow solid. Melting point of 195.5–197.0 °C.

^{1}H NMR (300 MHz, CDCl$_3$): δ 12.58 (s, O*H*, 1H), 8.34 (d, *J* = 1.8 Hz, H-1, 1H), 7.76 – 7.69 (m, H-2' and 6', 2H), 7.61 (d, *J* = 9.0 Hz, H-12, 1H), 7.55 – 7.41 (m, H-3', 4' and 5', 3H), 7.30 (d, *J* = 1.8 Hz, H-3, 1H), 6.53 (d, *J* = 2.5 Hz, H-9, 1H), 6.45 (dd, *J* = 9.0, 2.5 Hz, H-11, 1H), 4.06 (s, N*H$_2$*, 2H), 3.87 (s, OC*H$_3$*, 3H) ppm (Figure S59).

^{13}C NMR (75 MHz, CDCl$_3$): δ 197.8 (C-6), 166.8 (C-10), 166.5 (C-8), 147.6 (C-5), 140.1 (C-4), 139.6 (C-1), 137.8 (C-1'), 135.1 (C-12), 133.5 (C-2), 129.1 (C-3' and 5'), 129.1 (C-4'), 128.5 (C-2' and 6'), 122.4 (C-3), 113.4 (C-7), 107.9 (C-11), 101.3 (C-9), 55.9 (O*C*H$_3$) ppm (Figure S60).

HRMS (ESI) *m/z*: [M + H]$^+$ Calcd for $C_{19}H_{17}N_2O_3^+$ 321.1234 ; Found 321.1229.

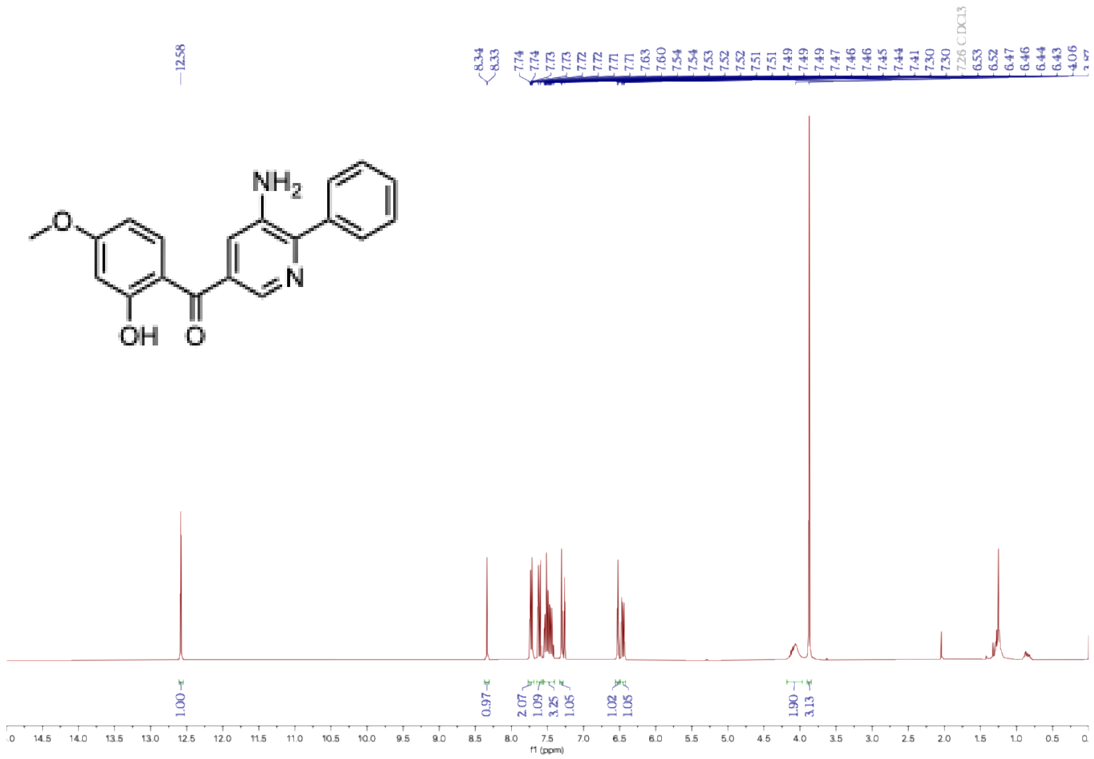

Figure S59 – ^{1}H-NMR (300 MHz, $CDCl_3$) of 3-aminopyridine **3i**.

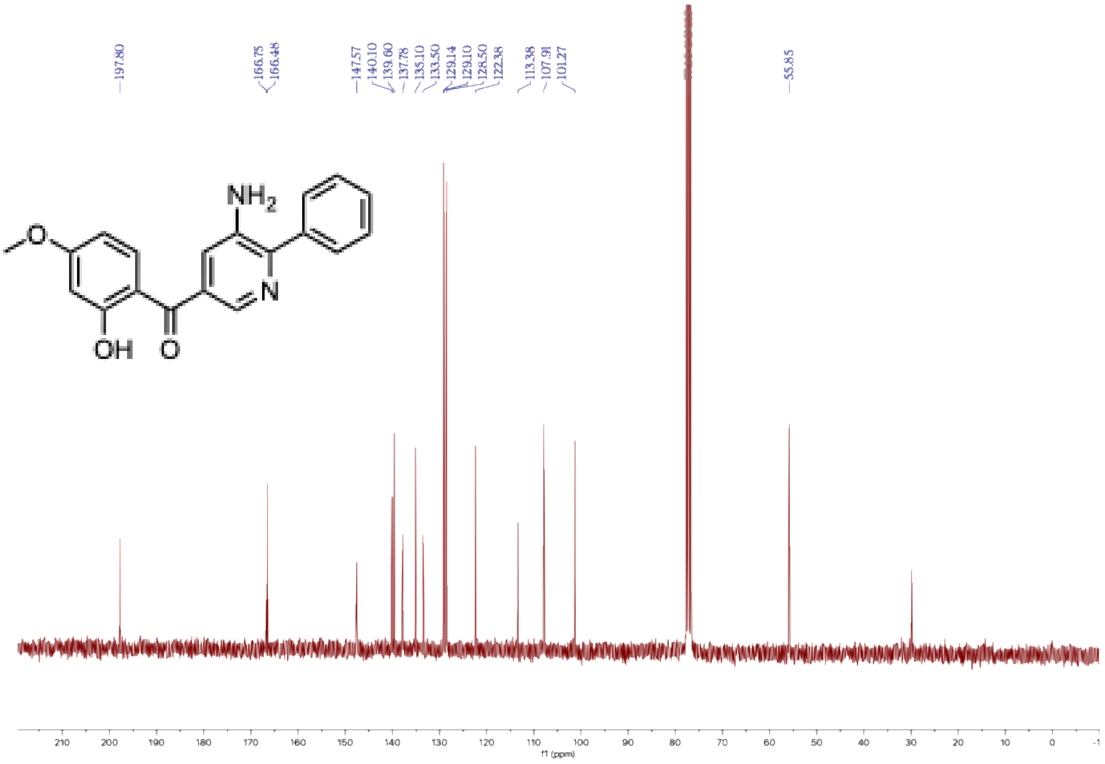

Figure S60 – ^{13}C-NMR (75 MHz, $CDCl_3$) of 3-aminopyridine **3i**.

(5-amino-6-phenylpyridin-3-yl)(1-hydroxynaphthalen-2-yl)methanone (3j)

Following the general procedure using 4-oxo-4*H*-benzo[*h*]chromene-3-carbaldehyde **1j** (100.7 mg) and 1-(2-oxo-2-phenylethyl)pyridin-1-ium bromide **2a** (191.7 mg). After purification by column chromatography using a gradient of 0% to 40% ethyl acetate in hexane, the compound **3j** (57.7 mg, 37% yield) was obtained.

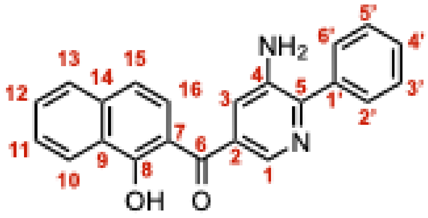

3j

Brown solid. Melting point of 198.8–201.3 °C.

^{1}H NMR (300 MHz, $CDCl_3$): δ 13.87 (s, O*H*, 1H), 8.52 (ddt, *J* = 8.3, 1.5, 0.8 Hz, H-10, 1H), 8.42 (d, *J* = 1.8 Hz, H-1, 1H), 7.79 – 7.72 (m, H-16, 2' and 6', 3H), 7.66 (ddd, *J* = 8.2, 6.8, 1.4 Hz, H-11, 1H), 7.63 (d, *J* = 8.9 Hz, H-13, 1H), 7.59 – 7.41 (m, H-3', 4' and 5', 4H), 7.35 (d, *J* = 1.8 Hz, H-3, 1H), 7.25 (dd, H-15, *J* = 9.1, 0.8 Hz, 1H), 4.10 (s, NH_2, 2H) ppm (Figure S61).

^{13}C NMR (75 MHz, $CDCl_3$): δ 199.3 (C-6), 164.2 (C-8), 147.7 (C-5), 140.1 (C-4), 139.9 (C-1), 137.8 (C-14 or 1'), 137.6 (C-14 or 1'), 133.4 (C-2), 130.7 (C-4'), 129.1 (C-16, 3' and 5', 3C), 128.5 (C-2' and 6', 2C), 127.6 (C-12), 127.0 (C-13), 126.3 (C-11), 125.3 (C-9), 124.7 (C-10), 122.5 (C-3), 118.4 (C-15), 112.8 (C-7) ppm (Figure S62).

HRMS (ESI) *m/z*: $[M + H]^+$ Calcd for $C_{22}H_{17}N_2O_2^+$ 341.1285 ; Found 341.1278.

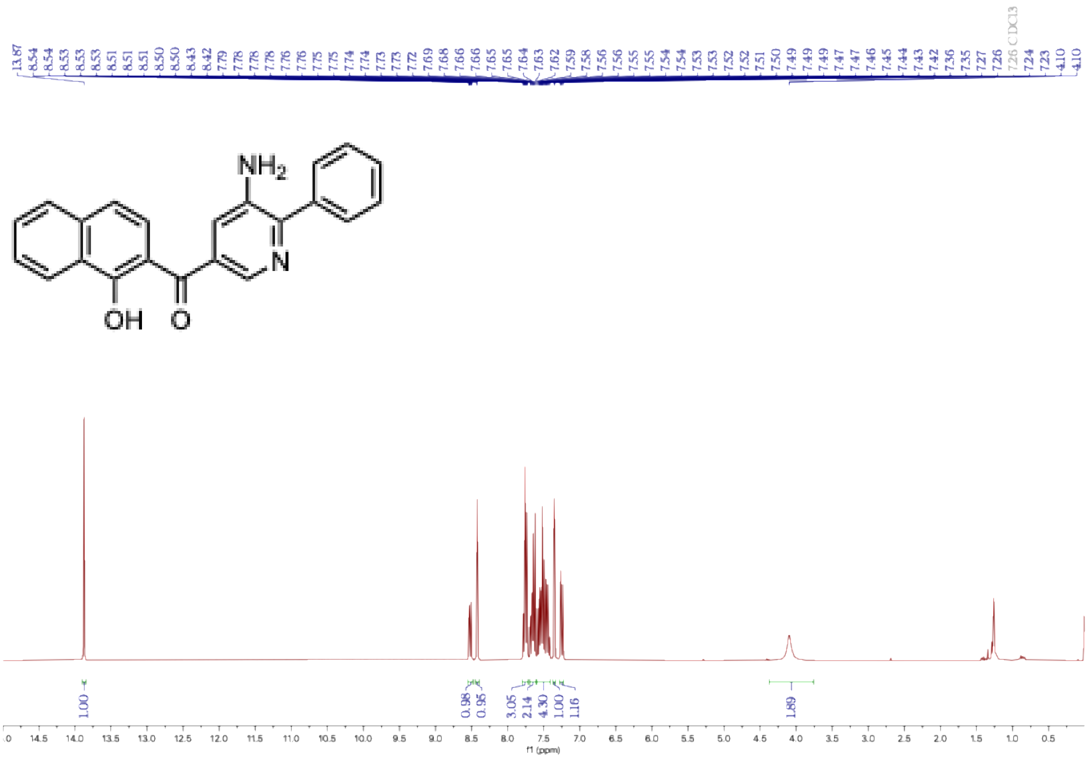

Figure S61 – ^{1}H-NMR (300 MHz, $CDCl_3$) of 3-aminopyridine **3j**.

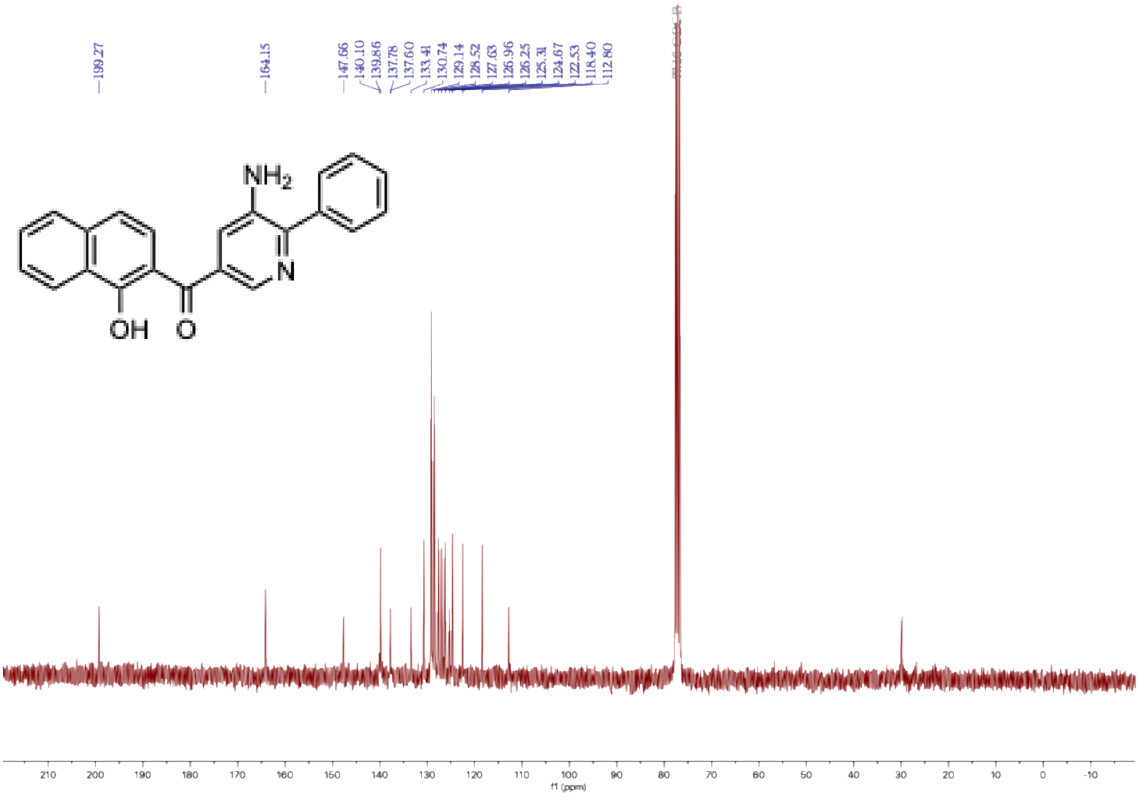

Figure S62 – ^{13}C-NMR (75 MHz, $CDCl_3$) of 3-aminopyridine **3j**.

[5-amino-6-(4-chlorophenyl)pyridin-3-yl](2-hydroxyphenyl)methanone (3k)

Following the general procedure using 4-oxo-4*H*-chromene-3-carbaldehyde **1a** (80.0 mg) and 1-[2-(4-chlorophenyl)-2-oxoethyl]pyridin-1-ium bromide **2b** (215.4 mg). After purification by column chromatography using a gradient of 0% to 40% ethyl acetate in hexane, the compound **3k** (131.1 mg, 88% yield) was obtained.

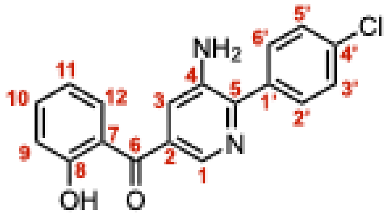

3k

Yellow solid. Melting point of 164.8–165.6 °C.

¹H NMR (300 MHz, CDCl₃): δ 11.90 (s, O*H*, 1H), 8.36 (d, *J* = 1.8 Hz, H-1, 1H), 7.74 – 7.64 (m, H-12, 2' and 6', 3H), 7.54 (ddd, *J* = 8.6, 7.2, 1.7 Hz, H-10, 1H), 7.50 – 7.46 (m, H-3' and 5', 2H), 7.33 (d, *J* = 1.8 Hz, H-3, 1H), 7.08 (dd, *J* = 8.6, 1.2 Hz, H-9, 1H), 6.91 (ddd, *J* = 8.2, 7.2, 1.2 Hz, H-11, 1H), 4.06 (s, NH_2, 2H) ppm (Figure S63).

^{13}C NMR (75 MHz, $CDCl_3$): δ 199.4 (C-6), 163.2 (C-8), 146.4 (C-5), 140.0 (C-4), 139.8 (C-1), 136.9 (C-10), 136.0 (C-1'), 135.1 (2C, C-2 and 4'), 133.3 (C-12), 129.9 (2C, C-2' and 6'), 129.3 (2C, C-3' and 5'), 122.7 (C-3), 119.1 (C-7), 119.0 (C-11), 118.6 (C-9) ppm (Figure S64).

HRMS (ESI) *m/z*: $[M + H]^+$ Calcd for $C_{18}H_{14}ClN_2O_2^+$ 325.0738 ; Found 325.0748.

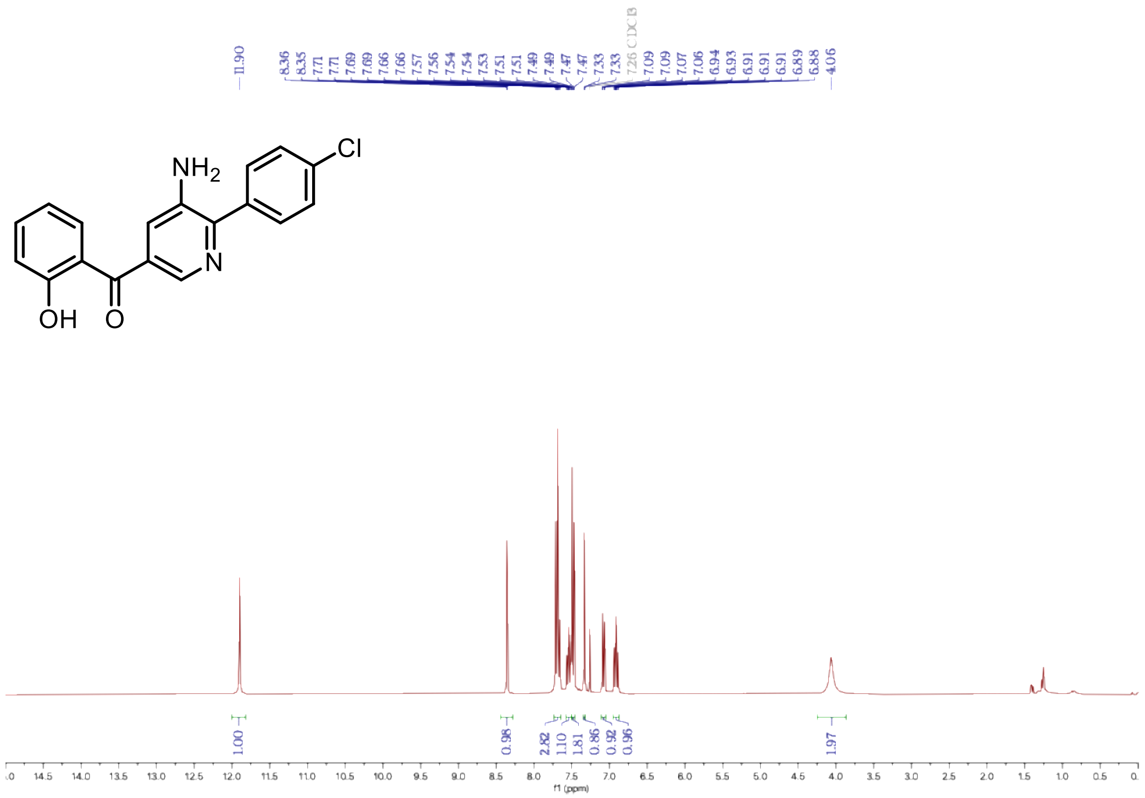

Figure S63 – ^{1}H-NMR (300 MHz, $CDCl_3$) of 3-aminopyridine **3k**.

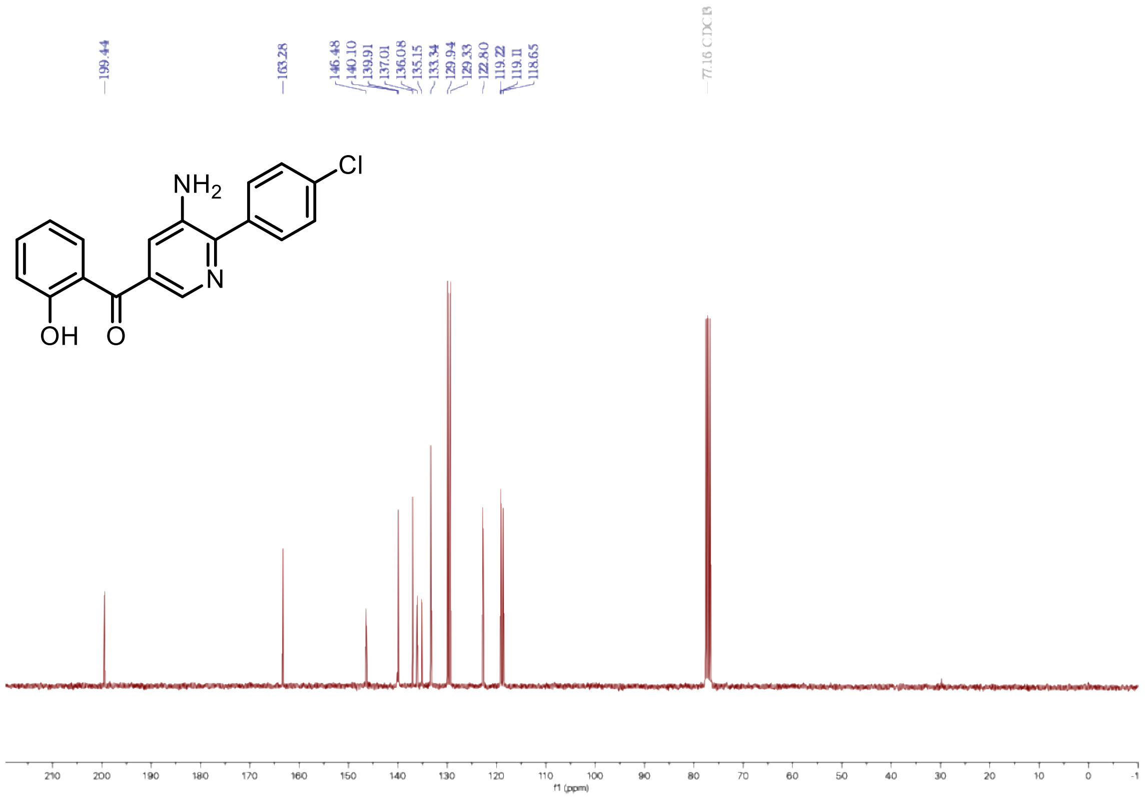

Figure S64 – ^{13}C-NMR (75 MHz, $CDCl_3$) of 3-aminopyridine **3k**.

[5-amino-6-(p-tolyl)pyridin-3-yl](2-hydroxyphenyl)methanone (3l)

Following the general procedure using 4-oxo-4*H*-chromene-3-carbaldehyde **1a** (80.0 mg) and 1-[2-oxo-2-(*p*-tolyl)ethyl]pyridin-1-ium bromide **2c** (201.3 mg). After purification by column chromatography using a gradient of 0% to 40% ethyl acetate in hexane, the compound **3l** (109.2 mg, 78% yield) was obtained.

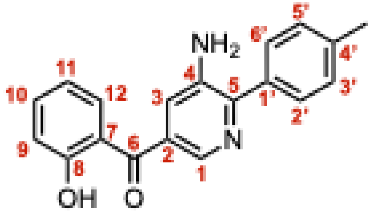

3l

Yellow solid. Melting point of 192.4–193.5 °C.

^{1}H NMR (300 MHz, $CDCl_3$): δ 11.94 (s, O*H*, 1H), 8.37 (d, *J* = 1.8 Hz, H-1, 1H), 7.71 (dd, *J* = 8.0, 1.7 Hz, H-12, 1H), 7.64 (d, *J* = 8.1 Hz, H-2' and 6', 2H), 7.54 (ddd, *J* = 8.7, 7.2, 1.7 Hz, H-10, 1H), 7.36 – 7.30 (m, H-3, 3' and 5', 3H), 7.09 (dd, *J* = 8.4, 1.2 Hz, H-9, 1H), 6.92 (ddd, *J* = 8.2, 7.2, 1.2 Hz, H-11, 1H), 4.08 (s, NH_2, 2H), 2.43 (s, CH_3, 3H) ppm (Figure S65).

^{13}C NMR (75 MHz, $CDCl_3$): δ 199.7 (C-6), 163.3 (C-8), 148.1 (C-5), 140.1 (C-4), 139.9 (C-1), 139.2 (C-1'), 136.9 (C-10), 134.7 (C-4'), 133.5 (C-12), 132.9 (C-2), 129.8 (2C, C-3' and 5'), 128.4 (2C, C-2' and 6'), 122.5 (C-3), 119.3 (C-7), 119.1 (C-11), 118.6 (C-9), 21.5 (CH_3) ppm (Figure S66).

HRMS (ESI) *m/z*: $[M + H]^+$ Calcd for $C_{19}H_{17}N_2O_2^+$ 305.1285 ; Found 305.1293.

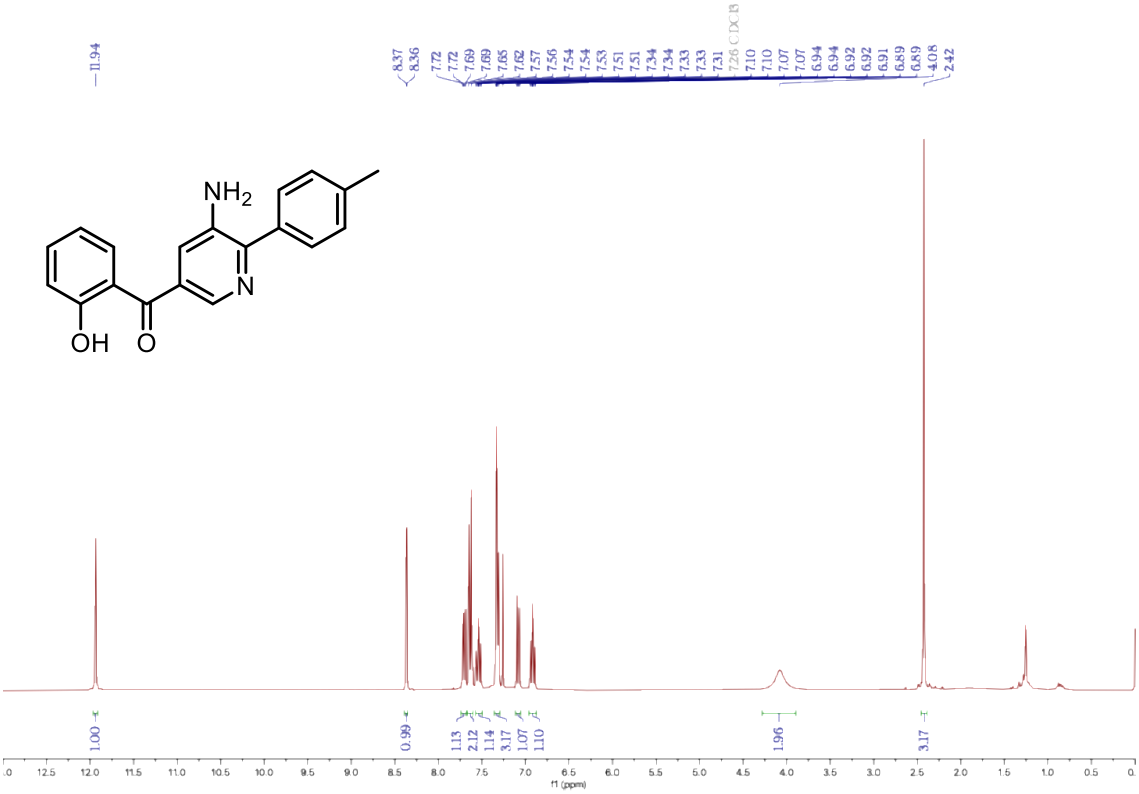

Figure S65 – ^{1}H-NMR (300 MHz, $CDCl_3$) of 3-aminopyridine **3l**.

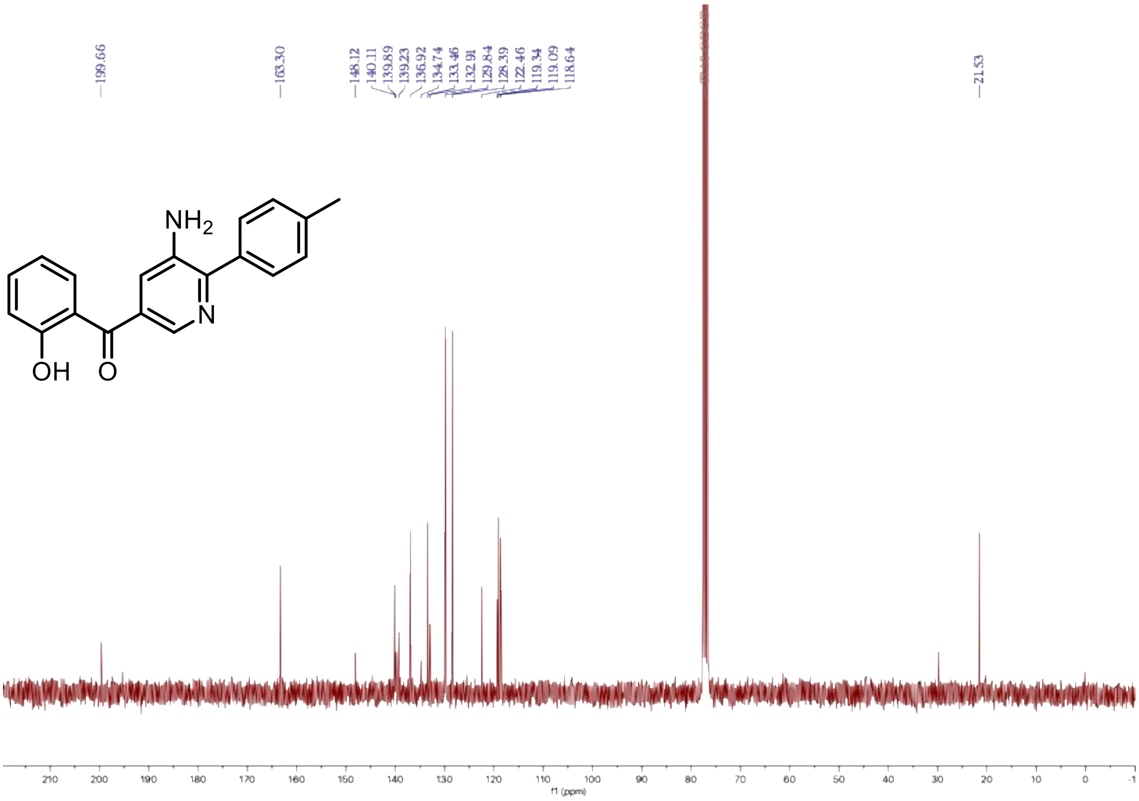

Figure S66 – ^{13}C-NMR (75 MHz, $CDCl_3$) of 3-aminopyridine **3l**.

[5-amino-6-(4-methoxyphenyl)pyridin-3-yl](2-hydroxyphenyl)methanone (3m)

Following the general procedure using 4-oxo-4*H*-chromene-3-carbaldehyde **1a** (80.0 mg) and 1-[2-(4-methoxyphenyl)-2-oxoethyl]pyridin-1-ium bromide **2d** (212.3 mg). After purification by column chromatography using a gradient of 0% to 40% ethyl acetate in hexane, the compound **3m** (110.9 mg, 75% yield) was obtained.

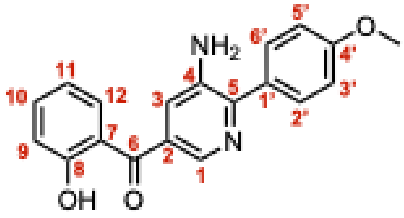

3m

Yellow solid. Melting point of 142.9–145.0 °C.

^{1}H NMR (300 MHz, CDCl$_3$): δ 11.93 (s, 1H, O*H*), 8.33 (d, *J* = 1.9 Hz, H-1, 1H), 7.72 – 7.65 (m, H-12, 2' and 6', 3H), 7.51 (ddd, *J* = 8.7, 7.2, 1.7 Hz, H-10, 1H), 7.28 (d, *J* = 1.9 Hz, H-3, 1H), 7.06 (dd, *J* = 8.4, 1.1 Hz, H-9, 1H), 7.04 – 6.97 (m, H-3' and 5', 2H), 6.89 (ddd, *J* = 8.2, 7.2, 1.2 Hz, H-11, 1H), 4.10 (s, NH_2, 2H), 3.84 (s, OCH_3, 3H) ppm (Figure S67).

^{13}C NMR (75 MHz, CDCl$_3$): δ 199.6 (C-6), 163.2 (C-8), 160.2 (C-4'), 147.8 (C-5), 140.0 (C-4), 139.8 (C-1), 136.8 (C-10), 133.4 (C-12), 132.6 (C-2), 130.0 (C-1'), 129.8 (2C, C-2' and 6'), 122.4 (C-3), 119.3 (C-7), 119.0 (C-11), 118.5 (C-9), 114.4 (2C, C-3' and 5'), 55.4 (O*C*H$_3$) ppm (Figure S68).

HRMS (ESI) *m/z*: [M + H]$^+$ Calcd for $C_{19}H_{17}N_2O_3^+$ 321.1234 ; Found 321.1243.

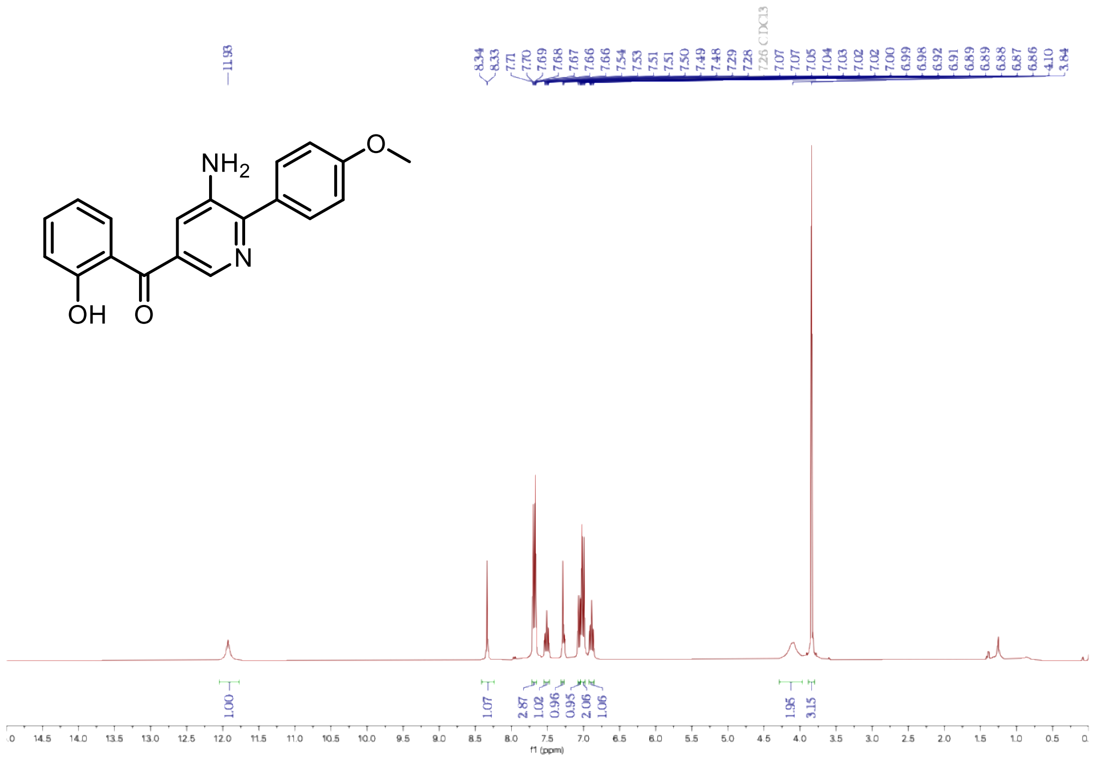

Figure S67 – ^{1}H-NMR (300 MHz, $CDCl_3$) of 3-aminopyridine **3m**.

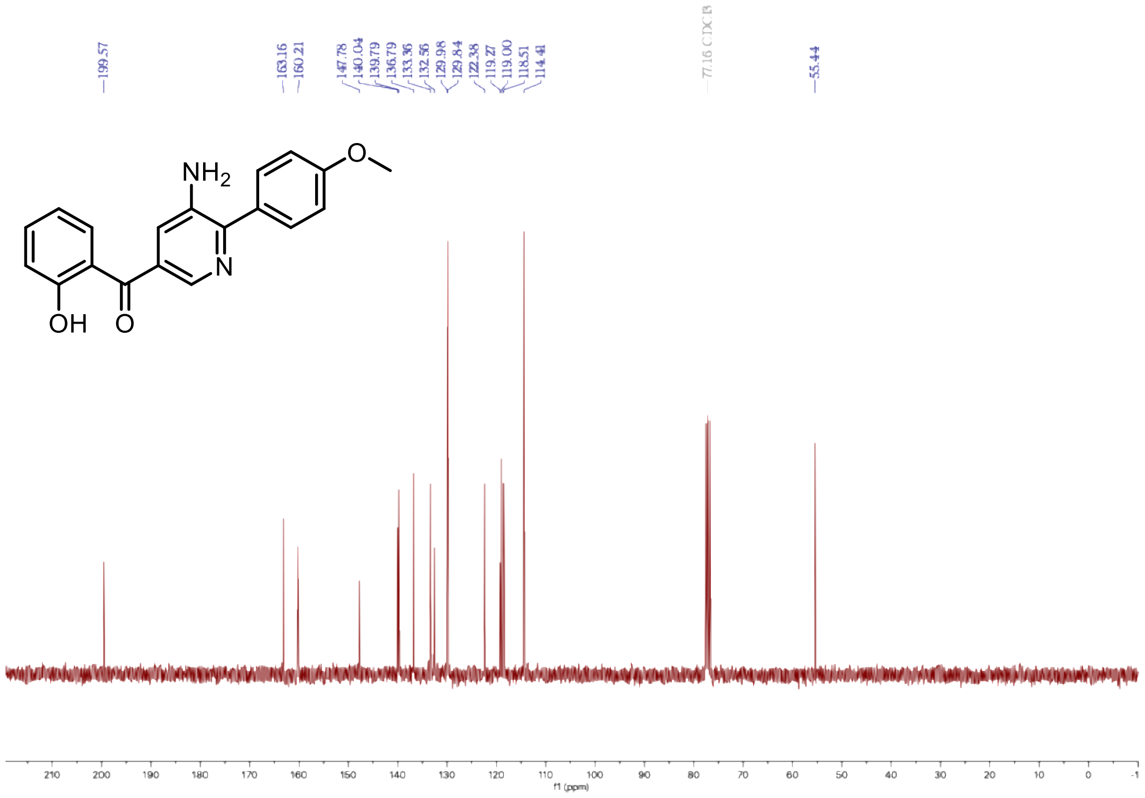

Figure S68 – ^{13}C-NMR (75 MHz, $CDCl_3$) of 3-aminopyridine **3m**.

{5-amino-6-[4-(trifluoromethyl)phenyl]pyridin-3-yl}(2-hydroxyphenyl)methanone (3n)

Following the general procedure using 4-oxo-4*H*-chromene-3-carbaldehyde **1a** (80.0 mg) and 1-{2-oxo-2-[4-(trifluoromethyl)phenyl]ethyl}pyridin-1-ium bromide **2e** (238.5 mg). After purification by column chromatography using a gradient of 0% to 40% ethyl acetate in hexane, the compound **3n** (155.3 mg, 94% yield) was obtained.

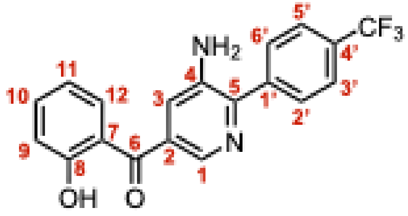

3n

Light yellow solid. Melting point of 156.4–157.7 °C.

^{1}H NMR (300 MHz, $CDCl_3$): δ 11.84 (s, O*H*, 1H), 8.36 (d, *J* = 1.8 Hz, H-1, 1H), 7.90 – 7.85 (m, H-2' and 6', 2H), 7.78 – 7.73 (m, H-3' and 5', 2H), 7.66 (dd, *J* = 8.1, 1.7 Hz, H-12, 1H), 7.52 (ddd, *J* = 8.5, 7.2, 1.7 Hz, H-10, 1H), 7.33 (d, *J* = 1.8 Hz, H-3, 1H), 7.08 – 7.04 (m, H-9, 1H), 6.90 (ddd, *J* = 8.1, 7.2, 1.2 Hz, H-11, 1H), 4.10 (s, NH_2, 2H) ppm (Figure S69).

^{13}C NMR (75 MHz, $CDCl_3$): δ 199.4 (C-6), 163.3 (C-8), 146.0 (C-5), 141.4 (C-1'), 140.3 (C-4), 139.9 (C-1), 137.0 (C-10), 133.8 (C-2), 133.3 (C-12), 131.1 (q, *J* = 32.6 Hz, C-4'), 129.0 (C-2' and 6'), 126.0 (q, *J* = 3.8 Hz, C-3' and 5'), 123.0 (C-3), 122.3 (q, *J* = 272.5 Hz, *C*F_3), 119.3 (C-7), 119.1 (C-11), 118.7 (C-9) ppm (Figure S70).

^{19}F NMR (282 MHz, $CDCl_3$): δ -59.36 ppm (Figure S71).

HRMS (ESI) *m/z*: $[M + H]^+$ Calcd for $C_{19}H_{14}F_3N_2O_2^+$ 359.1002 ; Found 359.1007.

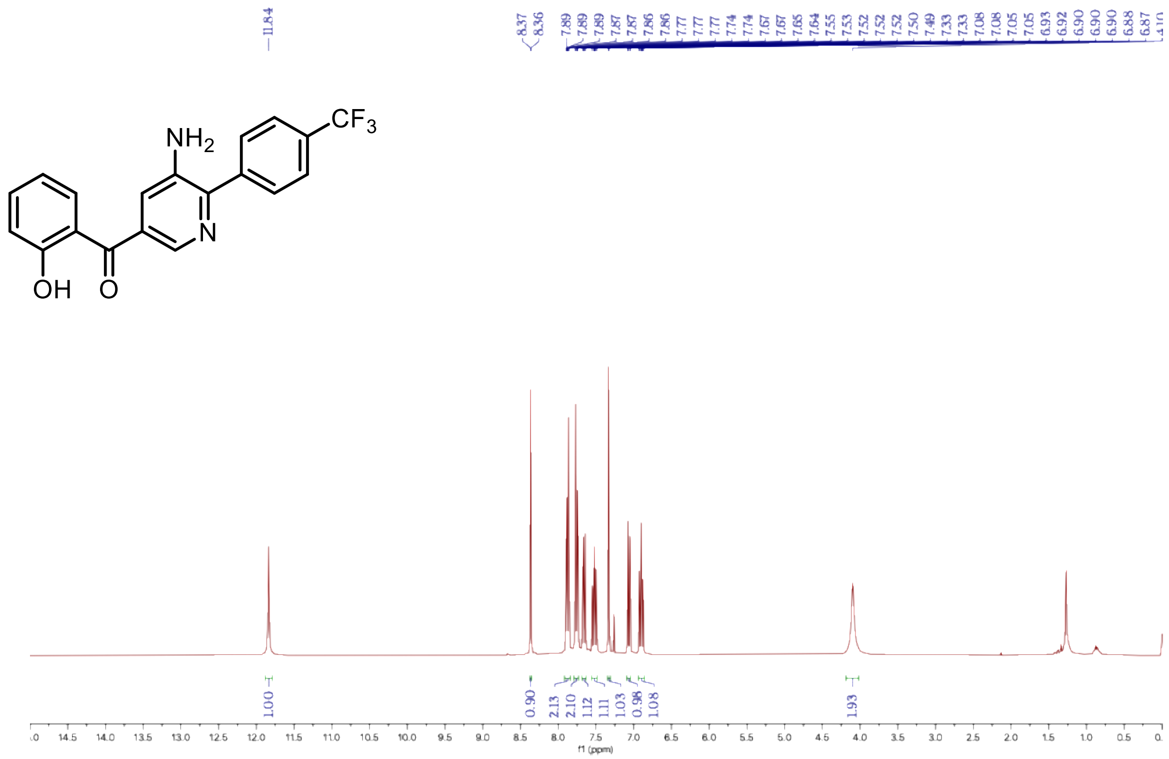

Figure S69 – ^{1}H-NMR (300 MHz, $CDCl_3$) of 3-aminopyridine **3n**.

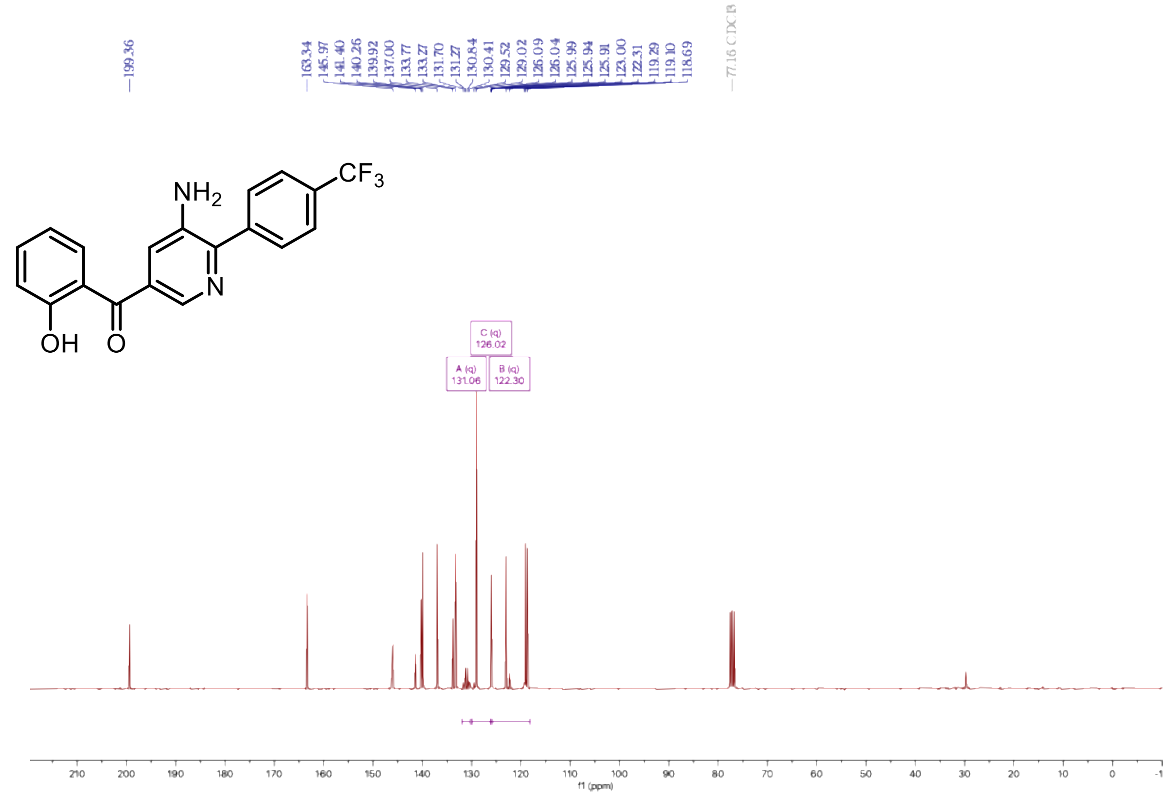

Figure S70 – ^{13}C-NMR (75 MHz, $CDCl_3$) of 3-aminopyridine **3n**.

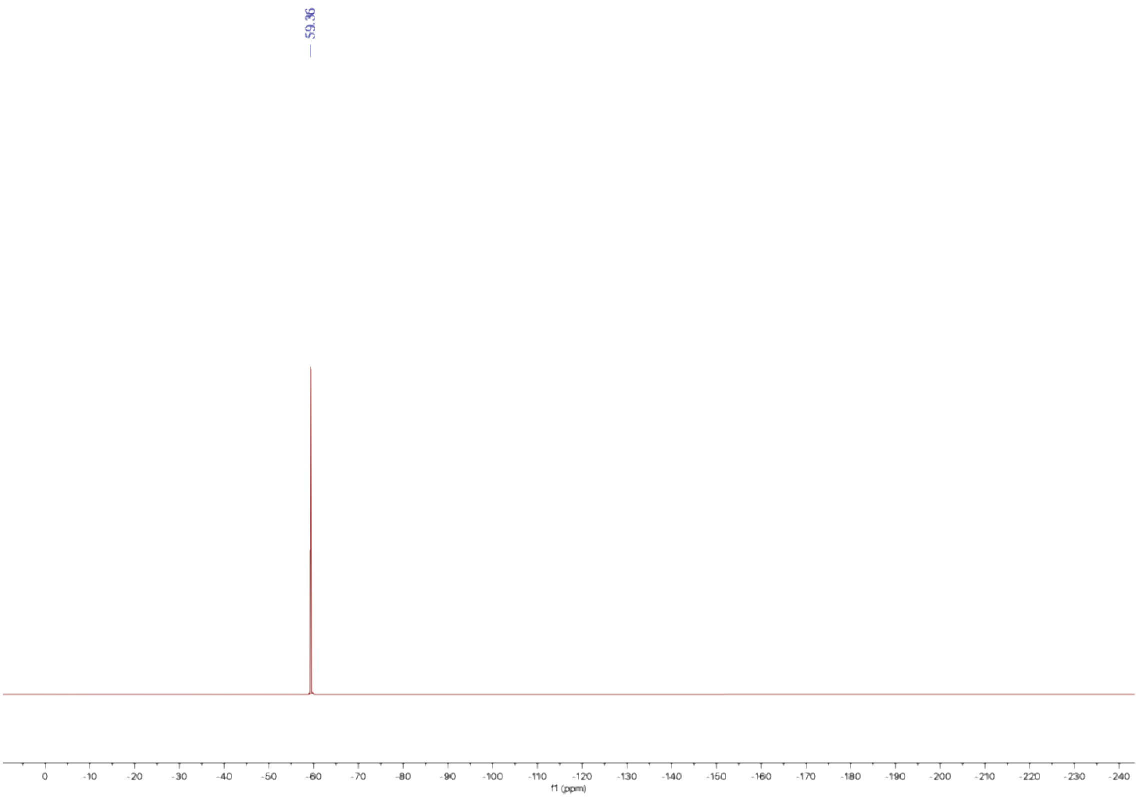

Figure S71 – ^{19}F-NMR (282 MHz, $CDCl_3$) of 3-aminopyridine **3n**.

4-[3-amino-5-(2-hydroxybenzoyl)pyridin-2-yl]benzoic acid (3o)

Following the general procedure using 4-oxo-4*H*-chromene-3-carbaldehyde **1a** (80.0 mg) and 1-[2-(4-carboxyphenyl)-2-oxoethyl]pyridin-1-ium bromide **2f** (228.9 mg), but instead of EtOH, THF was used as solvent. After purification by column chromatography using a gradient of 0% to 40% ethyl acetate in hexane, the compound **3o** (49.6 mg, 32% yield) was obtained.

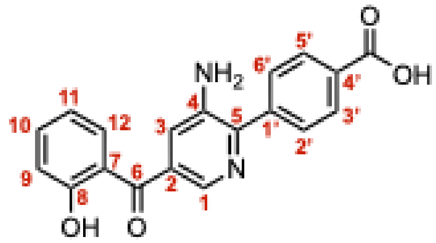

3o Dark yellow solid. Melting point of 236.5–239.2 °C

^{1}H NMR (300 MHz, DMSO-d_6): δ 13.04 (s, COO*H*, 1H), 10.48 (s, O*H*, 1H), 8.17 (d, *J* = 1.8 Hz, H-1, 1H), 8.07 – 8.02 (m, H- 3’ and 5’, 2H), 7.89 – 7.83 (m, H-2’ and 6’, 2H), 7.52 – 7.40 (m, H-1 and H-9, 10, 11 or 12, 3H), 7.03 – 6.94 (m, H-9, 10, 11 or 12, 2H), 5.54 (s, NH_2, 2H) ppm (Figure S72).

^{13}C NMR (75 MHz, DMSO-d_6): δ 196.6 (C-6), 167.1 (*C*OOH), 157.2 (C-8), 144.4 (C-5), 142.6 (C-4), 142.0 (C-1’), 138.1 (C-1), 133.8 (C-10 or 12), 132.8 (C-2), 130.6 (C-10 or 12), 130.2 (C-4’), 129.5 (C-3’ and 5’), 128.5 (C-2’ and 6’), 124.4 (C-7), 122.7 (C-3), 119.2 (C-9 or 11), 116.9 (C-9 or 11) ppm (Figure S73).

HRMS (ESI) *m/z*: $[M + H]^+$ Calcd for $C_{19}H_{15}N_2O_4^+$ 335.1026 ; Found 335.1023.

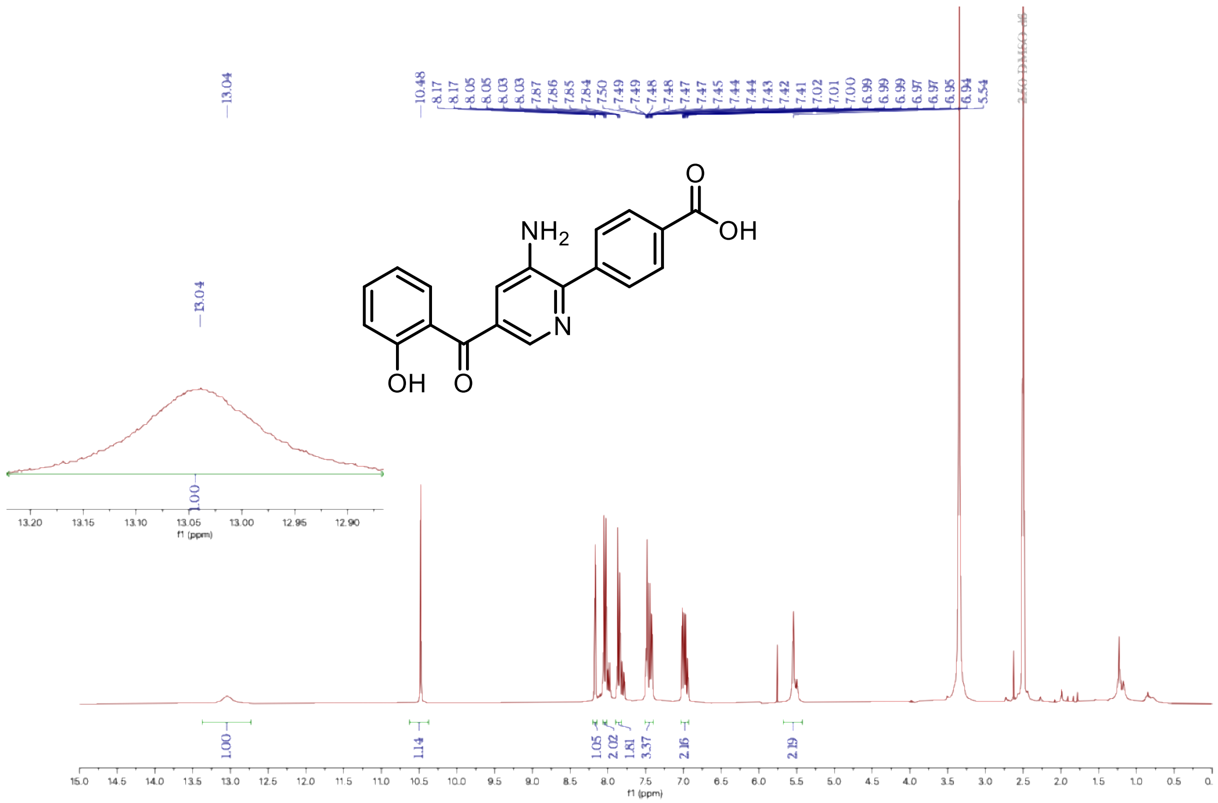

Figure S72 – ^{1}H-NMR (300 MHz, DMSO-d_6) of 3-aminopyridine **3o**.

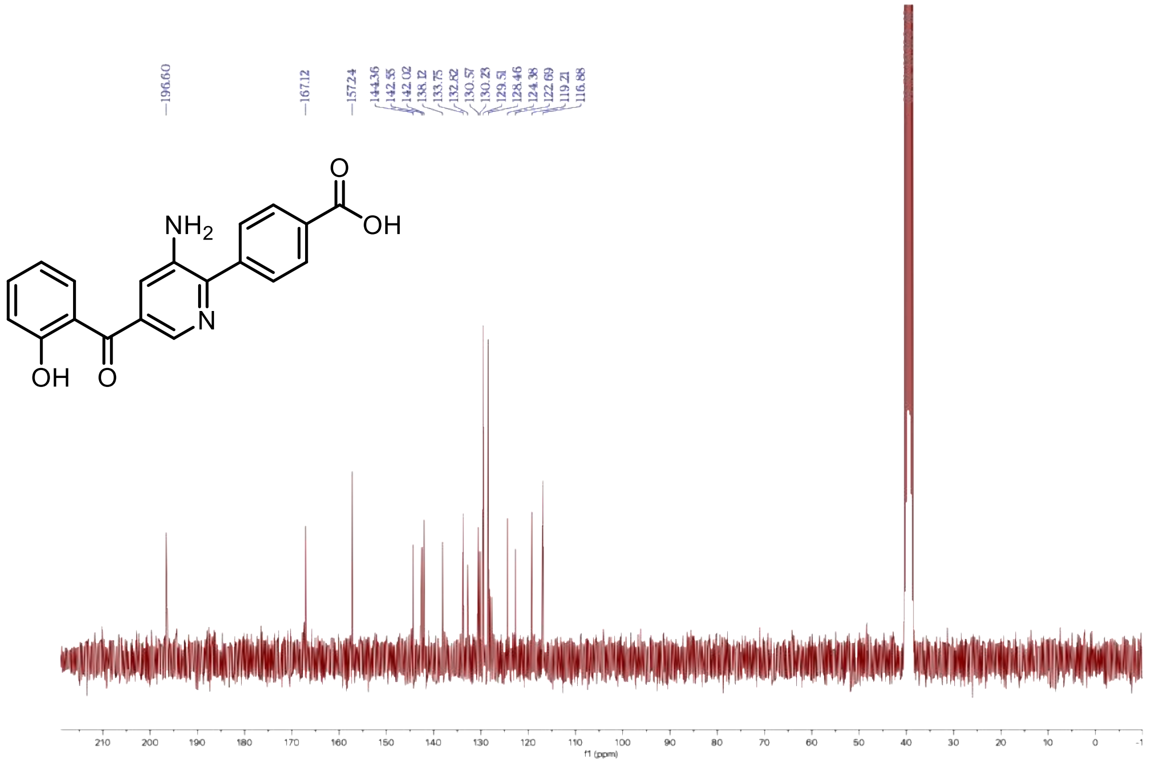

Figure S73 – ^{13}C-NMR (75 MHz, DMSO-d_6) of 3-aminopyridine **3o**.

[5-amino-6-(4-aminophenyl)pyridin-3-yl](2-hydroxyphenyl)methanone (3p)

Following the general procedure using 4-oxo-4*H*-chromene-3-carbaldehyde **1a** (80.0 mg) and 1-[2-(4-aminophenyl)-2-oxoethyl]pyridin-1-ium bromide **2g** (202.0 mg), but instead of NH_4OAc, $(NH_4)_2CO_3$ was used as the nitrogen source and base. After purification by column chromatography using a gradient of 0% to 40% ethyl acetate in hexane, the compound **3p** (67.5 mg, 48% yield) was obtained.

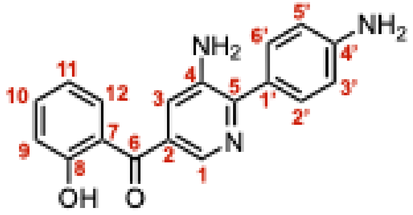

3p

Dark orange solid. Melting point of 142.2–143.8 °C.

^{1}H NMR (300 MHz, DMSO-*d*$_6$): δ 10.47 (s, O*H*, 1H), 8.09 (d, *J* = 2.0 Hz, H-1, 1H), 7.52 – 7.37 (m, H-3, 10, 12, 2' and 6', 5H), 7.10 – 6.87 (m, H-9 and 11, 2H), 6.70 – 6.60 (m, H-3' and 5', 2H), 5.98 – 4.94 (m, 4-N*H*$_2$ and 4'-N*H*$_2$, 4H) ppm (Figure S74).

^{13}C NMR (75 MHz, DMSO-*d*$_6$): δ 196.5 (C-6), 157.2 (C-8), 149.3 (C-4'), 147.0 (C-5), 141.1 (C-4), 138.2 (C-1), 133.5 (C-12), 131.0 (C-2), 130.5 (C-10), 129.3 (C-2' and 6', 2C), 125.0 (C-7), 124.6 (C-1'), 121.7 (C-3), 119.1 (C-11), 116.8 (C-9), 113.5 (C-3' and 5', 2C) ppm (Figure S75).

HRMS (ESI) *m/z*: $[M + H]^+$ Calcd for $C_{18}H_{16}N_3O_2^+$ 306.1237 ; Found 306.1245.

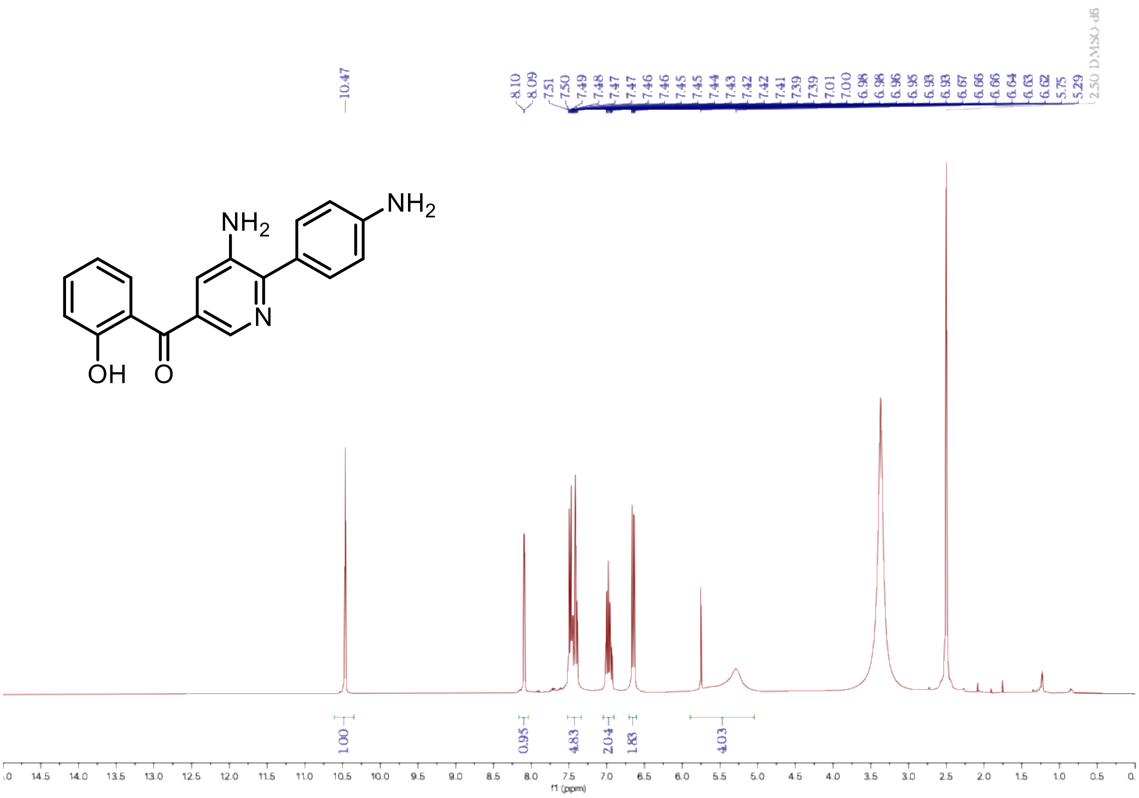

Figure S74 – ^{1}H-NMR (300 MHz, DMSO-d_6) of 3-aminopyridine **3p**.

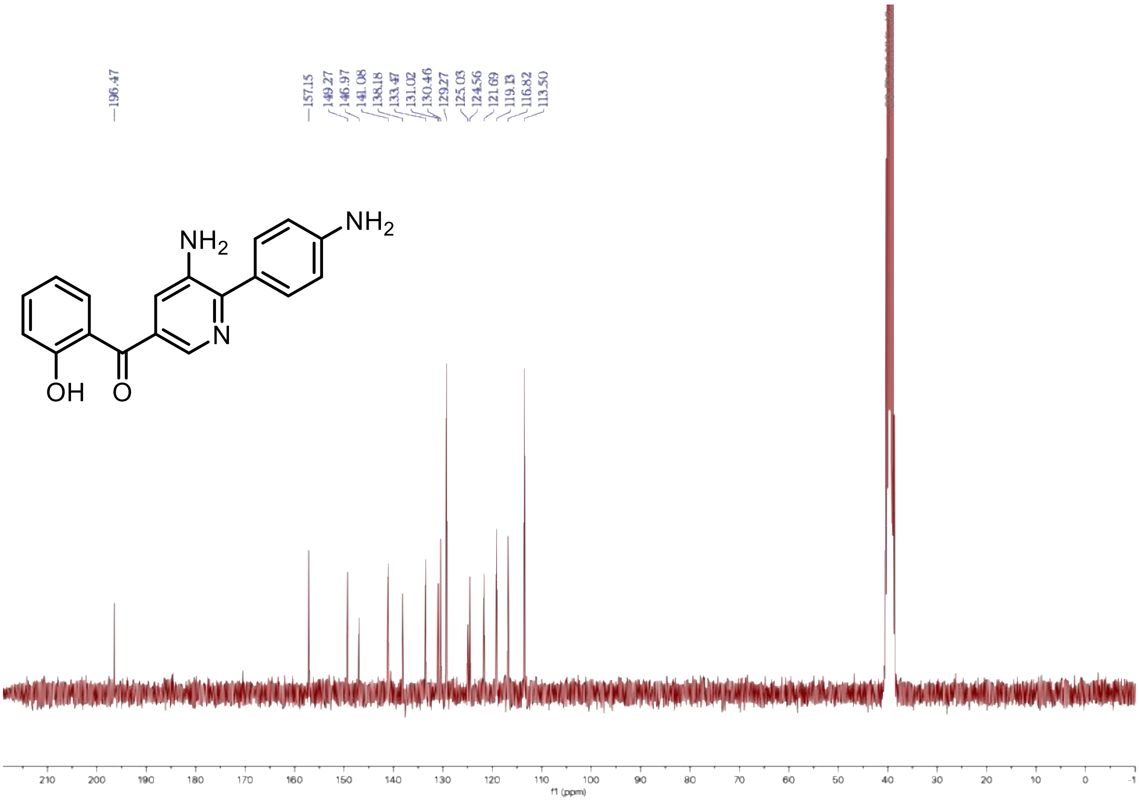

Figure S75 – ^{13}C-NMR (75 MHz, DMSO-d_6) of 3-aminopyridine **3p**.

4-[3-amino-5-(2-hydroxybenzoyl)pyridin-2-yl]benzonitrile (3q)

Following the general procedure using 4-oxo-4*H*-chromene-3-carbaldehyde **1a** (80.0 mg) and 1-[2-(4-cyanophenyl)-2-oxoethyl]pyridin-1-ium bromide **2h** (208.9 mg). After purification by column chromatography using a gradient of 0% to 40% ethyl acetate in hexane, the compound **3q** (82.0 mg, 57% yield) was obtained.

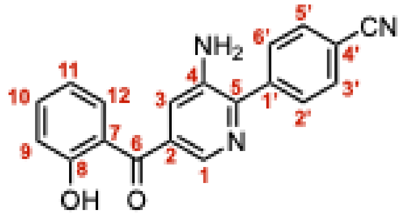

3q

Yellow solid. Melting point of 178.6–180.2 °C.

^{1}H NMR (300 MHz, $CDCl_3$): δ 11.86 (s, 1H), 8.38 (d, *J* = 1.8 Hz, H-1, 1H), 7.95 – 7.89 (m, H-2' and 6',2H), 7.83 – 7.77 (m, H-3' and 5', 2H), 7.66 (dd, *J* = 8.0, 1.7 Hz, H-12, 1H), 7.55 (ddd, *J* = 8.7, 7.2, 1.7 Hz, H-10, 1H), 7.38 (d, *J* = 1.9 Hz, H-3, 1H), 7.09 (dd, *J* = 8.5, 1.1 Hz, H-9, 1H), 6.92 (ddd, *J* = 8.2, 7.2, 1.1 Hz, H-11, 1H), 4.10 (s, ,N*H*$_2$, 2H) ppm (Figure S76).

^{13}C NMR (75 MHz, $CDCl_3$): δ 199.2 (C-6), 163.4 (C-8), 145.1 (C-5), 142.3 (C-1'), 140.3 (C-4), 140.1 (C-1), 137.2 (C-10), 134.0 (C-2), 133.3 (C-12), 132.9 (2C, C-3' and 5'), 129.4 (2C, C-2' and 6'), 123.4 (2C, C-3 and 7), 119.2 (C-11), 118.8 (C-9 and *C*N), 112.7 (C-4') ppm (Figure S77).

HRMS (ESI) *m/z*: [M + H]$^+$ Calcd for $C_{19}H_{14}N_3O_2^+$ 316.1081 ; Found 316.1087.

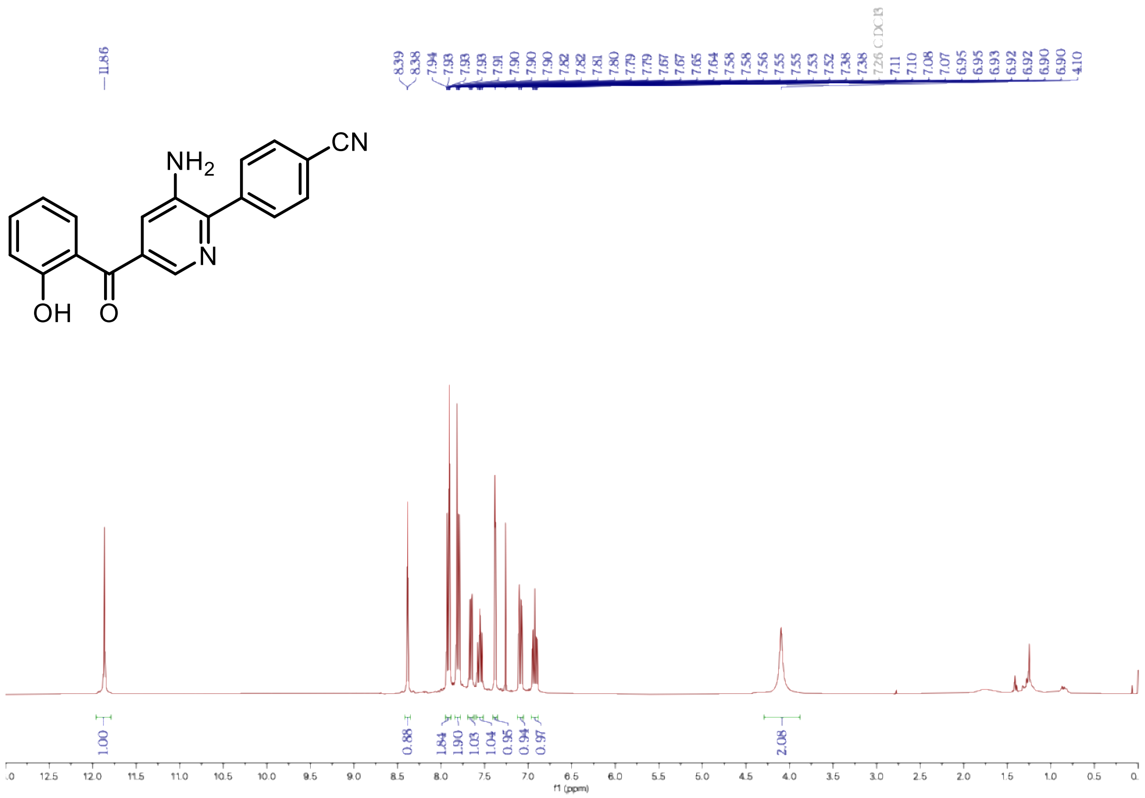

Figure S76 – ^{1}H-NMR (300 MHz, $CDCl_3$) of 3-aminopyridine **3q**.

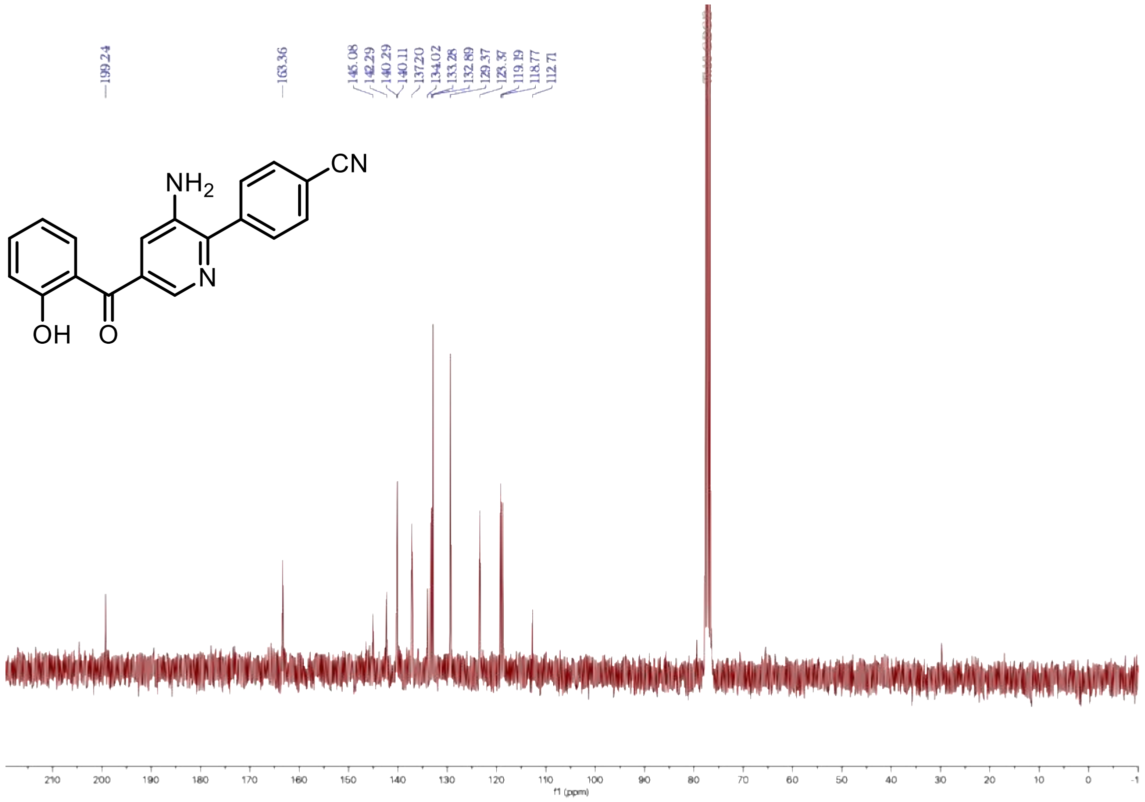

Figure S77 – ^{13}C-NMR (75 MHz, $CDCl_3$) of 3-aminopyridine **3q**.

[5-amino-6-(4-nitrophenyl)pyridin-3-yl](2-hydroxyphenyl)methanone (3r)

Following the general procedure using 4-oxo-4*H*-chromene-3-carbaldehyde **1a** (80.0 mg) and 1-[2-(4-nitrophenyl)-2-oxoethyl]pyridin-1-ium bromide **2i** (222.7 mg). After purification by column chromatography using a gradient of 0% to 40% ethyl acetate in hexane, the compound **3r** (121.9 mg, 79% yield) was obtained.

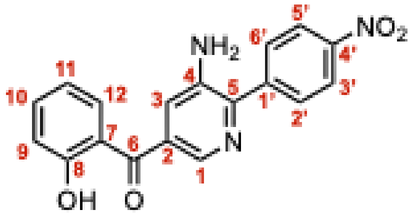

3r

Yellow solid. Melting point of 191.7–193.7 °C.

^{1}H NMR (300 MHz, DMSO-*d$_6$*): δ 10.50 (s, O*H*, 1H), 8.38 – 8.28 (m, H-3' and 5', 2H), 8.19 (d, *J* = 1.9 Hz, H-1, 1H), 8.10 – 7.98 (m, H-2' and 6', 2H), 7.51 (d, *J* = 1.9 Hz, H-3, 1H), 7.50 – 7.41 (m, H-10 and 12, 2H), 7.03 – 6.94 (m, H-9 and 11, 2H), 5.68 (s, N*H$_2$*, 2H) ppm (Figure S78).

^{13}C NMR (75 MHz, DMSO-*d$_6$*): δ 196.5 (C-6), 157.3 (C-8), 146.8 (C-4), 145.0 (C-5), 142.8 (C-4 or 1'), 142.4 (C-4 or 1'), 138.2 (C-1), 133.9 (C-10), 133.4 (C-2), 130.6 (C-12), 129.7 (C-2' and 6'), 124.3 (C-7), 123.7 (C-3' and 5'), 123.3 (C-3), 119.3 (C-11), 116.9 (C-9) ppm (Figure S79).

HRMS (ESI) *m/z*: $[M + H]^+$ Calcd for $C_{18}H_{14}N_3O_4^+$ 336.0979 ; Found 336.0984.

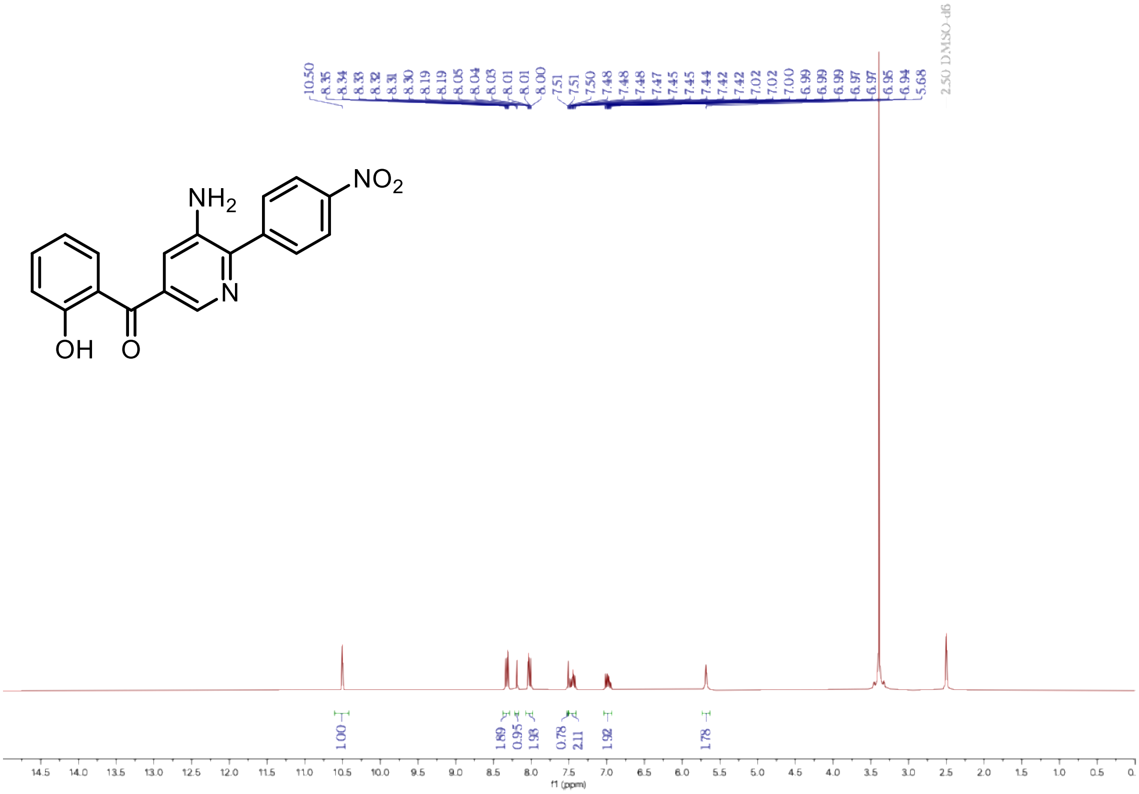

Figure S78 – ^{1}H-NMR (300 MHz, DMSO-d_6) of 3-aminopyridine **3r**.

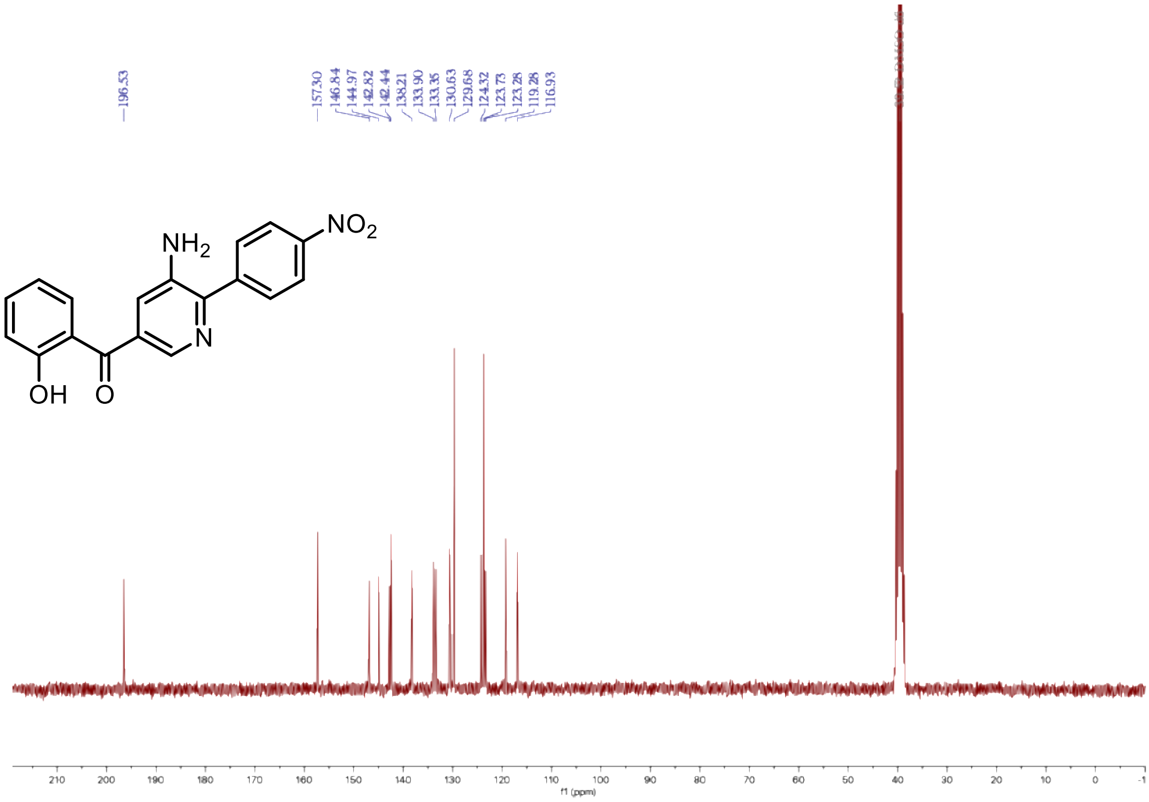

Figure S79 – ^{13}C-NMR (75 MHz, DMSO-d_6) of 3-aminopyridine **3r**.

[5-amino-6-(2-bromophenyl)pyridin-3-yl](2-hydroxyphenyl)methanone (3s)

Following the general procedure using 4-oxo-4*H*-chromene-3-carbaldehyde **1a** (80.0 mg) and 1-[2-(2-bromophenyl)-2-oxoethyl]pyridin-1-ium bromide **2j** (246.0 mg). After purification by column chromatography using a gradient of 0% to 40% ethyl acetate in hexane, compound **3s** (105.2 mg, 62% yield) was obtained.

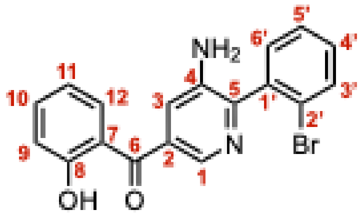

3s

Dark yellow solid. Melting point of 129.0–130.7 °C.

^{1}H NMR (300 MHz, $CDCl_3$): δ 11.93 (s, O*H*, 1H), 8.34 (d, *J* = 1.9 Hz, H-1, 1H), 7.75 – 7.66 (m, H-12 and 6', 2H), 7.55 (ddd, *J* = 8.5, 7.2, 1.7 Hz, H-10, 1H), 7.52 – 7.37 (m, H-4' and 5', 2H), 7.38 – 7.26 (m, H-3 and 3', 2H), 7.09 (dd, *J* = 8.5, 1.2 Hz, H-9, 1H), 6.93 (ddd, *J* = 8.5, 7.2, 1.2 Hz, H-11, 1H), 3.97 (s, NH_2, 2H) ppm (Figure S80).

^{13}C NMR (75 MHz, $CDCl_3$): δ 199.5 (C-6), 163.1 (C-8), 147.6 (C-5), 140.4 (C-4), 138.9 (C-1), 138.1 (C-1'), 136.9 (C-10), 133.8 (C-2), 133.4 (C-12 and 6', 2C), 131.0 (C-4'), 130.6 (C-3'), 128.2 (C-5'), 122.6 (C-2'), 122.1 (C-3), 119.1 (C-7), 119.1 (C-11), 118.5 (C-9) ppm (Figure S81).

HRMS (ESI) ***m/z*:** $[M + H]^+$ Calcd for $C_{18}H_{14}BrN_2O_2^+$ 369.0233 ; Found 369.0240.

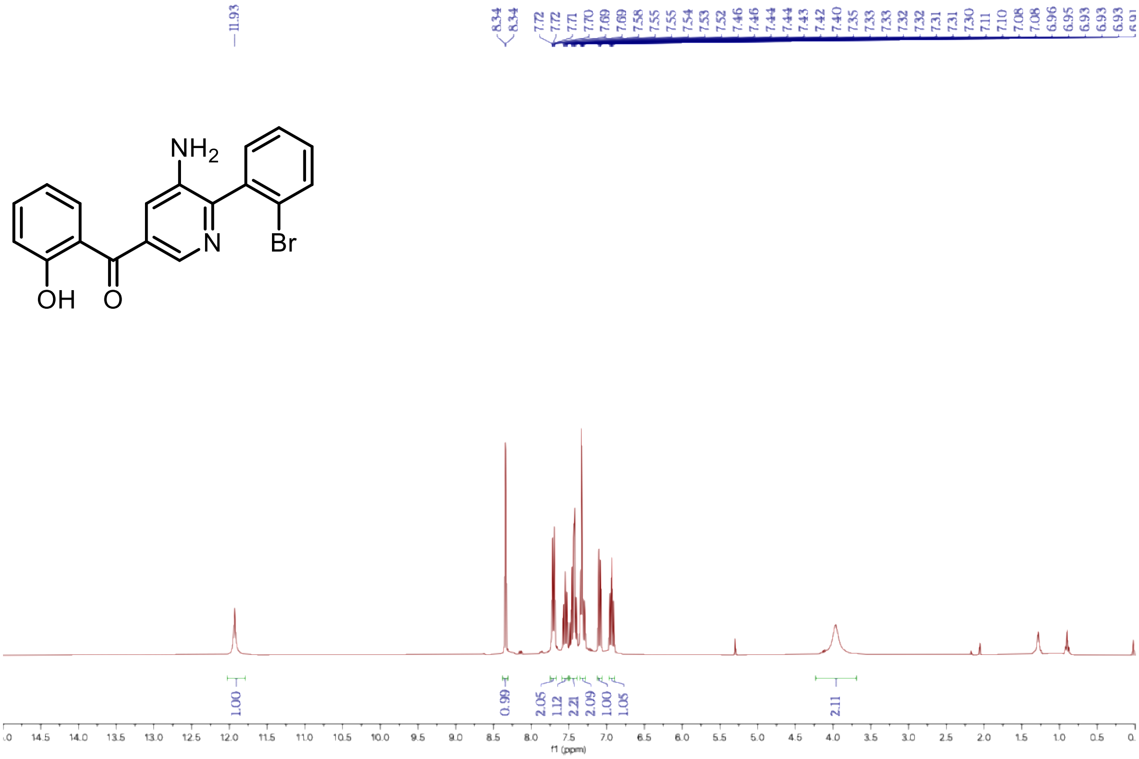

Figure S80 – ^{1}H-NMR (300 MHz, $CDCl_3$) of 3-aminopyridine **3s**.

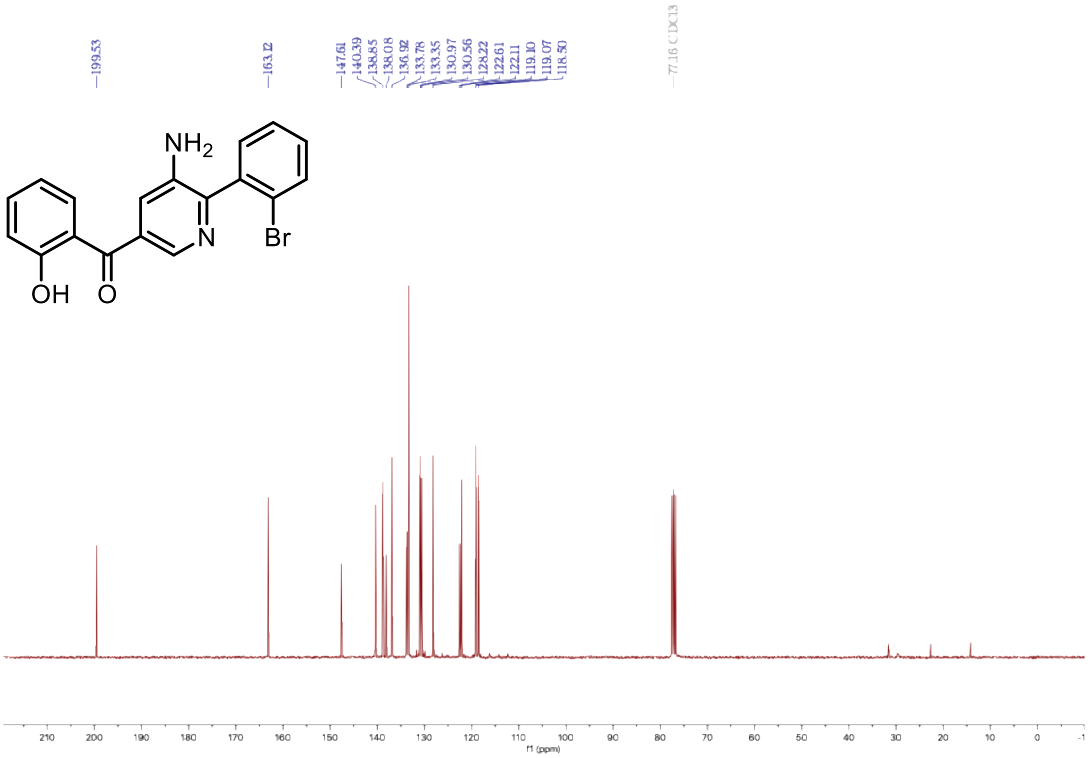

Figure S81 – ^{13}C-NMR (75 MHz, $CDCl_3$) of 3-aminopyridine **3s**.

[5-amino-6-(naphthalen-2-yl)pyridin-3-yl](2-hydroxyphenyl)methanone (3t)

Following the general procedure using 4-oxo-4*H*-chromene-3-carbaldehyde **1a** (80.0 mg) and 1-[2-(naphthalen-2-yl)-2-oxoethyl]pyridin-1-ium bromide **2k** (226.1 mg). After purification by column chromatography using a gradient of 0% to 40% ethyl acetate in hexane, compound **3t** (115.7 mg, 74% yield) was obtained.

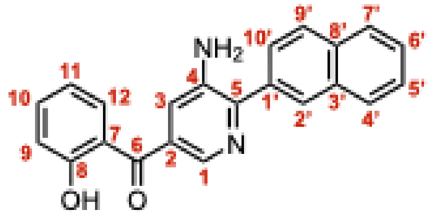

3t

Yellow solid. Melting point of 174.4–176.2 °C.

^{1}H NMR (300 MHz, $CDCl_3$): δ 11.94 (s, O*H*, 1H), 8.43 (d, *J* = 1.9 Hz, H-1, 1H), 8.25 (d, *J* = 1.7 Hz, H-2', 1H), 8.00 (d, *J* = 8.5 Hz, H-9', 1H), 7.95 – 7.88 (m, H-4' and 7', 2H), 7.85 (dd, *J* = 8.5, 1.7 Hz, H-10', 1H), 7.73 (dd, *J* = 8.2, 1.7 Hz, H-12, 1H), 7.60 – 7.51 (m, H-10, 5' and 6', 3H), 7.39 (d, *J* = 1.9 Hz, H-3, 1H), 7.10 (dd, *J* = 8.4, 1.2 Hz, H-9, 1H), 6.94 (ddd, *J* = 8.2, 7.2, 1.2 Hz, H-11, 1H), 4.16 (s, NH_2, 2H) ppm (Figure S82).

^{13}C NMR (75 MHz, $CDCl_3$): δ 199.5 (C-6), 163.2 (C-8), 147.6 (C-5), 140.3 (C-4), 139.9 (C-1), 136.9 (C-10), 134.8 (C-1'), 133.4 (C-3'), 133.3 (C-12), 133.3 (C-8'), 133.0 (C-2), 129.0 (C-9'), 128.4 (C-4'), 127.8 (C-7'), 127.8 (C-2'), 126.9 (C-5' and 6'), 126.6 (C-5' and 6'), 126.0 (C-10'), 122.6 (C-3), 119.2 (C-7), 119.0 (C-11), 118.6 (C-9) ppm (Figure S83).

HRMS (ESI) *m/z*: $[M + H]^+$ Calcd for $C_{22}H_{17}N_2O_2^+$ 341.1285 ; Found 341.1292.

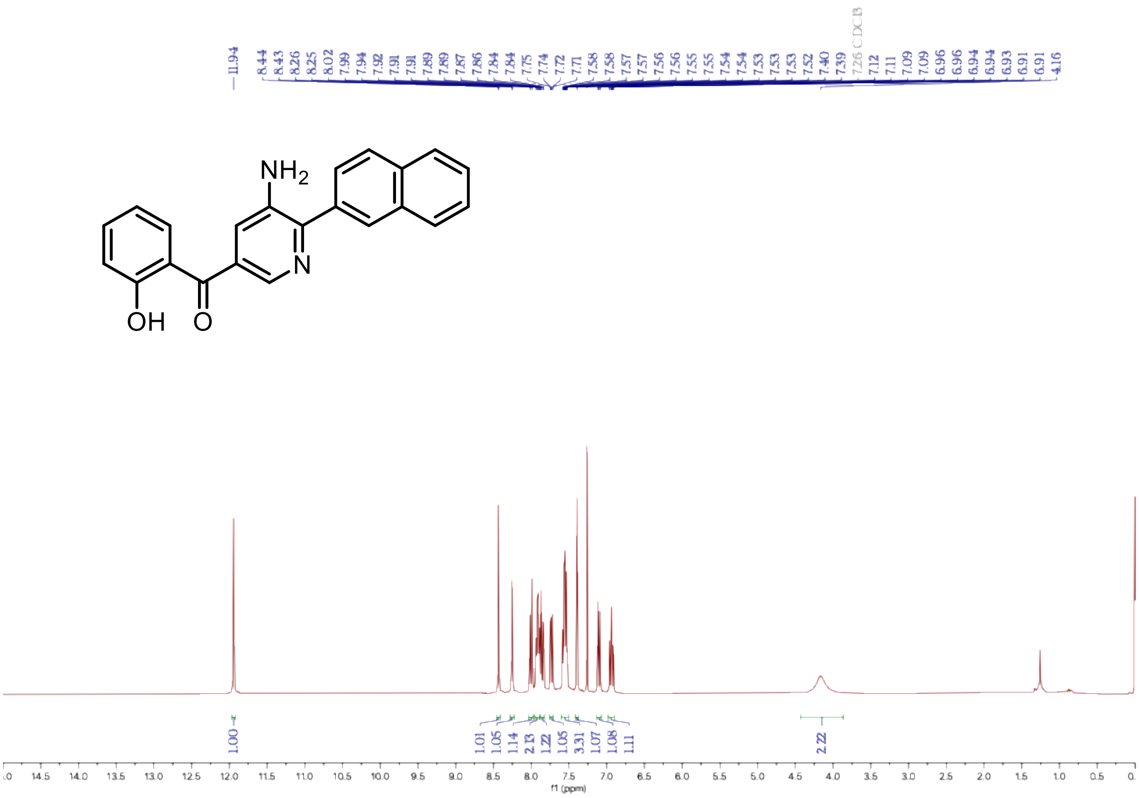

Figure S82 – ^{1}H-NMR (300 MHz, $CDCl_3$) of 3-aminopyridine **3t**.

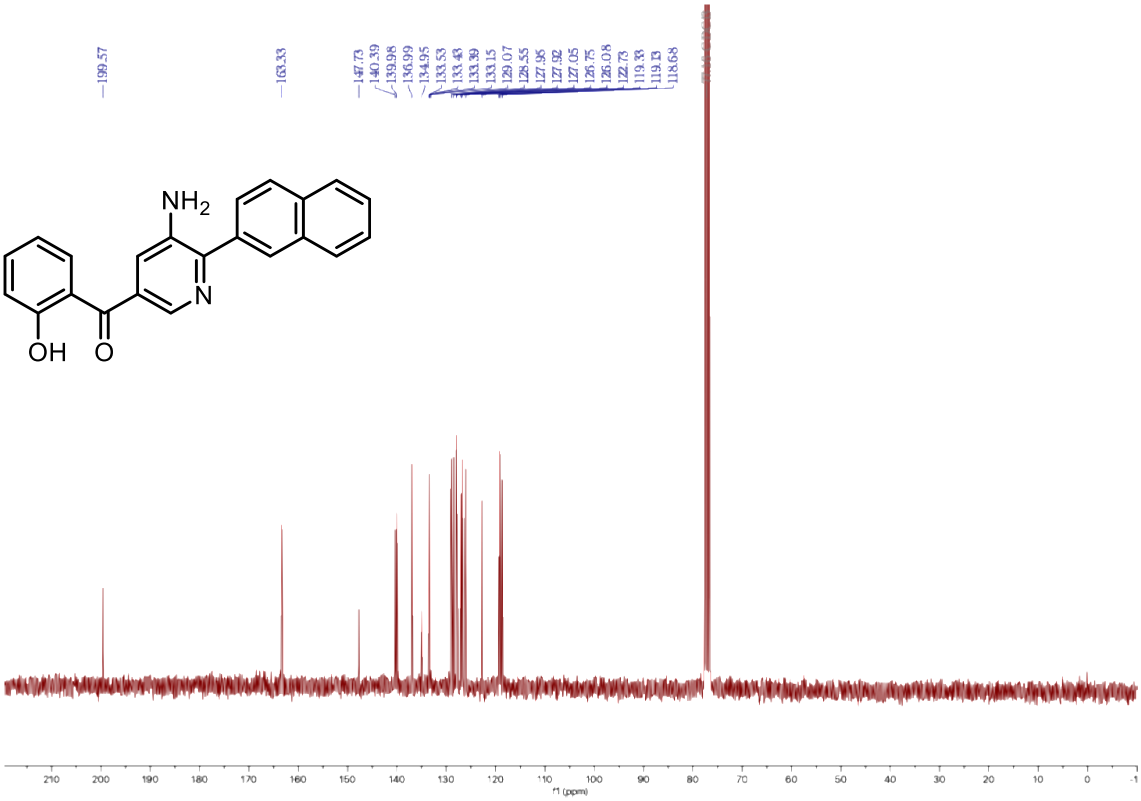

Figure S83 – ^{13}C-NMR (75 MHz, $CDCl_3$) of 3-aminopyridine **3t**.

[5-amino-6-(pyren-1-yl)pyridin-3-yl](2-hydroxyphenyl)methanone (3u)

Following the general procedure using 4-oxo-4*H*-chromene-3-carbaldehyde **1a** (80.0 mg) and 1-[2-oxo-2-(pyren-1-yl)ethyl]pyridin-1-ium bromide **2l** (277.2 mg). After purification by column chromatography using a gradient of 0% to 40% ethyl acetate in hexane, compound **3u** (155.1 mg, 82% yield) was obtained.

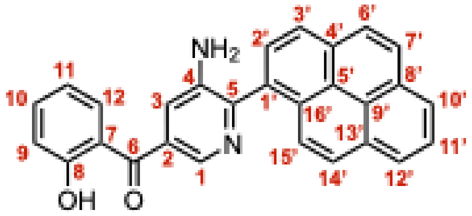

3u

Yellow solid. Melting point of 150.2–152.7 °C.

^{1}H NMR (300 MHz, $CDCl_3$): δ 12.03 (s, O*H*, 1H), 8.43 (m, H-1, 1H), 8.23 – 8.10 (m, H-Ar, 3H), 8.09 – 7.95 (m, H-Ar, 4H), 7.82 (d, *J* = 9.2 Hz, H-Ar, 1H), 7.71 (d, *J* = 7.9 Hz, H-Ar, 1H), 7.51 (t, *J* = 7.8 Hz, H-Ar, 1H), 7.22 (m, H-3, 1H), 7.10 (d, *J* = 8.4 Hz, H-Ar, 1H), 6.89 (t, *J* = 7.6 Hz, H-Ar, 1H), 3.84 (s, NH_2, 2H) ppm (Figure S84).

^{13}C NMR (75 MHz, $CDCl_3$): δ 199.5 (C-6), 163.1 (C-8), 147.7 (C-5), 141.2 (C-4), 139.2 (C-1), 136.8 (C-Ar), 133.3 (C-Ar), 133.3 (C-Ar), 131.7 (C-Ar), 131.5 (C-Ar), 131.1 (C-Ar), 130.7 (C-Ar), 128.4 (C-Ar), 128.3 (C-Ar), 128.1 (C-Ar), 127.2 (C-Ar), 126.9 (C-Ar), 126.2 (C-Ar), 125.6 (C-Ar), 125.5 (C-Ar), 125.2 (C-Ar), 124.9 (C-Ar), 124.5 (C-Ar), 124.4 (C-Ar), 121.8 (C-Ar), 119.1 (C-Ar), 119.0 (C-Ar), 118.4 (C-Ar) ppm (Figure S85).

HRMS (ESI) *m/z*: $[M + H]^+$ Calcd for $C_{28}H_{19}N_2O_2^+$ 415.1441 ; Found 415.1452.

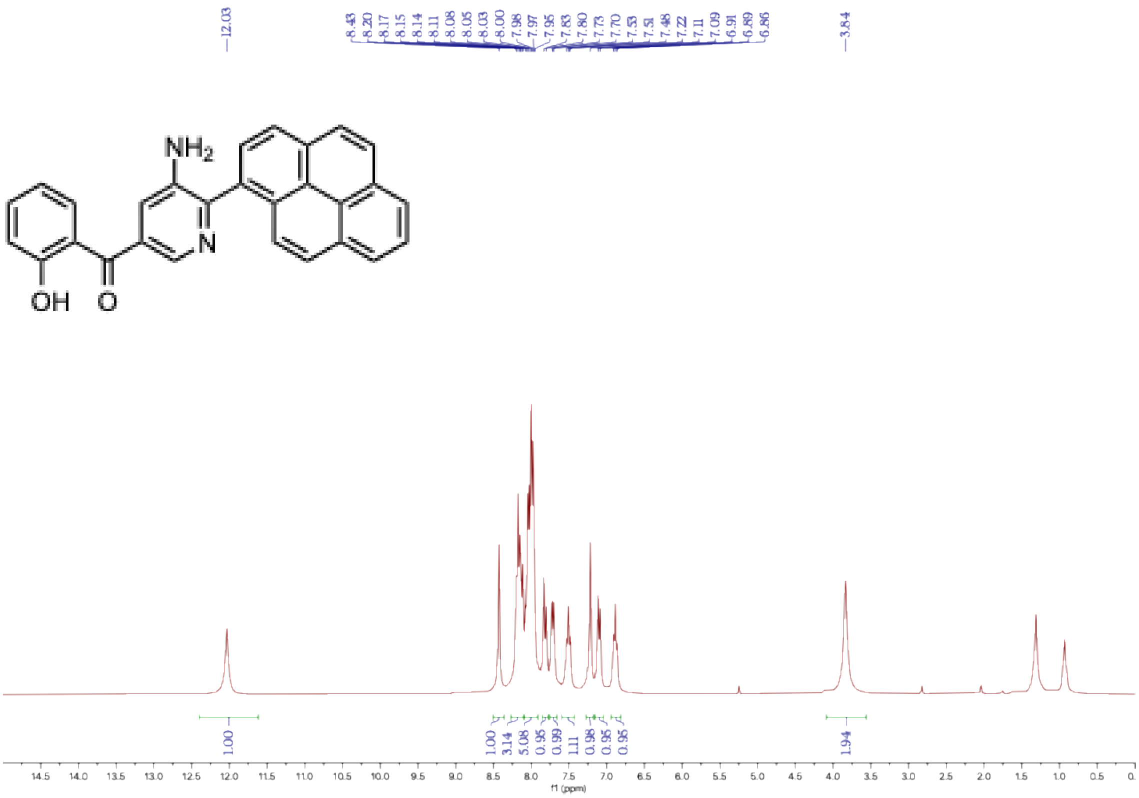

Figure S84 – ^{1}H-NMR (300 MHz, $CDCl_3$) of 3-aminopyridine **3u**.

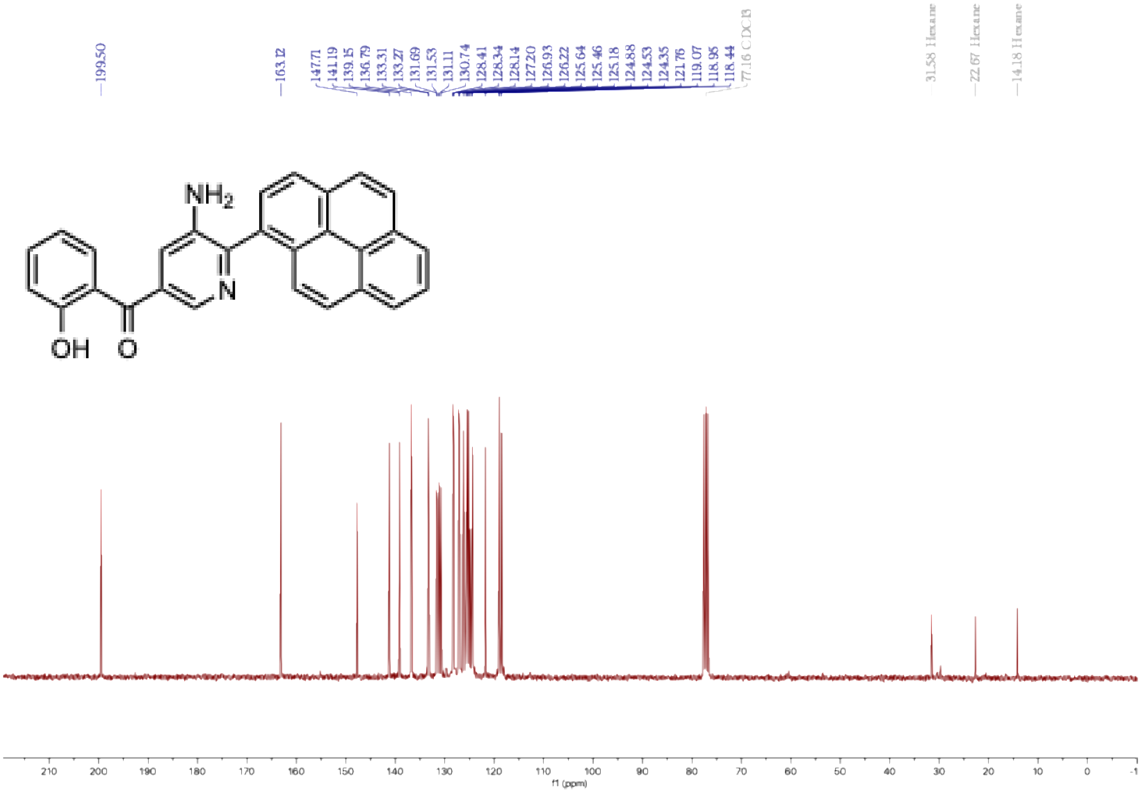

Figure S85 – ^{13}C-NMR (75 MHz, $CDCl_3$) of 3-aminopyridine **3u**.

[5-amino-6-(2,3-dihydrobenzo[*b*][1,4]dioxin-6-yl)pyridin-3-yl](2-hydroxyphenyl)methanone (3v)

Following the general procedure using 4-oxo-4*H*-chromene-3-carbaldehyde **1a** (80.0 mg) and 1-[2-(2,3-dihydrobenzo[*b*][1,4]dioxin-6-yl)-2-oxoethyl]pyridin-1-ium chloride **2m** (201.0 mg). After purification by column chromatography using a gradient of 0% to 40% ethyl acetate in hexane, compound **3v** (105.0 mg, 66% yield) was obtained.

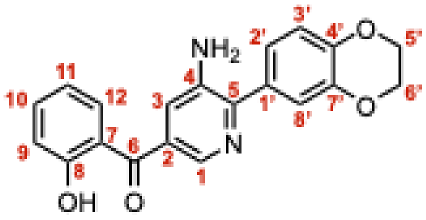

3v

Light yellow solid. Melting point of 169.4–170.7 °C.

^{1}H NMR (300 MHz, DMSO-*d*$_6$): δ 10.48 (s, O*H*, 1H), 8.11 (d, *J* = 1.9 Hz, H-1, 1H), 7.50 – 7.39 (m, Ar-H, 3H), 7.25 – 7.21 (m, Ar-H, 2H), 7.02 – 6.93 (m, Ar-H, 3H), 5.35 (s, N*H*$_2$, 2H), 4.28 (s, H-5 and 6, 4H) ppm (Figure S86).

^{13}C NMR (75 MHz, DMSO-*d*$_6$): δ 196.7 (C-6), 157.2 (C-8), 145.5 (Ar-C), 143.7 (Ar-C), 143.2 (Ar-C), 141.4 (Ar-C), 138.2 (C-1), 133.6 (Ar-C), 132.0 (Ar-C), 131.4 (Ar-C), 130.5 (Ar-C), 124.4 (Ar-C), 122.0 (Ar-C), 121.4 (Ar-C), 119.2 (Ar-C), 117.0 (Ar-C), 117.0 (Ar-C), 116.8 (Ar-C), 64.3 (C-5' or 6'), 64.0 (C-5' or 6') ppm (Figure S87).

HRMS (ESI) *m/z*: $[M + H]^+$ Calcd for $C_{20}H_{17}N_2O_4^+$ 349.1183 ; Found 349.1192.

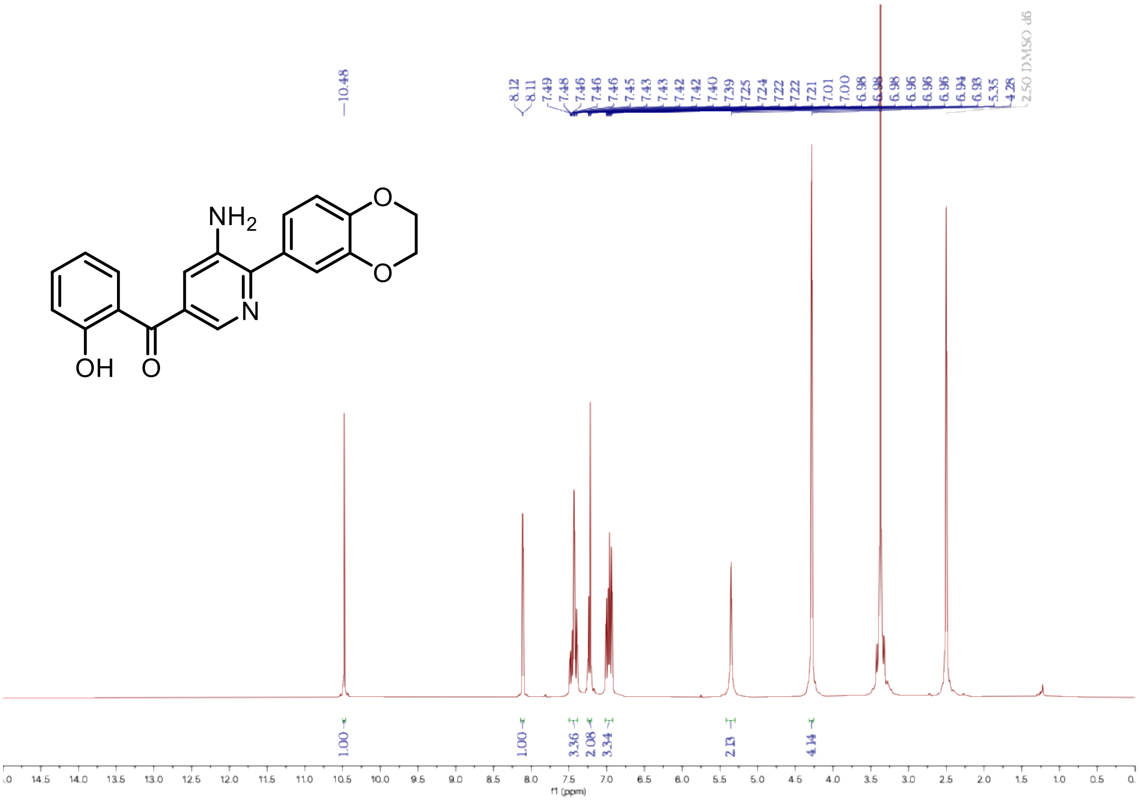

Figure S86 – ^{1}H-NMR (300 MHz, DMSO-d_6) of 3-aminopyridine **3v**.

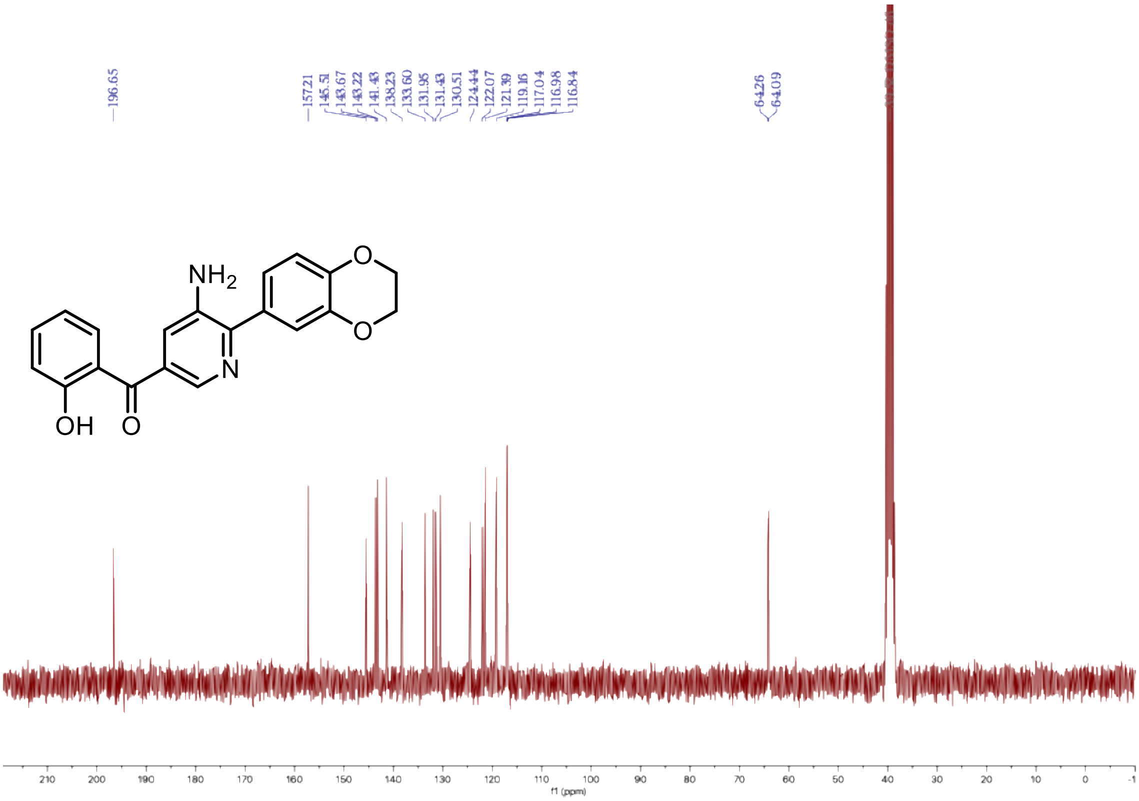

Figure S87 – ^{13}C-NMR (75 MHz, DMSO-d_6) of 3-aminopyridine **3v**.

(3-amino-[2,4'-bipyridin]-5-yl)(2-hydroxyphenyl)methanone (3w)

Following the general procedure using 4-oxo-4*H*-chromene-3-carbaldehyde **1a** (80.0 mg) and 1-[2-oxo-2-(pyridin-4-yl)ethyl]yridin-1-ium bromide **2n** (192.3 mg). After purification by column chromatography using a gradient of 0% to 40% ethyl acetate in hexane, the compound **3w** (43.0 mg, 32% yield) was obtained.

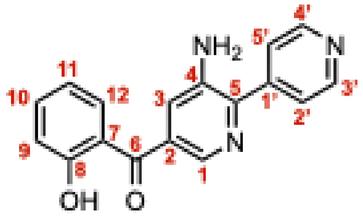

3w Orange solid. Melting point of 118.7–121.2 °C.

^{1}H NMR (500 MHz, CDCl$_3$): δ 11.87 (s, O*H*, 1H), 8.81 – 8.72 (m, H-3' and 4', 2H), 8.39 (d, *J* = 1.8 Hz, H-1, 1H), 7.72 – 7.69 (m, H-2' and 5', 2H), 7.66 (dd, *J* = 8.1, 1.7 Hz, H-12, 1H), 7.55 (ddd, *J* = 8.6, 7.2, 1.7 Hz, H-10, 1H), 7.37 (d, *J* = 1.8 Hz, H-3, 1H), 7.09 (dd, *J* = 8.6, 1.1 Hz, H-9, 1H), 6.92 (ddd, *J* = 8.1, 7.2, 1.1 Hz, H-11, 1H), 4.15 (s, N*H*$_2$, 2H) ppm (Figure S88).

^{13}C NMR (126 MHz, CDCl$_3$): δ 199.3 (C-6), 163.4 (C-8), 150.7 (C-3' and 4', 2C), 145.5 (C-1'), 144.2 (C-5), 140.4 (C-4), 140.1 (C-1), 137.2 (C-10), 134.2 (C-2), 133.3 (C-12), 123.3 (C-3), 123.0 (C-2' and 5', 2C), 119.2 (C-11), 119.2 (C-7), 118.8 (C-9) ppm (Figure S89).

HRMS (ESI) *m/z*: [M + H]$^+$ Calcd for $C_{17}H_{14}N_3O_2^+$ 292.1081 ; Found 292.1088.

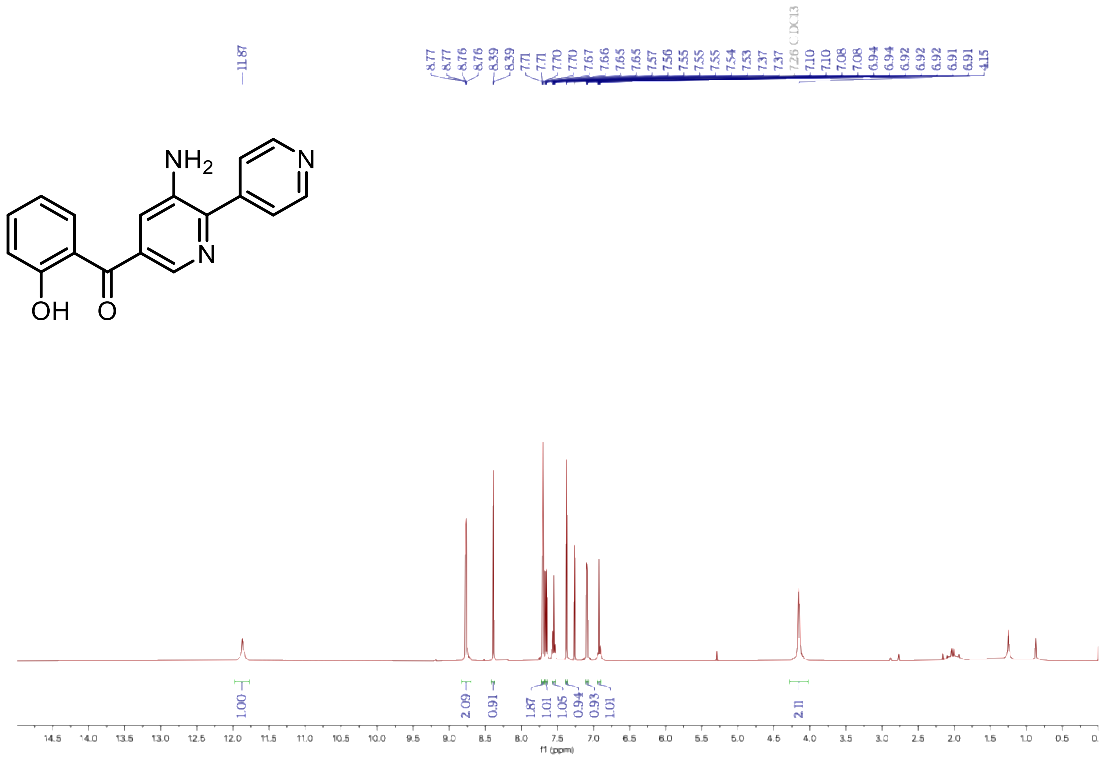

Figure S88 – ^{1}H-NMR (500 MHz, $CDCl_3$) of 3-aminopyridine **3w**.

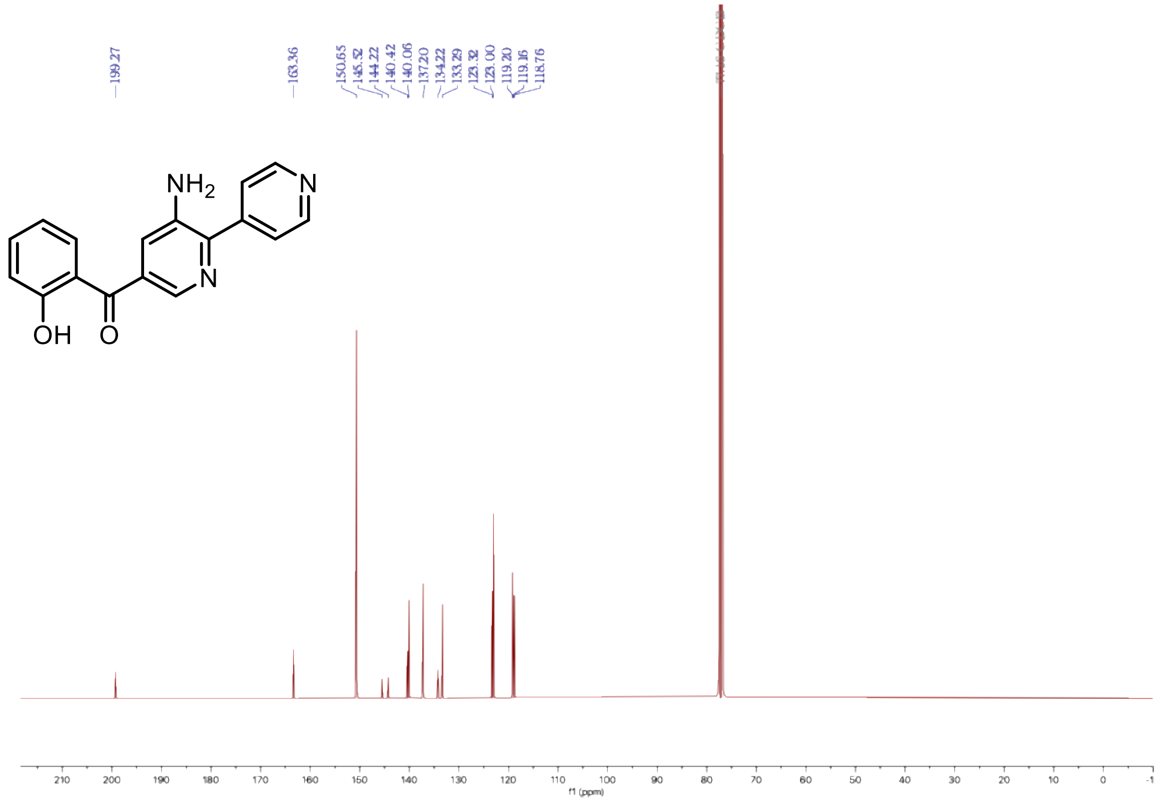

Figure S89 – ^{13}C-NMR (126 MHz, $CDCl_3$) of 3-aminopyridine **3w**.

[5-amino-6-(thiophen-2-yl)pyridin-3-yl](2-hydroxyphenyl)methanone (3x)

Following the general procedure using 4-oxo-4*H*-chromene-3-carbaldehyde **1a** (80.0 mg) and 1-[2-oxo-2-(thiophen-2-yl)ethyl]pyridin-1-ium bromide **2o** (195.8 mg). After purification by column chromatography using a gradient of 0% to 40% ethyl acetate in hexane, the compound **3x** (112.8 mg, 83% yield) was obtained.

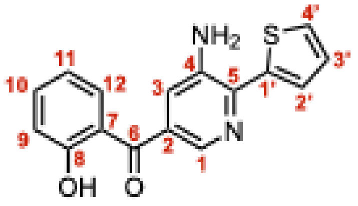

3x

Dark yellow solid. Melting point of 147.8–149.6 °C.

^{1}H NMR (300 MHz, CDCl$_3$): δ 11.89 (s, O*H*, 1H), 8.33 (d, *J* = 1.9 Hz, H-1, 1H), 7.68 – 7,62 (m, H-12 and H-2', 3' or 4', 2H), 7.55 – 7.47 (m, H-10 and H-2', 3' or 4', 2H), 7.36 (d, *J* = 1.9 Hz, H-3, 1H), 7.16 (dd, *J* = 5.1, 3.7 Hz, H-2', 3' or 4', 1H), 7.06 (dd, *J* = 8.4, 1.1 Hz, H-9, 1H), 6.88 (ddd, *J* = 8.2, 7.2, 1.2 Hz, H-11, 1H), 4.29 (s, NH_2, 2H) ppm (Figure S90).

^{13}C NMR (75 MHz, CDCl$_3$): δ 199.0 (C-6), 163.2 (C-8), 142.4 (C-1'), 141.7 (C-5), 140.2 (C-1), 139.3 (C-4), 136.9 (C-10), 133.3 (C-12), 132.4 (C-2), 128.5 (C-2', 3' or 4'), 128.1 (C-2', 3' or 4'), 126.3 (C-2', 3' or 4'), 123.7 (C-3), 119.2 (C-7), 119.1 (C-11), 118.6 (C-9) ppm (Figure S91).

HRMS (ESI) *m/z*: [M + H]$^+$ Calcd for $C_{16}H_{13}N_2O_2S^+$ 297.0692 ; Found 297.0700.

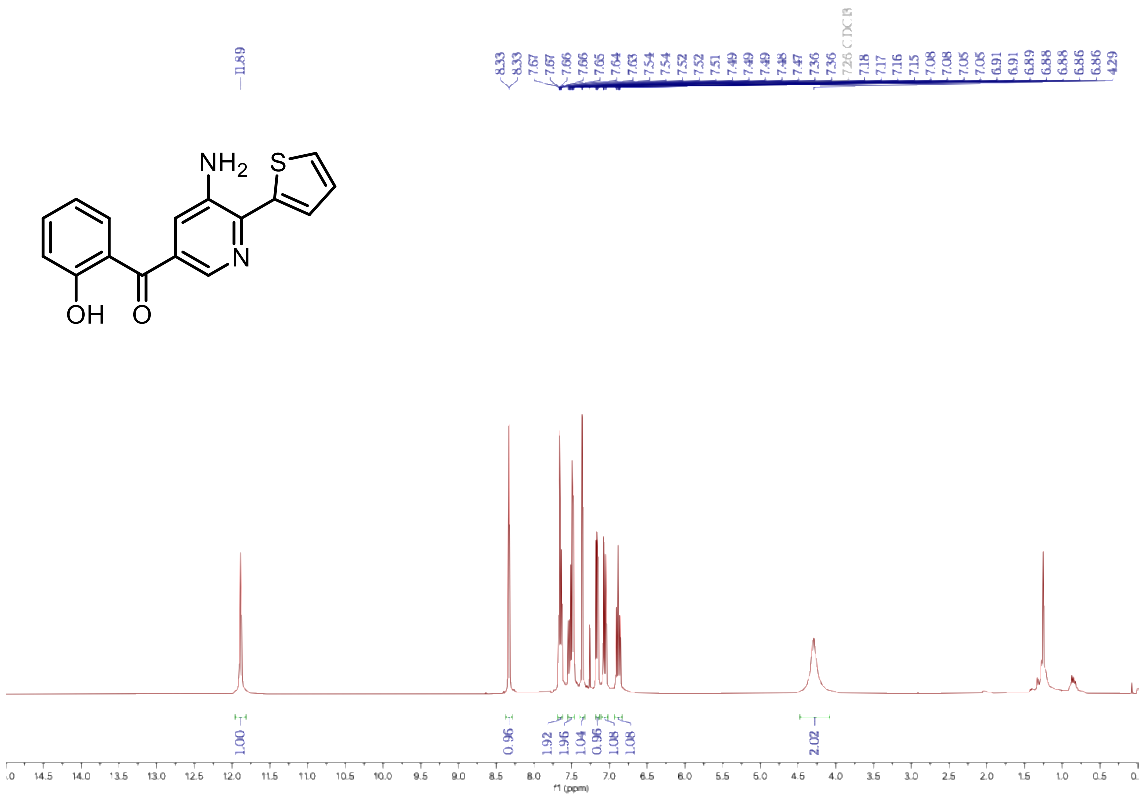

Figure S90 – ^{1}H-NMR (300 MHz, $CDCl_3$) of 3-aminopyridine **3x**.

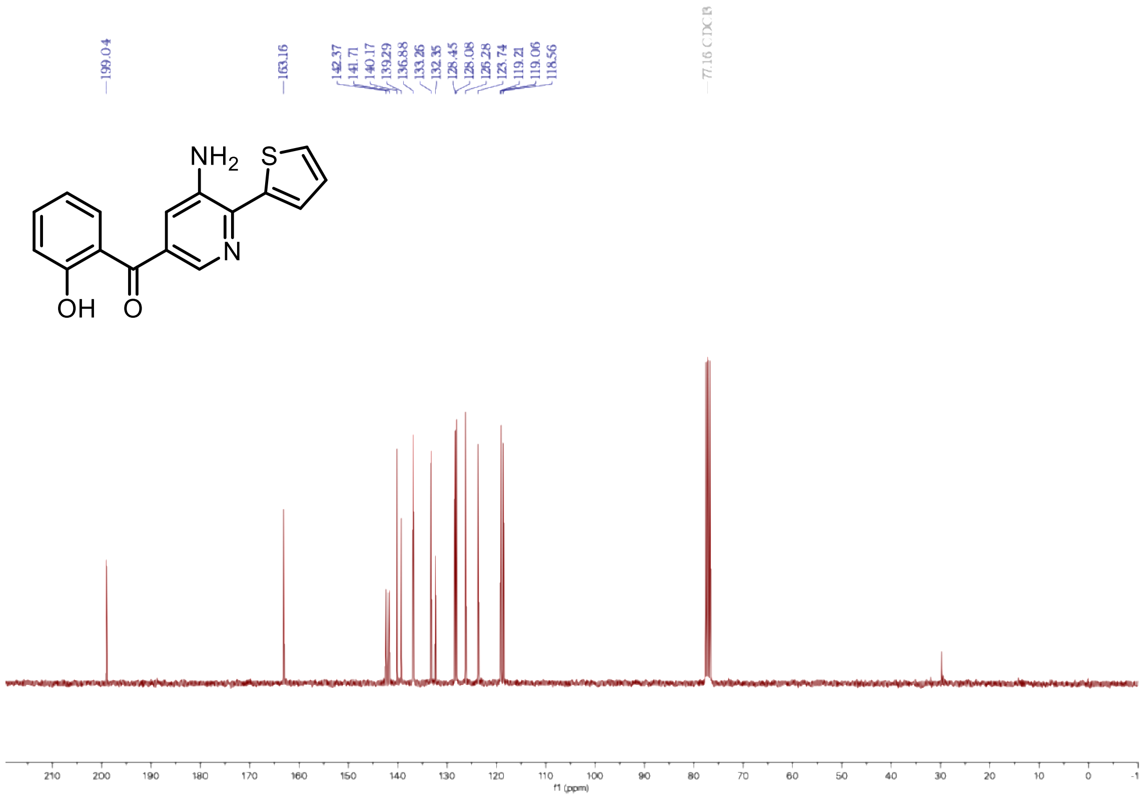

Figure S91 – ^{13}C-NMR (75 MHz, $CDCl_3$) of 3-aminopyridine **3x**.

7.3. Unexpected products

(5-ethoxy-6-phenylpyridin-3-yl)[2-hydroxy-5-(trifluoromethyl)phenyl]methanone (4)

Following the general procedure using 4-oxo-6-(trifluoromethyl)-4*H*-chromene-3-carbaldehyde **1l** (114.4 mg) and 1-(2-oxo-2-phenylethyl)pyridin-1-ium bromide **2a** (191.7 mg). After purification by column chromatography using a gradient of 0% to 40% ethyl acetate in hexane, the compound **4** (7.9 mg, 5% yield) was obtained.

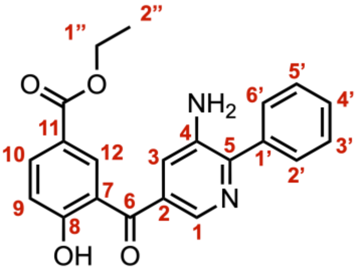

4

Yellow solid. Melting point of 152.8–155.2 °C.

^{1}H NMR (500 MHz, $CDCl_3$): δ 12.28 (s, O*H*, 1H), 8.43 (d, *J* = 2.1 Hz, H-12, 1H), 8.40 (d, *J* = 1.8 Hz, H-1, 1H), 8.21 (dd, *J* = 8.8, 2.2 Hz, H-10, 1H), 7.80 – 7.72 (m, H-2' and 6', 2H), 7.54 (td, *J* = 7.2, 1.4 Hz, H-3' and 5', 2H), 7.51 – 7.44 (m, H-4', 1H), 7.44 – 7.39 (m, H-3, 1H), 7.13 (d, *J* = 8.8 Hz, H-9, 1H), 4.48 – 4.26 (m, 2H), 4.34 (q, *J* = 7.1 Hz, H-1'', 2H), 1.36 (t, *J* = 7.1 Hz, H-2'', 2H) (Figure S92).

^{13}C NMR (126 MHz, $CDCl_3$): δ 199.0 (C-6), 166.7 (C-8), 165.5 (C-1''), 147.7 (C-5), 140.6 (C-1), 139.1 (C-4), 137.7 (C-10), 136.7 (C-1'), 135.4 (C-12), 132.8 (C-2), 129.6 (C-4'), 129.3 (C-3' and 5', 2C), 128.6 (C-2' and 6', 2C), 123.0 (C-3), 121.8 (C-11), 118.9 (C-9), 118.7 (C-7), 61.4 (C-2''), 14.5 (C-3'') ppm (Figure S93).

HRMS (ESI) *m/z*: $[M + H]^+$ Calcd for $C_{21}H_{19}N_2O_4^+$ 363.1339 ; Found 363.1334.

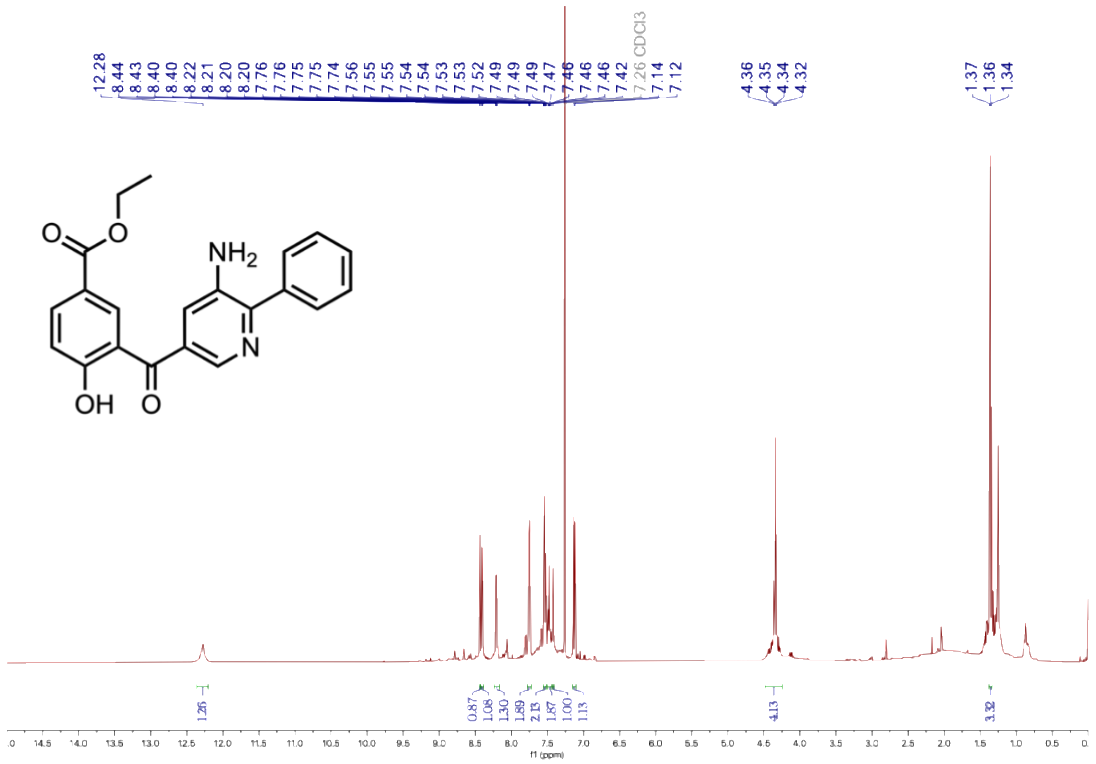

Figure S92 – ^{1}H-NMR (500 MHz, $CDCl_3$) of 3-aminopyridine **4**.

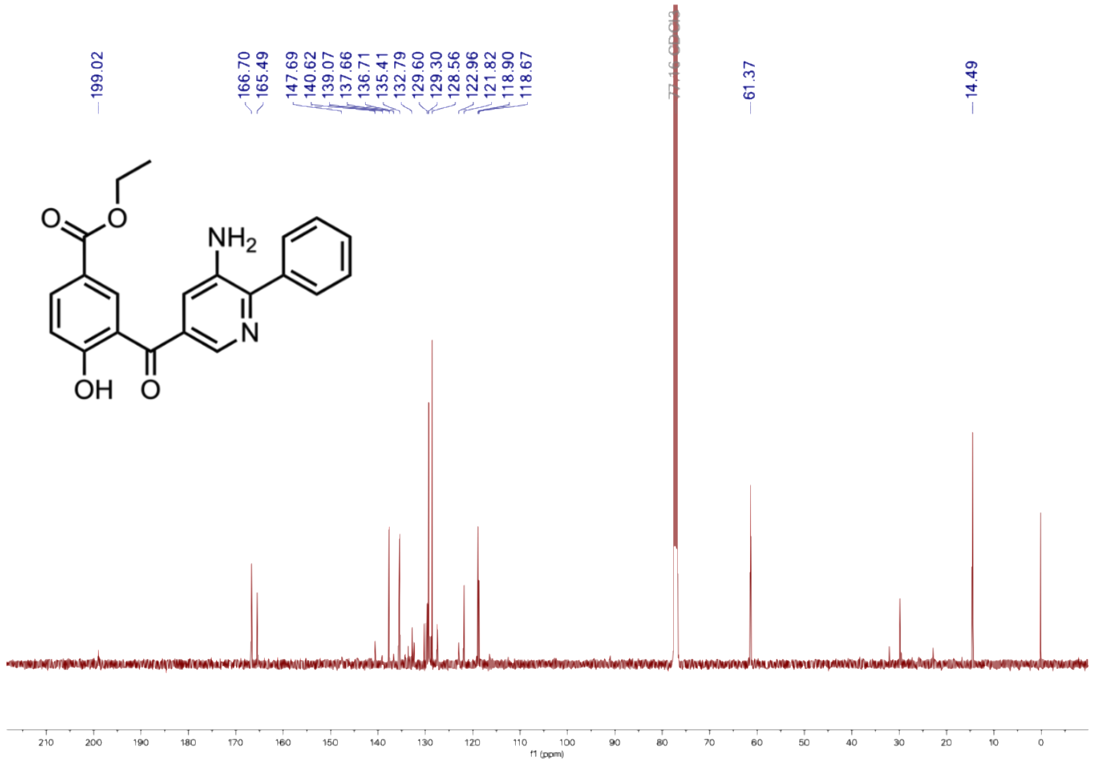

Figure S93 – ^{13}C-NMR (126 MHz, $CDCl_3$) of 3-aminopyridine **4**.

[10-(2-hydroxybenzyl)pyrido[2,3-*b*]indolizin-3-yl](2-hydroxyphenyl)methanone (5)

Following the general procedure using 4-oxo-4*H*-chromene-3-carbaldehyde **1a** (80.0 mg) and 1-(2-oxo-2-(2-oxo-2H-chromen-3-yl)ethyl)pyridin-1-ium bromide **2p** (238.5 mg). After purification by column chromatography using a gradient of 0% to 40% ethyl acetate in hexane, compound **5** (0.913 mmol, 36.0 mg, 20% yield) was obtained.

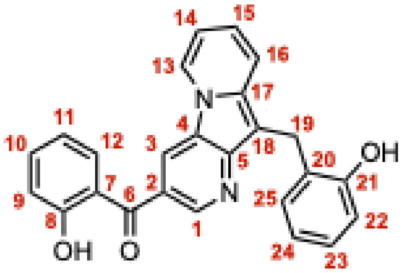

5

Bright orange solid. Melting point of 210.0–212.1 °C.

^{1}H NMR (500 MHz, $CDCl_3$): δ 11.80 (s, 8-O*H*, 1H), 10.78 (s, 21-O*H*, 1H), 9.05 (d, *J* = 1.8 Hz, H-1, 1H), 8.63 (d, *J* = 1.8 Hz, H-3, 1H), 8.39 (dd, *J* = 7.0, 1.3 Hz, H-13, 1H), 7.84 (dd, *J* = 9.3, 1.3 Hz, H-16, 1H), 7.66 (dd, *J* = 7.9, 1.7 Hz, H-12, 1H), 7.56 (ddd, *J* = 8.7, 7.1, 1.7 Hz, H-10, 1H), 7.30 (dd, *J* = 7.5, 1.7 Hz, H-25, 1H), 7.26 (m, H-15, 1H), 7.15 – 7.09 (m, H-9 and 23, 2H), 7.01 (dd, *J* = 8.1, 1.3 Hz, H-22, 1H), 6.96 (ddd, *J* = 7.9, 7.1, 1.7 Hz, H-11, 1H), 6.84 (td, *J* = 7.4, 1.3 Hz, H-24, 1H), 6.72 (ddd, *J* = 6.8, 6.7, 1.1 Hz, H-14, 1H), 4.35 (s, H-19, 2H) (Figure S94).

^{13}C NMR (126 MHz, $CDCl_3$): δ 198.4 (C-6), 163.0 (C-8), 155.3 (C-21), 147.0 (C-1), 145.1 (C-5), 139.8 (C-17), 136.4 (C-10), 133.2 (C-12), 130.2 (C-25), 129.1 (C-20), 128.3 (C-23), 126.7 (C-15), 125.8 (C-13), 123.9 (C-2), 122.3 (C-4), 120.9 (C-24), 120.3 (C-3), 120.0 (C-22), 119.7 (C-7), 119.2 (C-11), 118.7 (C-9), 117.7 (C-16), 110.4 (C-14), 105.7 (C-18), 24.5 (C-19) (Figure S95).

HRMS (ESI) *m/z*: $[M + H]^+$ Calcd for $C_{25}H_{18}N_2O_3^+$ 395.1390 ; Found 395.1380.

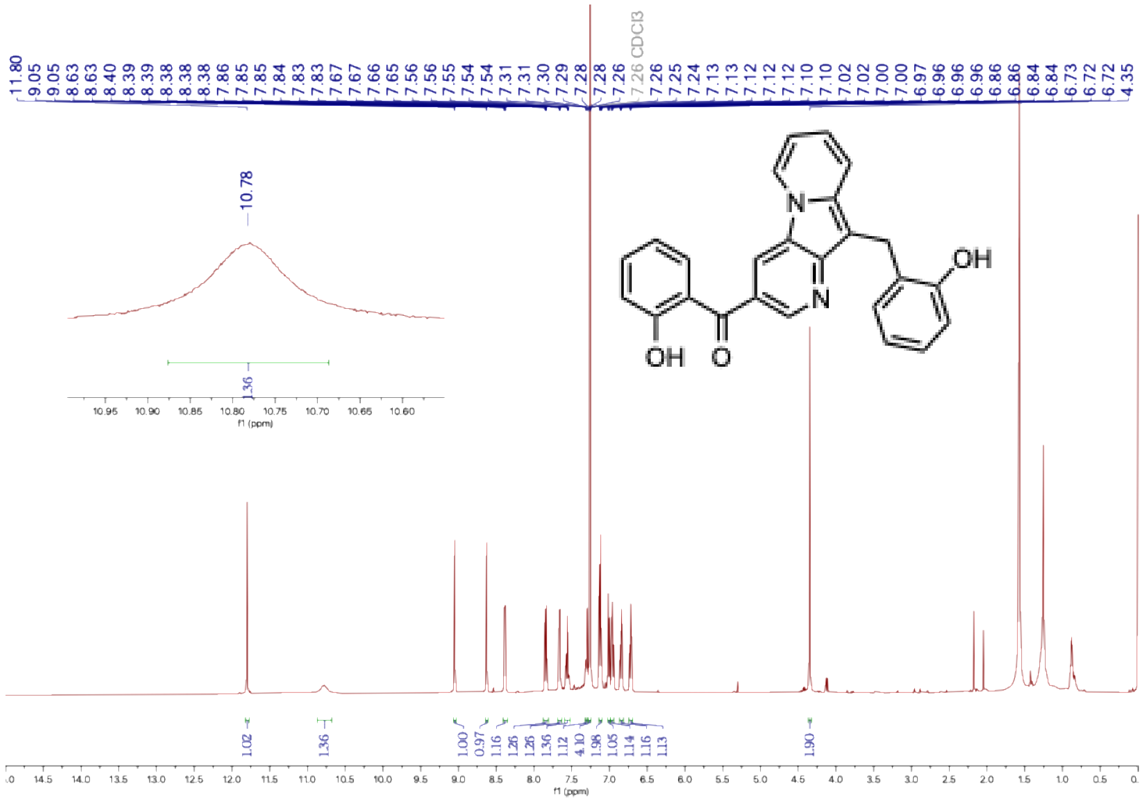

Figure S94 – ^{1}H-NMR (500 MHz, $CDCl_3$) of pyrido indolizine **5**.

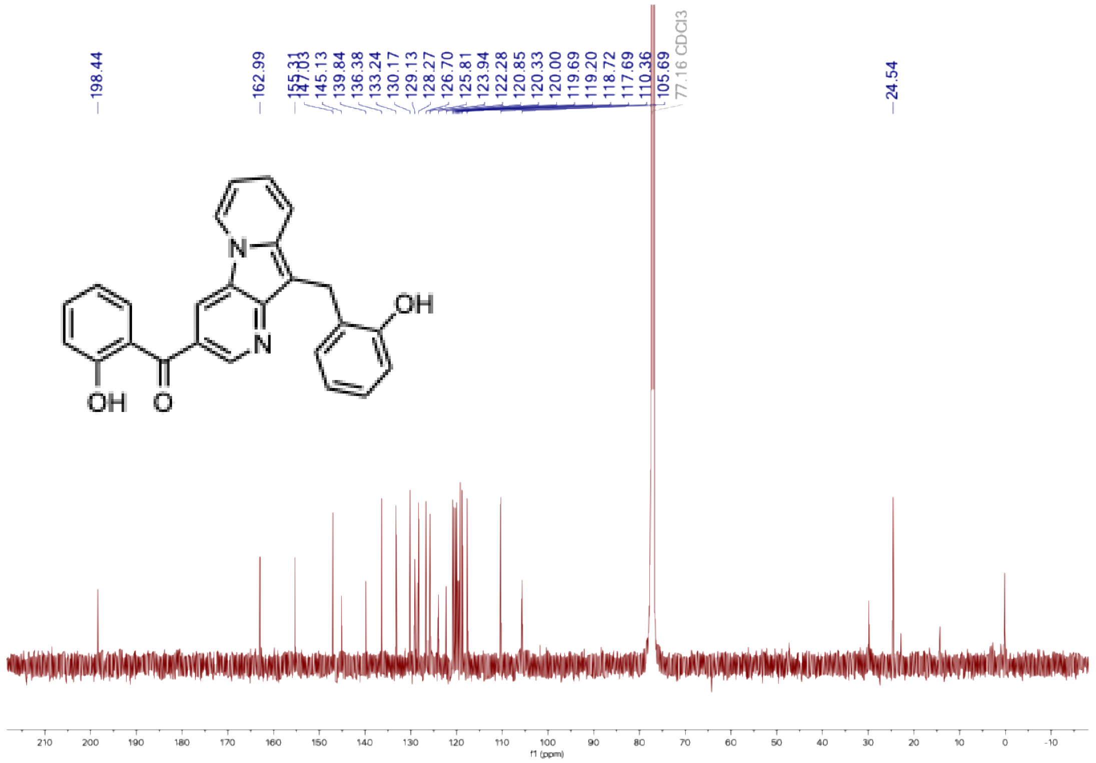

Figure S95 – ^{13}C-NMR (126 MHz, $CDCl_3$) of pyrido indolizine **5**.

Ethyl 4-(3-amino-5-(2-hydroxybenzoyl)pyridin-2-yl)benzoate (S4)

Following the general procedure using 4-oxo-4*H*-chromene-3-carbaldehyde **1a** (80.0 mg) and 1-[2-(4-carboxyphenyl)-2-oxoethyl]pyridin-1-ium bromide **2f** (228.9 mg). After purification by column chromatography using a gradient of 0% to 40% ethyl acetate in hexane, the compound **S4** (23.2 mg, 12% yield) was obtained.

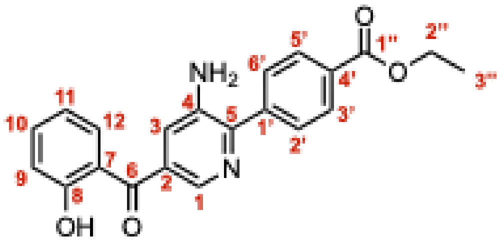

S4

Yellow solid. Melting point of 164.3–167.2 °C.

^{1}**H NMR (300 MHz, CDCl$_3$):** δ 11.85 (s, O*H*, 1H), 8.39 (d, *J* = 1.7 Hz, H-1, 1H), 8.23 – 8.16 (m, H-3' and 5', 2H), 7.88 – 7.81 (m, H-2' and 6', 2H), 7.67 (dd, *J* = 8.2, 1.7 Hz, H-12, 1H), 7.55 (ddd, *J* = 8.6, 7.2, 1.7 Hz, H-10, 1H), 7.41 (d, *J* = 1.8 Hz, H-3, 1H), 7.09 (dd, *J* = 8.5, 1.2 Hz, H-9, 1H), 6.92 (ddd, *J* = 8.2, 7.2, 1.2 Hz, H-11, 1H), 4.41 (q, *J* = 7.1 Hz, H-2'', 2H), 4.16 (s, N*H*$_2$, 2H), 1.42 (t, *J* = 7.1 Hz, H-3'', 3H) ppm (Figure S96).

^{13}**C NMR (75 MHz, CDCl$_3$):** δ 199.1 (C-6), 166.2 (C-1''), 163.3 (C-8), 145.8 (C-5), 141.2 (C-1'), 140.6 (C-4), 139.3 (C-1), 137.2 (C-10), 133.8 (C-2), 133.3 (C-12), 131.2 (C-4'), 130.4 (C-3' and 5', 2C), 128.6 (C-2' and 6', 2C), 123.4 (C-3), 119.2 (C-11), 119.2 (C-7), 118.8 (C-9), 61.4 (C-2''), 14.5 (C-3'') ppm (Figure S97).

HRMS (ESI) *m/z*: $[M + H]^+$ Calcd for $C_{21}H_{19}N_2O_4^+$ 363.1339 ; Found 363.1335.

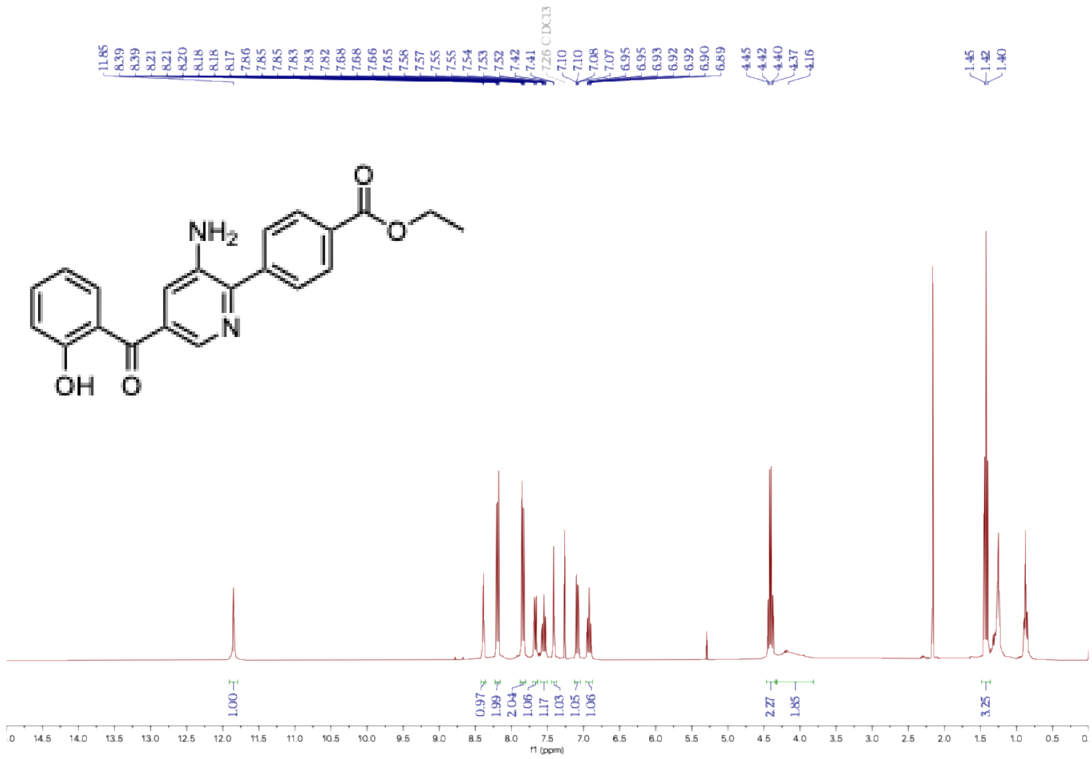

Figure S96 – ^{1}H-NMR (300 MHz, $CDCl_3$) of 3-aminopyridine **S4**.

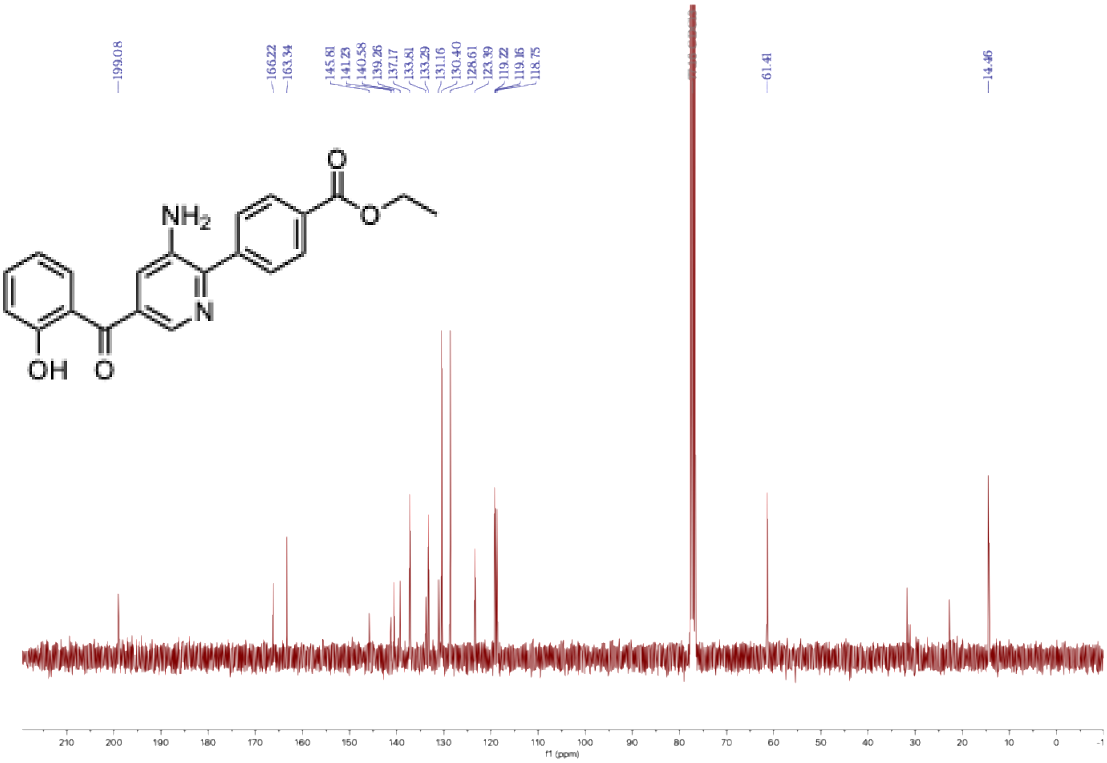

Figure S97 – ^{13}C-NMR (75 MHz, $CDCl_3$) of 3-aminopyridine **S4**.

N-{4-[3-amino-5-(2-hydroxybenzoyl)pyridin-2-yl]phenyl}acetamide (S5)

Following the general procedure using 4-oxo-4*H*-chromene-3-carbaldehyde **1a** (80.0 mg) and 1-[2-(4-aminophenyl)-2-oxoethyl]pyridin-1-ium bromide **2g** (202.0 mg. After purification by column chromatography using a gradient of 0% to 40% ethyl acetate in hexane, the compound **3p** (56.2 mg, 40% yield) and compound **S5** (22.5 mg, 12%) was obtained.

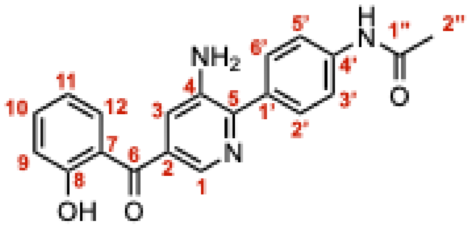

S5

Brown solid. Melting point of 177.2–179.0 °C.

^{1}H NMR (300 MHz, DMSO-d_6): δ 10.48 (s, O*H*, 1H), 10.13 (s, N*H*, 1H), 8.13 (d, *J* = 1.9 Hz, H-1, 1H), 7.76 – 7.64 (m, H-2', 3', 5' and 6', 4H), 7.51 – 7.39 (m, H-3, 10 and 12, 3H), 7.04 – 6.92 (m, H-9 and 11, 2H), 5.48 (s, NH_2, 2H), 2.08 (s, CH_3, 3H) ppm (Figure S98).

^{13}C NMR (75 MHz, DMSO-d_6): δ 196.5 (C-6), 168.9 (C-1''), 157.3 (C-8), 145.3 (C-5), 142.0 (C-4), 139.8 (C-4'), 137.7 (C-1), 134.0 (C-10), 132.5 (C-2 or 1'), 132.2 (C-2 or 1'), 130.7 (C-12), 129.0 (C-2' and 6', 2C), 124.5 (C-7), 122.7 (C-3), 119.5 (C-11), 119.0 (C-3' and 5', 2C), 117.0 (C-9), 24.2 (C-2'') ppm (Figure S99).

HRMS (ESI) *m/z*: $[M + H]^+$ Calcd for $C_{20}H_{18}N_3O_3^+$ 348.1343 ; Found 348.1337.

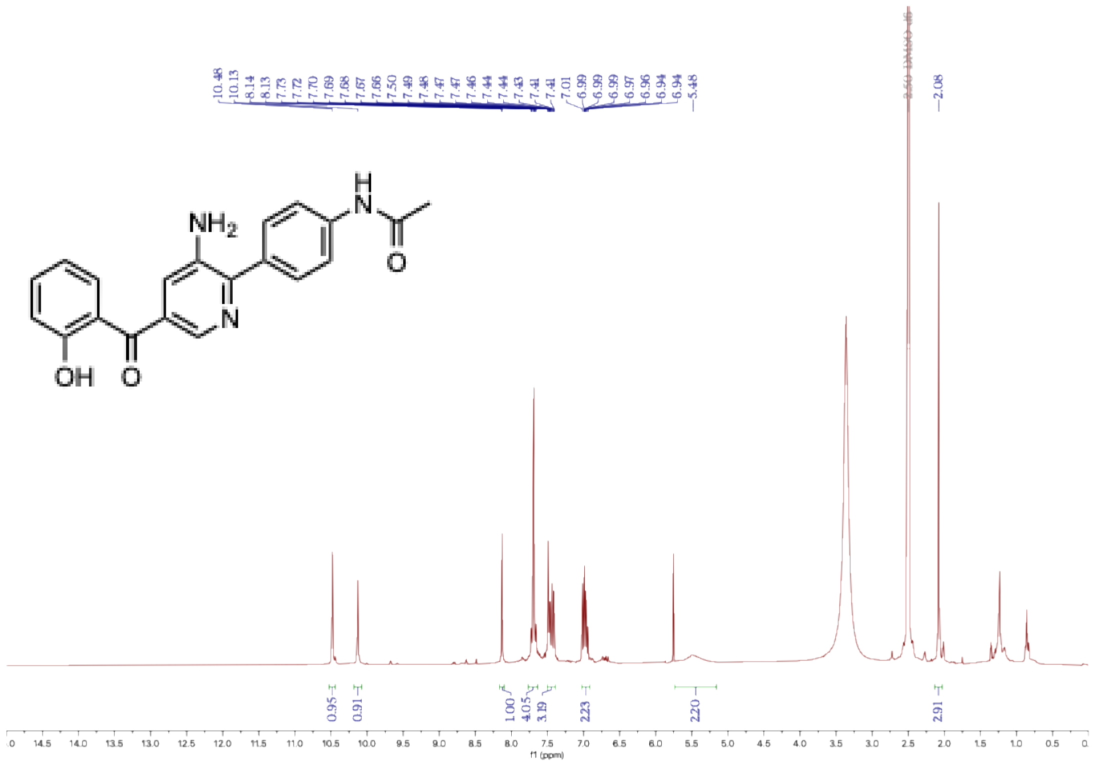

Figure S98 – ^{1}H-NMR (300 MHz, DMSO-d_6) of 3-aminopyridine **S5**.

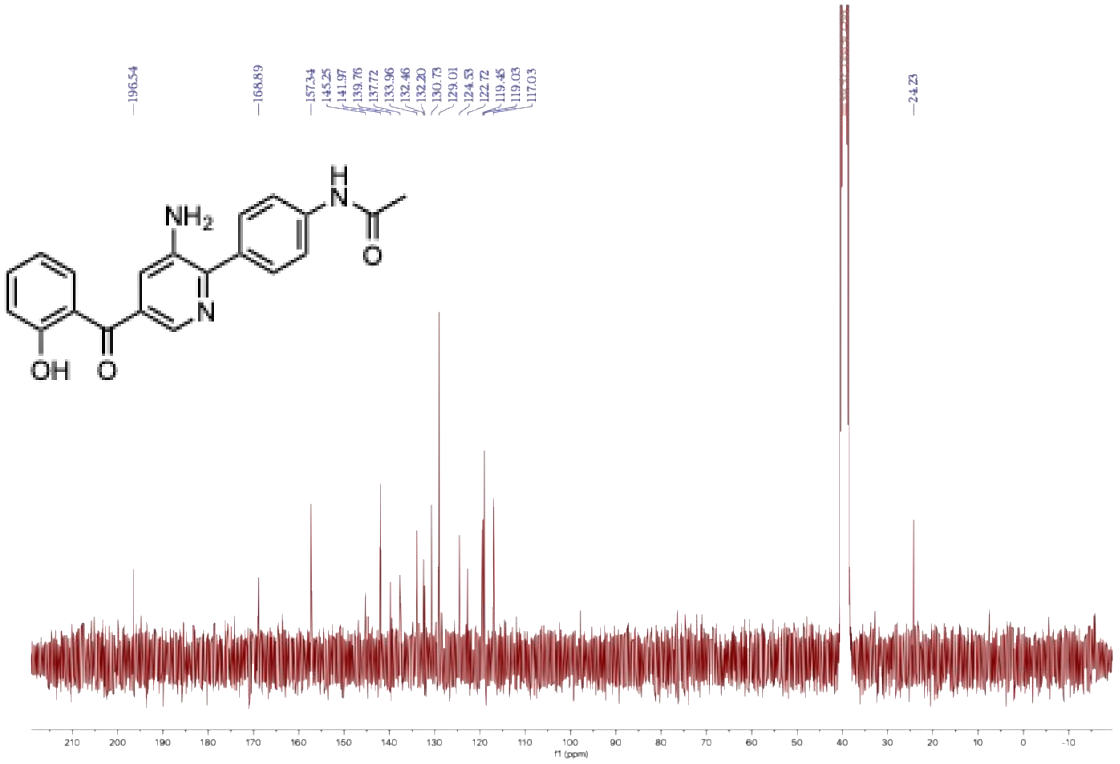

Figure S99 – ^{13}C-NMR (75 MHz, DMSO-d_6) of 3-aminopyridine **S5**.

8. Post-Functionalization of 3-Aminopyridine

8.1. Pyrrol Pyridine Synthesis

8.1.1. Procedure

In a 25 mL round bottom flask, (5-amino-6-phenylpyridin-3-yl)(2-hydroxyphenyl)methanone **3a** (1 equiv., 0.276 mmol, 80.0 mg), 2,5-dimethoxytetrahydrofuran (1.2 equiv., 0.331 mmol, 42.8 µL) and I_2 (10 mol%, 0.0276 mmol, 7.0 mg) were mixed in AcOH/EtOH (2 mL, 1:4). The mixture was let to stir at 80 °C for 16 h. The reaction was stopped with the addition of 5 mL of water and the product was extracted with DCM (3 × 10 mL). The product **8** (48%, 0.133 mmol, 45.3 mg) was purified by column chromatography using a gradient of 0% to 40% ethyl acetate in hexane (Scheme S8).[13]

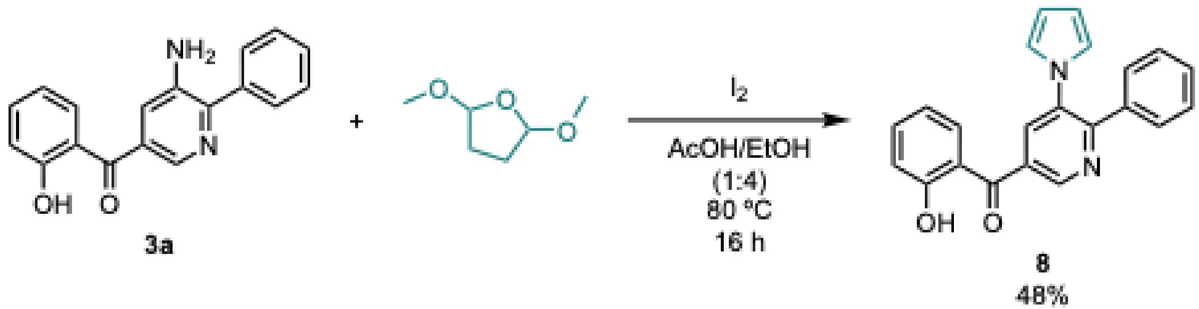

Scheme S8 - Synthesis of the (2-hydroxyphenyl)(6-phenyl-5-(1*H*-pyrrol-1-yl)pyridin-3-yl) **8**.

8.1.2. Characterization

(2-hydroxyphenyl)[6-phenyl-5-(1*H*-pyrrol-1-yl)pyridin-3-yl]methanone (8)

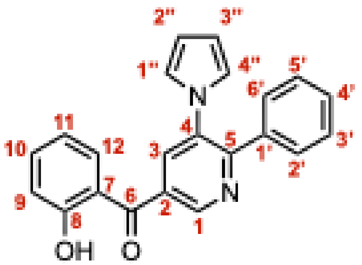

8

Light yellow solid. Melting point of 151.8–154.0 °C.

^{1}H NMR (300 MHz, $CDCl_3$): δ 11.81 (s, 1H), 8.97 (d, *J* = 2.0 Hz, 1H), 8.04 (d, *J* = 2.0 Hz, 1H), 7.68 (dd, *J* = 8.1, 1.7 Hz, 1H), 7.58 (ddd, *J* = 8.6, 7.2, 1.7 Hz, 1H), 7.43 – 7.33 (m, 5H), 7.13 (dd, *J* = 8.6, 1.2 Hz, 1H), 6.96 (ddd, *J* = 8.1, 7.2, 1.2 Hz, 1H), 6.68 (t, *J* = 2.2 Hz, 2H), 6.28 (t, *J* = 2.2 Hz, 2H) ppm (Figure S100).

^{13}C NMR (75 MHz, CDCl$_3$): δ 197.9 (C-6), 163.4 (C-8), 156.5 (C-5), 147.9 (C-1), 137.4 (C-10), 136.7 (C-1’), 135.5 (C-4), 135.1 (C-3), 133.0 (C-12), 132.7 (C-2), 129.7 (C-4’), 128.6 (C-2’ and 6’ or 3’ and 5’, 2C), 128.6 (C-2’ and 6’ or 3’ and 5’, 2C), 121.8 (C-1’’ and 4’’, 2C), 119.4 (C-11), 119.0 (C-7), 119.0 (C-9), 110.9 (C-2’’ and 3’’, 2C) ppm (Figure S101).

HRMS (ESI) *m/z*: [M + H]+ Calcd for $C_{22}H_{17}N_2O_2^+$ 341.1285 ; Found 341.1280.

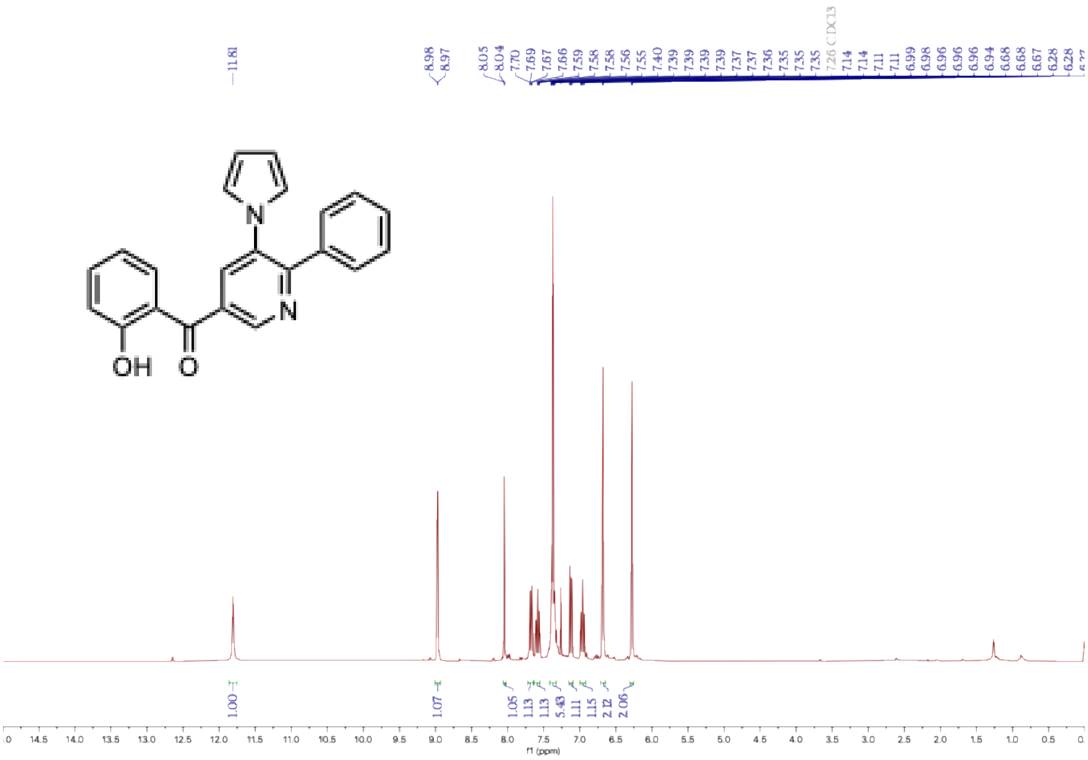

Figure S100 – ^{1}H-NMR (300 MHz, CDCl$_3$) of pyrrol pyridine **8**.

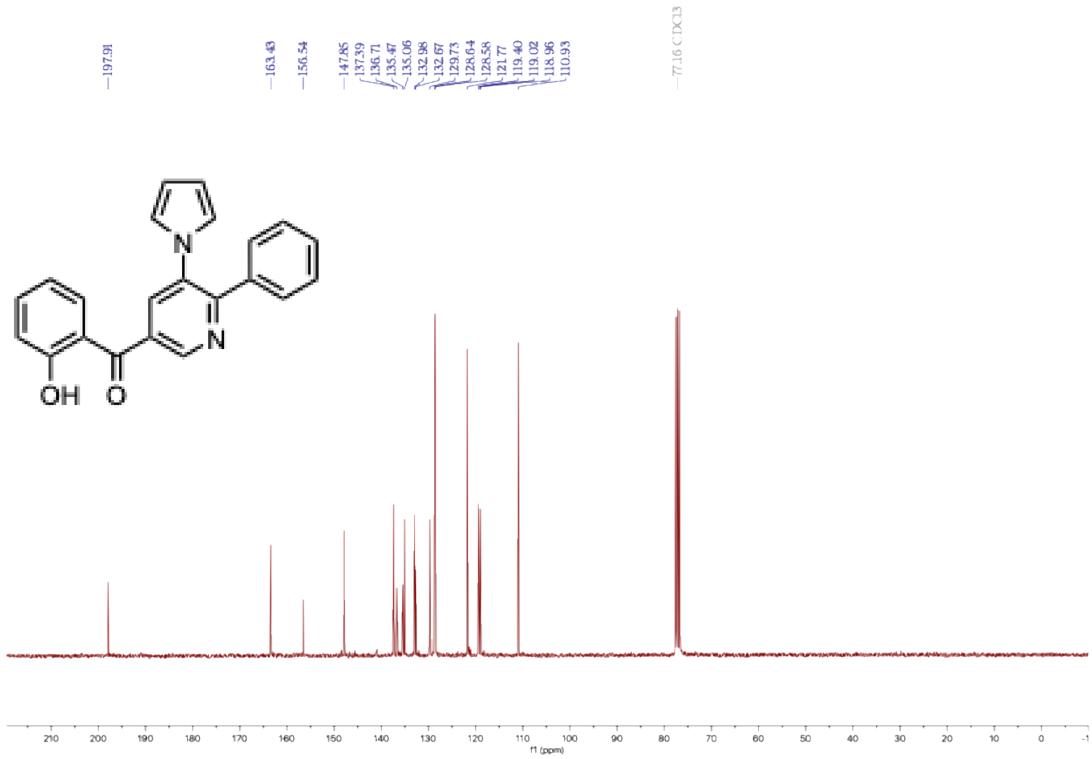

Figure S101 – ^{13}C-NMR (75 MHz, $CDCl_3$) of pyrrol pyridine **8**.

8.2. Pyridinyl Amide Synthesis

8.2.1. Pyridinyl Formamide – Procedure

In a Schlenk tube, (5-amino-6-phenylpyridin-3-yl)(2-hydroxyphenyl)methanone **3a** (1 equiv., 0.344 mmol, 100.0 mg) and KO*t*Bu (6 equiv., 2.06 mmol, 231.2 mg) in DMF (2 mL) under argon atmosphere were heated at 120 °C for 16 h. After cooling the reaction mixture, the organic phase was extracted with DCM (3 x 20 mL). The product **9** (0.152 mmol, 48.4 mg, 44%) was purified by column chromatography using a gradient of 0% to 40% ethyl acetate in hexane (Scheme S9).

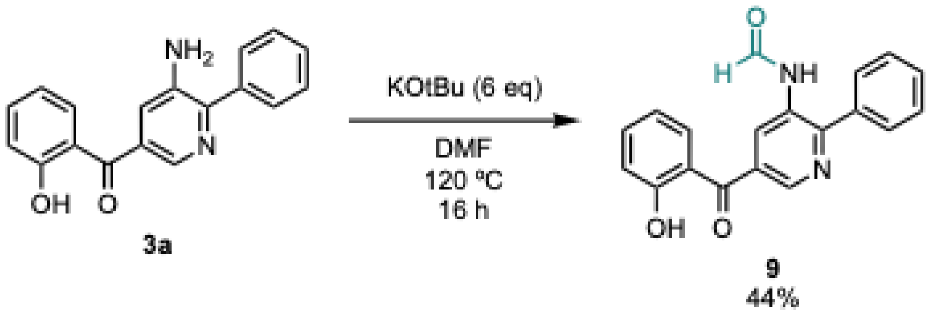

Scheme S9 - Synthesis of the *N*-(5-(2-hydroxybenzoyl)-2-phenylpyridin-3-yl)formamide **9**.

8.2.2. Pyridinyl Formamide – Characterization

N-[5-(2-hydroxybenzoyl)-2-phenylpyridin-3-yl]formamide (9)

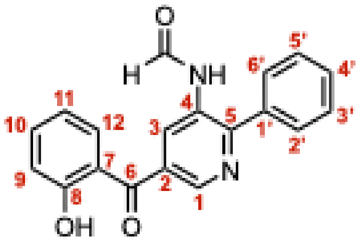

9

Orange solid. Melting point of 153.4–155.8 °C.

^{1}H NMR (300 MHz, $CDCl_3$): δ 11.91 (s, O*H*, 1H), 9.08 (d, *J* = 2.0 Hz, H-3, 1H), 8.74 (d, *J* = 2.0 Hz, H-1, 1H), 8.43 (d, *J* = 1.6 Hz, *H*CONH, 1H), 7.69 (dd, *J* = 8.1, 1.7 Hz, H-12, 1H), 7.66 – 7.51 (m, H-10, 2', 3', 4', 5', 6' and HCON*H*, 7H), 7.11 (dd, *J* = 8.5, 1.1 Hz, H-9, 1H), 6.96 (ddd, *J* = 8.2, 7.2, 1.2 Hz, H-11, 1H) (Figure S102).

^{13}C NMR (75 MHz, $CDCl_3$): δ 198.8 (C-6), 163.5 (C-8), 159.3 (H*C*ONH), 151.6 (C-5), 145.1 (C-1), 137.3 (C-12), 136.2 (C-1'), 133.3 (C-10), 133.0 (C-2), 130.8 (C-4), 130.1 (C-4'), 129.7 (C-3' and 5', 2C), 129.3 (C-3), 128.9 (C-2' and 6', 2C), 119.4 (C-11), 119.1 (C-7), 118.8 (C-9) (Figure S103).

HRMS (ESI) *m/z*: $[M + H]^+$ Calcd for $C_{19}H_{15}N_2O_3^+$ 319.1077 ; Found 319.1072.

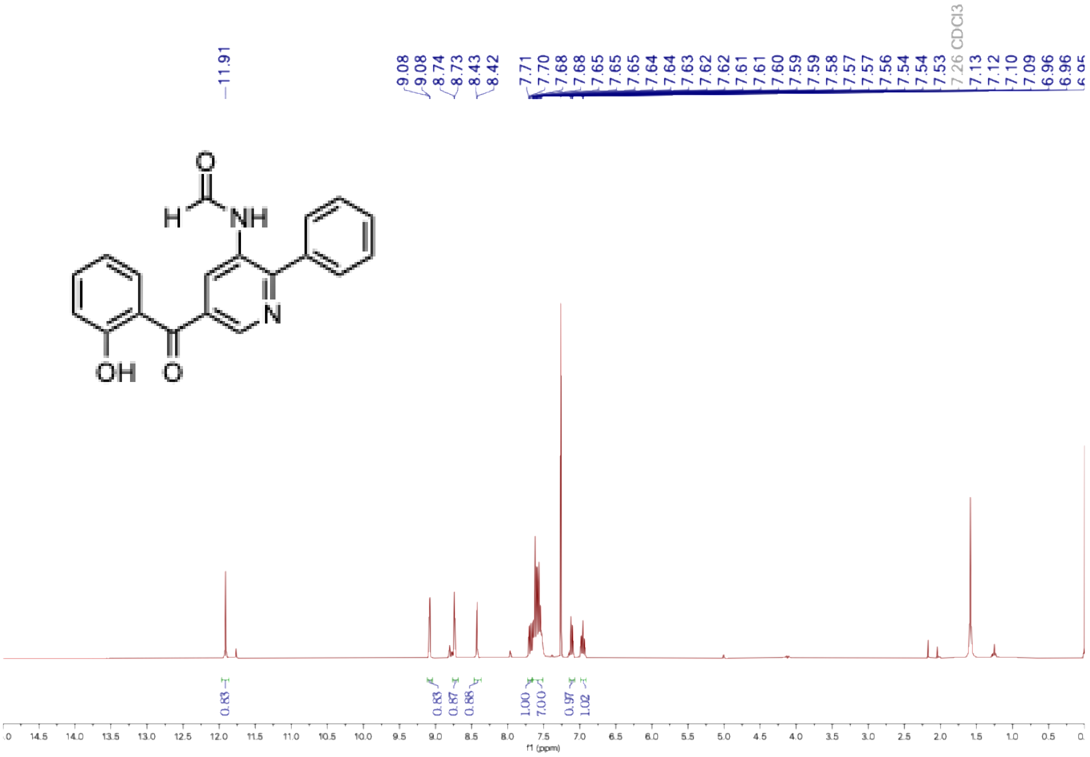

Figure S102 – ^{1}H-NMR (300 MHz, $CDCl_3$) of pyridinyl formamide **9**.

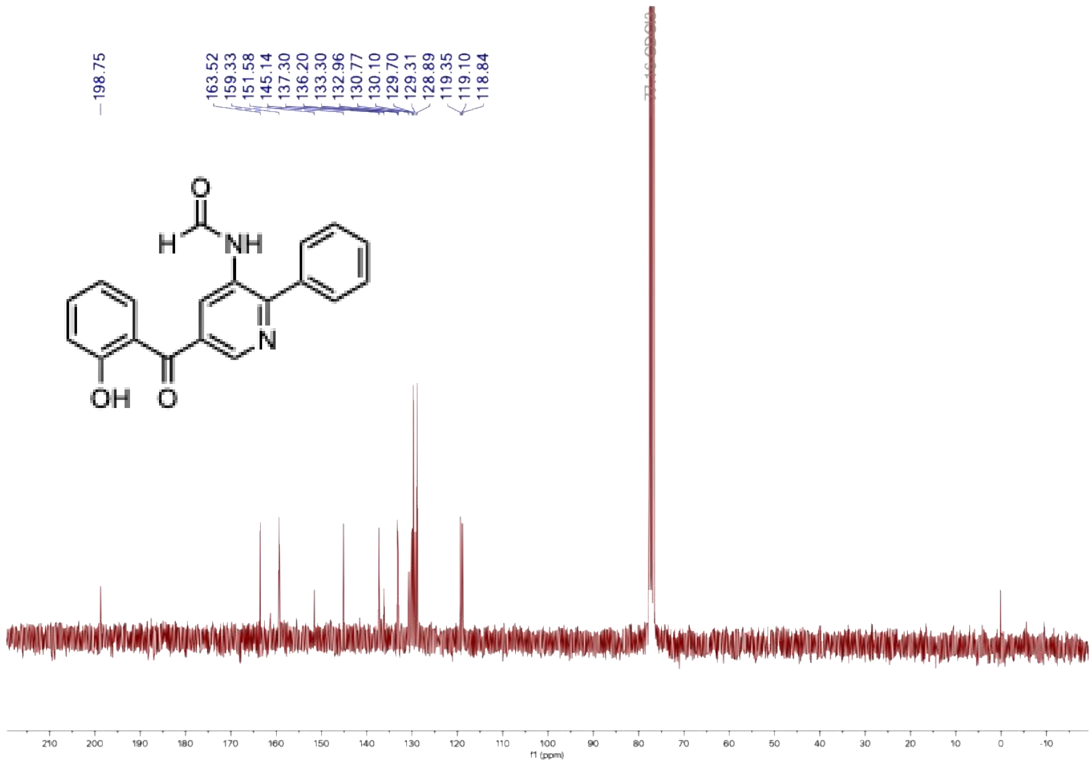

Figure S103 – ^{13}C-NMR (75 MHz, $CDCl_3$) of pyridinyl formamide **9**.

8.2.3. Pyridinyl Trifluoroacetamide – Procedure

In a 25 mL round bottom flask, (5-amino-6-phenylpyridin-3-yl)(2-hydroxyphenyl)methanone **3a** (1 equiv., 0.172 mmol, 50.0 mg), 2,2,2-trifluoroacetic anhydride (TFAA) (1.5 equiv., 0,26 mmol, 36.1 µL) and triethylamine (TEA) (1.1 equiv., 0.19 mmol, 26.4 µL) are mixed in DCM (1 mL, 0.17 M). The mixture was let to stir at room temperature for 16 h. The reaction was stopped with the addition of 5 mL of water and the product was extracted with DCM (3 × 3 mL). The product **10** (84%, 0.142 mmol, 54.9 mg) was purified by column chromatography using a gradient of 0% to 40% ethyl acetate in hexane (Scheme S10).

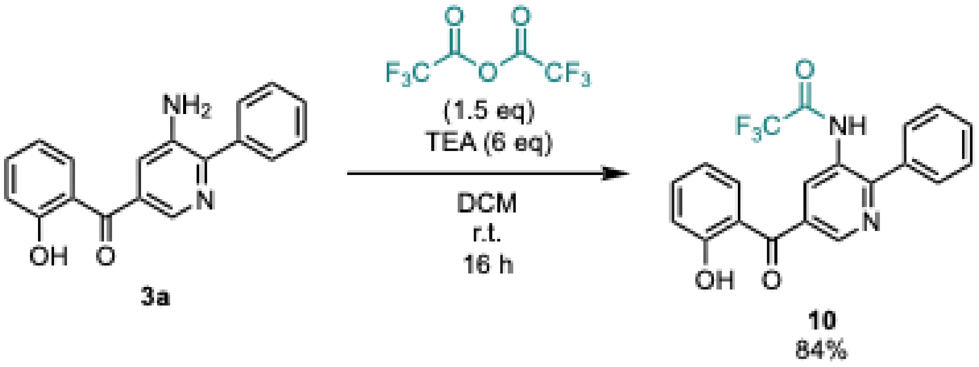

Scheme S10 - Synthesis of the 2,2,2-trifluoro-*N*-(5-(2-hydroxybenzoyl)-2-phenylpyridin-3-yl)acetamide **10**.

8.2.4. Pyridinyl Trifluoroacetamide – Characterization

2,2,2-trifluoro-*N*-(5-(2-hydroxybenzoyl)-2-phenylpyridin-3-yl)acetamide (10)

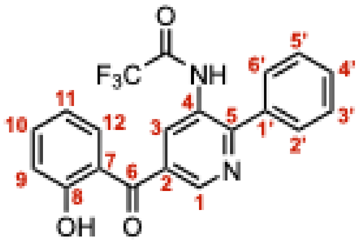

10

Yellow solid. Melting point of 164.2–166.3 °C.

^{1}H NMR (300 MHz, $CDCl_3$): δ 11.82 (s, O*H*, 1H), 8.81 (d, *J* = 1.9 Hz, H-3, 1H), 8.75 (d, *J* = 1.9 Hz, H-1, 1H), 8.56 (s, NH_2, 1H), 7.64 (dd, *J* = 8.1, 1.8 Hz, H-12, 1H), 7.61 – 7.51 (m, H-10, 2’, 3’, 4’, 5’ and 6’, 6H), 7.11 (dd, *J* = 8.4, 1.1 Hz, H-9, 1H), 6.96 (ddd, *J* = 8.1, 7.2, 1.1 Hz, H-11, 1H) ppm (Figure S104).

^{13}C NMR (75 MHz, $CDCl_3$): δ 198.0 (C-6), 163.5 (C-8), 155.4 (q, *J* = 38.3 Hz, 4-N*C*OCF_3), 152.9 (C-1’), 146.8 (C-1), 137.5 (C-10), 135.3 (C-5), 133.1 (C-12), 132.9 (C-4), 130.6 (C-1 or 4’), 130.1 (C-1 or 4’), 129.7 (C-2’ and 6’ or 3’ and 5’, 2C), 129.1 (C-2), 128.7 (C-2’ and 6’ or 3’ and 5’, 2C), 119.5 (C-11), 118.9 (C-9), 118.9 (C-7), 115.5 (q, *J* = 289.0 Hz, 4-NCO*C*F_3) ppm (Figure S105).

^{19}F NMR (282 MHz, $CDCl_3$): δ -72.52 (CF_3) ppm (Figure S106).

HRMS (ESI) *m/z*: $[M + H]^+$ Calcd for $C_{20}H_{14}F_3N_2O_3^+$ 387.0951 ; Found 387.0946.

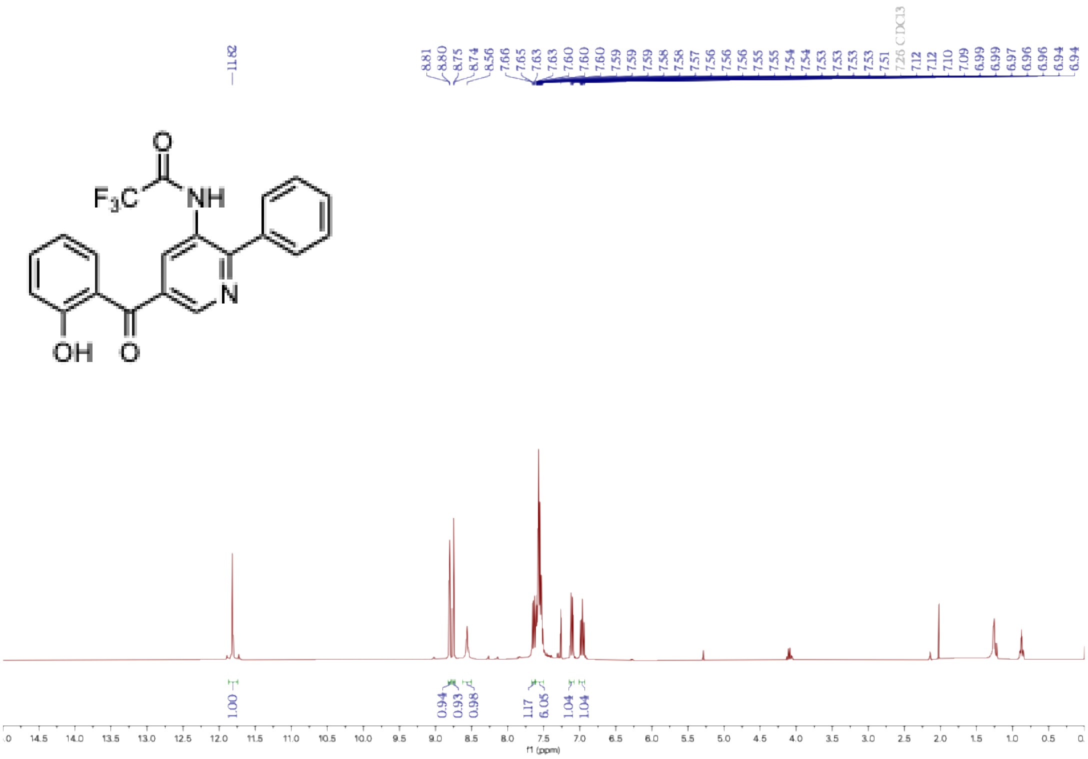

Figure S104 – ^{1}H-NMR (300 MHz, $CDCl_3$) of pyridinyl trifluoroacetamide **10**.

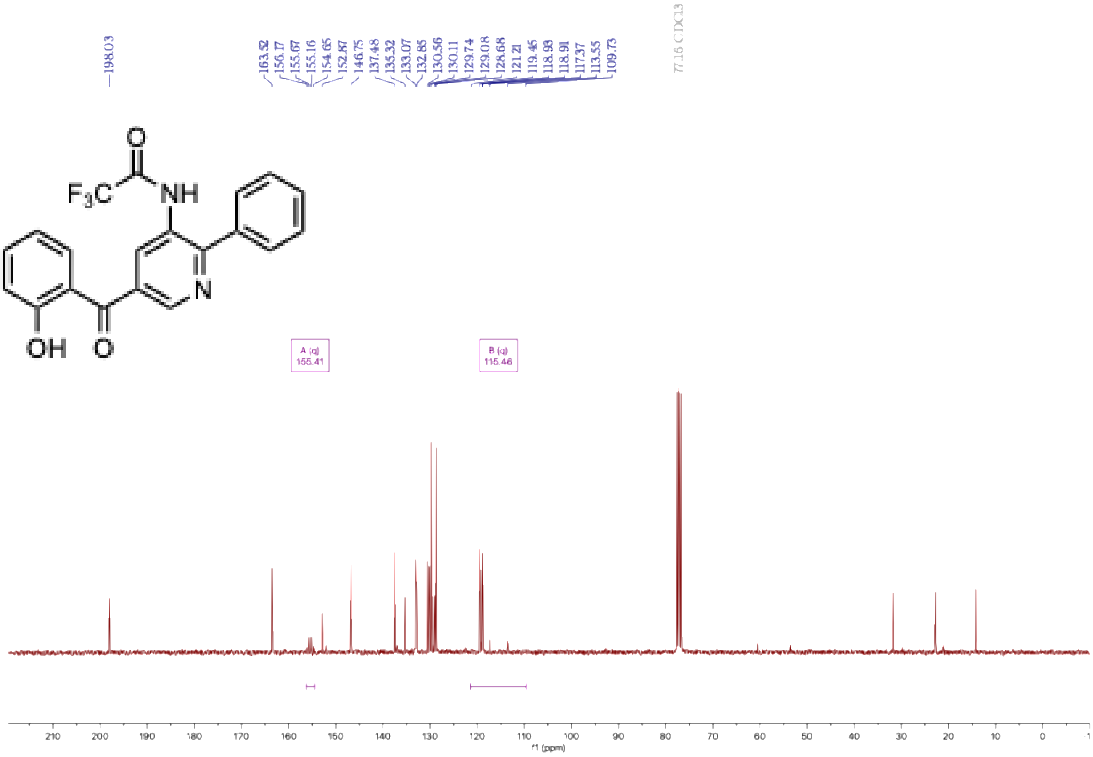

Figure S105 – ^{13}C-NMR (75 MHz, $CDCl_3$) of pyridinyl trifluoroacetamide **10**.

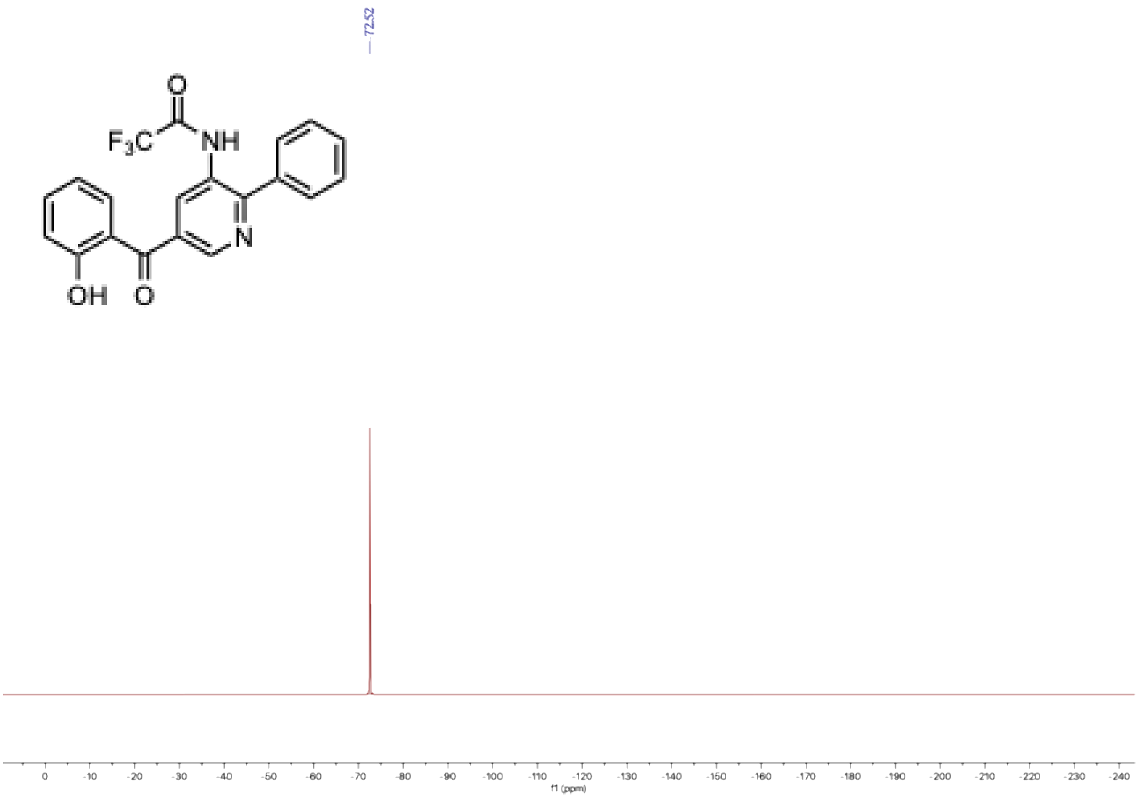

Figure S106 – ^{19}F-NMR (282 MHz, $CDCl_3$) of pyridinyl trifluoroacetamide **10**.

8.3. Halogen Pyridine

8.3.1. 3-Fluoropyridine – Procedure

In a Schlenk tube with 1,2-dichlorobenzene (ODCB) (2 mL) under argon atmosphere, the (5-amino-6-phenylpyridin-3-yl)(2-hydroxyphenyl)methanone **3a** (1 equiv., 0.34 mmol, 100,0 mg) and BF_3OEt_2 (2 equiv., 0.68 mmol, 83.9 µL) were added at room temperature. The mixture was heated to 100 °C and when the temperature was reached, the ^{t}BuONO (1.5 equiv., 0.51 mmol, 60.9 µL) was slowly added for 15 minutes. The mixture was let to react for 2 hours. After cooling, water (3 mL) was added into the medium and the organic phase extracted with DCM, dried with anhydrous sodium sulfate and concentrated under reduced pressure. The crude was purified by column chromatography using a gradient of 0% to 30% ethyl acetate in hexane obtaining the 3-fluoropyridine **11** (0.32 mmol, 94.4 mg, 93%) (Scheme S11).[14]

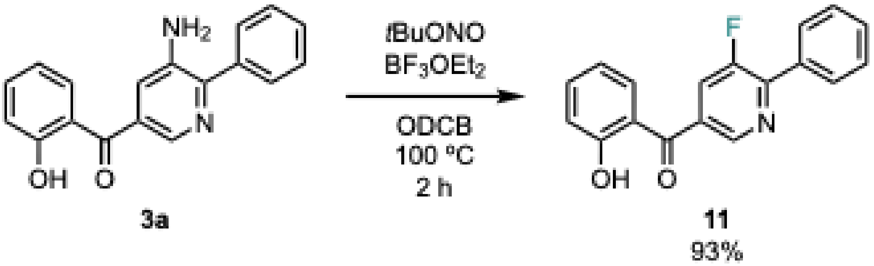

Scheme S11 - Synthesis of the (5-fluoro-6-phenylpyridin-3-yl)(2-hydroxyphenyl)methanone **11**.

8.3.2. 3-Fluoropyridine – Characterization

(5-fluoro-6-phenylpyridin-3-yl)(2-hydroxyphenyl)methanone (11)

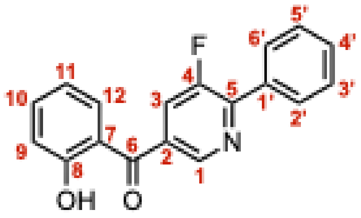

11

Yellow solid. Melting point of 165.8–168.3 °C.

^{1}H NMR (300 MHz, $CDCl_3$): δ 11.77 (s, O*H*, 1H), 8.82 (t, *J* = 1.7 Hz, H-1, 1H), 8.11 – 8.05 (m, H-2' and 6', 2H), 7.83 (dd, *J* = 11.0, 1.7 Hz, H-3, 1H), 7.64 (dd, *J* = 8.1, 1.7 Hz, H-12, 1H), 7.61 – 7.48 (m, H-10, 3', 4' and 5', 4H), 7.12 (dd, *J* = 8.4, 1.2 Hz, H-9, 1H), 6.95 (ddd, *J* = 8.1, 7.2, 1.2 Hz, H-11, 1H) ppm (Figure S107).

^{13}C NMR (75 MHz, $CDCl_3$): δ 197.4 (C-6), 163.4 (C-8), 157.0 (d, *J* = 263.5 Hz, C-4), 149.2 (d, *J* = 10.5 Hz, C-5), 145.5 (d, *J* = 5.3 Hz, C-1), 137.3 (C-10), 134.4 (d, *J* = 5.6 Hz, C-2), 133.4 (d, *J* = 3.0

Hz, C-1’), 132.9 (C-12), 130.3 (C-4’), 129.19 (d, *J* = 6.3 Hz, C-2’ and 6’, 2C), 128.8 (C-3’ and 5’, 2C), 124.8 (d, *J* = 22.4 Hz, C-3), 119.3 (C-9), 119.0 (C-7), 118.9 (C-11) ppm (Figure S108).

^{19}F NMR (282 MHz, $CDCl_3$): δ -118.7 (d, *J* = 11.0 Hz) ppm (Figure S109).

HRMS (ESI) *m/z*: $[M + H]^+$ Calcd for $C_{18}H_{13}FNO_2^+$ 294.0925 ; Found 294.0920.

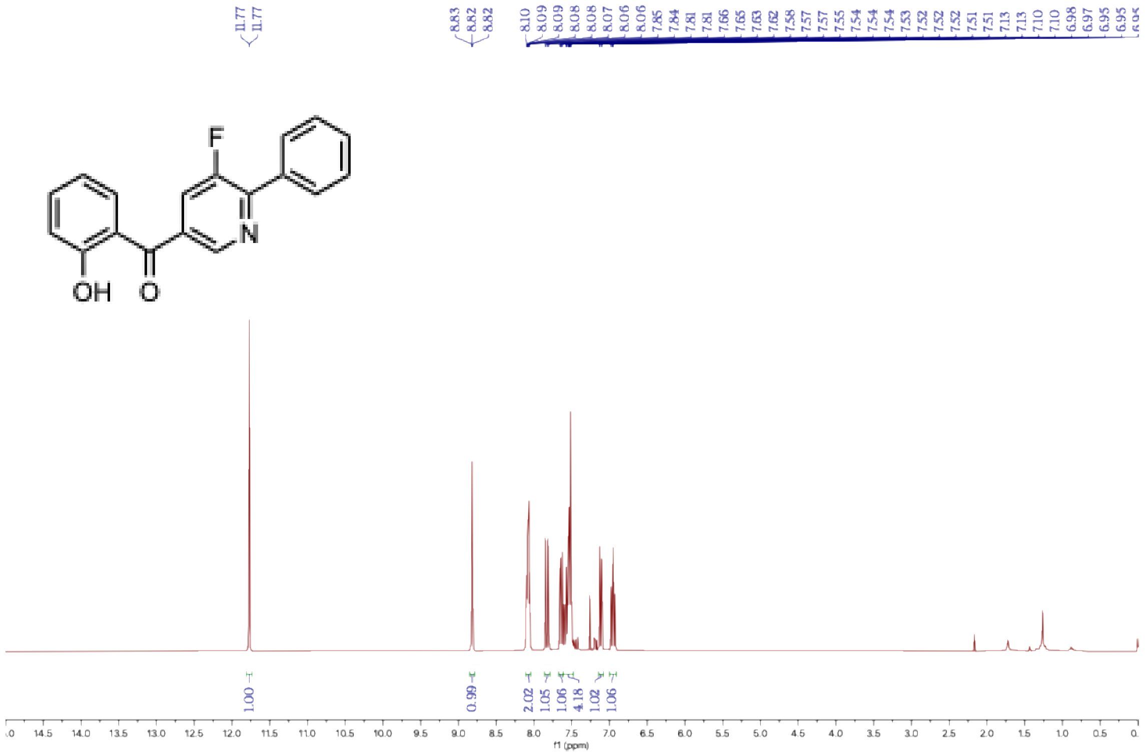

Figure S107 – ^{1}H-NMR (300 MHz, $CDCl_3$) of 3-fluoropyridine **11**.

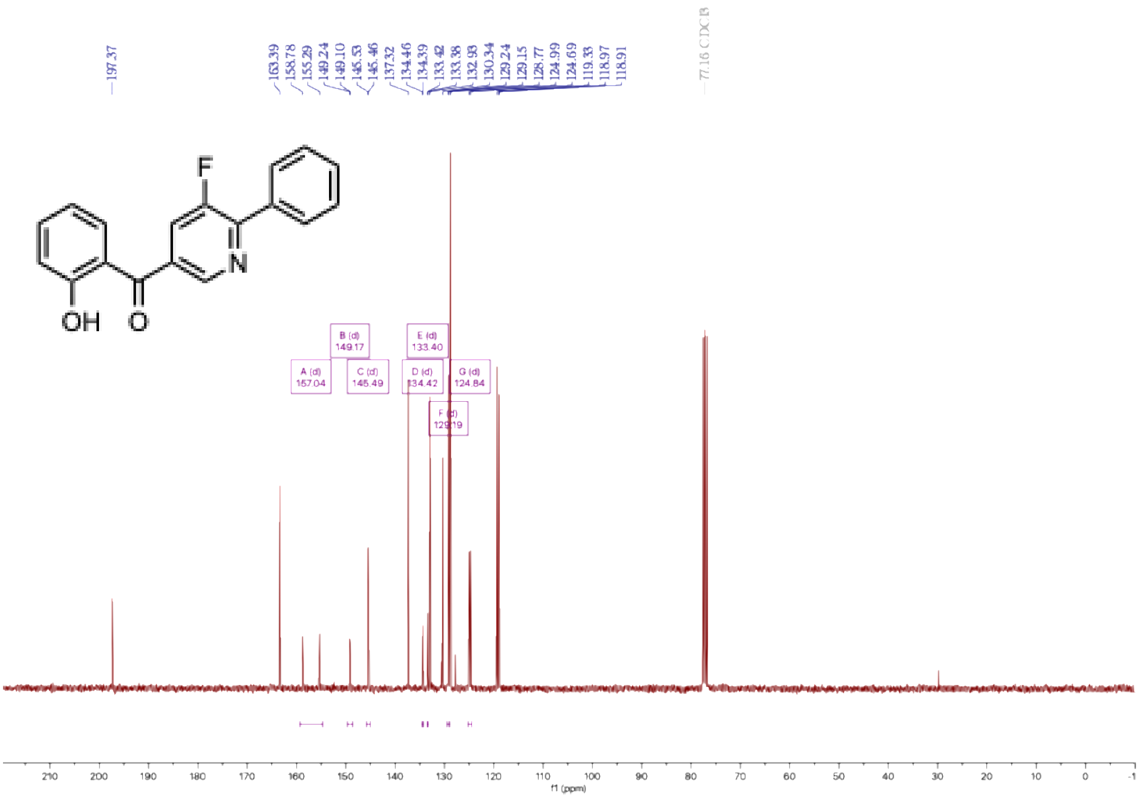

Figure S108 – ^{13}C-NMR (75 MHz, $CDCl_3$) of 3-fluoropyridine **11**.

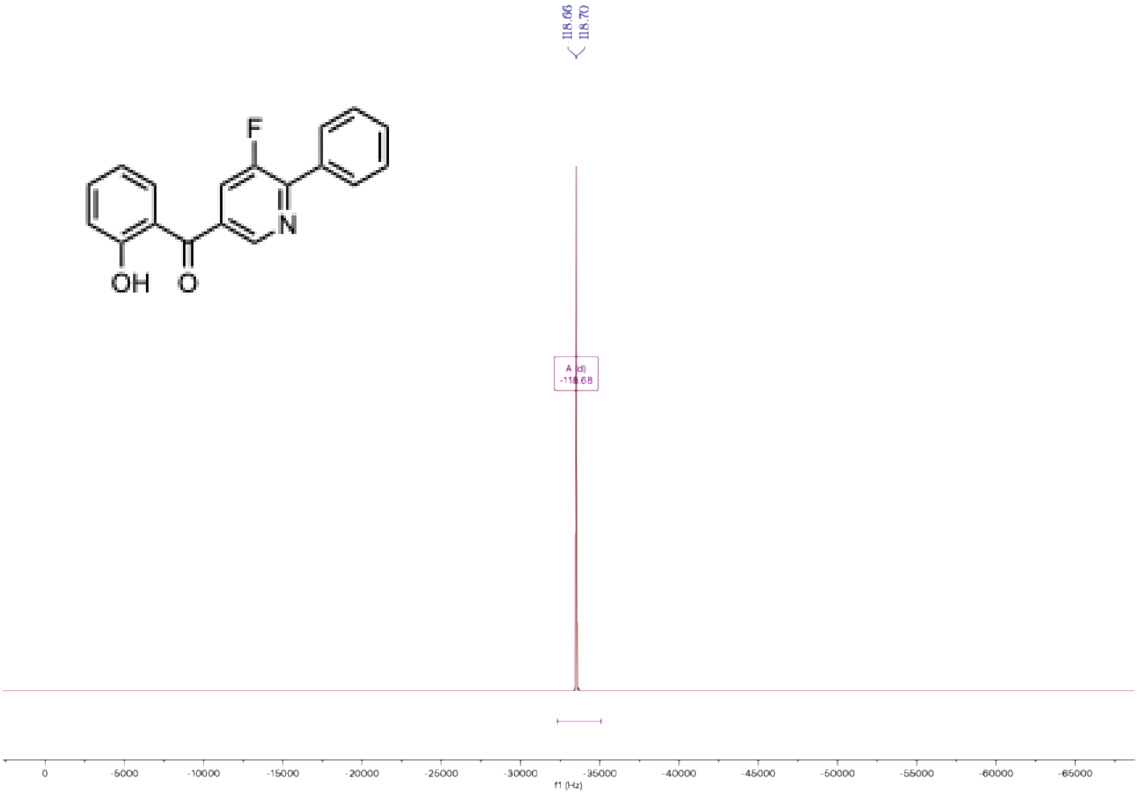

Figure S109 – ^{19}F-NMR (282 MHz, $CDCl_3$) of 3-fluoropyridine **11**.

8.3.3. 3-Chloropyridine – Procedure

In a 25 mL round flask with the (5-amino-6-phenylpyridin-3-yl)(2-hydroxyphenyl)methanone **3a** (1 equiv., 0.34 mmol, 100,0 mg) in 1,4-dioxane (0.5 mL), hydrochloric acid 37% (0.6 mL) was added. The mixture was cooled to 0 °C and then a solution of $NaNO_2$ (1.2 equiv., 0.41 mmol, 28.3 mg) in H_2O (0.2 mL) was added dropwise. The reaction was let to stir at 0 °C. Then, CuCl in hydrochloric acid 37% was added and the reaction was let to stir at room temperature for 1 hour. The reaction was stopped using iced water and the organic phase was extracted using ethyl acetate (3 x 20 mL), dried with sodium sulfate and concentrated under reduced pressure. The crude was purified by column chromatography using a gradient of 0% to 30% of ethyl acetate in hexane obtaining the 3-chloropyridine **12** (0.27 mmol, 79.6 mg, 79%) (Scheme S12).[15]

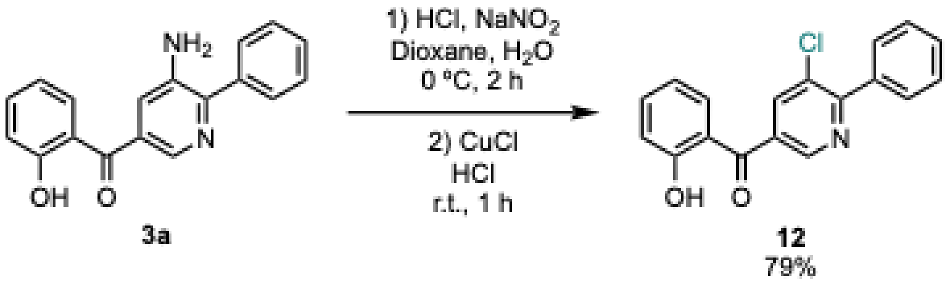

Scheme S12- Synthesis of the (5-chloro-6-phenylpyridin-3-yl)(2-hydroxyphenyl)methanone **12**.

8.3.4. 3-Chloropyridine – Characterization

(5-chloro-6-phenylpyridin-3-yl)(2-hydroxyphenyl)methanone (12)

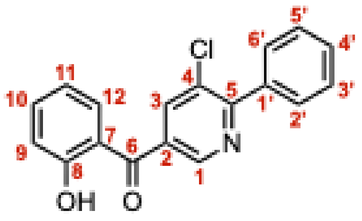

Light yellow solid. Melting point of 103.0–104.0 °C.

^{1}H NMR (300 MHz, DMSO-*d$_6$*): δ 10.59 (s, O*H*, 1H), 8.83 (d, *J* = 1.9 Hz, H-1, 1H), 8.20 (d, *J* = 1.9 Hz, H-3, 1H), 7.81 – 7.71 (m, H-2’ and 6’, 2H), 7.57 – 7.45 (m, H-10, 12, 3’, 4’ and 5’, 5H), 7.07 – 6.94 (m, H-9 and 11, 2H) ppm (Figure S110).

^{13}C NMR (75 MHz, DMSO-*d$_6$*): δ 194.1 (C-6), 158.2 (C-8), 157.3 (C-5), 148.0 (C-1), 138.3 (C-3), 137.2 (C-1’), 134.5 (C-10 or 12), 133.2 (C-2 or 4), 131.0 (C-10 or 12), 129.5 (C-4’), 129.4 (C-2’

and 6', 2C), 129.1 (C-2 or 4), 128.2 (C-3' and 5', 2C), 123.8 (C-7), 119.6 (C-11), 117.0 (C-9) ppm (Figure S111).

HRMS (ESI) ***m/z*:** $[M + H]^+$ Calcd for $C_{18}H_{13}ClNO_2^+$ 310.0629 ; Found 310.0623.

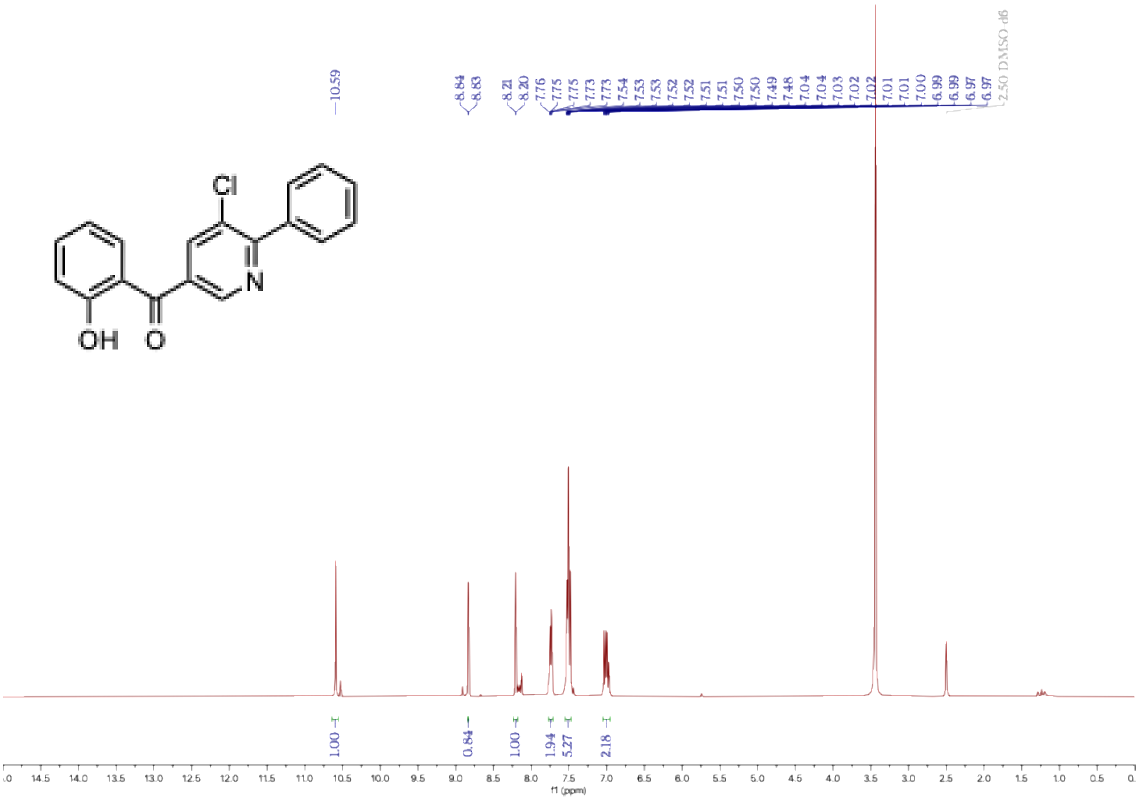

Figure S110 – ^{1}H-NMR (300 MHz, DMSO-d_6) of 3-chloropyridine **12**.

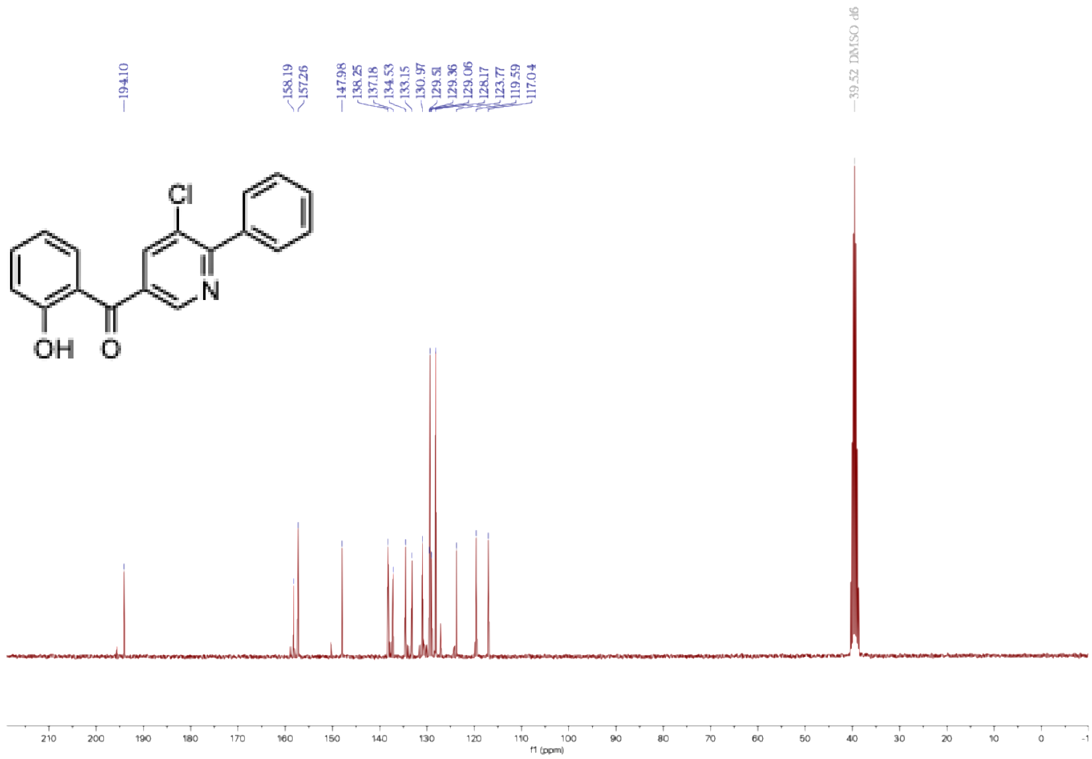

Figure S111 – ^{13}C-NMR (75 MHz, DMSO-*d₆*) of 3-chloropyridine **12**.

8.3.5. 3-Bromopyridine – Procedure

In a 25 mL round flask with (5-amino-6-phenylpyridin-3-yl)(2-hydroxyphenyl)methanone **3a** (1 equiv., 0.344 mmol, 100.0 mg) in acetonitrile (1 mL) was set to -10 °C. Then HBr 40% (0.5 mL) is added, followed by a solution of $NaNO_2$ (1.2 equiv., 0.413 mmol, 28.5 mg) in H_2O (0.5 mL). After 30 minutes, CuBr (1.5 equiv., 0.516 mmol, 74.0 mg) was added and the reaction mixture was let to stir for 16 h at room temperature. The reaction was quenched with iced water and neutralized with sodium carbonate till alkaline. The organic phase was extracted with DCM and the (5-bromo-6-phenylpyridin-3-yl)(2-hydroxyphenyl)methanone **13** (0.269 mmol, 95.3 mg, 78%) was purified with a gradient of 0% to 30% of ethyl acetate in hexane (Scheme S13).[16]

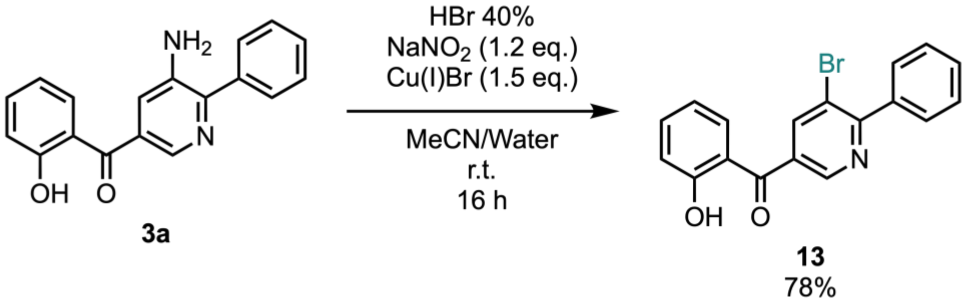

Scheme S13 - Synthesis of the (5-bromo-6-phenylpyridin-3-yl)(2-hydroxyphenyl)methanone **13**.

8.3.6. 3-Bromopyridine – Characterization

(5-bromo-6-phenylpyridin-3-yl)(2-hydroxyphenyl)methanone (13)

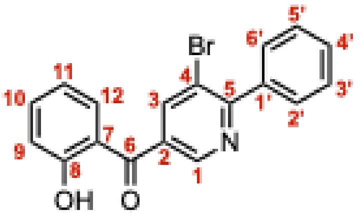

13

Yellow solid. Melting point of 120.9–123.8 °C.

^{1}H NMR (300 MHz, DMSO-*d$_6$*): δ 10.60 (s, O*H*, 1H), 8.86 (d, H-1, *J* = 1.8 Hz, 1H), 8.35 (d, *J* = 1.8 Hz, H-3, 1H), 7.73 – 7.65 (m, H-2’ and 6’, 2H), 7.55 – 7.46 (m, H-10, 12, 3’, 4’ and 5’, 5H), 7.05 – 6.96 (m, H-9 and 11, 2H) ppm (Figure S112).

^{13}C NMR (75 MHz, DMSO-*d$_6$*): δ 194.0 (C-6), 159.9 (C-5), 157.2 (C-8), 148.4 (C-1), 141.4 (C-3), 138.6 (C-1’), 134.5 (C-10), 133.2 (C-2), 130.9 (C-12), 129.3 (C-4’), 129.3 (C-2’ and 6’, 2C), 128.1 (C-3’ and 5’, 2C), 123.8 (C-7), 119.6 (C-11), 118.8 (C-4), 117.0 (C-9) ppm (Figure S113).

HRMS (ESI) *m/z*: $[M + H]^+$ Calcd for $C_{18}H_{13}BrNO_2^+$ 354.0124 ; Found 354.0120.

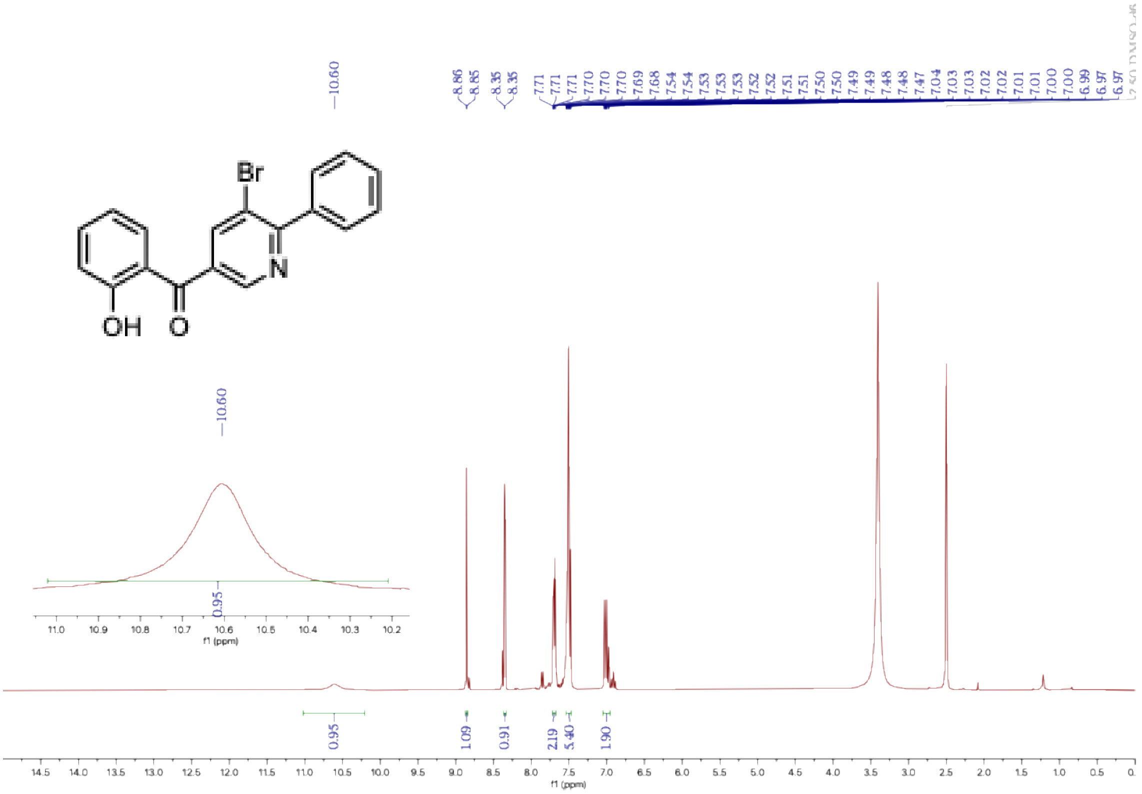

Figure S112 – ^{1}H-NMR (300 MHz, DMSO-d_6) of 3-bromopyridine **13**.

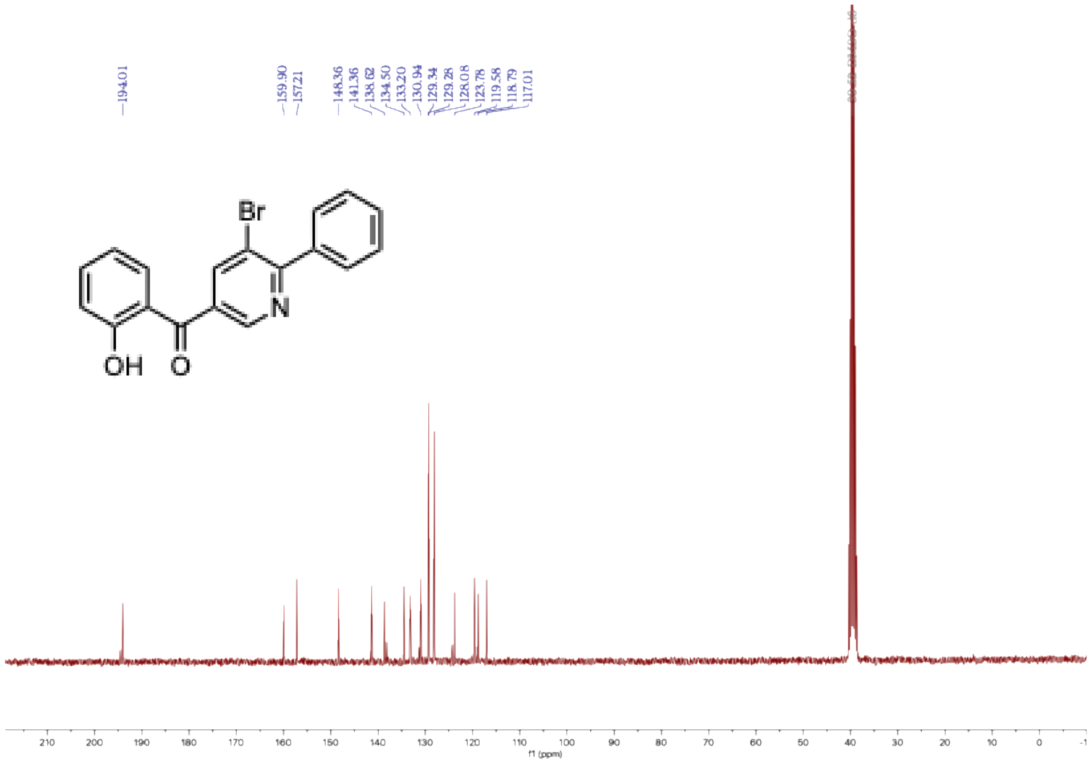

Figure S113 – ^{13}C-NMR (75 MHz, DMSO-d_6) of 3-bromopyridine **13**.

8.3.7. 3-Iodopyridine – Procedure

In a 25 mL round bottom flask cooled to 0 °C, the (5-amino-6-phenylpyridin-3-yl)(2-hydroxyphenyl)methanone **3a** (1 equiv., 0.34 mmol, 100.0 mg) was added into a solution of hydrochloric acid 4 M (2 mL). Then, a solution of $NaNO_2$ (1.2 equiv., 0.41 mmol, 28.2 mg) in H_2O (1 mL) was slowly added for 30 minutes. Afterwards, the reaction mixture was let to stir for 30 minutes at 0 °C. The temperature of the reaction mixture was set to -10 °C and a solution of KI (5 equiv., 1.7 mmol, 282.2 mg) in H_2O (3 mL) was added. The reaction was let to react at room temperature for 4 hours. The reaction was stopped with a concentrated solution of sodium bicarbonate. The resultant precipitate was washed with water to obtain the 3-iodopyridine **14** (0.331 mmol, 132.8 mg, 96%).[17]

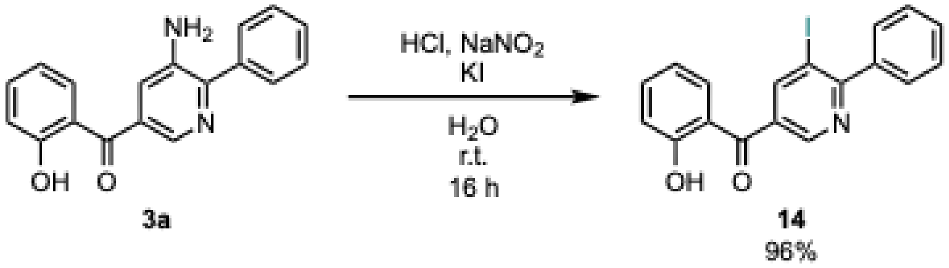

Scheme S14 - Synthesis of the (2-hydroxyphenyl)(5-iodo-6-phenylpyridin-3-yl)methanone **14**.

8.3.8. 3-Iodopyridine – Characterization

(2-hydroxyphenyl)(5-iodo-6-phenylpyridin-3-yl)methanone (14)

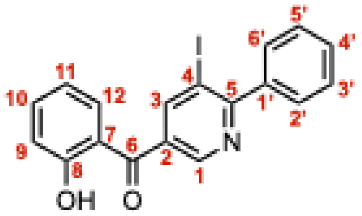

14

Dark orange solid. Melting point of 105.2–106.6 °C.

^{1}**H NMR (300 MHz, CDCl$_3$):** δ 11.77 (s, O*H*, 1H), 8.90 (d, *J* = 1.9 Hz, H-1, 1H), 8.57 (d, *J* = 1.9 Hz, H-3, 1H), 7.70 – 7.47 (m, H-10, 12, 2', 3', 4', 5' and 6', 7H), 7.12 (dd, *J* = 8.4, 1.1 Hz, H-1,2 1H), 6.97 (ddd, *J* = 8.3, 7.2, 1.2 Hz, H-9, 1H) ppm (Figure S114).

^{13}**C NMR (75 MHz, CDCl$_3$):** δ 197.4 (C-6), 164.3 (C-5), 163.5 (C-8), 148.6 (C-1), 148.2 (C-3), 141.1 (C-1'), 137.5 (C-12), 133.0 (C-10), 133.0 (C-2), 129.6 (C-4'), 129.4 (C-2' and 6' or 3' and 5', 2C), 128.3 (C-2' and 6' or 3' and 5', 2C), 119.5 (C-9), 119.0 (C-11), 119.0 (C-7), 93.7 (C-4) ppm (Figure S115).

HRMS (ESI) ***m/z*****:** [M + H]$^+$ Calcd for $C_{18}H_{13}INO_2^+$ 401.9986 ; Found 401.9981.

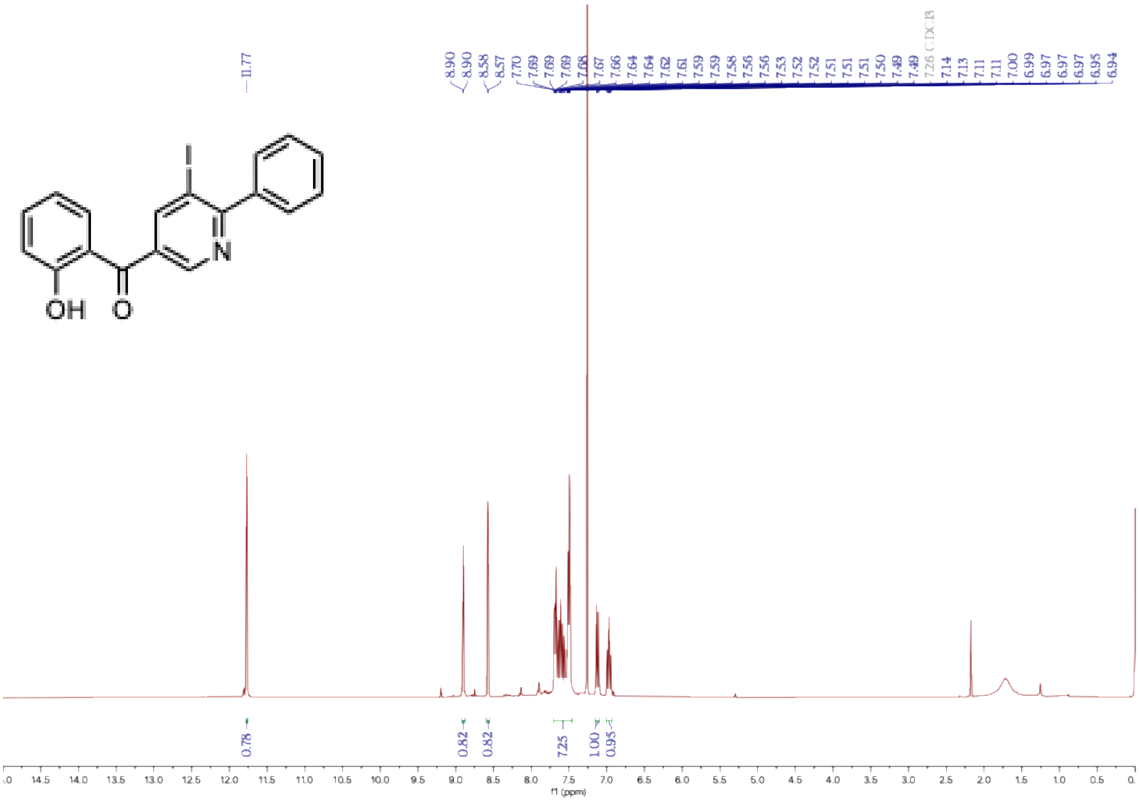

Figure S114 – ^{1}H-NMR (300 MHz, CDCl$_3$) of 3-iodopyridine **14**.

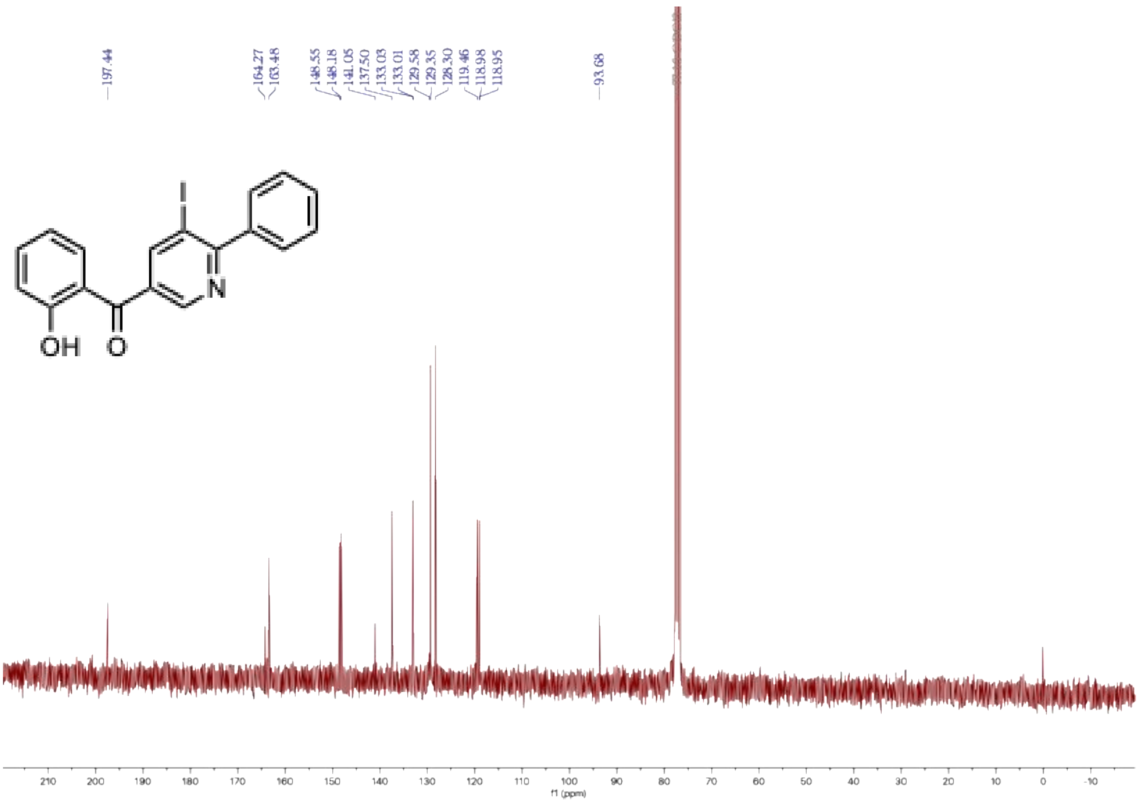

Figure S115 – ^{13}C-NMR (75 MHz, $CDCl_3$) of 3-iodopyridine **14**.

8.4. Azido Pyridine Synthesis

8.4.1. Procedure

A 25 mL round bottom flask with the (5-amino-6-phenylpyridin-3-yl)(2-hydroxyphenyl)methanone **3a** (1 equiv., 0.34 mmol, 100.0 mg) in acetonitrile (2 mL) was set to 0 °C, then *t*BuONO (1.2 equiv., 0.41 mmol, 48.8 μL) was added. Following, trimethylsilyl azide ($TMSN_3$) (1.2 equiv., 0.41 mmol, 54.4 μL) was added dropwise. After the reaction mixture reached room temperature, it was let to react for 2 hours. Then, the crude was concentrated under reduced pressure and purified with column chromatography with a gradient of 0% to 30% of ethyl acetate in hexane obtaining the 3-azidepyridine **15** (0.332 mmol, 105.0 mg, 97%) (Scheme S15).[18]

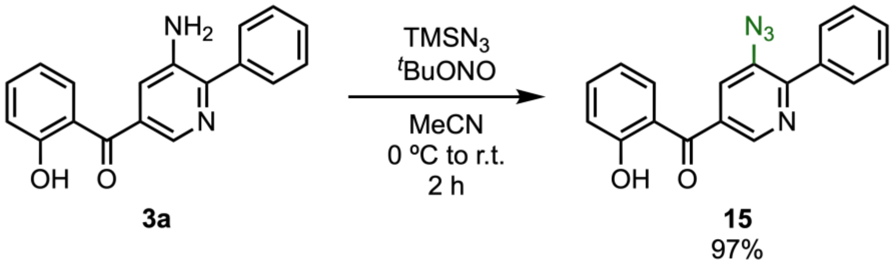

Scheme S15 - Synthesis of the (5-azido-6-phenylpyridin-3-yl)(2-hydroxyphenyl)methanone **15**.

8.4.2. Characterization

(5-azido-6-phenylpyridin-3-yl)(2-hydroxyphenyl)methanone (15)

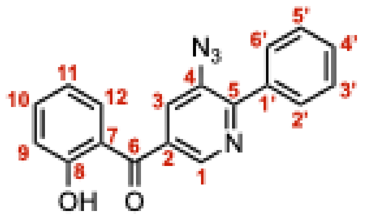

15

Yellow solid. Melting point of 98.9–100.8 °C.

^{1}H NMR (300 MHz, $CDCl_3$): δ 11.80 (s, O*H*, 1H), 8.77 – 8.71 (m, H-1, 1H), 7.94 – 7.88 (m, H-2’ and 6’, 2H), 7.86 (d, *J* = 1.7 Hz, H-3, 1H), 7.65 (dd, *J* = 8.1, 1.7 Hz, H-12, 1H), 7.57 (ddd, *J* = 8.6, 7.2, 1.7 Hz, H-10, 1H), 7.53 – 7.46 (m, H-3’, 4’ and 5’, 3H), 7.11 (dd, *J* = 8.6, 1.1 Hz, H-9, 1H), 6.95 (ddd, *J* = 8.1, 7.2, 1.1 Hz, H-11, 1H) ppm (Figure S116).

^{13}C NMR (75 MHz, $CDCl_3$): δ 197.9 (C-6), 163.3 (C-8), 152.8 (C-5), 145.5 (C-1), 137.3 (C-12), 136.3 (C-1’), 134.7 (C-2 or 4), 132.9 (C-10), 132.8 (C-2 or 4), 129.7 (C-4’), 129.6 (C-2’ and 6’, 2C), 128.3 (C-3’ and 5’, 2C), 127.1 (C-3), 119.3 (C-11), 118.9 (C-7), 118.8 (C-9) ppm (Figure S117).

HRMS (ESI) *m/z*: $[M + H]^+$ Calcd for $C_{18}H_{13}N_4O_2^+$ 317.1033 ; Found 317.1030.

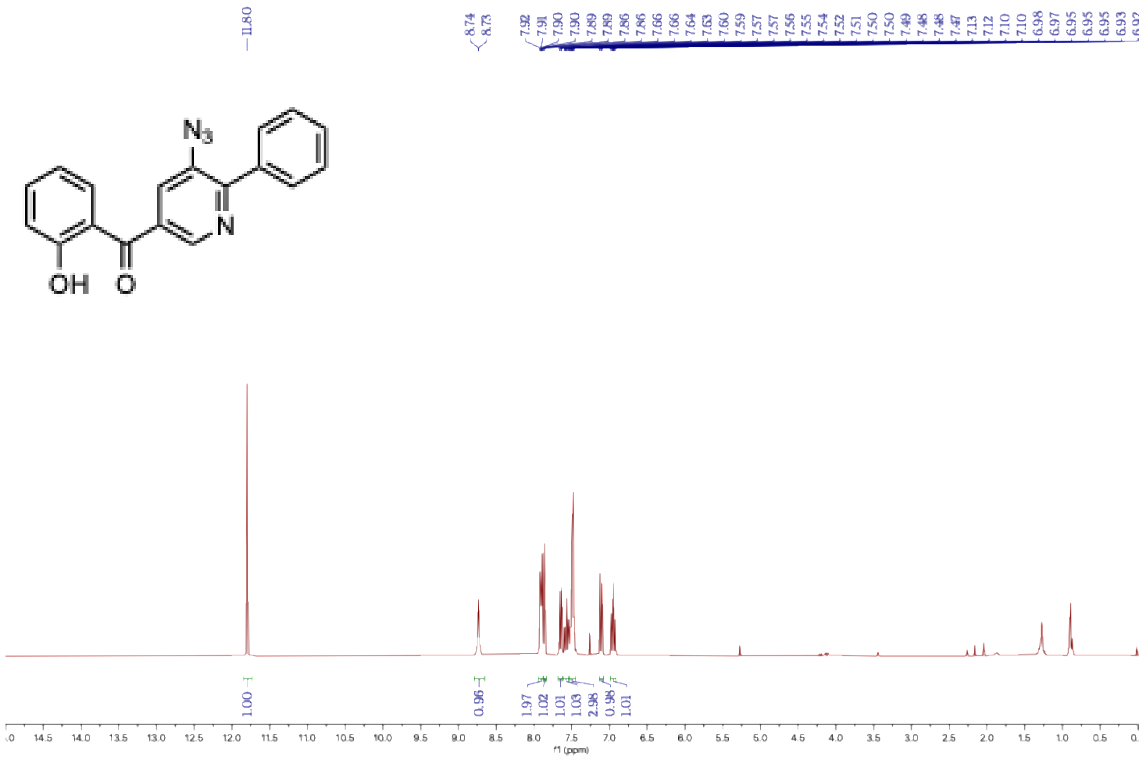

Figure S116 – ^{1}H-NMR (300 MHz, $CDCl_3$) of 3-azidopyridine **15**.

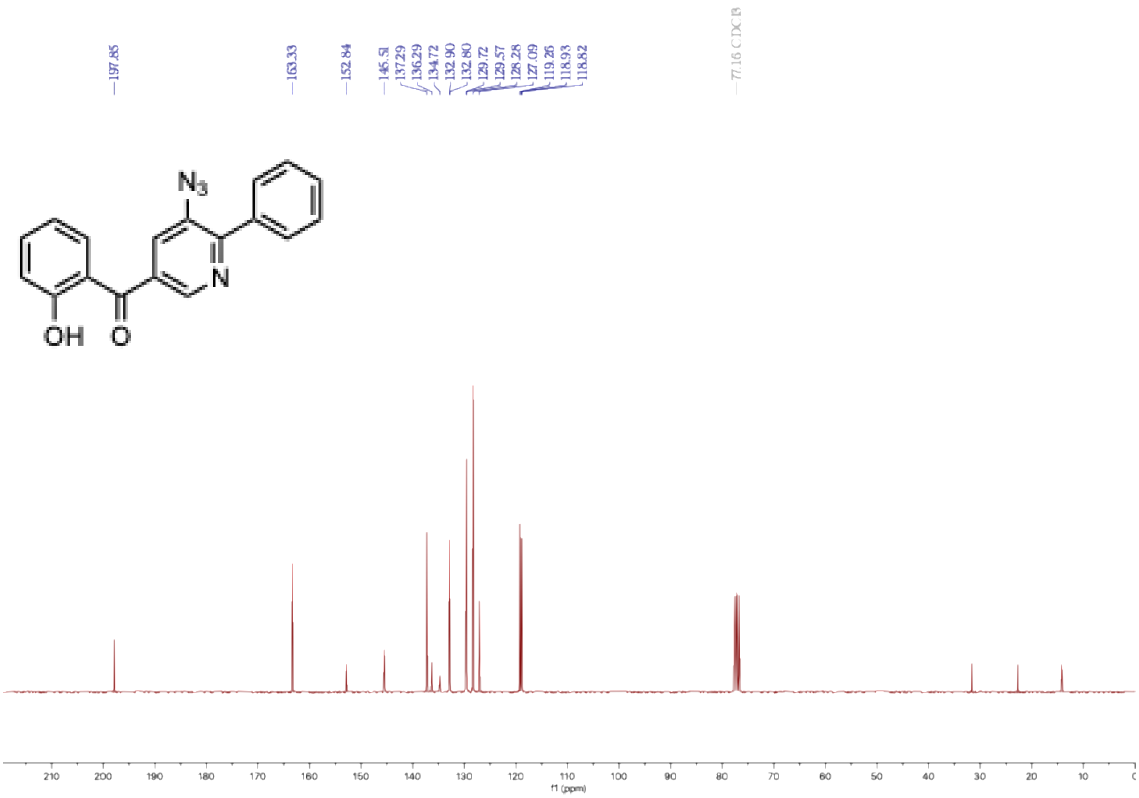

Figure S117 – ^{13}C-NMR (75 MHz, $CDCl_3$) of 3-azidopyridine **15**.

8.5. 4-Azacarbazole

8.5.1. Procedure

In a Schlenk tube, (5-amino-6-phenylpyridin-3-yl)(2-hydroxyphenyl)methanone **3a** (1 equiv., 0.344 mmol, 100.0 mg) was added into DCM (2 mL). The mixture was set to 0 °C and ^{t}BuONO (1.2 equiv., 0.413 mmol, 49.2 μL) and $TMSN_3$ (1.2 equiv., 0.413 mmol, 54.8 μL) were added dropwise. The mixture was let to react till all the components become completely dissolved (around 2 hours) at room temperature. Then, silica gel (34.0 mg) and H_2O (2 mL) were added, and the reaction was set under 370 nm LED-irradiation (Kessil PR160L Gen 2) for 36 h. After the reaction was stopped, the organic phase was extracted with DCM and dried with sodium sulfate. The (2-hydroxyphenyl)(5*H*-pyrido[3,2-*b*]indol-3-yl)methanone **16** (0.158 mmol, 45.6 mg, 46%) was purified by column chromatography with a gradient of 0% to 50% of ethyl acetate in hexane (Scheme S16).(Adapted from ref [19])

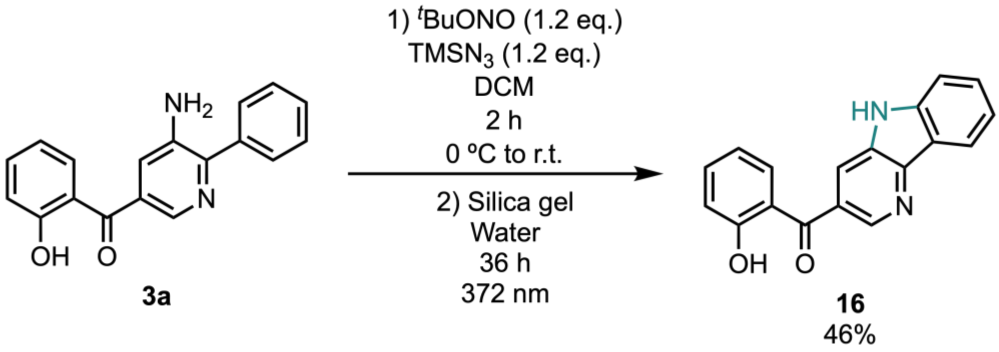

Scheme S16 -Synthesis of the (2-hydroxyphenyl)(5*H*-pyrido[3,2-*b*]indol-3-yl)methanone **16**.

8.5.2. Characterization

(2-hydroxyphenyl)(5*H*-pyrido[3,2-b]indol-3-yl)methanone (16)

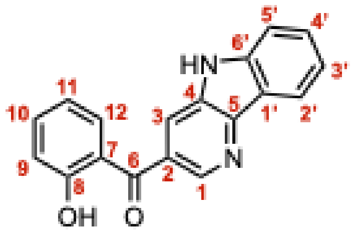

Yellow solid. Melting point of 146.7–148.2 °C.

^{1}H NMR (300 MHz, DMSO-*d$_6$*): δ 11.67 (s, N*H*, 1H), 10.40 (s, O*H*, 1H), 8.78 (d, *J* = 1.9 Hz, H-1, 1H), 8.28 – 8.23 (m, H-2', 1H), 8.15 (d, *J* = 1.9 Hz, H-3, 1H), 7.68 – 7.55 (m, H-4' and 5', 2H), 7.53

– 7.43 (m, H-10 and 12, 2H), 7.31 (ddd, *J* = 8.0, 6.6, 1.4 Hz, H-3', 1H), 7.05 – 6.95 (m, H-9 and 11, 2H) ppm (Figure S118).

^{13}C NMR (75 MHz, DMSO-*d$_6$*): δ 196.5 (C-6), 156.8 (C-8), 143.7 (C-5), 142.5 (C-1), 142.3 (C-6'), 133.4 (C-10), 131.9 (C-4), 130.6 (C-12), 129.4 (C-2), 129.0 (C-4'), 125.1 (C-7), 120.9 (C-2'), 120.8 (C-1'), 120.1 (C-3'), 119.3 (C-11), 119.0 (C-3), 116.8 (C-9), 112.2 (C-5') ppm (Figure S119).

HRMS (ESI) *m/z*: [M + H]$^+$ Calcd for $C_{18}H_{13}N_2O_2^+$ 289.0972 ; Found 289.0967.

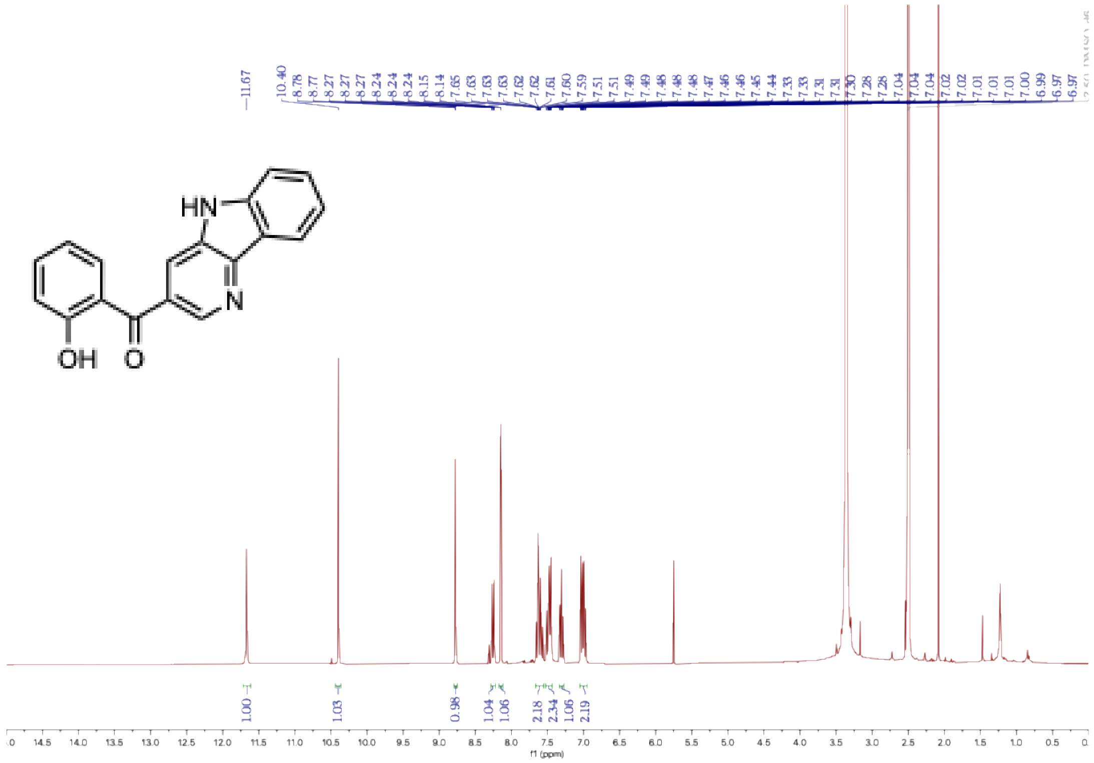

Figure S118 – ^{1}H-NMR (300 MHz, DMSO-*d$_6$*) of 4-azacarbazole **16**.

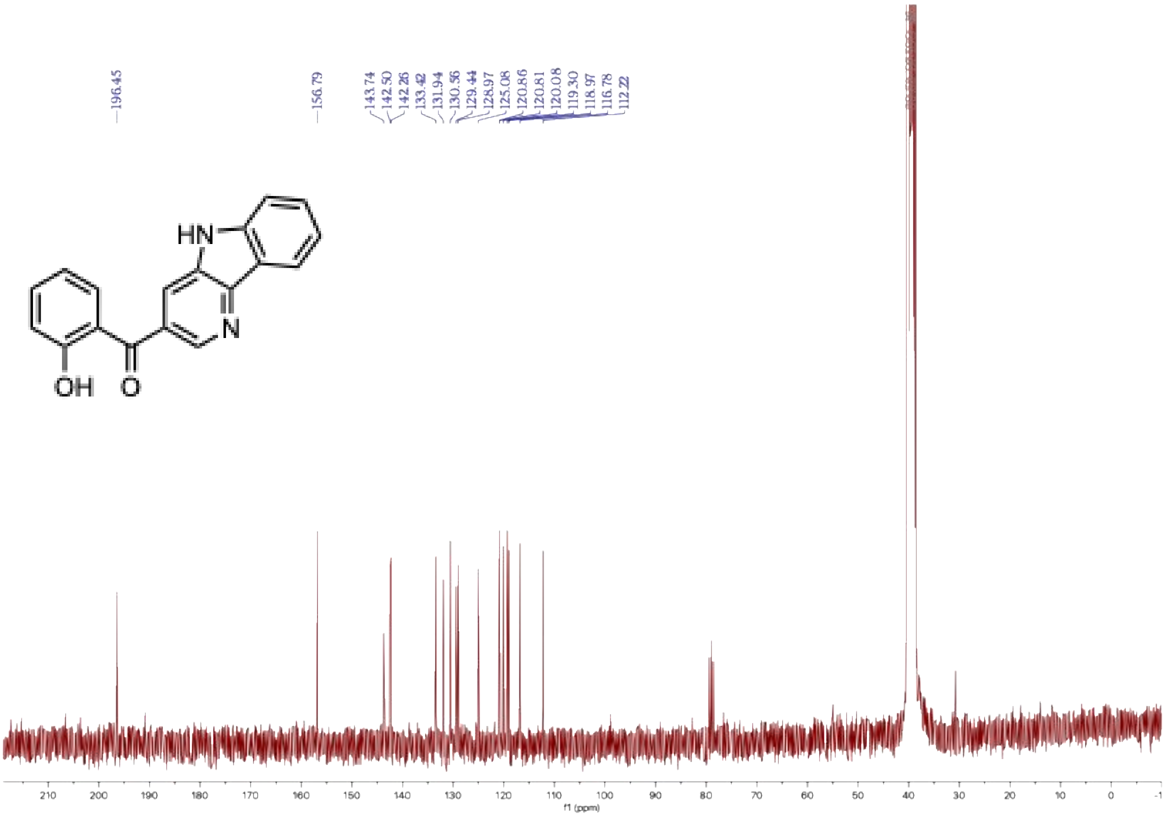

Figure S119 – ^{13}C-NMR (75 MHz, DMSO-*d$_6$*) of 4-azacarbazole **16**.

8.6. Hydroxypyridine Synthesis

8.6.1. Procedure

In a 25 mL round bottom flask with the (5-amino-6-phenylpyridin-3-yl)(2-hydroxyphenyl)methanone **3a** (1 equiv., 0.34 mmol, 100.0 mg) in H_2SO_4 2% (5 mL) at 0 °C, a solution of $NaNO_2$ (1.3 equiv., 0.44 mmol, 30.4 mg) in H_2O (0.5 mL) was added dropwise. The reaction was let to stir for 30 minutes at room temperature and then an extra 30 min at reflux. The reaction mixture was cooled to room temperature and the organic phase was extracted with ethyl acetate. The crude was purified through a column chromatography with a gradient of 0% to 30% of ethyl acetate in hexane obtaining the 3-hydroxypyridine **17** (0.139 mmol, 40.6 mg, 41%) (Scheme S17).(Adapted from ref [20])

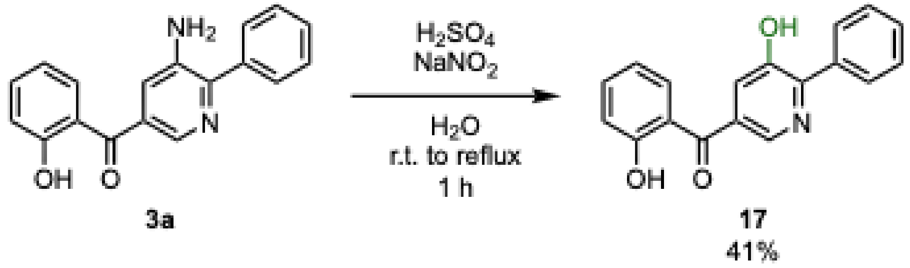

Scheme S17 - Synthesis of the (5-hydroxy-6-phenylpyridin-3-yl)(2-hydroxyphenyl)methanone **17**.

8.6.2. Characterization

(5-hydroxy-6-phenylpyridin-3-yl)(2-hydroxyphenyl)methanone (17)

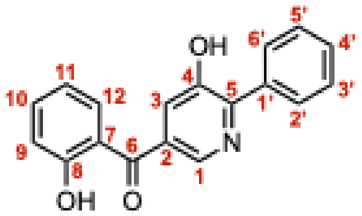

17

Light yellow solid. Melting point of 210.2–211.9 °C.

^{1}H NMR (300 MHz, DMSO-*d$_6$*): δ 10.60 (s, 8-O*H*, 1H), 10.42 (s, 4-O*H*, 1H), 8.43 (d, *J* = 1.9 Hz, H-1, 1H), 8.12 – 8.05 (m, H-2' and 6', 2H), 7.63 (d, *J* = 1.9 Hz, H-3, 1H), 7.51 – 7.38 (m, H-10, 12, 3' 4' and 5', 5H), 7.03 – 6.95 (m, H-9 and 11, 2H) ppm (Figure S120).

^{13}C NMR (75 MHz, DMSO-*d$_6$*): δ 195.7 (C-6), 156.9 (C-8), 151.3 (C-4), 147.7 (C-5), 140.8 (C-1), 137.1 (C-1'), 133.6 (C-10), 132.5 (C-2), 130.4 (C-12), 129.1 (C-2' and 6', 2C), 128.7 (C-4'), 127.9 (C-3' and 5'), 124.6 (C-7), 123.4 (C-3), 119.3 (C-11), 116.8 (C-9) ppm (Figure S121).

HRMS (ESI) *m/z*: $[M + H]^+$ Calcd for $C_{18}H_{14}NO_3^+$ 292.0968 ; Found 292.0964.

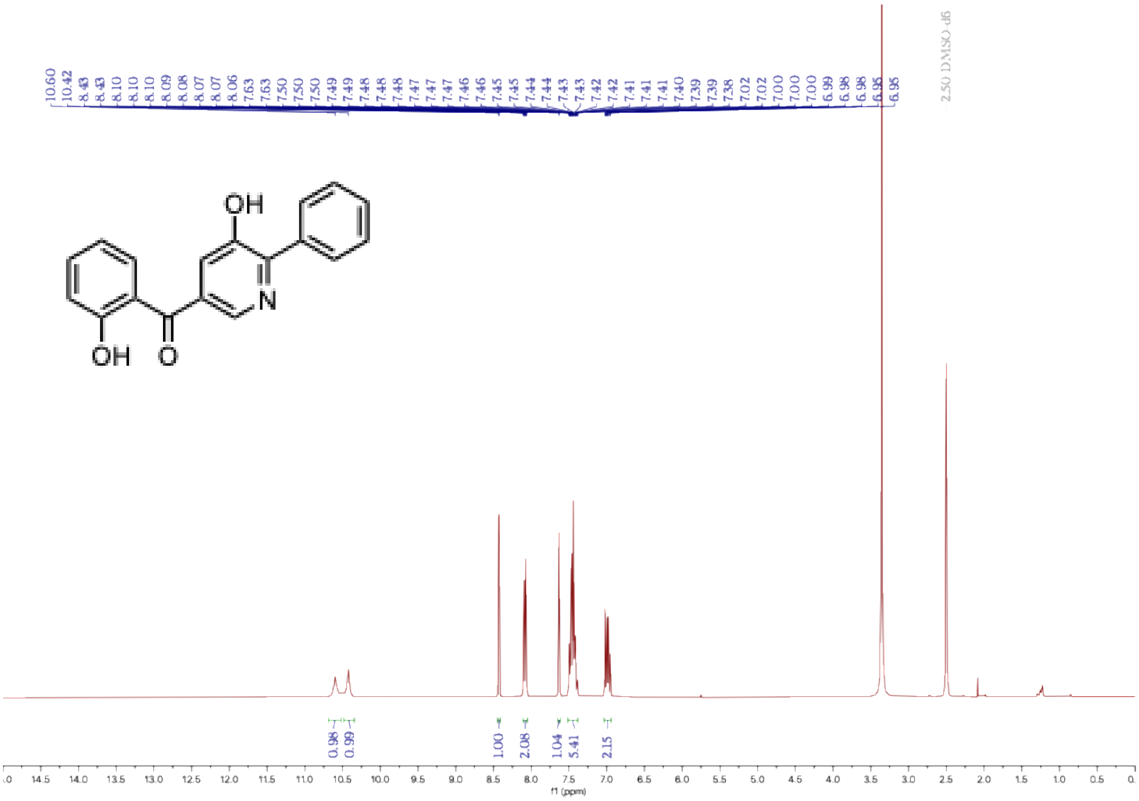

Figure S120 – ^{1}H-NMR (300 MHz, DMSO-d_6) of 3-hydroxypyrdine **17**.

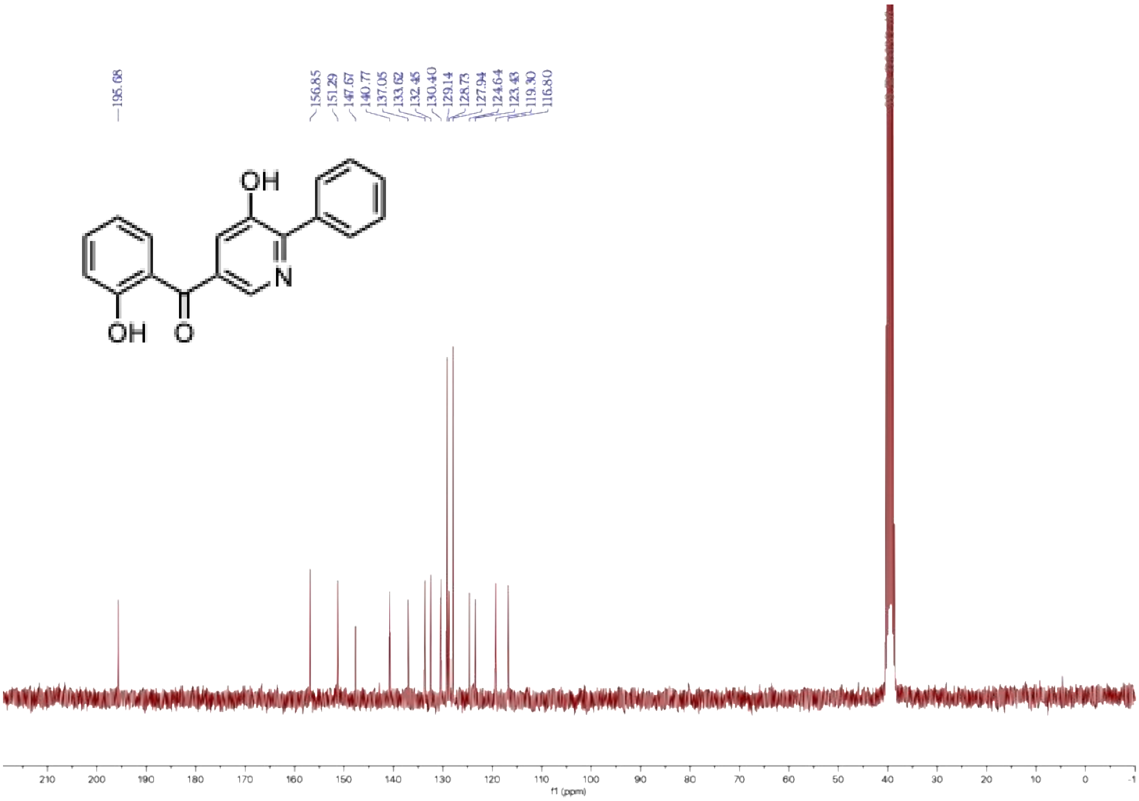

Figure S121 – ^{13}C-NMR (75 MHz, DMSO-d_6) of 3-hydroxypyrdine **17**.

8.7. Pyridine-3-Sulfinic Acid Synthesis

8.7.1. Pyridinyl Carbonodithioate – Procedure

In a Schlenk flask, (5-amino-6-phenylpyridin-3-yl)(2-hydroxyphenyl)methanone **3a** (1 equiv., 0.344 mmol, 100.0 mg) and $NaNO_2$ (1.2 equiv, 0.413 mmol, 28.3 mg) were added into 1 mL of H_2O and cooled to 0 °C. Then, 0.3 mL of HCl (35 %) was added dropwise, and the reaction was stirred for 30 minutes. Separately, KSSCOEt (3 equiv., 1.02 mmol, 163.5 mg) was dissolved in 2 mL of H_2O and added dropwise into the reaction mixture. The mixture was then heated to 65 °C for 30 minutes. After cooling to room temperature, a solution of 10% of sodium hydroxide was added and stirred for 5 minutes. The organic phase was extracted with ethyl acetate, dried with sodium sulfate and concentrated under reduced pressure. The *O*-ethyl *S*-[5-(2-hydroxybenzoyl)-2-phenylpyridin-3-yl] carbonodithioate **18** (0.149 mmol, 58.8 mg, 43%) was purified through column chromatography with a gradient from 0 to 40% of ethyl acetate in hexane (Scheme S18).(Adapted from ref [21]).

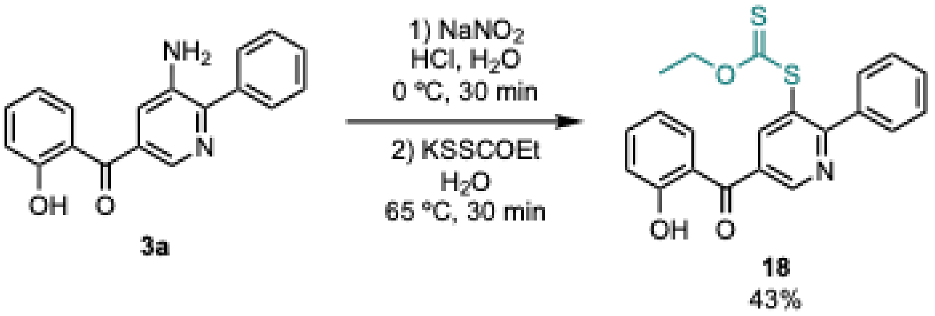

Scheme S18 -Synthesis of the *O*-ethyl *S*-[5-(2-hydroxybenzoyl)-2-phenylpyridin-3-yl] carbonodithioate **18**.

8.7.2. Pyridinyl Carbonodithioate – Characterization

***O*-ethyl *S*-[5-(2-hydroxybenzoyl)-2-phenylpyridin-3-yl] carbonodithioate (18)**

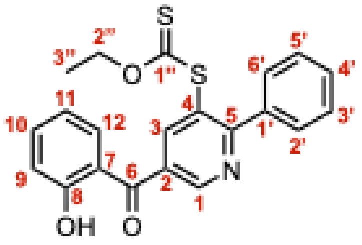

18

Yellow oil.

^{1}**H NMR (300 MHz, $CDCl_3$):** δ 11.81 (s, O*H*, 1H), 9.04 (d, *J* = 2.1 Hz, H-1, 1H), 8.24 (d, *J* = 2.1 Hz, H-3, 1H), 7.70 – 7.64 (m, H-12, 2' and 6', 3H), 7.57 (ddd, *J* = 8.6, 7.2, 1.9 Hz, H-10, 1H), 7.49 – 7.44

(m, H-3’, 4’ and 5’, 3H), 7.11 (dd, *J* = 8.6, 1.2 Hz, H-9, 1H), 6.95 (ddd, *J* = 8.1, 7.2, 1.2 Hz, H-11, 1H), 4.55 (q, *J* = 7.1 Hz, H-2’’, 2H), 1.32 (t, *J* = 7.1 Hz, H-3’’, 3H) ppm (Figure S122).

^{13}C NMR (75 MHz, $CDCl_3$): δ 210.7 (C-1’’), 198.0 (C-6), 164.5 (C-5), 163.4 (C-8), 150.5 (C-1), 146.3 (C-3), 138.4 (C-1’), 137.4 (C-10), 133.1 (C-12), 132.4 (C-12), 129.6 (C-4’), 129.5 (C-2’ and 6’, 2C), 128.2 (C-3’ and 5’, 2C), 125.9 (C-4), 119.4 (C-11), 119.0 (C-7), 118.9 (C-9), 71.1 (C-2’’), 13.7 (C-3’’) ppm (Figure S123).

HRMS (ESI) *m/z*: $[M + H]^+$ Calcd for $C_{21}H_{18}NO_3S_2^+$ 396.0723 ; Found 396.0716.

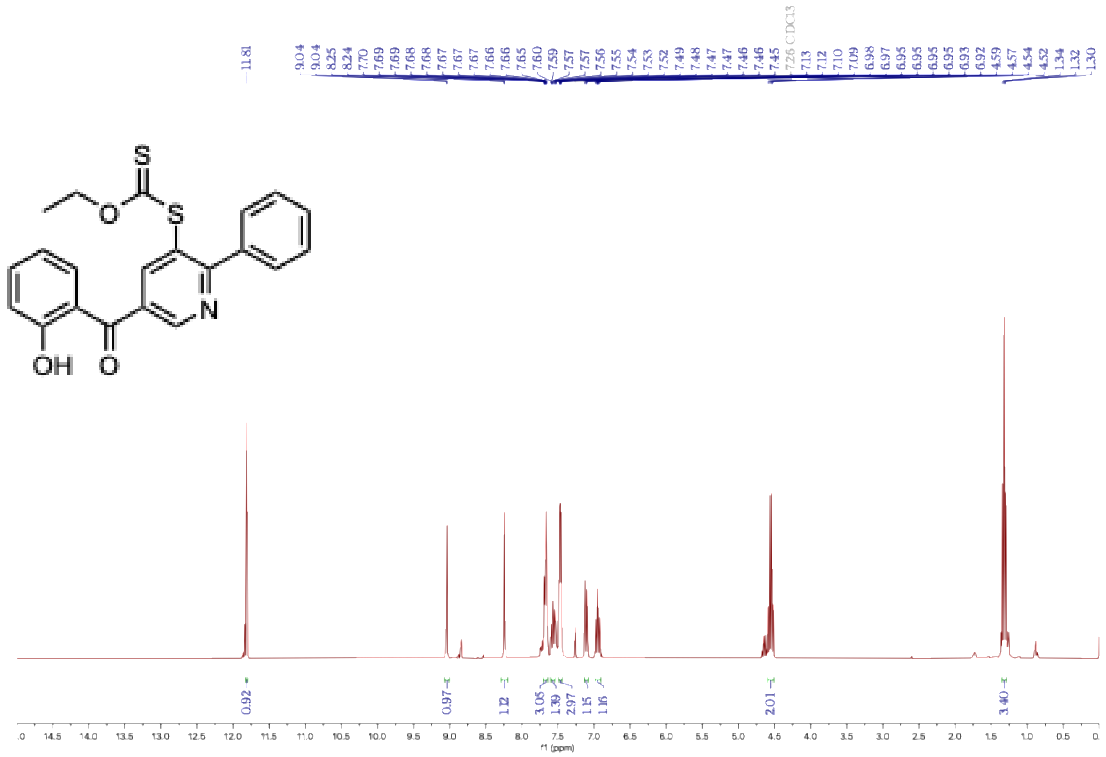

Figure S122 – ^{1}H-NMR (300 MHz, $CDCl_3$) of pyridinyl carbonodithioate **18**.

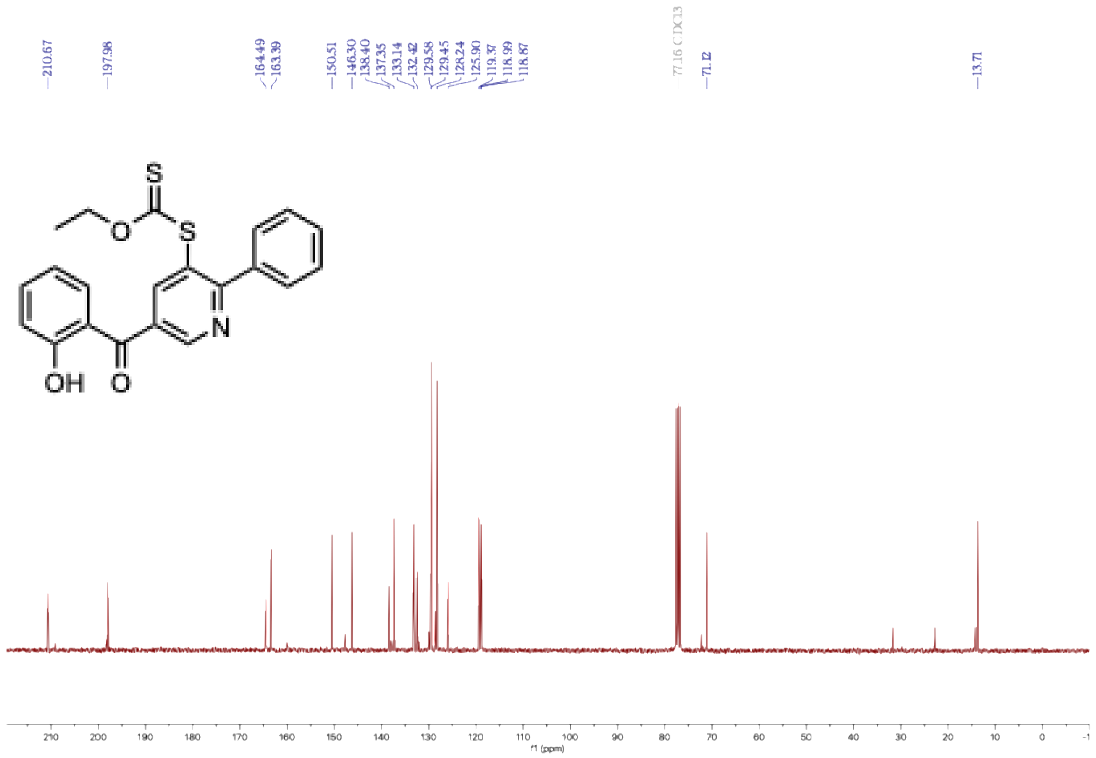

Figure S123 – ^{13}C-NMR (75 MHz, $CDCl_3$) of pyridinyl carbonodithioate **18**.

8.7.3. Pyridine-3-Sulfinic Acid – Procedure

In a Schlenk, pyridinyl carbonodithioate **19** (1 equiv., 0.136 mmol, 53.9 mg) was added into a mixture of EtOH (1 mL) and an aqueous solution of NaOH (3M, 0.5 mL). The reaction mixture was stirred for 3 hours at 65 °C. After cooling to room temperature, the organic phase was extracted with ethyl acetate, dried with sodium sulfate and concentrated under reduced pressure. The 5-(2-hydroxybenzoyl)-2-phenylpyridine-3-sulfinic acid **19** (0.106 mmol, 32.7 mg, 71%) was purified through column chromatography with a gradient from 0 to 40% of ethyl acetate in hexane (Scheme S19). (Adapted from ref [22])

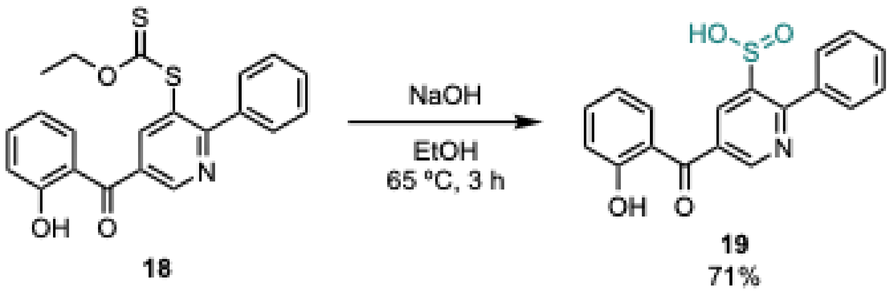

Scheme S19 -Synthesis of the 5-(2-hydroxybenzoyl)-2-phenylpyridine-3-sulfinic acid **19**.

8.7.4. Pyridine-3-Sulfinic Acid– Characterization

5-(2-hydroxybenzoyl)-2-phenylpyridine-3-sulfinic acid (19)

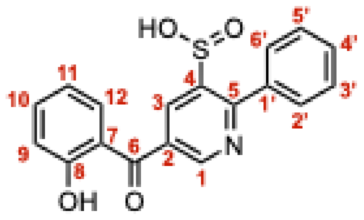

19

Light yellow solid. Melting point of 150.0–152.0 °C.

^{1}H NMR (500 MHz, $CDCl_3$): δ 11.82 (s, O*H*, 1H), 8.79 (s, H-1, 1H), 8.13 (d, *J* = 1.8 Hz, H-3, 1H), 7.63 – 7.50 (m, H-10, 2' and 6', 3H), 7.50 – 7.37 (m, H-12, 3', 4' and 5', 4H), 7.12 (dd, *J* = 8.4, 1.1 Hz, H-9, 1H), 6.81 (ddd, *J* = 8.2, 7.2, 1.1 Hz, H-11, 1H) (Figure S124).

^{13}C NMR (126 MHz, $CDCl_3$) δ: 198.2 (C-6), 163.5 (C-8), 160.0 (C-5), 147.6 (C-1), 137.7 (C-1'), 137.4 (C-10), 136.1 (C-3), 132.9 (C-12), 132.8 (C-2 or 4), 131.6 (C-2 or 4), 130.2 (C-4'), 129.2 (C-3' and 5', 2C), 128.7 (C-2' and 6', 2C), 119.3 (C-11), 119.0 (C-7 and 9, 2C) (Figure S125 and Figure S126).

IR (neat) $_{max}$: 3053, 2923, 2851, 2250, 2232, 1955, 1813, 1715, 1625, 1606, 1574 cm^{-1}.

HRMS (ESI) ***m/z***: $[M + H]^+$ Calcd for $C_{18}H_{14}NO_4S^+$ 340.0638 ; Found 340.0638.

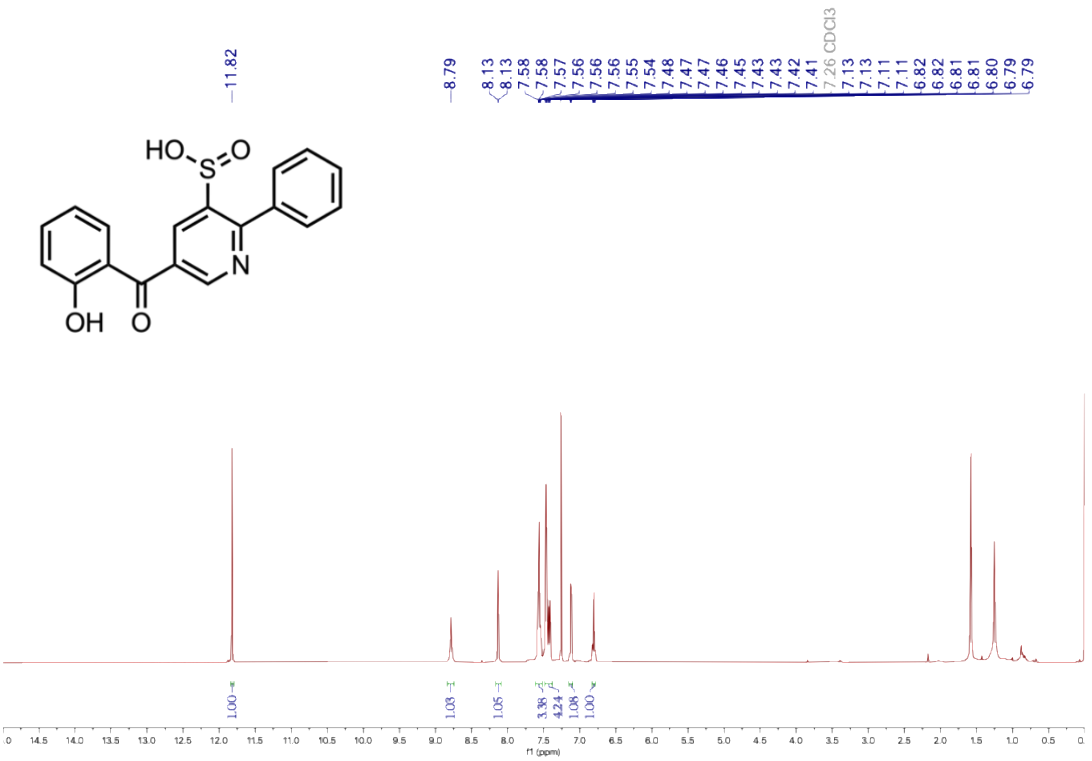

Figure S124 – ^{1}H-NMR (500 MHz, $CDCl_3$) of pyridine-3-sulfinic acid **19**.

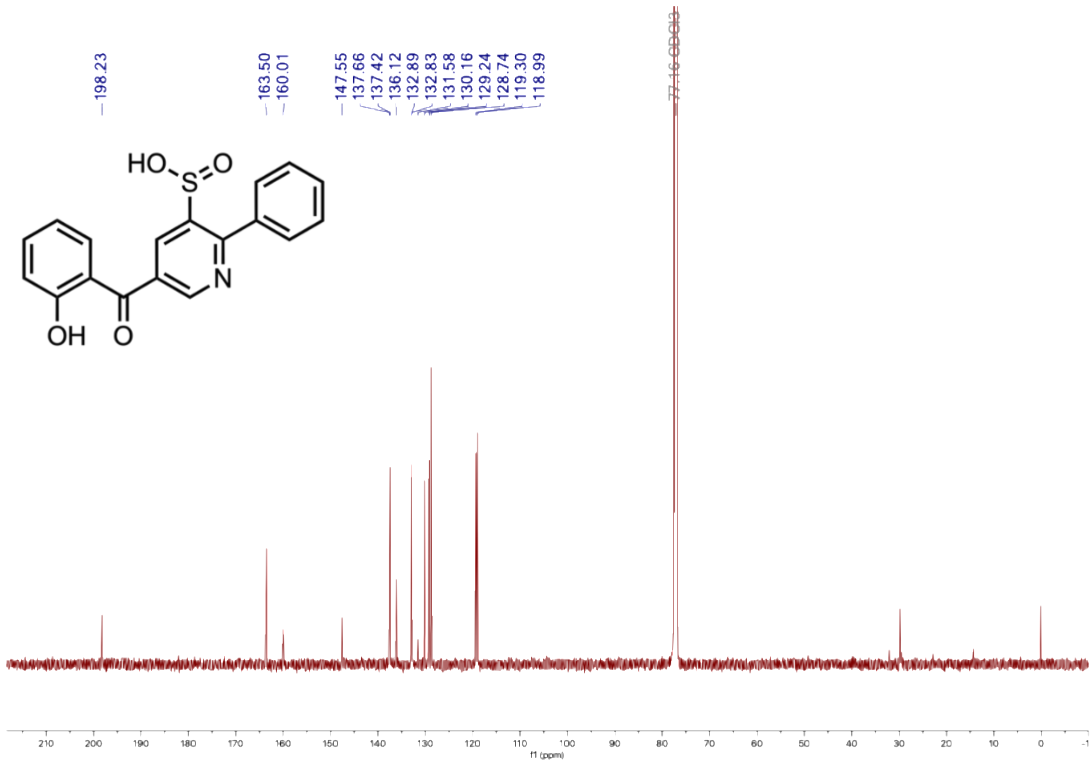

Figure S125 – ^{13}C-NMR (126 MHz, $CDCl_3$) of pyridine-3-sulfinic acid **20**.

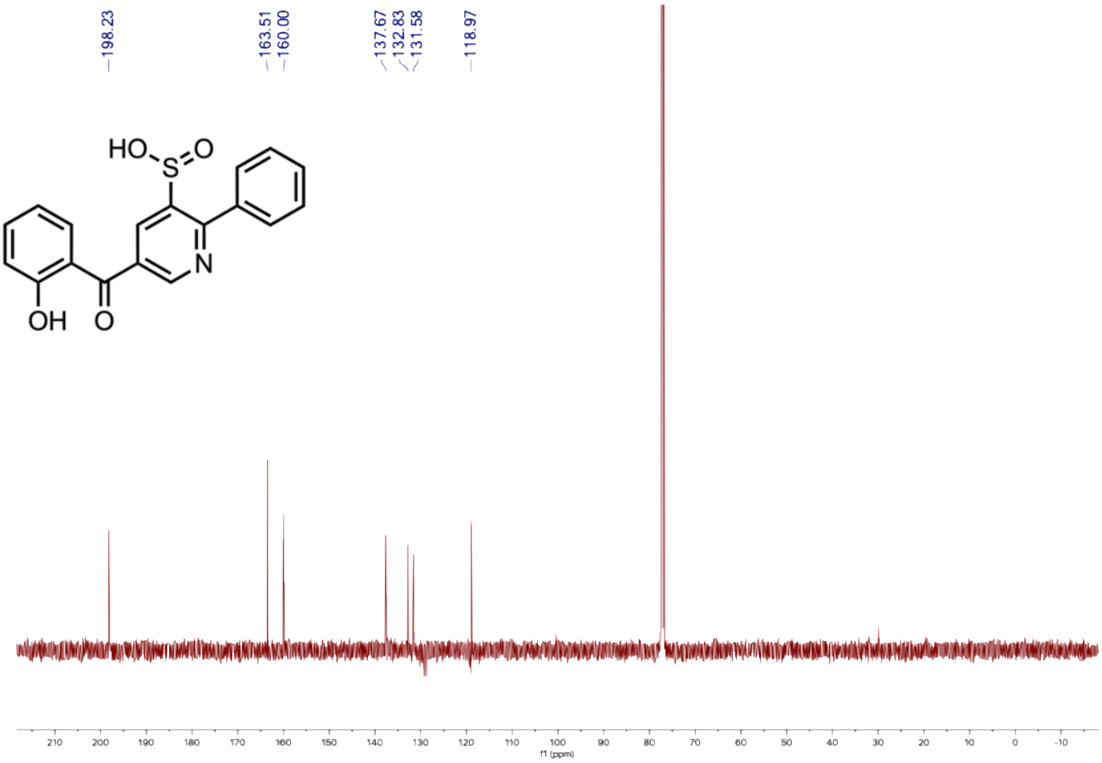

Figure S126 – DEPTQ-135 with only quaternary carbons of 3-thiopyridine **19**.

9. Synthesis of 3-Formylchromones

9.1. General Procedure

In a round flask, the respective 2-hydroxyacetophenone (1 equiv.) was added into DMF (1.3 M). The reaction mixture was set to 0 °C, and the phosphorus oxychloride ($POCl_3$) (5 equiv.) was added dropwise. The reaction mixture was then allowed to reach room temperature, and it was let to react for 16 h. At the end of the reaction, the mixture was set to 0 °C and H_2O was added to the mixture. Resultant precipitate was filtered under reduced pressure and washed with water (Scheme S20).

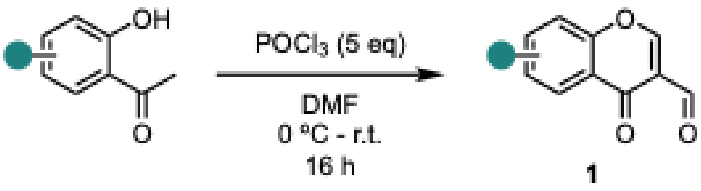

Scheme S20 - General procedure for the synthesis of 3-formylchromones **1**.

9.2. Characterization

4-oxo-4*H*-benzo[*h*]chromene-3-carbaldehyde (1j)

Following the general procedure using 1-(1-hydroxynaphthalen-2-yl)ethan-1-one (2.68 mmol, 500.0 mg) and $POCl_3$ (13.4 mmol, 1.2 mL) in 2 mL of DMF. After filtration, the compound **1i** (2.63 mmol, 589.3 mg, 98% yield) was obtained.

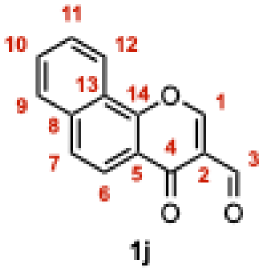

Analytical data according with literature.[23]

^{1}H NMR (500 MHz, $CDCl_3$): δ 10.46 (s, 1H), 8.71 (s, 1H), 8.49 (ddd, *J* = 8.9, 1.6, 0.8 Hz, 1H), 8.21 (d, *J* = 8.7 Hz, 1H), 8.00 – 7.95 (m, 1H), 7.90 – 7.84 (m, 1H), 7.78 – 7.75 (m, 1H), 7.73 (ddd, *J* = 8.2, 7.0, 1.5 Hz, 1H) ppm (Figure S127).

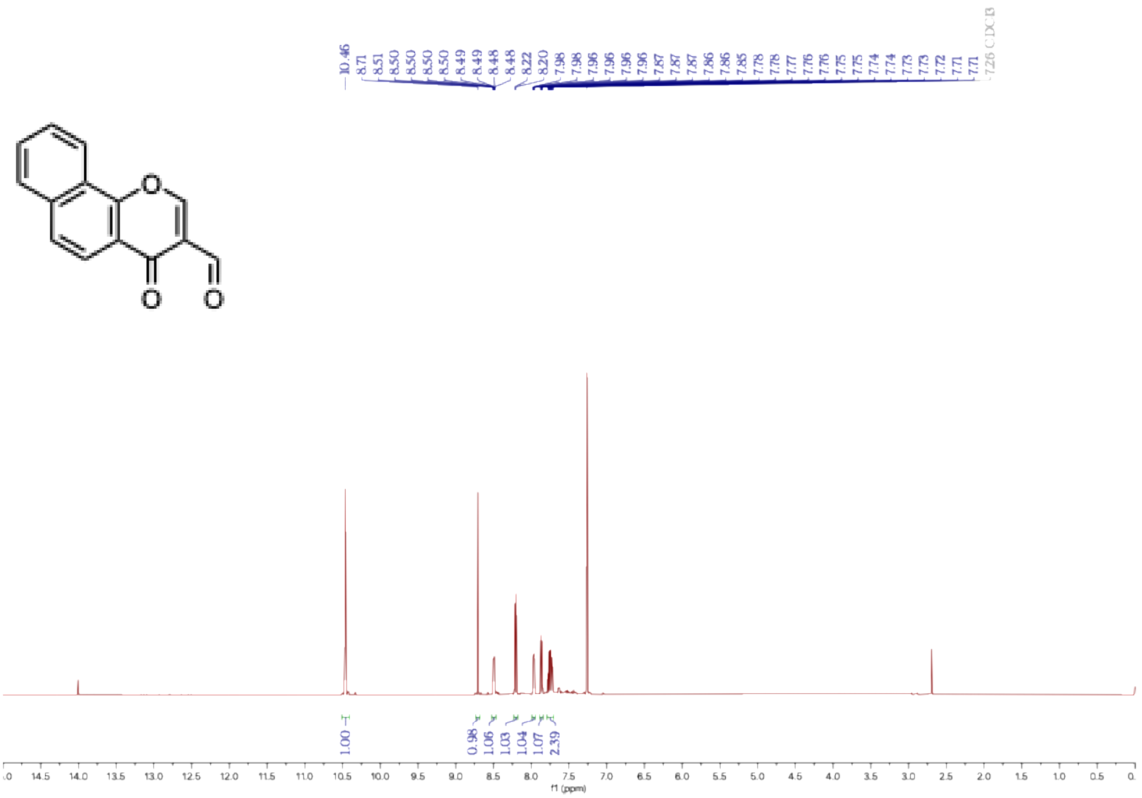

Figure S127 – ^{1}H-NMR (500 MHz, $CDCl_3$) of 3-formylchromone **1j**.

3-formyl-4-oxo-4*H*-chromene-6-carboxylic acid (1k)

Following the general procedure using 3-acetyl-4-hydroxybenzoic acid (2.78 mmol, 500.0 mg) and $POCl_3$ (13.9 mmol, 1.3 mL) in 2 mL of DMF. After filtration, the compound **1k** (1.92 mmol, 417.8 mg, 69% yield) was obtained.

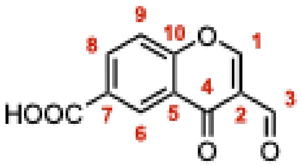

1k

Analytical data according with literature.[24]

^{1}H NMR (300 MHz, DMSO-*d*$_6$): δ 13.32 (br, COO*H*, 1H), 10.10 (s, H-3, 1H), 8.95 (s, H-1, 1H), 8.60 (d, *J* = 2.2 Hz, H-6, 1H), 8.31 (dd, *J* = 8.7, 2.2 Hz, H-8, 1H), 7.83 (d, *J* = 8.8 Hz, H-9, 1H) ppm (Figure S128).

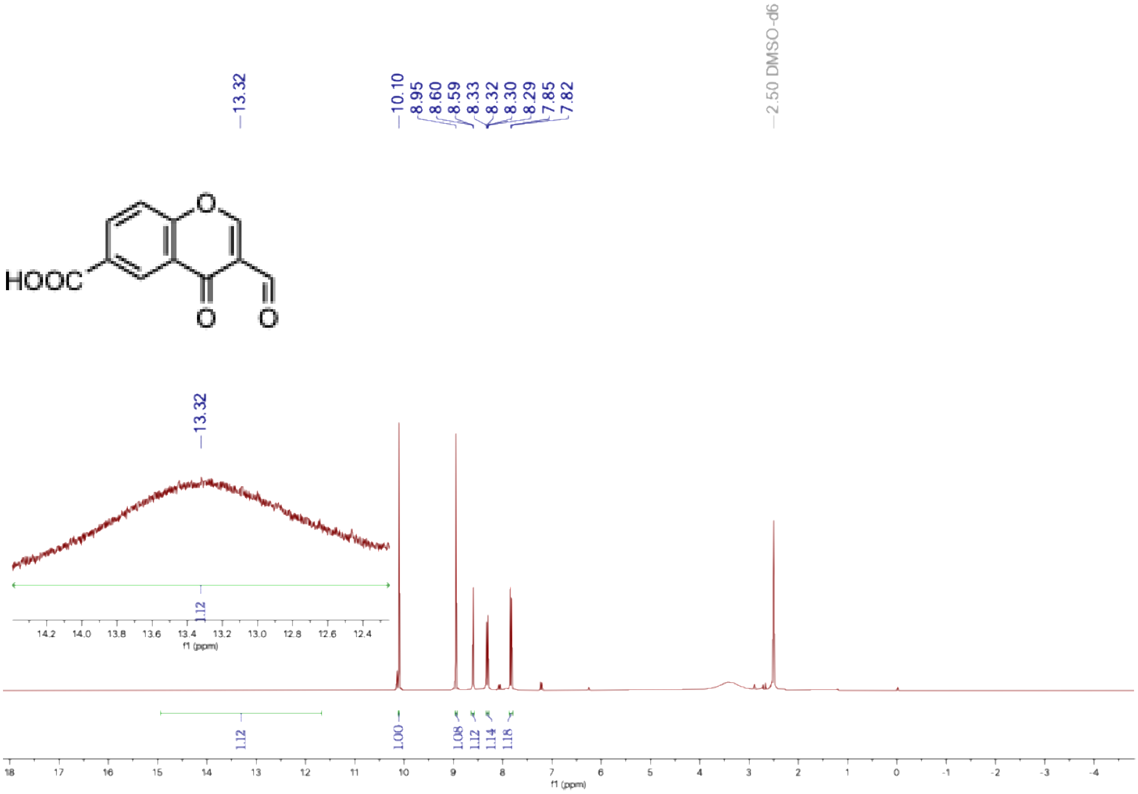

Figure S128 – ^{1}H-NMR (300 MHz, DMSO-d_6) of 3-formylchromone **1k**.

10. Synthesis of Pyridinium Salts

10.1. General Procedures

In a round flask, the respective halogenated precursor (1 equiv.) and pyridine (0.5 M) were mixed. The reaction mixture was let to stir at 50 °C for 2 h. Upon formation of a precipitate, the reaction was removed from heating and cooled in an iced bath. After 15 minutes, the crude mixture was filtered under reduced pressure. The obtained solid was rinsed with pyridine and hexane (Scheme S21).

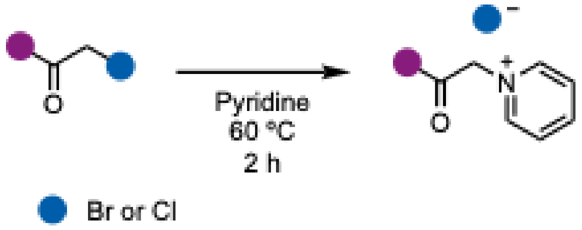

Scheme S21 - General procedure for the synthesis of pyridinium salts **2**.

10.2. Characterization

1-(2-oxo-2-phenylethyl)pyridin-1-ium bromide (2a)

Following the general procedure using 2-bromo-1-phenylethan-1-one (62.4 mmol, 12.42 g) in 250 mL of pyridine. After filtration, the compound **2a** (56.3 mmol, 15.65 g, 90% yield) was obtained.

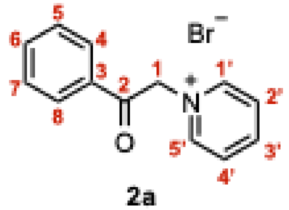

2a

Analytical data according with literature.[25]

^{1}H NMR (300 MHz, DMSO-*d*$_6$): δ 9.15 – 9.05 (m, H-1' and 5', 2H), 8.76 (tt, *J* = 7.8, 1.4 Hz, H-3', 1H), 8.30 (dd, *J* = 7.8, 6.4 Hz, H-2' and 4', 2H), 8.08 (dt, *J* = 7.0, 1.4 Hz, H-4 and 8, 2H), 7.83 – 7.75 (m, H-6, 1H), 7.66 (dd, *J* = 8.3, 7.0 Hz, H-5 and 7, 2H), 6.64 (s, H-1, 2H) ppm (Figure S129).

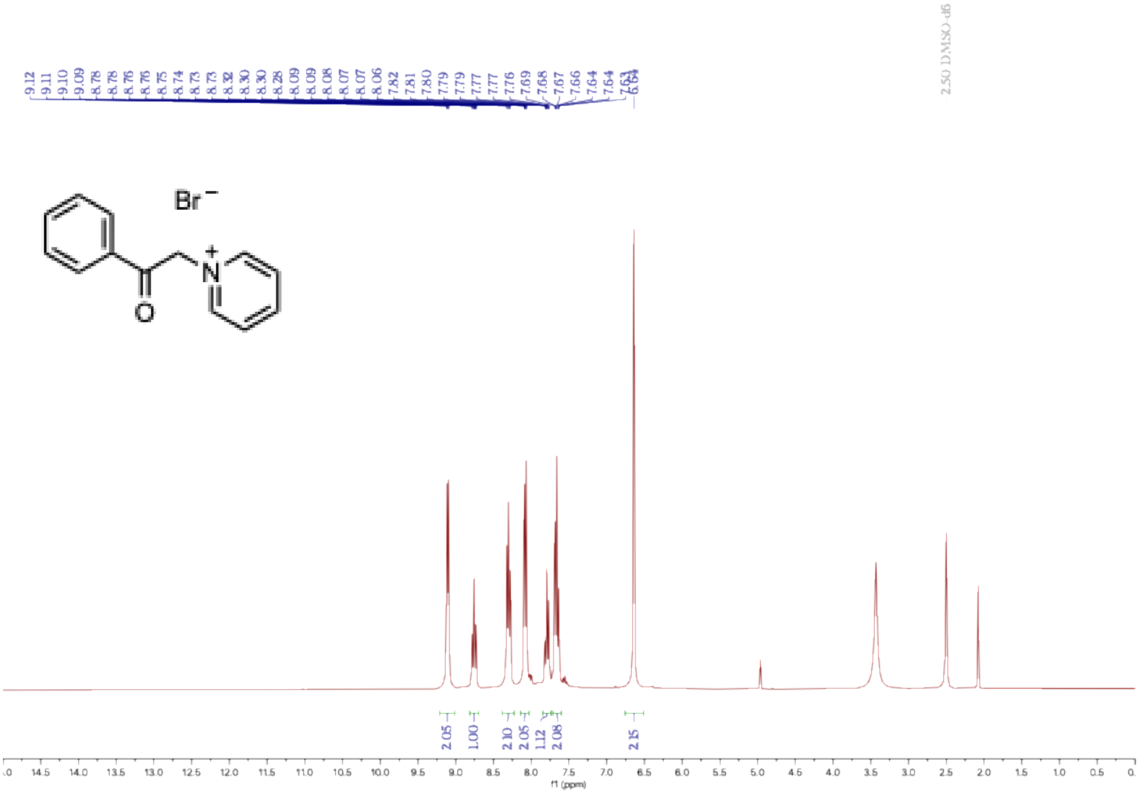

Figure S129 – ^{1}H-NMR (300 MHz, DMSO-d_6) of pyridinium salt **2a**.

1-[2-(4-chlorophenyl)-2-oxoethyl]pyridin-1-ium bromide (2b)

Following the general procedure using 2-bromo-1-(4-chlorophenyl)ethan-1-one (6.24 mmol, 1.46 g) in 25 mL of pyridine. After filtration, the compound **2b** (5.34 mmol, 1.67 g, 86% yield) was obtained.

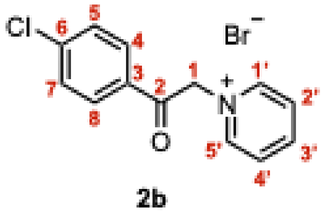

2b Analytical data according with literature.[26]

^{1}H NMR (300 MHz, DMSO-d_6): δ 9.18 (d, J = 6.7 Hz, 2H, H-1' and 5'), 8.76 (t, J = 7.9 Hz, 1H, H-3'), 8.30 (dd, J = 7.9, 6.7 Hz, 2H, H-2' and 4'), 8.08 (d, J = 8.6 Hz, 2H, H-4 and 8), 7.70 (d, J = 8.6 Hz, 2H, H-5 and 7), 6.74 (s, 2H, H-1) ppm (Figure S130).

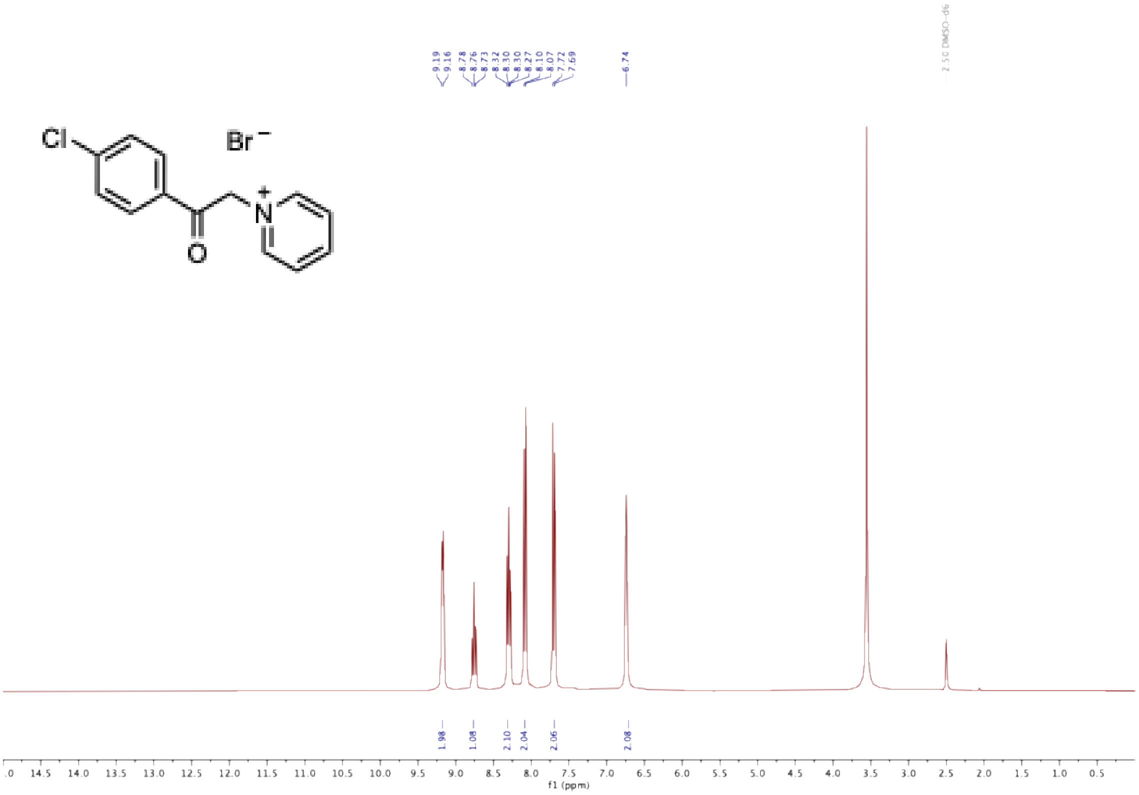

Figure S130 – ^{1}H-NMR (300 MHz, DMSO-d_6) of pyridinium salt **2b**.

1-[2-oxo-2-(*p*-tolyl)ethyl]pyridin-1-ium bromide (2c)

Following the general procedure using 2-bromo-1-(p-tolyl)ethan-1-one (532.7 mg, 2.46 mmol) in 10 mL of pyridine. After filtration, the compound **2c** (1.84 mmol, 563.6 mg, 77% yield) was obtained.

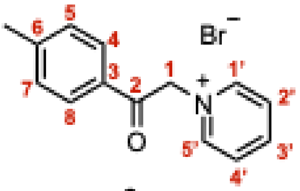

2c

Analytical data according to literature.[26]

^{1}H NMR (300 MHz, DMSO-*d$_6$*): δ 9.09 (d, *J* = 6.0 Hz, H-1' and 5', 2H), 8.80 – 8.69 (m, H-3', 1H), 8.33 – 8.25 (m, H-2' and 4', 2H), 8.03 – 7.94 (m, H-4 and 8, 2H), 7.47 (d, *J* = 7.6 Hz, H-5 and 7, 2H), 6.60 (s, H-1, 2H), 2.43 (s, C*H$_3$*, 3H) ppm (Figure S131).

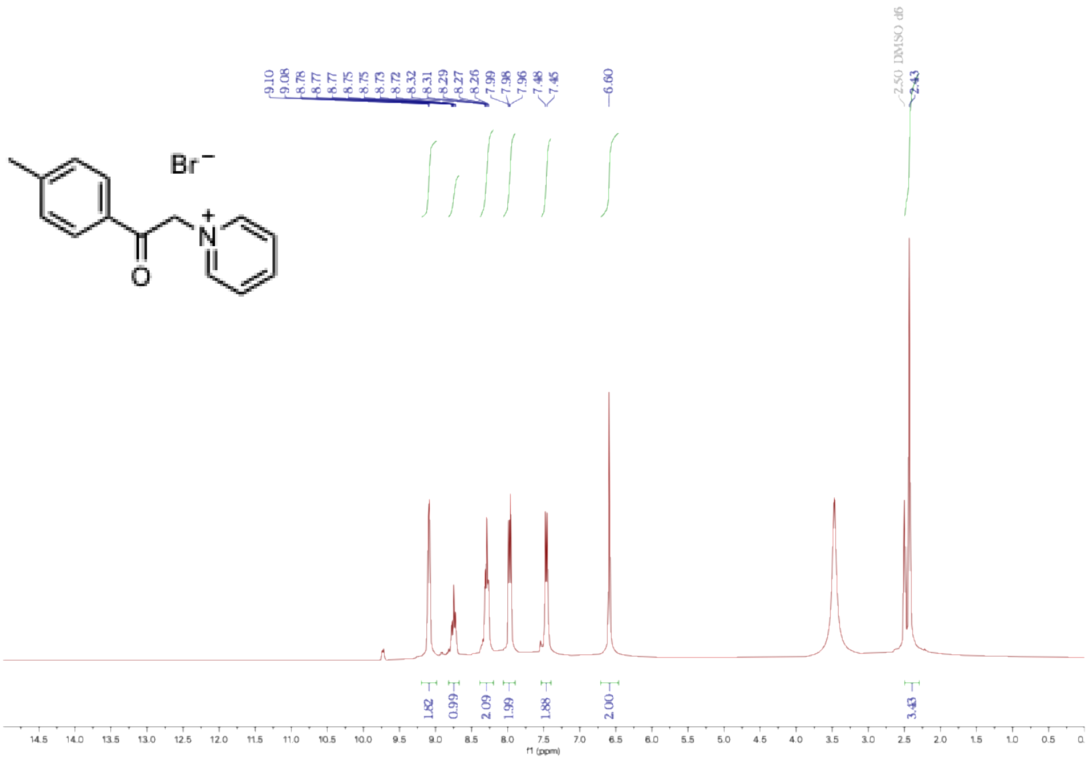

Figure S131 – ^{1}H-NMR (300 MHz, DMSO-*d$_6$*) of pyridinium salt **2c**.

1-[2-(4-methoxyphenyl)-2-oxoethyl]pyridin-1-ium bromide (2d)

Following the general procedure using 2-bromo-1-(4-methoxyphenyl)ethan-1-one (3,12 mmol, 715.0 mg) in 13 mL of pyridine. After filtration, the compound **2d** (2.64 mmol, 812.1 mg, 84% yield) was obtained.

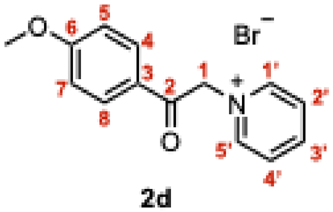

2d Analytical data according to literature.[26]

^{1}H NMR (300 MHz, DMSO-d_6): δ 9.08 (d, *J* = 6.6, 1.5 Hz, H-1’ and 5’, 2H), 8.75 (t, *J* = 7.9, 1.5 Hz, H-3’, 1H), 8.29 (dd, *J* = 7.9, 6.6 Hz, H-2’ and 4’, 2H), 7.97 (d, *J* = 8.2 Hz, H-4 and 8, 3H), 7.47 (d, *J* = 8.2 Hz, H-5 and 7, 2H), 6.58 (s, H-1, 2H), 2.43 (s, OC*H*$_3$, 3H) ppm (Figure S132).

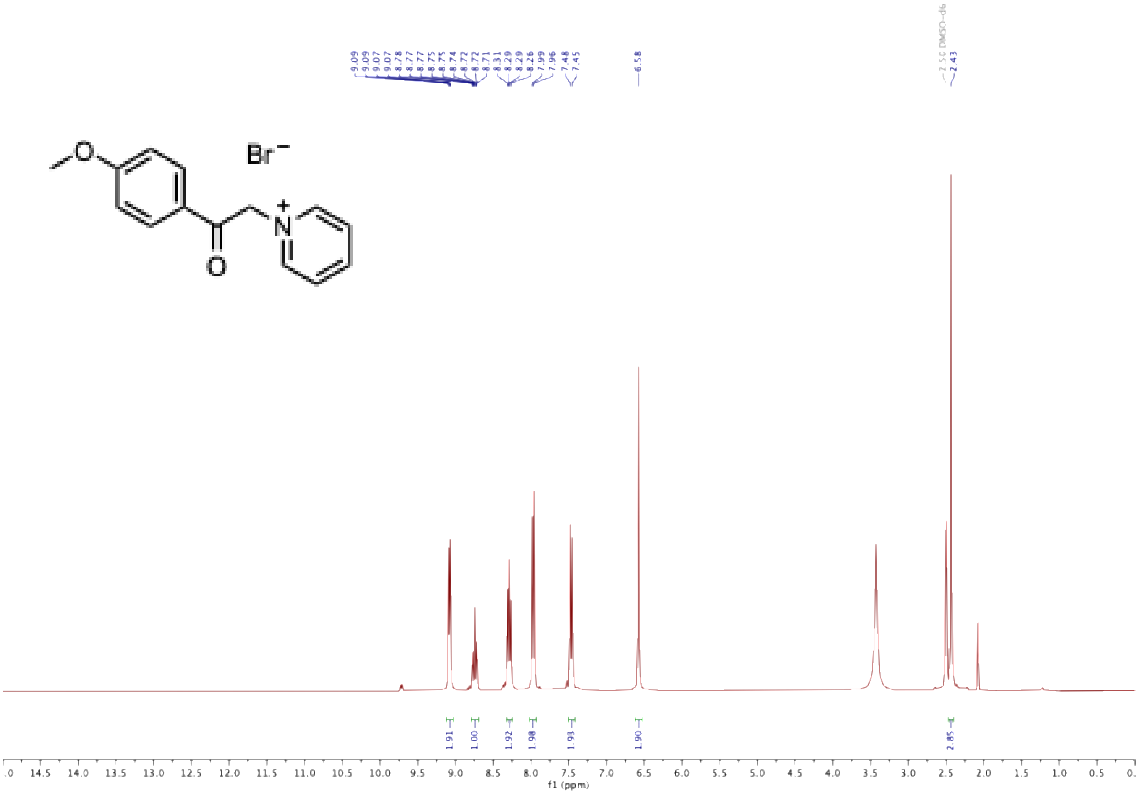

Figure S132 – ^{1}H-NMR (300 MHz, DMSO-d_6) of pyridinium salt **2d**.

1-{2-oxo-2-[4-(trifluoromethyl)phenyl]ethyl}pyridin-1-ium bromide (2e)

Following the general procedure using 2-bromo-1-[4-(trifluoromethyl)phenyl]ethan-1-one (5.24 mmol, 1.00 g) in 10 mL of pyridine. After filtration, the compound **2e** (5.02 mmol, 1.36 g, 96% yield) was obtained.

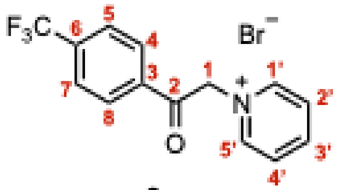

2e Analytical data according with literature.[26]

^{1}H NMR (300 MHz, DMSO-*d$_6$*): δ 9.09 – 9.02 (m, H-1’ and 5’, 2H), 8.76 (tt, *J* = 7.9, 1.4 Hz, H-3’, 1H), 8.36 – 8.24 (m, H-4, 8, 2’ and 4’, 4H), 8.06 (d, *J* = 8.3 Hz, H-5 and 7, 2H), 6.60 (s, H-1, 2H) ppm (Figure S133).

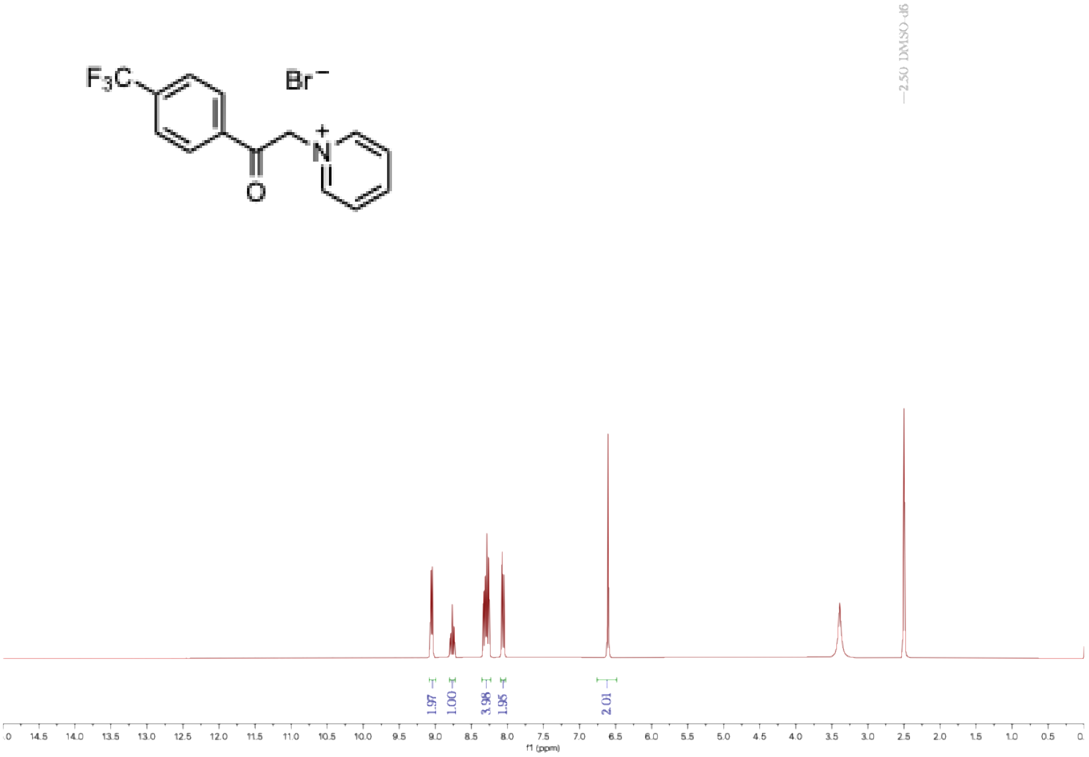

Figure S133 – ^{1}H-NMR (300 MHz, DMSO-*d$_6$*) of pyridinium salt **2e**.

1-[2-(4-carboxyphenyl)-2-oxoethyl]pyridin-1-ium bromide (2f)

Following the general procedure using 4-(2-bromoacetyl)benzoic acid (4.11 mmol, 1.00 g) in 10 mL of pyridine. After filtration, the compound **2f** (4.01 mmol, 1.29 g, 98% yield) was obtained.

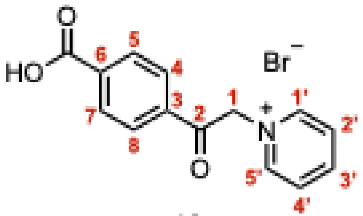

2f White solid. Melting point of 257.9–259.9 °C.

^{1}H NMR (300 MHz, DMSO-*d$_6$*): δ 9.13 – 8.95 (m, H-1' and 5', 2H), 8.75 (tt, *J* = 7.9, 1.4 Hz, H-2', 1H), 8.36 – 8.24 (m, H-2' and 4', 2H), 8.17 (s, H-4, 5, 7 and 8, 4H), 6.58 (s, H-1, 2H) ppm (Figure S134).

^{13}C NMR (75 MHz, DMSO-*d$_6$*): δ 190.7 (C-2), 166.6 (*C*OOH), 146.6 (C-3'), 146.4 (C-1' and 5', 2C), 136.7 (C-3), 135.8 (C-6), 130.0 (C-4 and 8 or 5 and 7, 2C), 128.6 (C-4 and 8 or 5 and 7, 2C), 128.0 (C-2' and 4', 2C), 66.5 (C-1) ppm (Figure S135).

HRMS (ESI) *m/z*: [M]+ Calcd for $C_{14}H_{12}N_2O_3^+$ 242.0812 ; Found 242.0823.

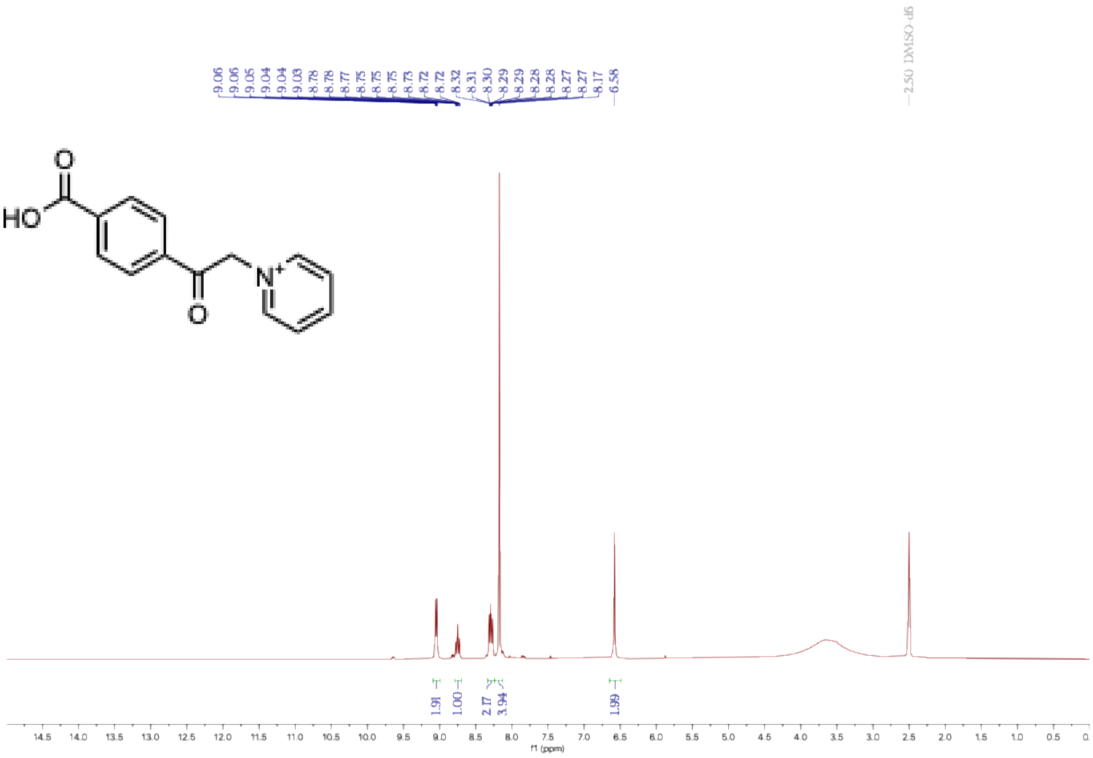

Figure S134 – ^{1}H-NMR (300 MHz, DMSO-*d$_6$*) of pyridinium salt **2f**.

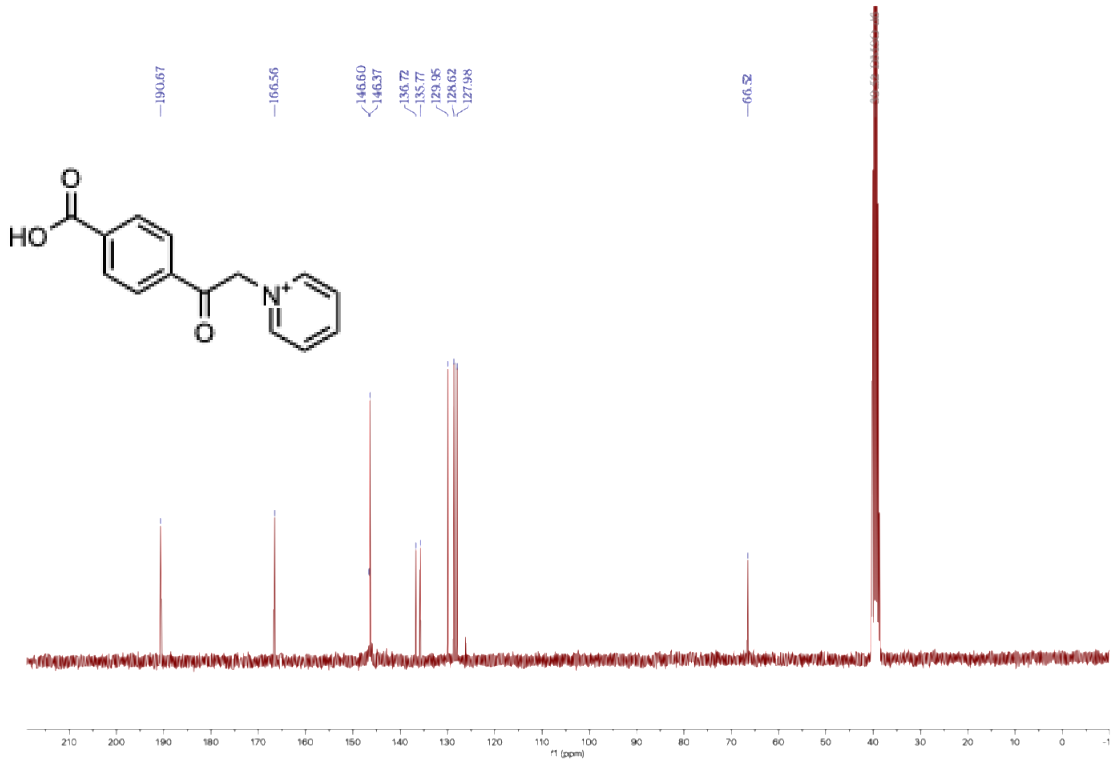

Figure S135 – ^{13}C-NMR (75 MHz, DMSO-d_6) of pyridinium salt **2f**.

1-[2-(4-aminophenyl)-2-oxoethyl]pyridin-1-ium bromide (2g)

Following the general procedure using 1-(4-aminophenyl)-2-bromoethan-1-one (1-40 mmol, 300.0 mg) in 3 mL of pyridine. After filtration, the compound **2g** (1.34 mmol, 392,6 mg, 96% yield) was obtained.

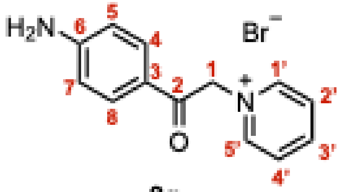

2g

Light brown solid. Melting point of >410.0 °C.

^{1}H NMR (300 MHz, DMSO-d_6): δ 9.07 – 8.99 (m, H-1' and 5', 2H), 8.69 (tt, *J* = 7.9, 1.4 Hz, H-3', 1H), 8.29 – 8.17 (m, H-2' and 4', 2H), 7.77 – 7.69 (m, H-4 and 8, 2H), 6.72 – 6.64 (m, H-5 and 7, 2H), 6.36 (s, H-1, 2H) ppm (Figure S136).

^{13}C NMR (75 MHz, DMSO-d_6): δ 186.9 (C-2), 155.2 (C-6), 146.3 (C-1' and 5', 2C), 146.0 (C-3'), 130.9 (C-4 and 8, 2C), 127.7 (C-2' and 4', 2C), 120.5 (C-3), 112.8 (C-5 and 7, 2C), 65.4 (C-1) ppm (Figure S137).

HRMS (ESI) *m/z*: [M]+ Calcd for $C_{13}H_{11}N_2O^+$ 213.1022 ; Found 213.1032.

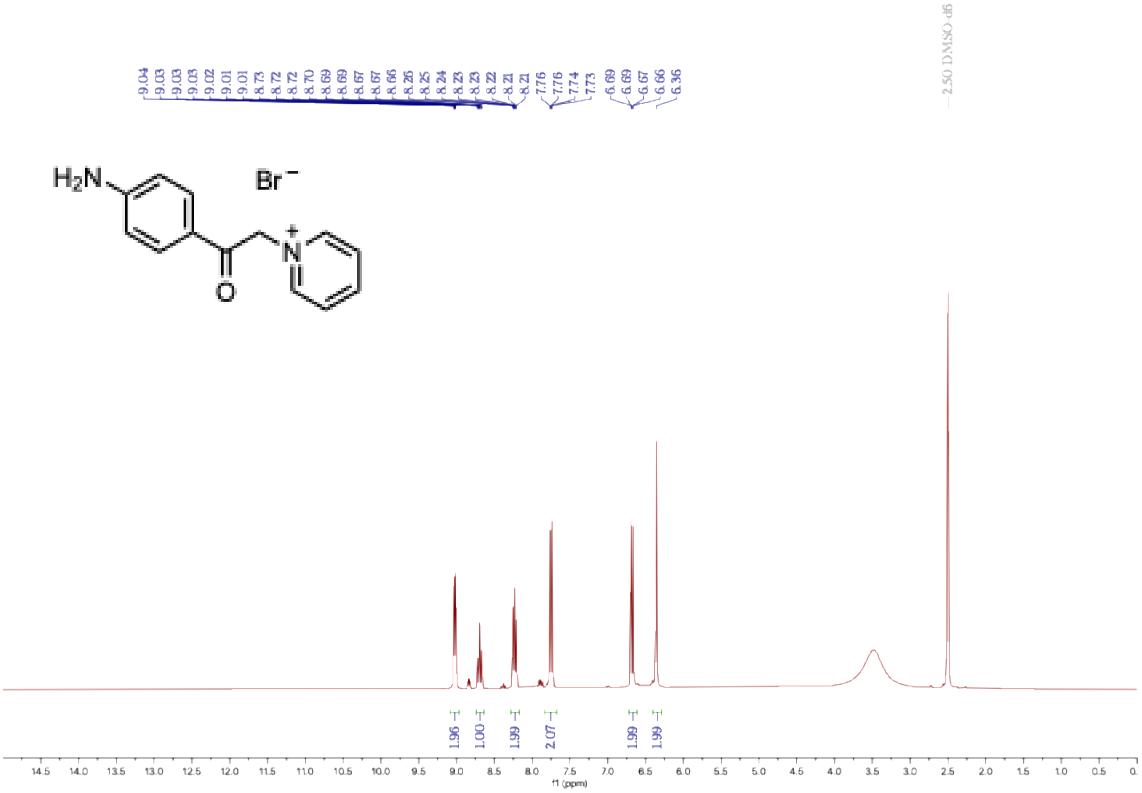

Figure S136 – ^{1}H-NMR (300 MHz, DMSO-d_6) of pyridinium salt **2g**.

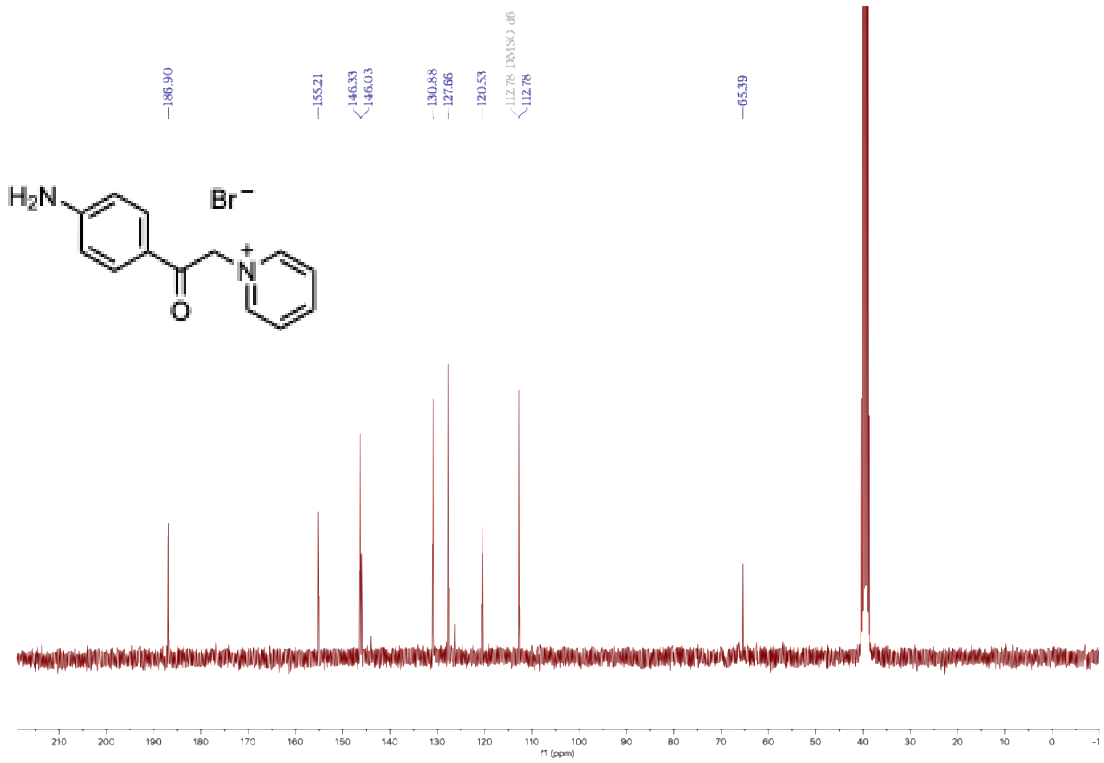

Figure S137 – ^{13}C-NMR (75 MHz, DMSO-d_6) of pyridinium salt **2g**.

1-[2-(4-cyanophenyl)-2-oxoethyl]pyridin-1-ium bromide (2h)

Following the general procedure using 4-(2-bromoacetyl)benzonitrile (3.12 mmol, 699.0 mg) in 13 mL of pyridine. After filtration, the compound **2h** (2.95 mmol, 893.9 mg, 95% yield) was obtained.

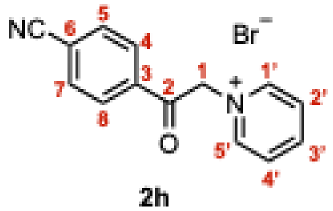

2h

Analytical data according to literature.[26]

^{1}H NMR (300 MHz, DMSO-*d$_6$*): δ 9.16 – 9.06 (m, 2H, H-1' and 5'), 8.77 (tt, *J* = 7.8, 1.4 Hz, 1H, H-3'), 8.31 (dd, *J* = 7.8, 6.4 Hz, 2H, H-2' and 4'), 8.23 (d, *J* = 8.7 Hz, 2H, H-4 and 8), 8.15 (d, *J* = 8.7 Hz, 2H, H-5 and 7), 6.70 (s, 2H, H-1) ppm (Figure S138).

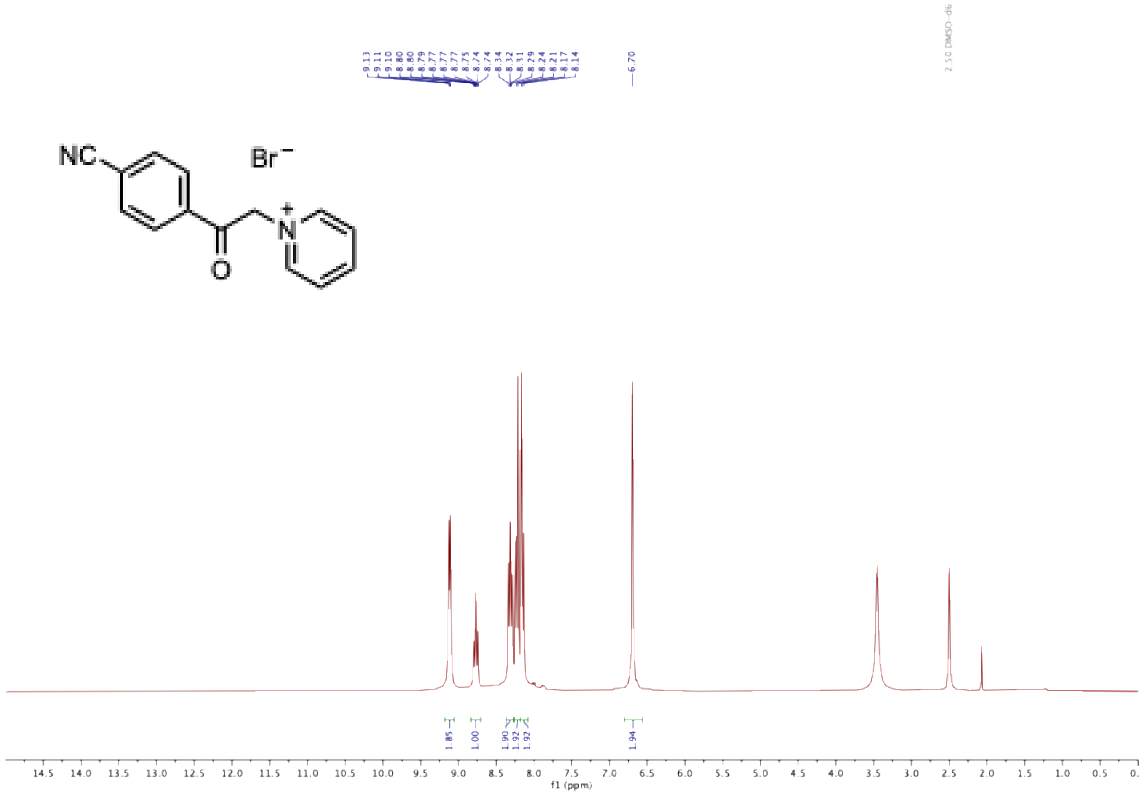

Figure S138 – ^{1}H-NMR (300 MHz, DMSO-*d$_6$*) of pyridinium salt **2h**.

1-[2-(4-nitrophenyl)-2-oxoethyl]pyridin-1-ium bromide (2i)

Following the general procedure using 2-bromo-1-(4-nitrophenyl)ethan-1-one (6.24 mmol, 1.53 g) in 25 mL of pyridine. After filtration, the compound **2i** (5.97 mmol, 1.93 mg, 96% yield) was obtained.

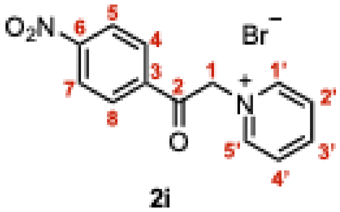

2i White solid. Melting point of 267.8–269.5 °C.

^{1}H NMR (300 MHz, DMSO-*d6*): δ 9.18 – 9.09 (m, 2H, H-1' and 5'), 8.78 (td, J = 7.8, 1.4 Hz, 1H, H-3'), 8.46 (d, J = 8.8 Hz, 2H, H-5 or 7), 8.37 – 8.27 (m, 4H, H-4, 8, 2' and 4'), 6.74 (s, 2H, H-1) ppm (Figure S139).

^{13}C NMR (75 MHz, DMSO-*d6*): δ 190.2 (C-2), 150.6 (C-6), 146.6 (C-3'), 146.3 (C1' and 5', 2C), 138.3 (C-3), 129.8 (C-4 and 8, 2C), 127.9 (C-2' and 3', 2C), 124.2 (C-5 and 7, 2C), 66.5 (C-1) ppm (Figure S140).

HRMS (ESI) *m/z*: [M]+ Calcd for $C_{13}H_{11}N_2O_3^+$ 243.0764 ; Found 243.0775.

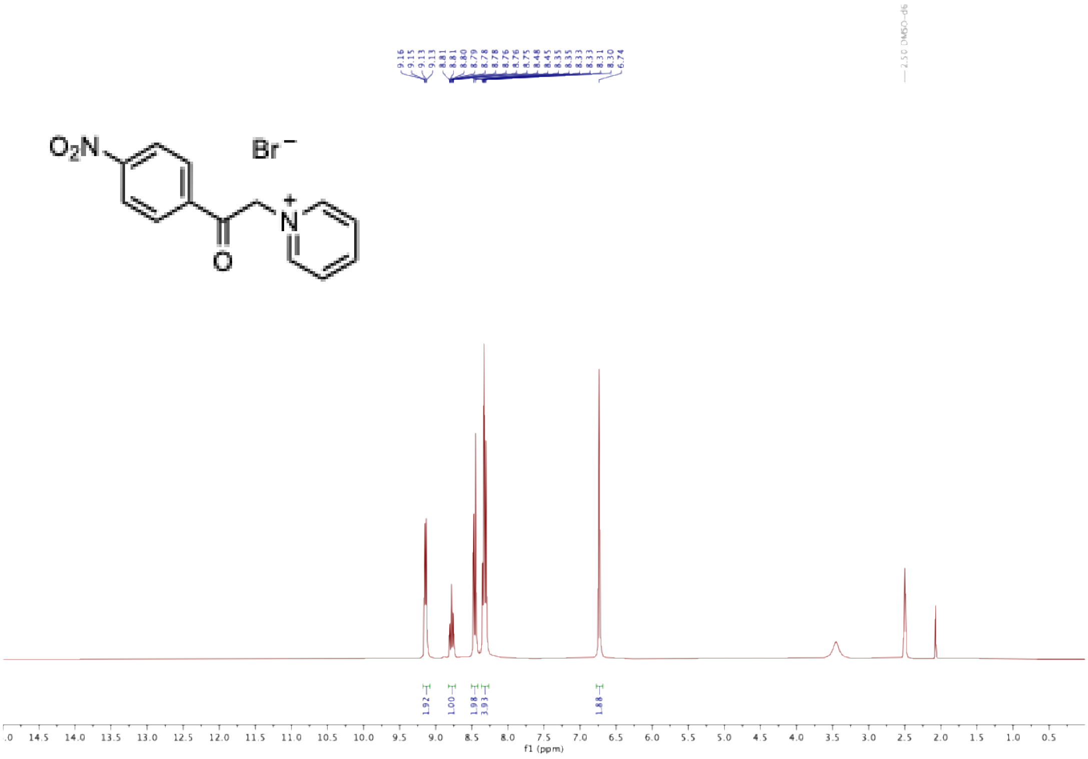

Figure S139 – ^{1}H-NMR (300 MHz, DMSO-*d6*) of pyridinium salt **2i**.

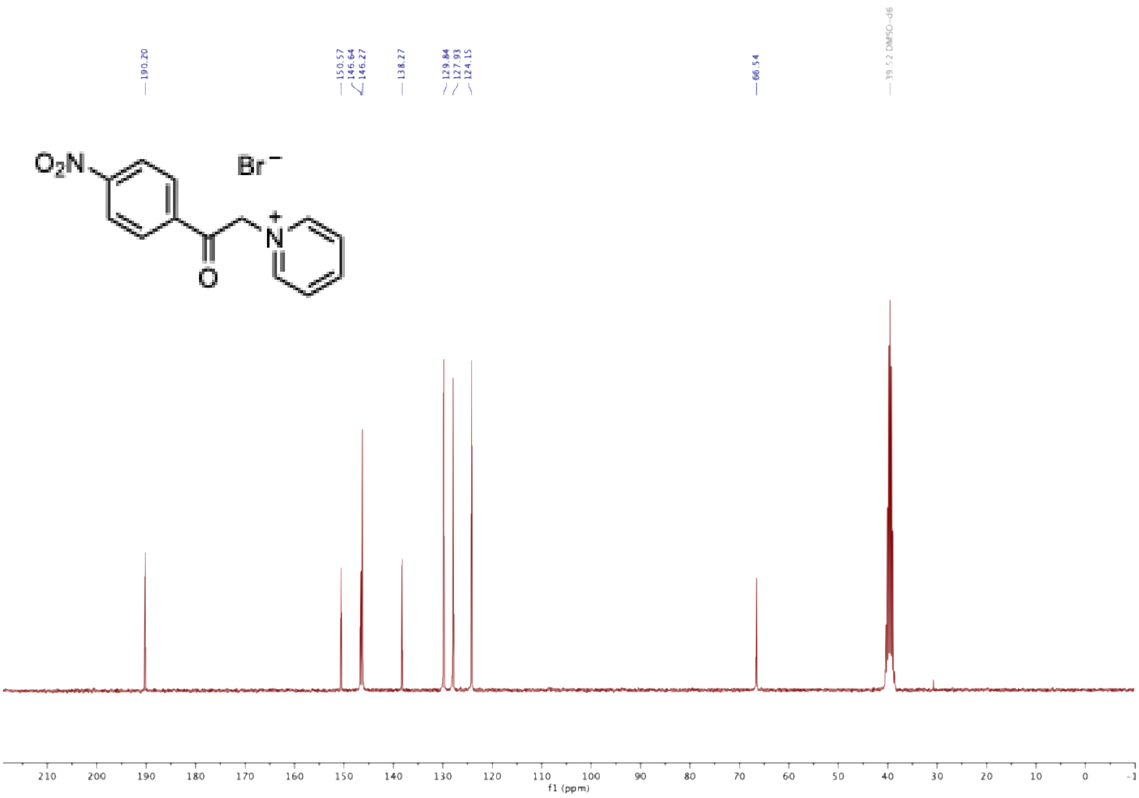

Figure S140 – ^{13}C-NMR (75 MHz, DMSO-d_6) of pyridinium salt **2i**.

1-[2-(2-bromophenyl)-2-oxoethyl]pyridin-1-ium bromide (2j)

Following the general procedure using 2-bromo-1-(2-bromophenyl)ethan-1-one (5,40 mmol, 1.50 g) in 15 mL of pyridine. After filtration, the compound **2j** (4.88 mmol, 1.74 g, 90% yield) was obtained.

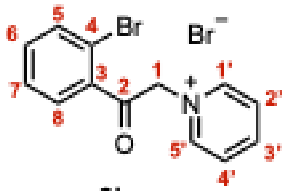

2j Analytical data according to literature.[27]

^{1}H NMR (300 MHz, DMSO-*d$_6$*): δ 9.15 – 9.06 (m, H-1’ and 5’, 2H), 8.77 (tt, *J* = 7.8, 1.4 Hz, H-3’, 1H), 8.37 – 8.28 (m, H-2’ and 4’, 2H), 8.17 (dd, *J* = 7.6, 1.9 Hz, H-8, 1H), 7.87 (dd, *J* = 7.7, 1.5 Hz, H-5, 1H), 7.68 (ddd, *J* = 7.6, 7.5, 1.5 Hz, H-7, 1H), 7.61 (ddd, *J* = 7.7, 7.5, 1.9 Hz, H-6, 1H), 6.53 (s, H-1, 2H) ppm (Figure S141).

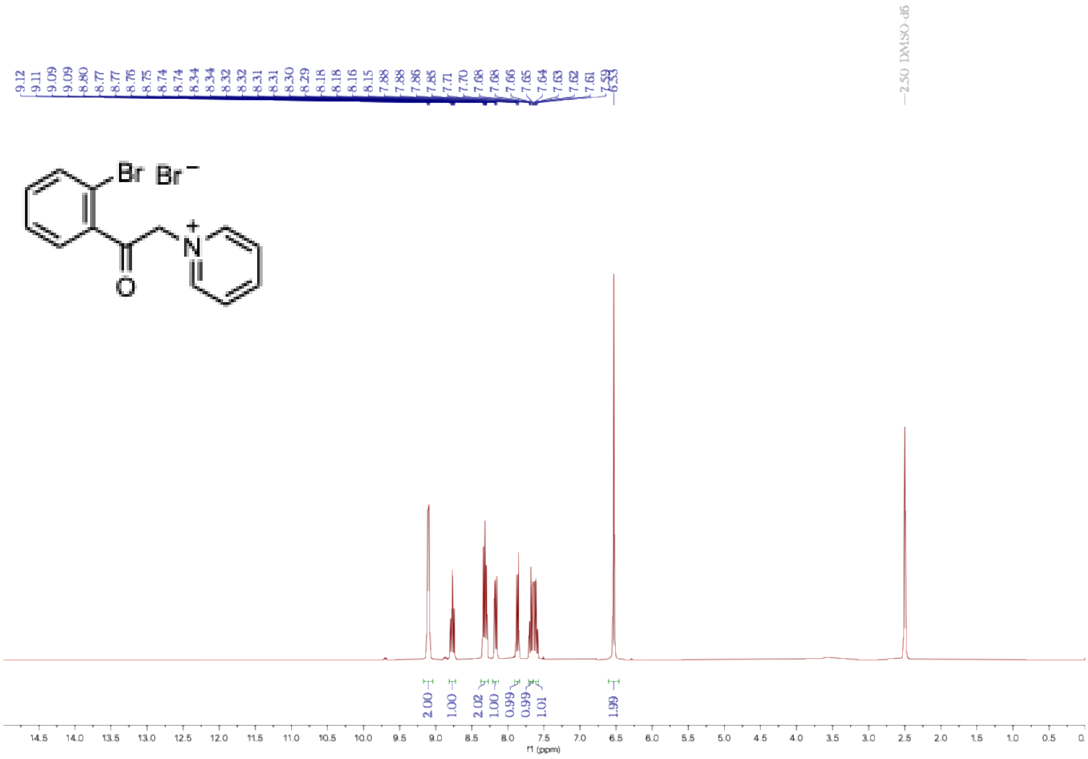

Figure S141 – ^{1}H-NMR (300 MHz, DMSO-*d$_6$*) of pyridinium salt **2j**.

1-[2-(naphthalen-2-yl)-2-oxoethyl]pyridin-1-ium bromide (2k)

Following the general procedure using 2-bromo-1-(naphthalen-2-yl)ethan-1-one (3.12 mmol, 777.2 mg) in 13 mL of pyridine. After filtration, the compound **2k** (2.23 mmol, 732.4 mg, 84% yield) was obtained.

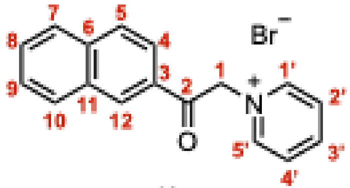

2k

Analytical data according with literature.[26]

^{1}H NMR (300 MHz, DMSO-*d$_6$*): δ 9.16 (m, H-1’ and 5’, 2H), 8.97 – 8.83 (m, H-12, 1H), 8.84 – 8.73 (m, H-3’, 1H), 8.33 (dd, *J* = 7.9, 6.5 Hz, H-8 or 9, 2H), 8.24 (dd, *J* = 7.9, 1.5 Hz, H-7 or 10, 1H), 8.16 (d, *J* = 8.7 Hz, H-5, 1H), 8.07 (ddd, *J* = 10.6, 8.4, 1.7 Hz, H-2’ and 4’, 2H), 7.81 – 7.67 (m, H-7 and 8 or 9 and 10, 2H), 6.77 (s, 2H) ppm (Figure S142).

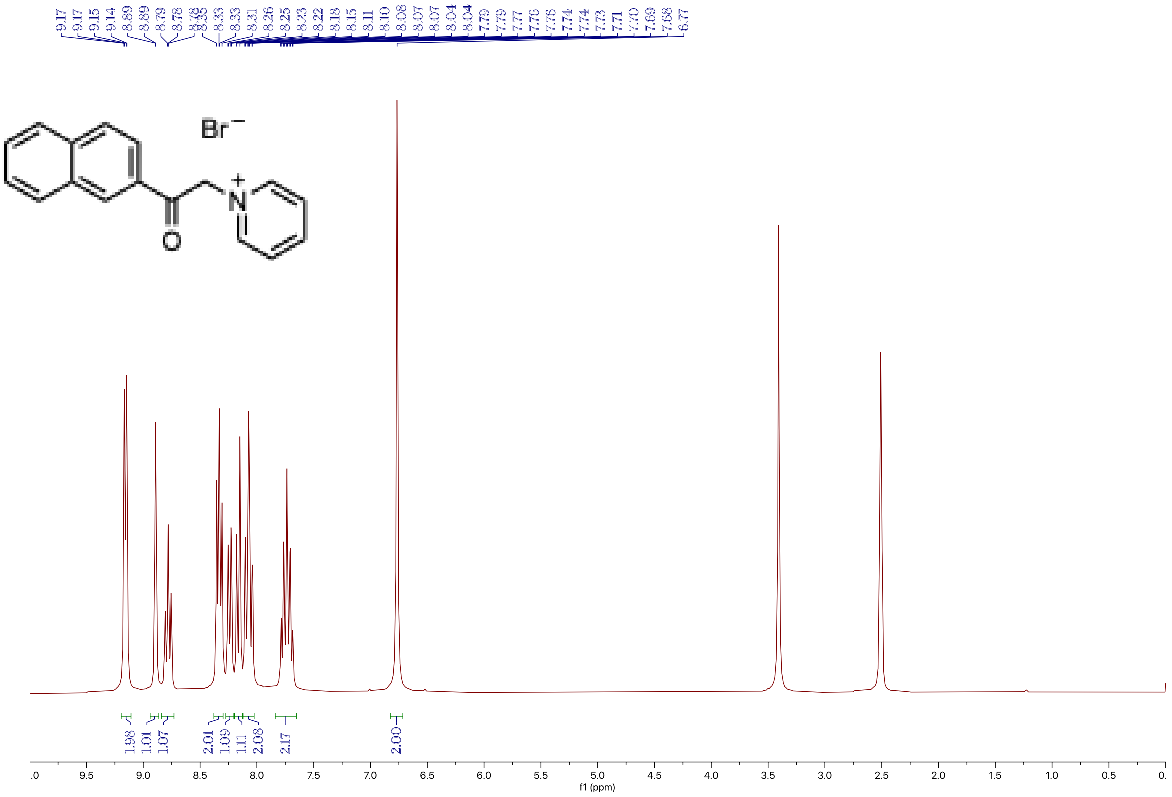

Figure S142 – ^{1}H-NMR (300 MHz, DMSO-*d$_6$*) of pyridinium salt **2k**.

1-[2-oxo-2-(pyren-1-yl)ethyl]pyridin-1-ium bromide (2l)

Following the general procedure using 1-(2-oxo-2-(pyren-1-yl)ethyl)pyridin-1-ium bromide (1.24 mmol, 400.0 mg) in 5 mL of pyridine. After filtration, the compound **2l** (1.15 mmol, 463.5 mg, 93% yield) was obtained.

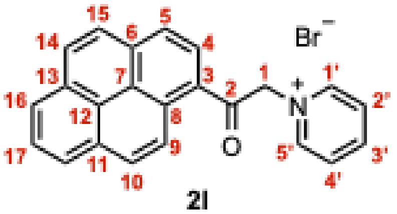

2l

Yellow solid. Melting point of 279.0–281.0 °C.

^{1}H NMR (300 MHz, DMSO-*d$_6$*): δ 9.23 – 9.17 (m, H-Ar, 2H), 9.07 (d, *J* = 9.4 Hz, H-Ar, 1H), 8.86 (d, *J* = 8.2 Hz, H-Ar, 1H), 8.80 (tt, *J* = 7.8, 1.4 Hz, H-Ar, 1H), 8.55 (d, *J* = 8.2 Hz, H-Ar, 1H), 8.51 – 8.41 (m, H-Ar, 4H), 8.40 – 8.31 (m, H-Ar, 3H), 8.20 (t, *J* = 7.7 Hz, H-Ar, 1H), 6.78 (s, H-1, 2H) ppm (Figure S143).

^{13}C NMR (75 MHz, DMSO-*d$_6$*): δ 193.2 (C-2), 146.6 (C-Ar), 146.5 (C-Ar), 134.7 (C-Ar), 130.7 (C-Ar), 130.6 (C-Ar), 130.5 (C-Ar), 129.9 (C-Ar), 129.8 (C-Ar), 128.0 (C-Ar), 127.4 (C-Ar), 127.3 (C-Ar), 127.2 (C-Ar), 126.9 (C-Ar), 126.7 (C-Ar), 124.6 (C-Ar), 124.4 (C-Ar), 124.1 (C-Ar), 123.2 (C-Ar), 67.9 (C-1) ppm (Figure S144).

HRMS (ESI) *m/z*: [M]$^+$ Calcd for $C_{23}H_{16}NO^+$ 322.1226 ; Found 322.1240.

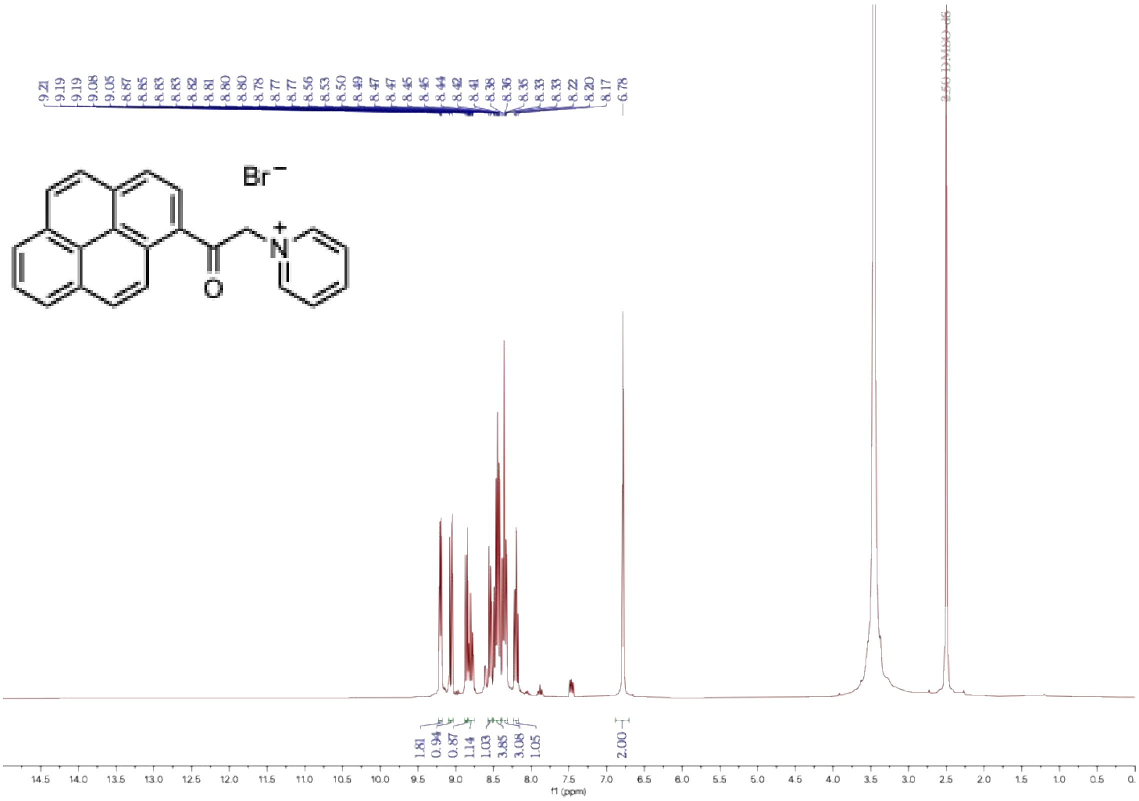

Figure S143 – ^{1}H-NMR (300 MHz, DMSO-*d₆*) of pyridinium salt **2l**.

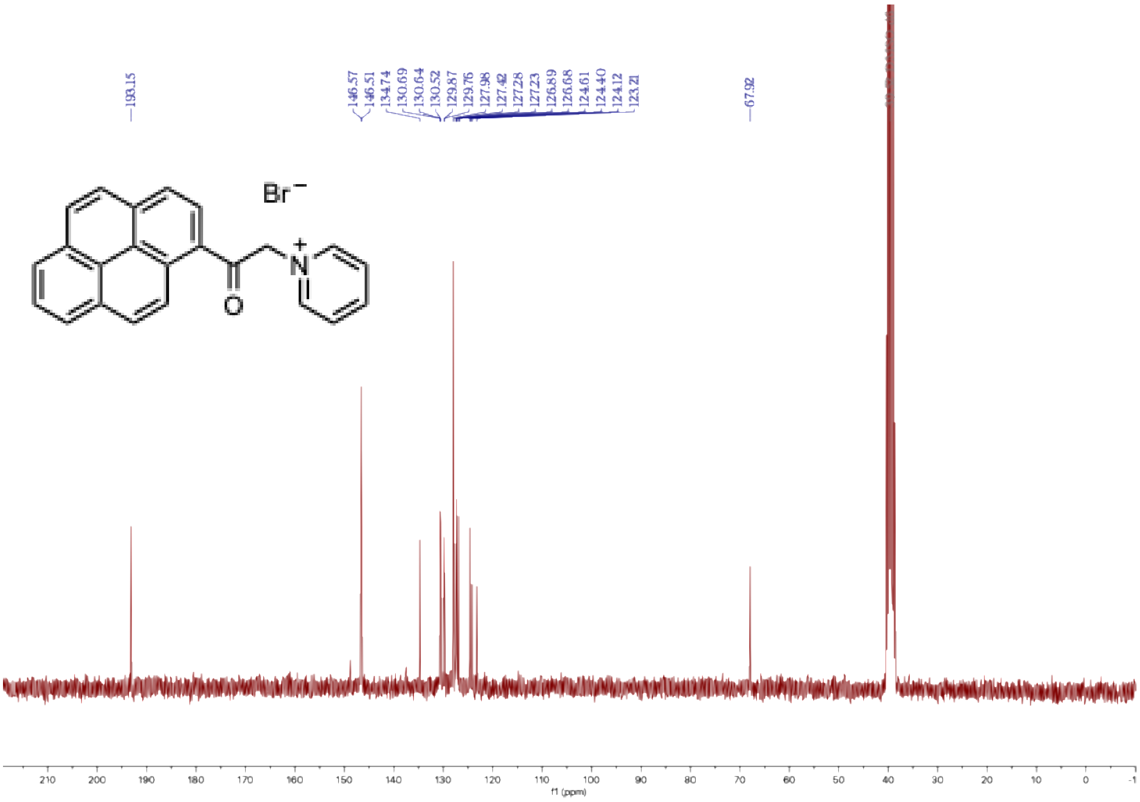

Figure S144 – ^{13}C-NMR (75 MHz, DMSO-*d₆*) of pyridinium salt **2l**.

1-[2-(2,3-dihydrobenzo[b][1,4]dioxin-6-yl)-2-oxoethyl]pyridin-1-ium chloride (2m)

Following the general procedure using 2-chloro-1-(2,3-dihydrobenzo[b][1,4]dioxin-6-yl)ethan-1-one (2.50 mmol, 531.6 mg) in 10 mL of pyridine. After filtration, the compound **2m** (2.28 mmol, 664.1 mg, 91% yield) was obtained.

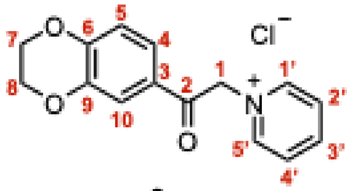

2m White solid. Melting point of 274.1–275.9 °C.

^{1}H NMR (300 MHz, DMSO-*d*$_6$): δ 9.07 – 9.01 (m, H-1' and 5', 2H), 8.72 (tt, *J* = 7.8, 1.4 Hz, H-3', 1H), 8.26 (dd, *J* = 7.9, 6.5 Hz, H-2' and 4', 2H), 7.60 (dd, *J* = 8.4, 2.2 Hz, H-4, 1H), 7.56 (d, *J* = 2.1 Hz, H-10, 1H), 7.12 (d, *J* = 8.4 Hz, H-5, 1H), 6.48 (s, H-1, 2H), 4.42 – 4.30 (m, H-7 and 8, 4H) ppm (Figure S145).

^{13}C NMR (75 MHz, DMSO-*d*$_6$): δ 189.1 (C-1), 149.2 (C-6 or 9), 146.3 (C-1', 3'and 5', 3C), 143.5 (C-6 or 9), 127.8 (C-2' and 4', 2C), 126.9 (C-3), 122.5 (C-4), 117.7 (C-5), 117.3 (C-10), 65.9 (C-1), 64.8 (C-7 or 8), 64.0 (C-7 or 8) ppm (Figure S146).

HRMS (ESI) *m/z*: [M]$^+$ Calcd for $C_{15}H_{14}NO_3^+$ 256.0968 ; Found 256.0979.

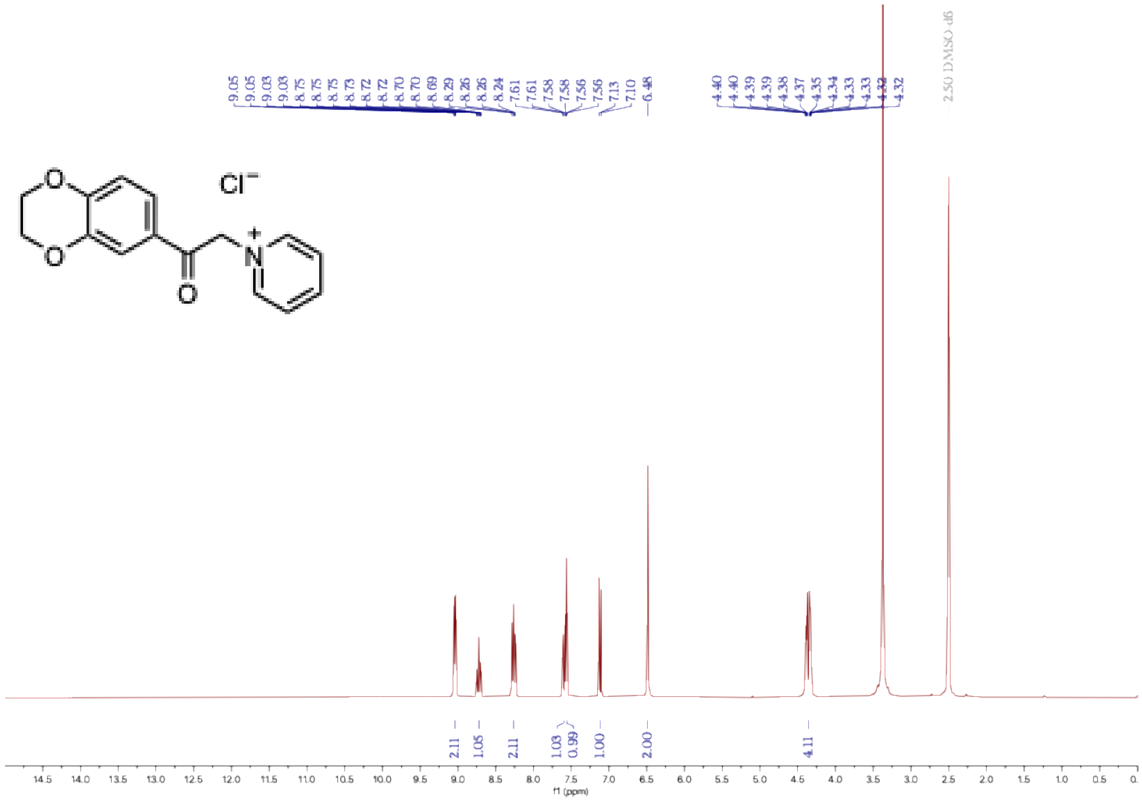

Figure S145 – ^{1}H-NMR (300 MHz, DMSO-d_6) of pyridinium salt **2m**.

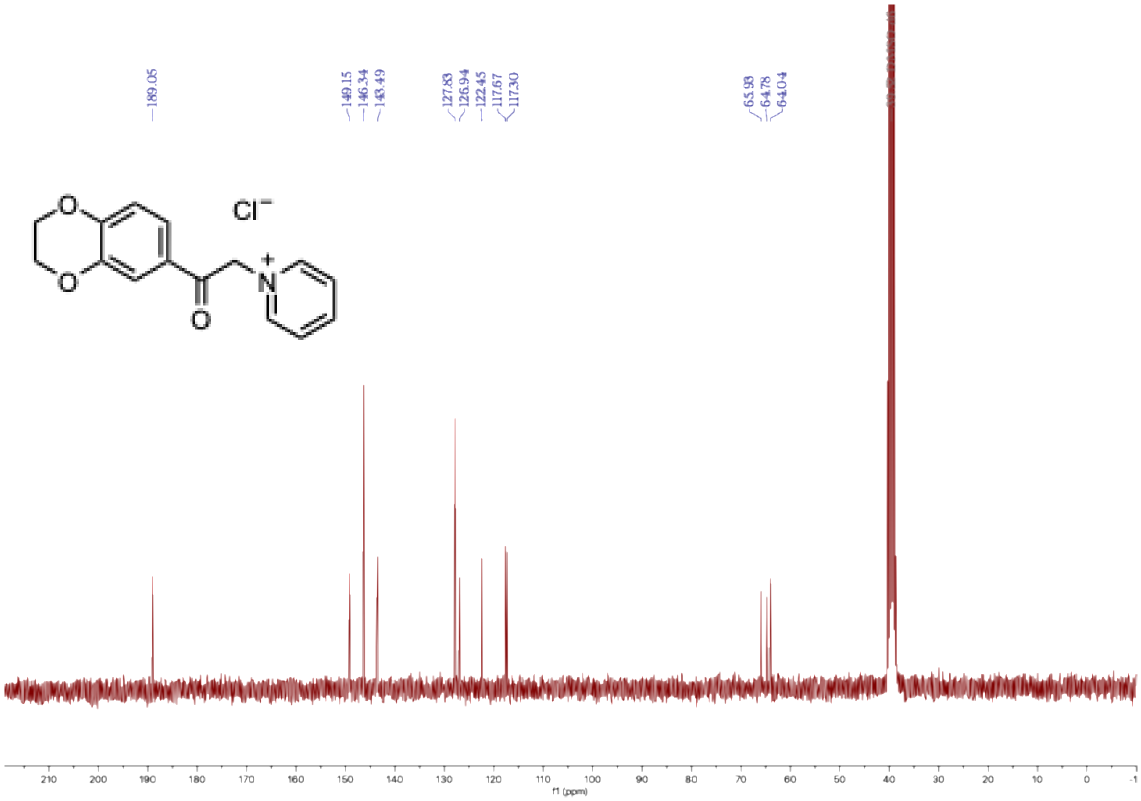

Figure S146 – ^{13}C-NMR (75 MHz, DMSO-d_6) of pyridinium salt **2m**.

1-[2-oxo-2-(pyridin-4-yl)ethyl]pyridin-1-ium bromide (2n)

Following the general procedure using 2-bromo-1-(pyridin-4-yl)ethan-1-one (2.50 mmol, 702.4 mg) in 20 mL of pyridine. After filtration, the compound **2o** (1.97 mmol, 551.2 mg, 79% yield) was obtained.

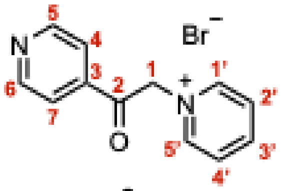

2n Dark orange solid. Melting point of 176.6–179.1 °C.

^{1}H NMR (300 MHz, DMSO-*d*$_6$): δ 9.04 (d, *J* = 5.9 Hz, H-1' and 5', 2H), 8.95 (s, H-5 and 6, 2H), 8.76 (t, *J* = 7.8 Hz, H-3', 1H), 8.35 – 8.26 (m, H-2' and 4', 2H), 7.95 (s, H-4 and 7, 2H), 6.57 (s, H-1, 2H) ppm (Figure S147).

^{13}C NMR (75 MHz, DMSO-*d*$_6$): δ 191.1 (C-2), 151.2 (C-5 and 6, 2C), 146.7 (C-3'), 146.3 (C-1' and 5', 2C), 139.7 (C-3), 128.0 (C-2' and 4', 2C), 121.2 (C-4 and 7, 2C), 66.5 (C-1) ppm (Figure S148).

HRMS (ESI) *m/z*: [M]$^+$ Calcd for $C_{12}H_{11}N_2O^+$ 199.0866 ; Found 199.0875.

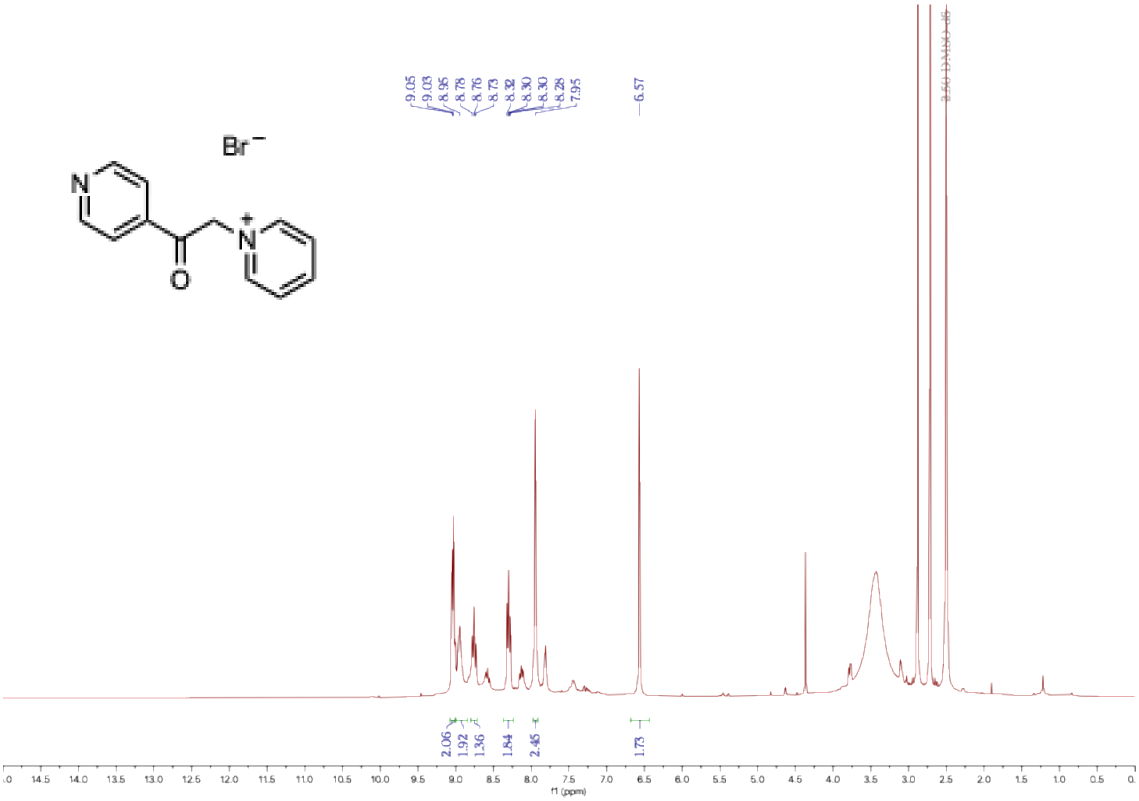

Figure S147 – ^{1}H-NMR (300 MHz, DMSO-*d*$_6$) of pyridinium salt **2n**.

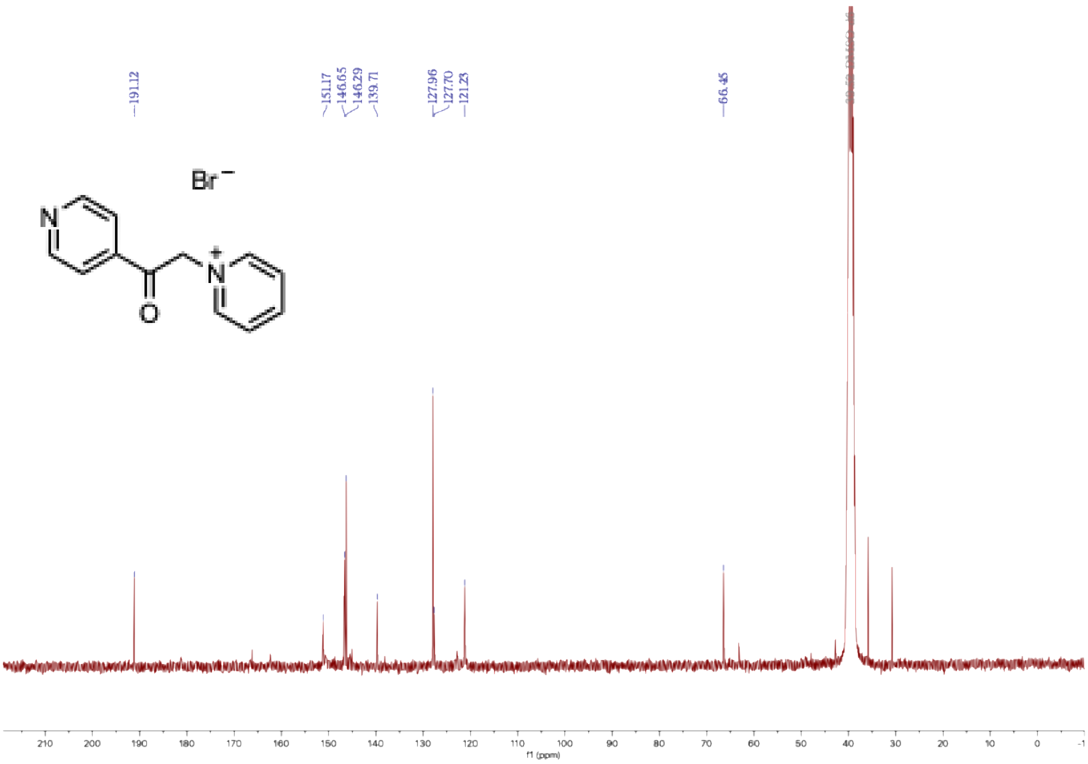

Figure S148 – ^{13}C-NMR (75 MHz, DMSO-*d6*) of pyridinium salt **2n**.

1-[2-oxo-2-(thiophen-2-yl)ethyl]pyridin-1-ium bromide (2o)

Following the general procedure using 2-bromo-1-(thiophen-2-yl)ethan-1-one (1.80 mmol, 369.1 mg) in 7 mL of pyridine. After filtration, the compound **2o** (1.56 mmol, 442.8 mg, 87% yield) was obtained.

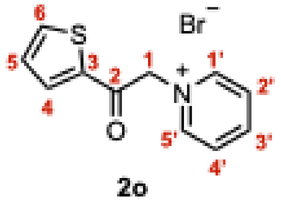

2o

Analytical data according with literature.[29]

^{1}H NMR (300 MHz, DMSO-*d$_6$*): δ 9.12 – 9.06 (m, H-1' and 5', 2H), 8.74 (tt, *J* = 7.8, 1.4 Hz, H-3', 1H), 8.31 – 8.21 (m, H-4, 6, 2' and 4', 4H), 7.41 (dd, *J* = 4.9, 3.9 Hz, H-5, 1H), 6.51 (s, H-1, 2H) ppm (Figure S149).

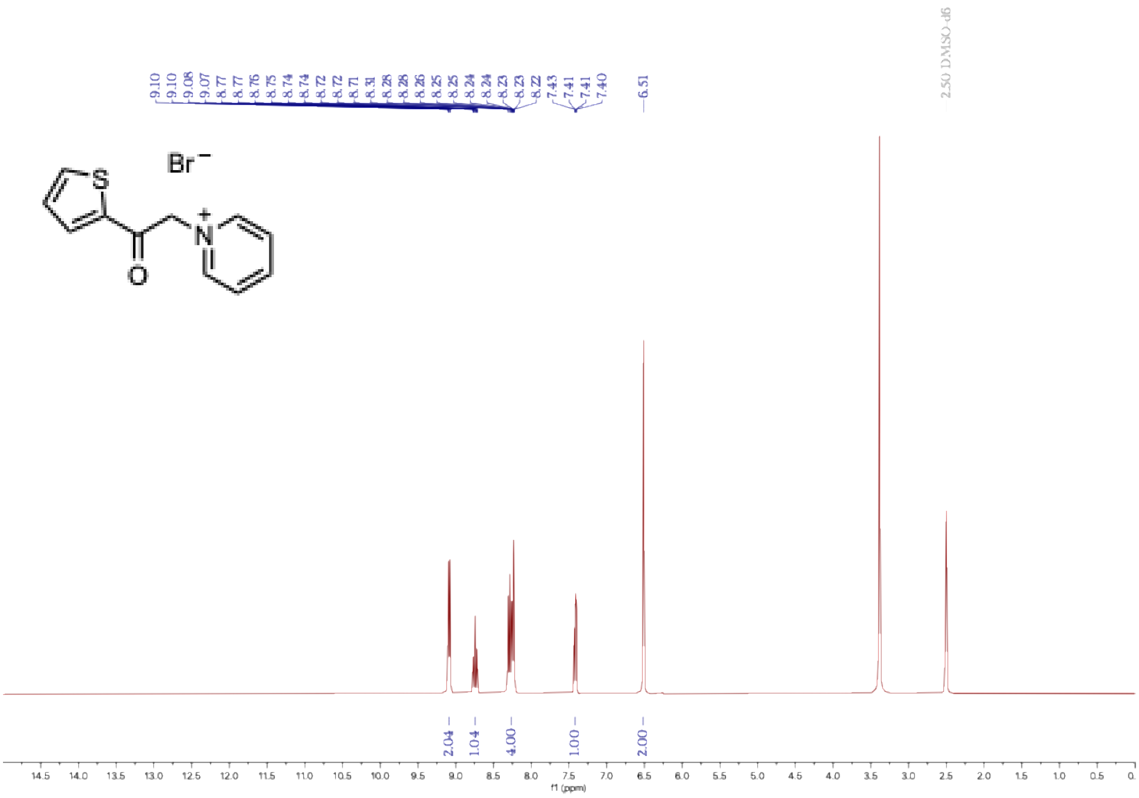

Figure S149 – ^{1}H-NMR (300 MHz, DMSO-*d$_6$*) of pyridinium salt **2o**.

1-[2-oxo-2-(2-oxo-2H-chromen-3-yl)ethyl]pyridin-1-ium bromide (2p)

Following the general procedure using 3-(2-bromoacetyl)-2H-chromen-2-one (3.12 mmol, 833.3 mg) in 13 mL of pyridine. After filtration, the compound **2p** (3.04 mmol, 1.06 g, 98% yield) was obtained.

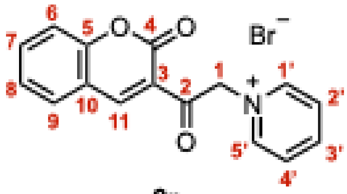

2p

Analytical data according with literature.[28]

^{1}H NMR (300 MHz, DMSO-*d$_6$*): δ 8.98 (s, H-11, 1H), 8.95 (dd, *J* = 6.6, 1.4 Hz, H-1' and 5', 2H), 8.72 (t, *J* = 7.9 Hz, H-3', 1H), 8.26 (dd, *J* = 7.9, 6.6 Hz, H-2' or 4', 2H), 8.09 (dd, *J* = 7.8, 1.6 Hz, H-6 or 9, 1H), 7.87 (ddd, *J* = 8.8, 7.3, 1.6 Hz, H-7 or 8, 1H), 7.59 (d, *J* = 8.3 Hz, H-6 or 9, 1H), 7.50 (td, *J* = 7.5, 1.1 Hz, H-7 or 8, 1H), 6.31 (s, H-1, 2H) ppm (Figure S150).

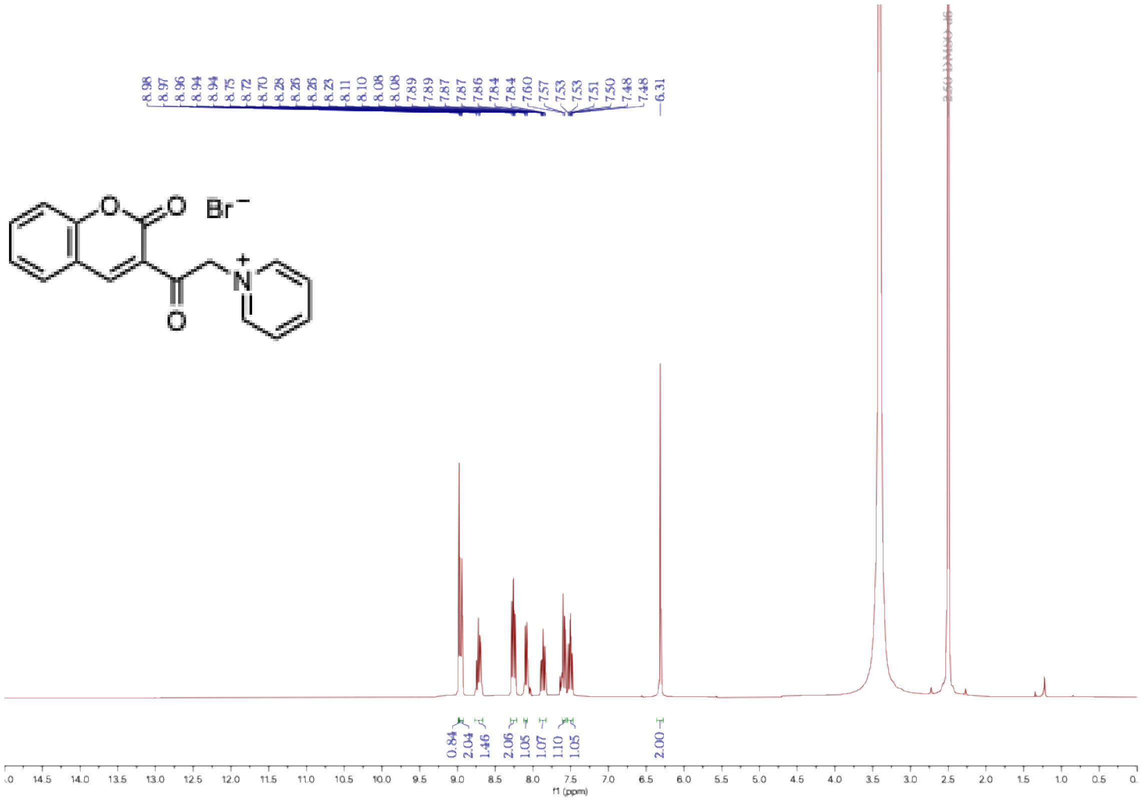

Figure S150 – ^{1}H-NMR (300 MHz, DMSO-*d$_6$*) of pyridinium salt **2p**.

1-[2-(3,4-dihydroxyphenyl)-2-oxoethyl]pyridin-1-ium chloride (2q)

Following the general procedure using 2-chloro-1-(3,4-dihydroxyphenyl)ethan-1-one (2.50 mmol, 466.5 mg) in 10 mL of pyridine. After filtration, the compound **2q** (2.19 mmol, 581.1 mg, 87% yield) was obtained.

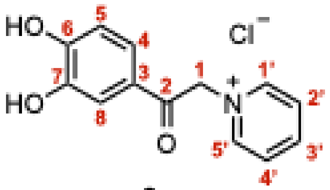

2q White solid. Melting point of 279.2–281.5 °C.

^{1}H NMR (300 MHz, DMSO-*d_6*): δ 10.51 (s, 6-O*H* or 7-O*H*, 1H), 9.76 (s, 6-O*H* or 7-O*H*, 1H), 9.09 – 9.02 (m, H-1' and 5', 2H), 8.71 (td, *J* = 7.8, 1.4 Hz, H-3', 1H), 8.25 (dd, *J* = 7.9, 6.6 Hz, H-2' and 4', 2H), 7.49 – 7.44 (m, H-5 and 8, 2H), 7.06 – 7.01 (m, H-4, 1H), 6.45 (s, 2H) ppm (Figure S151).

^{13}C NMR (75 MHz, DMSO-*d_6*): δ 188.7 (C-2), 152.4 (C-6 or C-7), 146.4 (C-1' and 5', 2C), 146.2 (C-3'), 145.8 (C-6 or C-7), 127.7 (C-2' and 4', 2C), 125.1 (C-3), 121.8 (C-5 or 8), 115.7 (C-4), 115.2 (C-5 or 8), 65.8 (C-1) ppm (Figure S152).

HRMS (ESI) *m/z*: [M]$^+$ Calcd for $C_{13}H_{12}NO_3^+$ 230.0812 ; Found 230.0823.

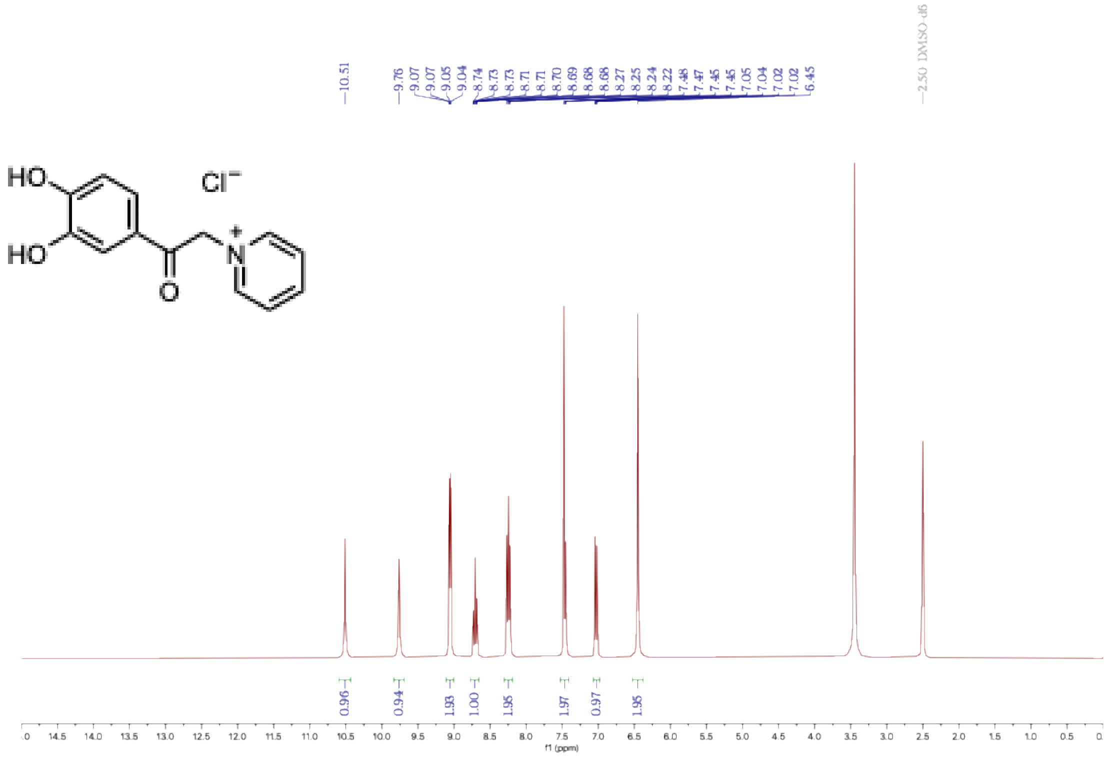

Figure S151 – ^{1}H-NMR (300 MHz, DMSO-*d_6*) of pyridinium salt **2q**.

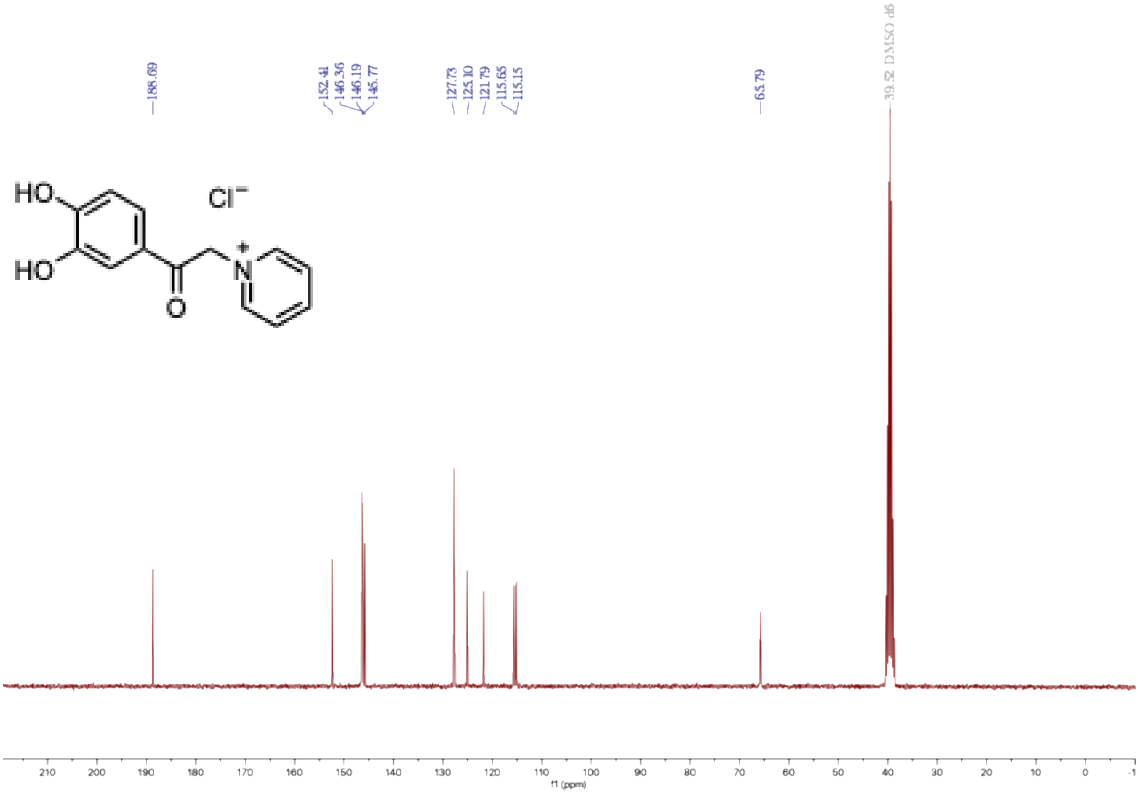

Figure S152 – ^{13}C-NMR (75 MHz, DMSO-d_6) of pyridinium salt **2q**.

11. References

[1] M. J. Frisch, G. W. Trucks, H. B. Schlegel, G. E. Scuseria, M. A. Robb, J. R. Cheeseman, G. Scalmani, V. Barone, G. A. Petersson, H. Nakatsuji, X. Li, M. Caricato, A. V. Marenich, J. Bloino, B. G. Janesko, R. Gomperts, B. Mennucci, H. P. Hratchian, J. V. Ortiz, A. F. Izmaylov, J. L. Sonnenberg, Williams, F. Ding, F. Lipparini, F. Egidi, J. Goings, B. Peng, A. Petrone, T. Henderson, D. Ranasinghe, V. G. Zakrzewski, J. Gao, N. Rega, G. Zheng, W. Liang, M. Hada, M. Ehara, K. Toyota, R. Fukuda, J. Hasegawa, M. Ishida, T. Nakajima, Y. Honda, O. Kitao, H. Nakai, T. Vreven, K. Throssell, J. A. Montgomery Jr., J. E. Peralta, F. Ogliaro, M. J. Bearpark, J. J. Heyd, E. N. Brothers, K. N. Kudin, V. N. Staroverov, T. A. Keith, R. Kobayashi, J. Normand, K. Raghavachari, A. P. Rendell, J. C. Burant, S. S. Iyengar, J. Tomasi, M. Cossi, J. M. Millam, M. Klene, C. Adamo, R. Cammi, J. W. Ochterski, R. L. Martin, K. Morokuma, O. Farkas, J. B. Foresman, D. J. Fox, Wallingford, CT, **2016**.

[2] L. R. W. J. Hehre, P. v. R. Schleyer, J. Pople, *Ab initio molecular orbital theory*, John Wiley & Sons, New York, **1986**.

[3] R. G. Parr;, W. Yang, *Density Functional Theory of Atoms and Molecules*, Oxford University Press, New York, **1989**.

[4] aJ. P. Perdew, *Phys. Rev. B* **1986**, *33*, 8822-8824; bJ. P. Perdew, K. Burke, M. Ernzerhof, *Phys. Rev. Lett.* **1997**, *78*, 1396-1396.

[5] aR. Ditchfield, W. J. Hehre, J. A. Pople, *J. Chem. Phys.* **1971**, *54*, 724-728; bW. J. Hehre, R. Ditchfield, J. A. Pople, *J. Chem. Phys.* **1972**, *56*, 2257-2261; cP. C. Hariharan, J. A. and Pople, *Mol. Phys.* **1974**, *27*, 209-214; dM. S. Gordon, *Chem. Phys. Lett.* **1980**, *76*, 163-168; eP. C. Hariharan, J. A. Pople, *Theor. Chim. Acta* **1973**, *28*, 213-222.

[6] aC. Peng, P. Y. Ayala, H. B. Schlegel, M. J. Frisch, *J. Comput. Chem.* **1996**, *17*, 49-56; bC. Peng, H. Bernhard Schlegel, *Isr. J. Chem.* **1993**, *33*, 449-454.

[7] aJ. E. Carpenter, F. Weinhold, *J. Mol. Struct. Theochem.* **1988**, *169*, 41-62; bJ. E. Carpenter, University of Wisconsin (Madison WI), **1987**; cJ. P. Foster, F. Weinhold, *J. Am. Chem. Soc.* **1980**, *102*, 7211-7218; dA. E. Reed, F. Weinhold, *J. Chem. Phys.* **1983**, *78*, 4066-4073; eA. E. Reed, F. Weinhold, *J. Chem. Phys.* **1985**, *83*, 1736-1740; fA. E. Reed, R. B. Weinstock, F. Weinhold, *J. Chem. Phys.* **1985**, *83*, 735-746; gA. E. Reed, L. A. Curtiss, F. Weinhold, *Chem. Rev.* **1988**, *88*, 899-926; hF. Weinhold;, J. E. Carpenter, *The Structure of Small Molecules and Ions*, Plenum Press, New York, **1988**.

[8] aK. B. Wiberg, *Tetrahedron* **1968**, *24*, 1083-1096; bM.-L. Yao, M. S. Reddy, W. Zeng, K. Hall, I. Walfish, G. W. Kabalka, *J. Org. Chem.* **2009**, *74*, 1385-1387.

[9] aE. Cancès, B. Mennucci, J. Tomasi, *J. Chem. Phys.* **1997**, *107*, 3032-3041; bB. Mennucci, J. Tomasi, *J. Chem. Phys.* **1997**, *106*, 5151-5158; cM. Cossi, V. Barone, B. Mennucci, J. Tomasi, *Chem. Phys. Lett.* **1998**, *286*, 253-260.

[10] A. V. Marenich, C. J. Cramer, D. G. Truhlar, *J. Phys. Chem. B* **2009**, *113*, 6378-6396.

[11] K. L. Manasa, Y. Tangella, N. H. Krishna, M. Alvala, *Beilstein J. Org. Chem.* **2019**, *15*, 1864-1871.

[12] H. S. Sim, H. D. Khanal, Y. R. Lee, *J. Org. Chem.* **2022**, *87*, 12890-12899.

[13] D. Bandyopadhyay, S. Mukherjee, B. K. Banik, *Molecules* **2010**, *15*, 2520-2525.

[14] L. Garel, L. Saint-Jalmes, *Tetrahedron Lett.* **2006**, *47*, 5705-5708.

[15] B. Lv, Y. Feng, J. Dong, M. Xu, B. Xu, W. Zhang, Z. Sheng, A. Welihinda, B. Seed, Y. Chen, *ChemMedChem* **2010**, *5*, 827-831.
[16] C. Wang, Y.-B. Yu, S. Fan, X. Zhang, *Org. Lett.* **2013**, *15*, 5004-5007.
[17] P. Gobbo, W. Luo, S. J. Cho, X. Wang, M. C. Biesinger, R. H. E. Hudson, M. S. Workentin, *Org. Biomol. Chem.* **2015**, *13*, 4605-4612.
[18] J.-W. Zhao, J.-W. Guo, M.-J. Huang, Y.-Z. You, Z.-H. Wu, H.-M. Liu, L.-H. Huang, *Steroids* **2019**, *150*, 108431.
[19] aL. Yang, Y. Zhang, X. Zou, H. Lu, G. Li, *Green Chem.* **2018**, *20*, 1362-1366; bE. Bremus-Köbberling, A. Gillner, F. Avemaria, C. Réthoré, S. Bräse, *Beilstein J. Org. Chem.* **2012**, *8*, 1213-1218.
[20] B. Baur, K. Storch, K. E. Martz, M. I. Goettert, A. Richters, D. Rauh, S. A. Laufer, *J. Med. Chem.* **2013**, *56*, 8561-8578.
[21] H. Chai, K. Le Mai Hoang, M. D. Vu, K. Pasunooti, C.-F. Liu, X.-W. Liu, *Angew. Chem. Int. Ed.* **2016**, *55*, 10363-10367.
[22] J. T. Manka, F. Guo, J. Huang, H. Yin, J. M. Farrar, M. Sienkowska, V. Benin, P. Kaszynski, *J. Org. Chem.* **2003**, *68*, 9574-9588.
[23] S. Sathish Kumar, F. M., S. K. S., K. S., G. S. Mahasampath, H. S., K. K. and Balasubramanian, *Synth. Commun.* **2018**, *48*, 553-560.
[24] A. Nohara, T. Umetani, Y. Sanno, *Tetrahedron* **1974**, *30*, 3553-3561.
[25] D. S. Allgäuer, H. Mayr, *Eur. J. Org. Chem.* **2013**, 6379-6388.
[26] X. He, R. Li, P. Y. Choy, T. Liu, J. Wang, O. Y. Yuen, M. P. Leung, Y. Shang, F. Y. Kwong, *Org. Lett.* **2020**, *22*, 9444-9449.
[27] R. R. Castillo, C. Burgos, J. J. Vaquero, J. Alvarez-Builla, *Eur. J. Org. Chem.* **2011**, 619-628.
[28] D. I. Brahmbhatt, C. V. Patel, V. G. Bhila, N. H. Patel, A. A. Patel, *Med. Chem. Res.* **2015**, *24*, 1596-1604.
[29] M. Gianini, A. v. Zelewsky, *Synthesis* **1996**, 702-706.
[30] Y. Zhang, W. Fan, Y. Li, D. Huang, *J. Org. Chem.* **2023**, *88*, 12244-12250.
[31] M. Costa, A. I. Rodrigues, F. Proença, *Tetrahedron* **2014**, *70*, 4869-4875.
[32] B. Febriansyah, C. S. D. Neo, D. Giovanni, S. Srivastava, Y. Lekina, T. M. Koh, Y. Li, Z. X. Shen, M. Asta, T. C. Sum, N. Mathews, J. England, *Chem. Mater.* **2020**, *32*, 4431-4441.
[33] D. Lapointe, T. Markiewicz, C. J. Whipp, A. Toderian, K. Fagnou, *J. Org. Chem.* **2011**, *76*, 749-759.
